# Palladium-Catalyzed *trans*-Hydroalkoxylation: Counterintuitive Use of an Aryl Iodide Additive to Promote C–H Bond Formation

**DOI:** 10.1021/acscatal.2c01809

**Published:** 2022-06-13

**Authors:** Ashis Das, Luca Buzzetti, Mikus Puriņš, Jerome Waser

**Affiliations:** Laboratory of Catalysis and Organic Synthesis and NCCR Catalysis, Institute of Chemical Sciences and Engineering, Ecole Polytechnique Fédérale de Lausanne, EPFL SB ISIC LCSO, BCH 1402, 1015 Lausanne, Switzerland

**Keywords:** enantioselective catalysis, palladium catalysis, hydrogenation, chiral auxiliary, amino alcohols, tethers, dynamic kinetic asymmetric transformation

## Abstract

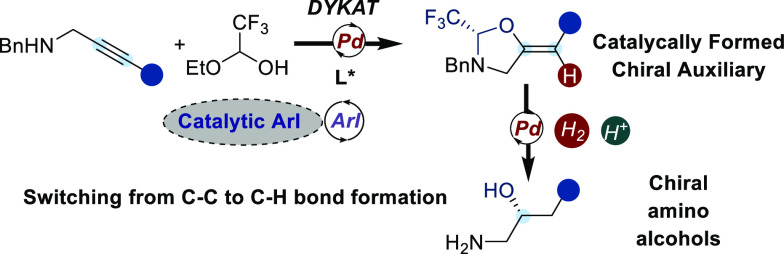

We report an enantioselective palladium-catalyzed *trans*-hydroalkoxylation of propargylic amines with a trifluoroacetaldehyde-derived tether to build chiral oxazolidines. Diastereoselective hydrogenation using a heterogeneous palladium catalyst then gave access to protected benzylic amino alcohols in 45–87% yields and 84–94% ee values. Hydroalkoxylation of the alkynes required a catalytic amount of aryl iodide, highlighting the counterintuitive key role played by a putative Pd(II)/ArI oxidative addition complex to promote oxypalladation/protodemetalation.

The efficient preparation of enantioenriched molecules is a longstanding challenge for catalysis.^1^ Enantiomers have different bioactivities, and access to enantiopure drugs is therefore needed.^2^ As part of these efforts, our group recently reported a new strategy for accessing chiral molecules based on the catalytic formation of chiral auxiliaries (Scheme 1A).^3^ In a three-component reaction, a palladium-catalyzed dynamic kinetic asymmetric transformation (DYKAT)^4^ rapidly led to chiral oxazolidine intermediate **3** on starting from propargylic amine **1**, an aryl iodide, and the trifluoroacetaldehyde-derived tether **2**.^5^ The trifluoromethyl group then efficiently blocked one face of the alkene, leading to a diastereoselective hydrogenation to give enantioenriched protected diaryl amino alcohols **4**. It could be also used to control other processes, such as epoxidation and cyclopropanation.^3b^ Amino alcohols are key building blocks in synthetic and medicinal chemistry.^6^ In this approach, we combined the advantage of using only a catalytic amount of the enantiopure material with the robust selectivity control being ensured by covalently bound auxiliaries.

**Scheme 1:**
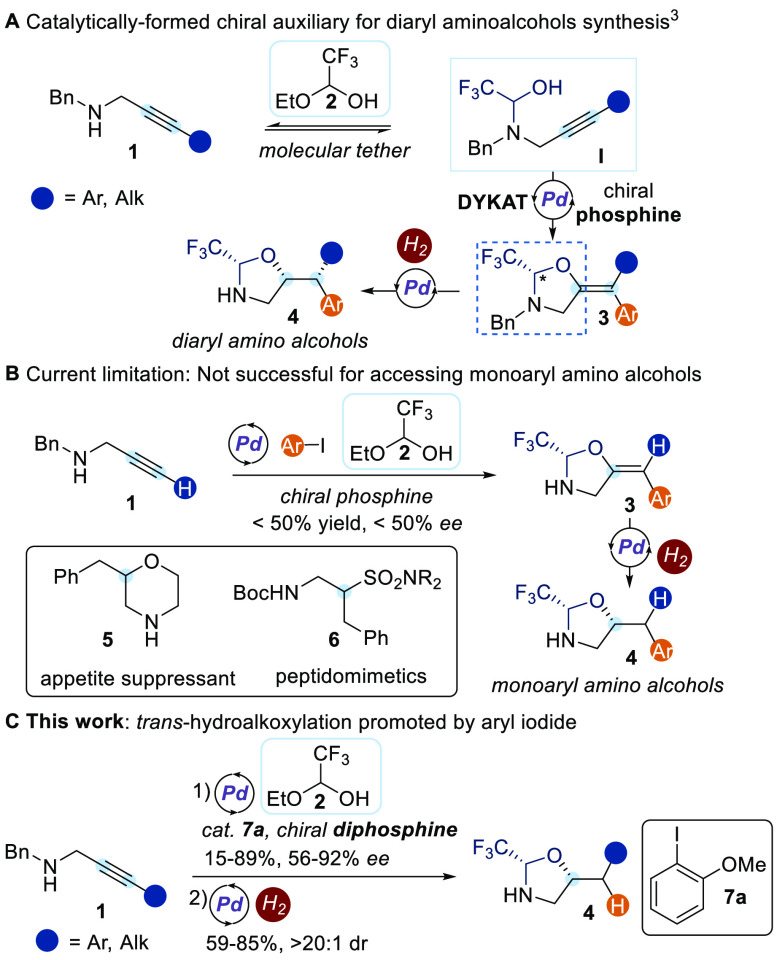
Synthesis of Amino Alcohols via a Catalytically Formed Chiral Auxiliary

A current limitation of our methodology is that it failed to give good enantioinduction and yield for terminal alkynes (Scheme 1B). The corresponding protected amino alcohols **4** bearing a single aryl group obtained upon diastereoselective hydrogenation have found widespread applications in the synthesis of pharmacologically relevant molecules,^7^ including the appetite suppressant (*R*)-2-benzylmorpholine (**5**)^7a^ and the α-substituted aminoethane sulfonamides **6**,^7b^ used in the preparation of peptidomimetics. Their asymmetric synthesis is limited to multistep procedures,^7,8^ relying on building blocks available in the chiral pool, with the exception of one strategy based on a Sharpless asymmetric epoxidation to forge the key stereocenter.^7a^

In order to access this important subclass of amino alcohols, we envisioned a new catalytic process via hydroalkoxylation of the triple bond instead of the arylalkoxylation. For it to be successful, a catalyst will need to be designed to promote C–H bond formation via protodemetalation, which had been observed only as a minor side reaction in our previous studies.

Herein, we report the first enantioselective palladium-catalyzed *trans*-hydroalkoxylation of propargylic amines via *in situ* tethering (Scheme 1C). The key for success was the counterintuitive use of a catalytic amount of aryl iodide **7a** as additive together with a commercially available chiral diphosphine ligand to promote oxypalladation/protodemetalation instead of oxypalladation/reductive elimination. Diastereoselective hydrogenation under standard heterogeneous conditions then gave access to monoaryl amino alcohol derivatives in high yield and stereoselectivity. Fine-tuning of the structure of aryl iodide **7** was essential to promote the desired transformation.

In our previous work,^3^ an interesting result was obtained for the tethered oxyarylation of propargylic amine **1a** when DACH-phenyl Trost diphosphine ligand **L1**(9) and Pd_2_(dba)_3_·CHCl_3_ as the palladium source were used.^10^ The desired oxyarylation product **3a′** was obtained in only 66% yield and 66% ee, but the protodemetalation product **3a** was observed in 29% yield and 96% ee (Scheme 2).

**Scheme 2:**
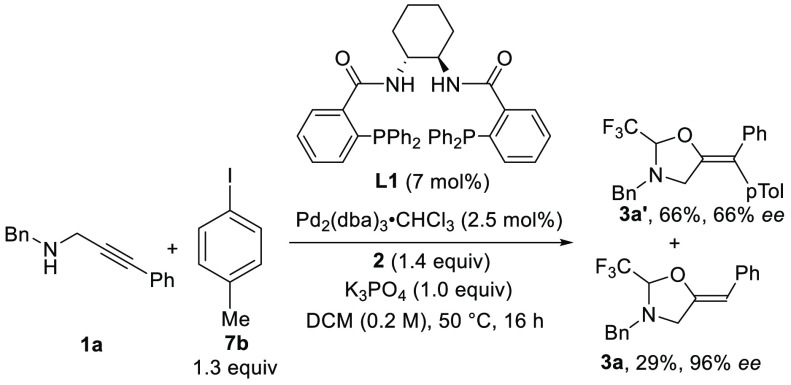
Preliminary Result Obtained with DACH-phenyl Trost Ligand **L1** in the Alkoxyarylation of Propargylamine **1a**

We therefore decided to optimize the *trans*-hydroalkoxylation process as an alternative to the failed alkoxyarylation of terminal alkynes (Table 1). The first obvious experiment was to remove aryl iodide **7b**, as it should not be needed for the transformation (entry 1). Surprisingly, no product **3a** was formed and we only recovered the starting materials. This result indicated that a Pd–Ar complex may be necessary to promote the hydroalkoxylation step. In fact, when a catalytic amount (20 mol %) of iodobenzene (**7c**) was added, product **3a** was obtained in 23% yield and 94% ee (entry 2). In addition, we also observed the formation of the arylated product in about 20% yield. The role of the aryl iodide is not only to oxidize palladium, as the use of Pd(II) catalysts in its absence did not provide **3a** (entry 3). Instead, we recovered only the tethered starting material. When the monophosphine ligand **L2**,^11^ which gave the best results in our previous work,^3^ was used, **3a** was obtained only in 13% yield and 38% ee (entry 4). We then investigated the effect of substitution on the arene ring. 2-Iodotoluene (**7d**) provided product **3a** in 27% yield and 86% ee (entry 5). 2-Iodobenzotrifluoride (**7e**) delivered **3a** in 30% yield and 76% ee (entry 6), while 2-iodoanisole (**7a**) gave **3a** in good yield (90%) and enantioselectivity (92%) (entry 7). When the methoxy group was substituted with a fluoro group (**7f**), **3a** was obtained in 90% yield and 86% ee (entry 8), while the large *tert*-butyldimethylsilyloxy-substituted aryl iodide **7g** gave **3a** in just 9% yield and 64% ee (entry 9). With a methoxy group in the *para* position (**7h**), **3a** was formed only in 14% yield with 89% ee (entry 10). From these results, it is apparent that *ortho* substitution with a small potentially coordinating group is beneficial for the yield but has only a slight influence on the enantioselectivity. The DACH-phenyl Trost ligand **L1** was the best ligand. Other ligands (entries 11 and 12), including (*R*)-SIPHOS-PE (**L3**) and (*R*)-MOP (**L4**), delivered **3a** in lower yields (50% and 80%, respectively) as a racemate. In more “industrially preferred” solvents such as toluene (entry 13) and ethyl acetate (entry 14), the yield and enantioselectivity were lower. Finally, the reaction could be scaled up to 0.4 mmol, reducing the catalyst and the ligand loading to 1.25 and 3.5 mol %, respectively, to give a similar yield and stereoselectivity (entry 15).

**Table 1:**
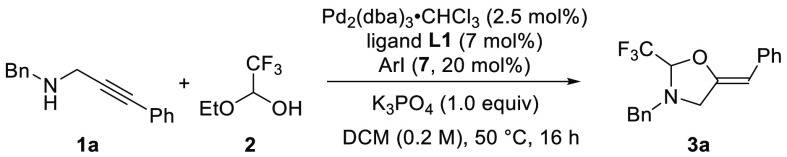
Optimization of the Formation of Oxazolidine **3a**^a^

entry	deviation from conditions	yield (%)^b^^,^^c^	ee (%)
1	no **7b**	<5	
2	**7c**	23	94
3	no **7**, PdCl_2_, Pd(OAc)_2_, PdI_2_, or Pd[MeCN]_4_(BF_4_)_2_	<5	
4	**7c**, **L2** instead of **L1**	13	38
5	**7d**	27	86
6	**7e**	30	76
7	**7a**	90	92
8	**7f**	90	86
9	**7g**	9	64
10	**7h**	14	89
11	**L3** instead of **L1**	50	<5
12	**L4** instead of **L1**	80	<5
13	toluene instead of DCM	>95	80
14	ethyl acetate instead of DCM	50	85
15	**7a**, **L1**, 0.4 mmol scale^d^	83	90

a Reaction conditions: 0.1 mmol of **1** (1 equiv), **2** (1.4 equiv), ligand (7 mol %), K_3_PO_4_ (1.0 equiv), ArI **7** (20 mol %), and Pd catalyst (2.5 mol %) in 0.5 mL of solvent unless specified otherwise.
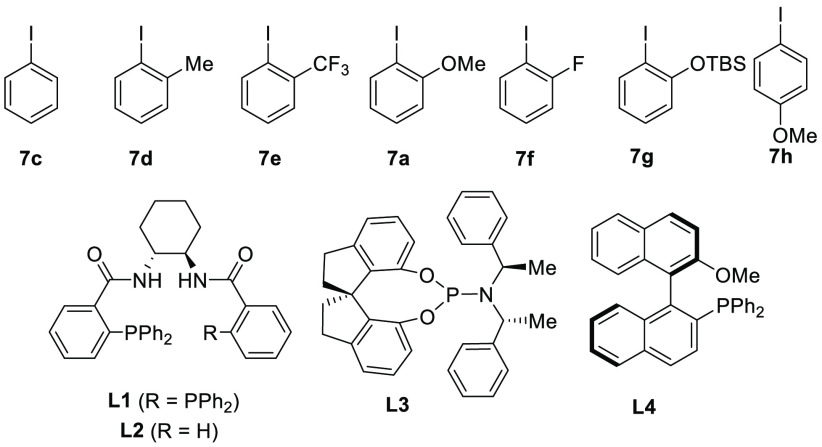

b ^1^H NMR yields were determined by addition of 1 equiv of trichloroethylene as an internal standard after the reaction.

c Arylation products were obtained in up to 20% yield. See the Supporting Information for details.

d Reaction performed using 1.25 mol % of Pd_2_(dba)_3_·CHCl_3_ and 3.5 mol % of ligand.

We then evaluated the scope of the transformation (Scheme 3). Aryl propargylic amines, prepared in a single step from the terminal alkyne (see the Supporting Information),^12^ gave access to the corresponding trisubstituted olefins bearing the chiral oxazolidine auxiliary in good yield and stereoselectivity. On the *para* position of the aryl ring, both electron-rich and electron-poor substituents were tolerated and the products **3b**–**d** and **3e**–**l** were obtained in 72–87% yields and 84–94% ee values.

**Scheme 3:**
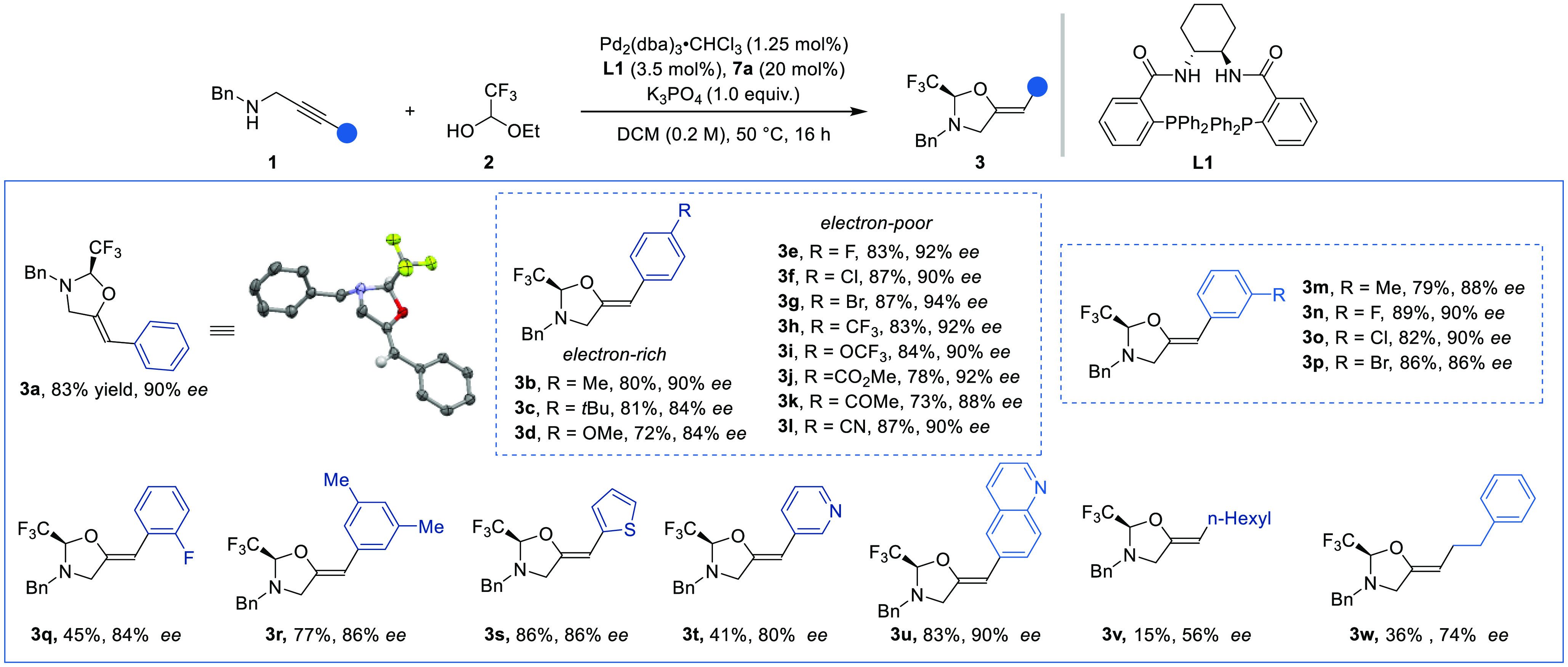
Scope of the Enantioselective Hydroalkoxylation Reactions performed on a 0.4 mmol scale using 0.2 equiv of aryl iodide **7a** and 1.4 equiv of 1-ethoxy trifluoroethanol (**2**). Isolated yields and HPLC enantiomeric excess are given.

The functional group tolerance included halogens (**3e**–**i**) and even a potentially Pd(0) sensitive bromine (**3g**), an ester (**3j**), a ketone (**3k**), and a cyanide (**3l**). *meta*-substituted products **3m**–**p** were obtained in 79–89% yields and 86–90% ee values. The reaction was more sluggish with substituents in an *ortho* position, and only product **3q** bearing a small fluorine group could be isolated in 45% yield and 84% ee. The disubstituted product **3r** was obtained in 77% yield and 86% ee.

The reaction tolerated heterocycles such as thiophene (**3s**), pyridine (**3t**), and quinoline (**3u**) on the alkyne. Propargylic amines with alkyl substituents on the alkyne delivered products **3v**,**w** in lower yield and enantioselectivity. To evaluate the scalability of this protocol, the reaction on propargylic amine **1a** was performed on a 3 mmol scale and gave an 82% yield of **3a** without loss of the optical purity. The absolute configuration of the products was assigned by an X-ray crystallographic analysis of **3a**, confirming the *Z* geometry of the double bond.

We then examined the stereoselective hydrogenation directed by the installed chiral oxazolidine. We submitted alkene **3a** to hydrogenation with Pearlman’s catalyst.^13^ Under these conditions, we could access the reduced and benzyl-deprotected product **4a** in 85% yield and 90% ee with perfect diastereoselectivity and retention of the enantiopurity (Scheme 4). Substitution at the *para* (**4a**–**j**), *meta* (**4m**,**n**,**r**), and *ortho* (**4q**) positions of the arene was well tolerated, as were different electronic properties. However, chlorine-, bromine-, and heterocycle-containing olefins did not deliver the hydrogenation products. An ester was well tolerated and gave product **4j** in 82% yield, while ketone **3k** and nitrile **3l** were further reduced to the corresponding alcohol **4k** and amine **4l**. The hydrogenation of **3a** proceeded on a 1 mmol scale without any loss of stereoselectivity. The deprotection of the trifluoroacetal group on **4a** could be easily performed with toluenesulfonic acid to give deprotected amino alcohol **8** in 74% yield.

**Scheme 4:**
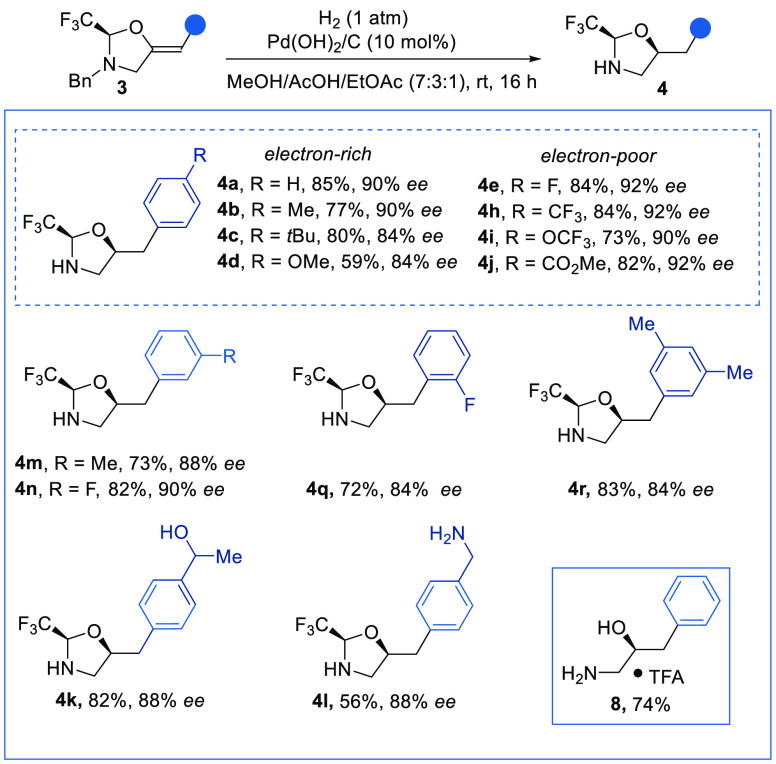
Scope of the Stereoselective Hydrogenation Reactions performed on a 0.2 mmol scale using Pd(OH)_2_/C (∼20 wt %). Isolated yields and HPLC enantiomeric excess are given. Product **11** was obtained after treating **4a** with TsOH·H_2_O in a 2/1 THF/H_2_O mixture at room temperature for 16 h; the trifluoroacetate salt was obtained after purification by reverse-phase preparative HPLC.

A speculative reaction mechanism based on literature precedents in palladium catalysis is presented in Scheme 5.^14^ From NMR experiments, we saw a reversible reaction of propargylic amine **1a** with ethoxy trifluoroethanol **2** to produce hemiaminal **I**.^3^ The catalytic cycle is most probably initiated by oxidative addition of ArI on Pd(0) complex **II** to give Pd(II) complex **III**. Reaction with **I** can then occur either via *syn*- or *anti*-palladation,^15^ both being well established.^16^ Both pathways would require decoordination of the X ligand (most probably iodide) on palladium, to enable either coordination of the alkyne for *anti*-palladation (**IV** to **VII**) or coordination of the oxygen for *syn*-palladation (**V** to **VI**). As the geometry of product **3a** indicates that protodemetalation is occurring from *trans*-palladation complex **VII**, an isomerization of *cis*-palladation complex **VI** would be required to explain the formation of the product in case of *syn*-palladation. Although rare, similar isomerizations have been proposed.^17^ In case of **VI**, it could be facilitated by the donating effect of the oxygen atom. From **VII**, protodemetalation then gives product **3a** and regenerates Pd(II) complex **III**. Alternatively, reductive elimination would lead to tetrasubstituted product **3a′**. As oxypalladation can be reversible, it is not clear if the dynamic kinetic resolution process of **I** would occur at this step or only at the stage of isomerization/reductive elimination.

**Scheme 5:**
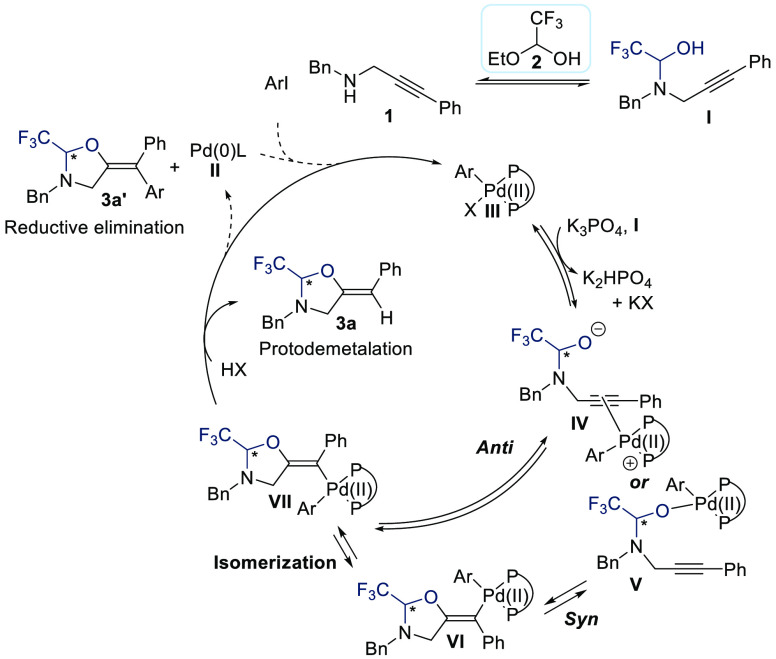
Speculative Catalytic Cycles

^31^P{^1^H} NMR studies first confirmed the formation of a Pd(0)dba diphosphine (**L1**) complex, as reported in the literature.^18^ When *o*-iodoanisole **7a** was added to the Pd(0)**L1**·dba species, an immediate reaction was observed with the appearance of two new signals in the NMR (see section E in the Supporting Information). However, the exact structure of this species remains unclear, as the NMR data does not match the reported spectra of Pd oxidative addition complexes with bidentate phosphine ligands.^19^ With regard to the promotion of the reaction by the aryl iodide additive, it would be difficult to understand why more electrophilic palladium salts such as PdCl_2_, Pd(OAc)_2_, PdI_2_, and Pd[MeCN]_4_(BF_4_)_2_ would fail in the oxypalladation step. Therefore, the aryl ligand may be important to accelerate the protodemetalation step by increasing the electron density on palladium. The potentially coordinating small *ortho* substituent in **7a**,**f** may play a role in promoting protodemetalation over reductive elimination. More in-depth mechanism studies are needed to elucidate the reaction mechanism and propose a model for stereoinduction and additive effects.

In conclusion, we have developed a palladium-catalyzed hydroalkoxylation of propargylic amines based on *in situ* tether formation. After diastereoselective hydrogenation directed by the catalytically formed chiral oxazolidine auxiliary, valuable enantioenriched amino alcohol precursors were obtained. The key for success in the hydroalkoxylation reaction was the use of an *ortho*-substituted aryl iodide as an additive. Currently, this effect is not well understood and mechanistic investigations will be the topic of future work. The discovery of the importance of aryl palladium oxidative addition complexes in promoting alkyne functionalization and protodemetalation has nevertheless already set the basis for the development of new catalytic processes.

## Supporting Information Available

Experimental procedures and analytical data for all new compounds (PDF)

## Notes

Raw data for NMR, IR and HPLC is available free of charge from Zenodo.org: https://doi.org/10.5281/zenodo.6634788.
