# Supplementary material for: Palladium-Catalyzed trans-Hydroalkoxylation: Counterintuitive Use of an Aryl Iodide Additive to Promote C–H Bond Formation
Source: ACS Catal. 2022 Jun 13;12(13):7565–70. doi: 10.1021/acscatal.2c01809 (PMC9251722; doi:10.1021/acscatal.2c01809)
Supplement: Supplementary file 1 — cs2c01809_si_001.pdf [file cs2c01809_si_001.pdf]

Supporting Information for

"Palladium-Catalyzed *trans*-Hydroalkoxylation: Counterintuitive Use of an Aryl Iodide Additive to Promote C-H Bond Formation"

Ashis Das,[1,2†] Luca Buzzetti,[1,3†] Mikus Puriņš[1] and Jerome Waser[1]*

Laboratory of Catalysis and Organic Synthesis and NCCR Catalysis, Institute of Chemical Sciences and Engineering, Ecole Polytechnique Fédérale de Lausanne, EPFL SB ISIC LCSO, BCH 1402, 1015 Lausanne, Switzerland.

†These authors contributed equally to this work.

[2]Present address: Sygnature discovery, Biocity, Pennyfoot Street, Nottingham, NG1 1GR, United Kingdom. Email: ashisdas28394@gmail.com

[3]Present address: FIS-Fabbrica Italiana Sintetici, viale Milano 26, 36075, Montecchio Maggiore (Italy) Email: luca.buzzetti@fisvi.com

*Correspondence to: jerome.waser@epfl.ch

Table of Contents

A. GENERAL INFORMATION 3

B. SYNTHESIS OF THE STARTING MATERIALS 4

B.1. Synthesis of the Propargylic Amines Precursors 9 4
B.2. Synthesis of the Propargylic Amines 5

C. OPTIMIZATION STUDIES 15

C.1. Cyclization: Screening of ArI (1.4 eqv) 15
C.2. Cyclization: Stoichiometry of ArI (o-MeO) 15
C.3. Cyclization: Ligand Screening 16
C.4. Cyclization: Screening of Solvents and Temperatures 16
C.5. Cyclization: Stoichiometry of K_3PO_4 17
C.6. Asymmetric Hydrogenation: Optimization Studies 18

D. STEREOSELECTIVE TETHERED CYCLIZATION OF PROPARGYLIC AMINES 19

D.1. General Procedure for the Enantioselective Cyclization of Propargylic Amines 19
D.2. Characterization of Products of the Enantioselective Cyclization of Propargyl amines 19
D.3. General Procedure for the Asymmetric Hydrogenation of the Trisubstituted Olefins. 28
D.4. Characterization of Hydrogenated Products 29
D.5. Unsuccessful Substrates 35

E. NMR STUDIES 36

F. X-RAY CRYSTALLOGRAPHIC DATA 40

F.1. Single Crystal X-Ray Diffraction for the chiral compound *(S)*-3a 40
F.2. Single Crystal X-Ray Diffraction for the chiral compound 4b 42

G. REFERENCES 44

H. HPLC SPECTRA 46

I. NMR SPECTRA 83

A. General Information

The NMR spectra were recorded on a Brucker DPX-400 spectrometer at 400 MHz for ^{1}H, 101 MHz for ^{13}C, 376 MHz for ^{19}F and 162 MHz for ^{31}P. The chemical shift (δ) for ^{1}H and ^{13}C are given in ppm relative to residual signals of the solvents (chloroform-d - 7.26 ppm ^{1}H NMR and 77.16 ppm ^{13}C NMR; methanol-d4 3.31 ppm ^{1}H NMR and 49.0 ppm ^{13}C NMR; dmso-d6 2.50 ppm ^{1}H NMR and 39.52 ppm ^{13}C NMR). Carbon spectra have been measured using broadband {^{1}H} decoupling. Coupling constants are given in Hertz. The following abbreviations are used to indicate the multiplicity: s, singlet; d, doublet; q, quartet; m, multiplet; bs, broad signal; app, apparent. Infrared spectra were recorded on a JASCO FT-IR B4100 spectrophotometer with an ATR PRO410-S and a ZnSe prisma and are reported as cm^{-1} (w = weak, m = medium, s = strong, br = broad). High resolution mass spectrometric measurements were performed by the mass spectrometry service of ISIC at the EPFL on a MICROMASS (ESI) Q-TOF Ultima API. The raw data obtained from the Q-TOF Waters instrument does not take into account the mass of the electron for the ion, the obtained raw data has been therefore corrected by removing the mass of the electron (5 mDa).
The diffraction data for crystal structures were collected by X-Ray service of ISIC at the EPFL at low temperature using Cu (323) or Mo (520) K_a radiation on a Rigaku SuperNova dual system in combination with Atlas type CCD detector. The data reduction and correction were carried out by *CrysAlis^{Pro}* (Rigaku Oxford Diffraction, release 1.171.40.68a, **2019**). The solutions and refinements were performed by *SHELXT*[1] and *SHELXL*[2], respectively. The crystal structures were refined using full-matrix least-squares based on F^2 with all non-H atoms defined in anisotropic manner. Hydrogen atoms were placed in calculated positions by means of the “riding” model. Yields of isolated products refer to materials of >95% purity as determined by ^{1}H NMR.

The authors are indebted to the team of the research support service of ISIC at EPFL, particularly to the NMR, X-Ray, and the High-Resolution Mass Spectrometry Units.

General Procedures. All reactions were set up under a nitrogen atmosphere in oven-dried glassware using standard Schlenk techniques, unless otherwise stated. Synthesis grade solvents were used as purchased; anhydrous solvents (THF, Et_2O, Toluene, Acetonitrile and DCM) were taken from a commercial SPS solvent dispenser (H_2O content < 10 ppm, *Karl-Fischer* titration). Chromatographic purification of products was accomplished using flash chromatography (FC) on SiliaFlash P60 silica gel (230 - 400 mesh). For thin layer chromatography (TLC) analysis throughout this work, Pre-coated TLC sheets ALUGRAM® Xtra SIL G/UV_{254} were employed, using UV light as the visualizing agent and basic aqueous potassium permanganate ($KMnO_4$) stain solutions, and heat as developing agents. Organic solutions were concentrated under reduced pressure on a Büchi rotatory evaporator.

Determination of Enantiomeric Purity: HPLC analysis on chiral stationary phase was performed on a Agilent Acquity instrument using a Daicel CHIRALPAK IA, IB-N5 and IC chiral columns. The exact conditions for the analyses are specified within the characterization section. HPLC traces were compared to racemic samples prepared by running the reactions using racemic ligands. Absolute values of enantiomeric excesses are reported.

Materials. Most of the starting materials used in this study are commercial and were purchased in the highest purity available from Sigma-Aldrich, Fluka, Alfa Aesar, Fluorochem, Enamine and used as received, without further purifications. $Pd(OH)_2$/C, Pearlman's catalyst was purchased from abcr GmbH (ABCR) as 2.0 g container. Tris(dibenzylideneacetone)dipalladium was purchased from Fluorochem and recrystalised in 200 mg portions following a reported procedure.[3] Deactivated silica gel was prepared by making a slurry of silica gel (230-400 mesh) with 5% Et_3N in pentane solution followed by complete removal of solvent by rotary evaporation until obtaining a free-flowing powder. The synthesis of **1a-b**, **1d-f**, **1h** and **1o-t** has already been described by our group. The procedures are taken from the indicated publication[4] for clarity and to facilitate the reproduction of the results.

B. Synthesis of the Starting Materials

B.1. Synthesis of the Propargylic Amines Precursors 9

N-Benzylprop-2-yn-1-amine (9)

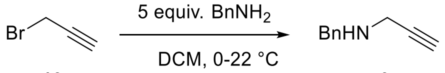

10 **9**

Scheme 1. Synthesis of Benzyl Propargyl amine **9**.

To a flame-dried 250 mL two-necked round-bottom flask, benzylamine (55 mL, 0.50 mol, 5.0 equiv.) and DCM (60 mL) were added. The mixture was cooled to 0 °C. Then, *via* an addition funnel, propargyl bromide (80 wt% solution in toluene, 10.8 mL, 100 mmol, 1.0 equiv.) in DCM (40 mL) was added dropwise over 1 hour. The reaction mixture was allowed to reach room temperature and stirred for 5 h. The reaction mixture was filtered through a plug of silica and concentrated *in vacuo* to approx. 100 mbar. The mixture was distilled under reduced pressure to give the *N*-benzylprop-2-yn-1-amine **9** as a colorless oil (7.3 g, 50 mmol, ~90% purity according to ^{1}H NMR (T = 50 – 55 °C, 0.35 mbar).

^{1}H NMR (400 MHz, Chloroform-*d*) δ 7.41 – 7.31 (m, 4H, Ar*H*), 7.31 – 7.24 (m, 1H, ArH), 3.90 (s, 2H, PhC*H*$_2$), 3.44 (d, *J* = 2.4 Hz, 2H, C*H*$_2$C≡CH), 2.28 (t, *J* = 2.4 Hz, 1H, C≡C*H*), 1.49 (s, 1H, N*H*).

^{13}C{^{1}H} NMR (101 MHz, Chloroform-*d*) δ 139.5, 128.52, 128.49, 127.2, 82.2, 71.6, 52.4, 37.4.

Spectral data were consistent with the values reported in literature.[5]

N-Benzyl propynyl trifluoroacetamide (14)

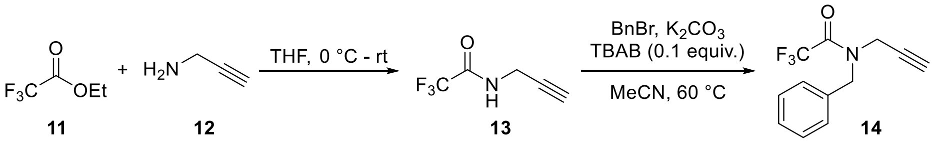

Scheme 2. Synthesis of compound **14**.

Following a modified version of a reported procedure.[6] In a flame dried round-bottom flask, to a solution of ethyl trifluoroacetate **11** (8.0 g, 56 mmol, 1.2 equiv.) in THF (12 mL) at 0 °C was slowly added propargyl amine **12** (2.6 g, 47 mmol, 1 equiv.). The reaction mixture was stirred at 0 °C for 10 minutes; it was then allowed to reach room temperature and stirred for a further 7 hours. The solvent was removed by rotary evaporation and the product was isolated by distillation (90 °C at 17 mbar) to afford propynyl trifluoroacetamide **13** as a colourless oil (5.5 g, 37 mmol, 78% yield).

^{1}H NMR (400 MHz, Chloroform-*d*) δ 6.94 (br. s., 1H, N*H*), 4.14 (dd, *J* = 6.0, 2.5 Hz, 2H, C*H*$_2$C≡C,) 2.32 (q, *J* = 2.2 Hz, 1H, C≡C*H*).

^{13}C NMR (101 MHz, Chloroform-*d*) δ 157.0 (q, *J* = 38.1 Hz), 115.5 (q, *J* = 287.5 Hz), 77.0, 73.1, 29.6.

^{19}F NMR (376 MHz, Chloroform-*d*) δ -76.3.

Spectra data was consistent with the values reported in literature.[6]

To a mixture of K_2CO_3 (8.2 g, 59 mmol, 2 equiv.) and TBAB (0.95 g, 3.0 mmol, 0.1 equiv.) in MeCN (150 mL) was added propynyl trifluoroacetamide **13** (4.5 g, 30 mmol, 1 equiv.) and benzyl bromide (6.0 g, 33 mmol, 1.1 equiv.) and the reaction mixture was stirred at 60 °C. After 3 hours (progress determined by TLC (SiO_2, 20% EtOAc in pentane)), the mixture was filtered through a plug of Celite, which was washed with Et_2O. The resulting filtrate was concentrated by rotary evaporation. Purification of the crude product by column chromatography (SiO_2, 0-8% EtOAc in pentane) afforded *N*-Benzyl propynyl trifluoroacetamide (**14**) as a colourless oil (5.0 g, 21 mmol, 71% yield)

^{1}H NMR (400 MHz, Chloroform-*d*; 1:1.2 mixture of rotamers) δ 7.46 – 7.23 (m, 10H, Ar*H*), 4.79 (s, 2H, C*H*$_2$Ar), 4.77 (s, 2H, C*H*$_2$Ar), 4.12 (d, *J* = 2.5 Hz, 2H, C*H*$_2$C≡C), 4.06 (d, *J* = 2.4 Hz, 2H, C*H*$_2$C≡C), 2.37 (t, *J* = 2.4 Hz, 1H, C≡C*H*), 2.29 (t, *J* = 2.5 Hz, 1H, C≡C*H*).

^{13}C{^{1}H} NMR (101 MHz, Chloroform-*d*; 1:1.2 mixture of rotamers) δ 156.7 (q, *J* = 36.5 Hz, 2×C=O), 134.5, 133.8, 129.1, 129.0, 128.6, 128.6, 128.3, 127.7, 116.4 (q, *J* = 287.9 Hz), 116.3 (q, *J* = 288.1 Hz), 76.6 (overlapping with solvent), 76.5, 73.7, 73.3, 49.7 (q, *J* = 3.6 Hz), 48.7, 35.8 (q, *J* = 4.2 Hz), 34.4.

^{19}F NMR (376 MHz, Chloroform-*d*; 1:1.2 mixture of rotamers) δ -68.5, -69.3.

HRMS (LTQ-Orbitrap) m/z: $[M + H]^+$ Calculated for $C_{12}H_{11}F_3NO^+$ 242.0787; Found 242.0783.

B.2. Synthesis of the Propargylic Amines

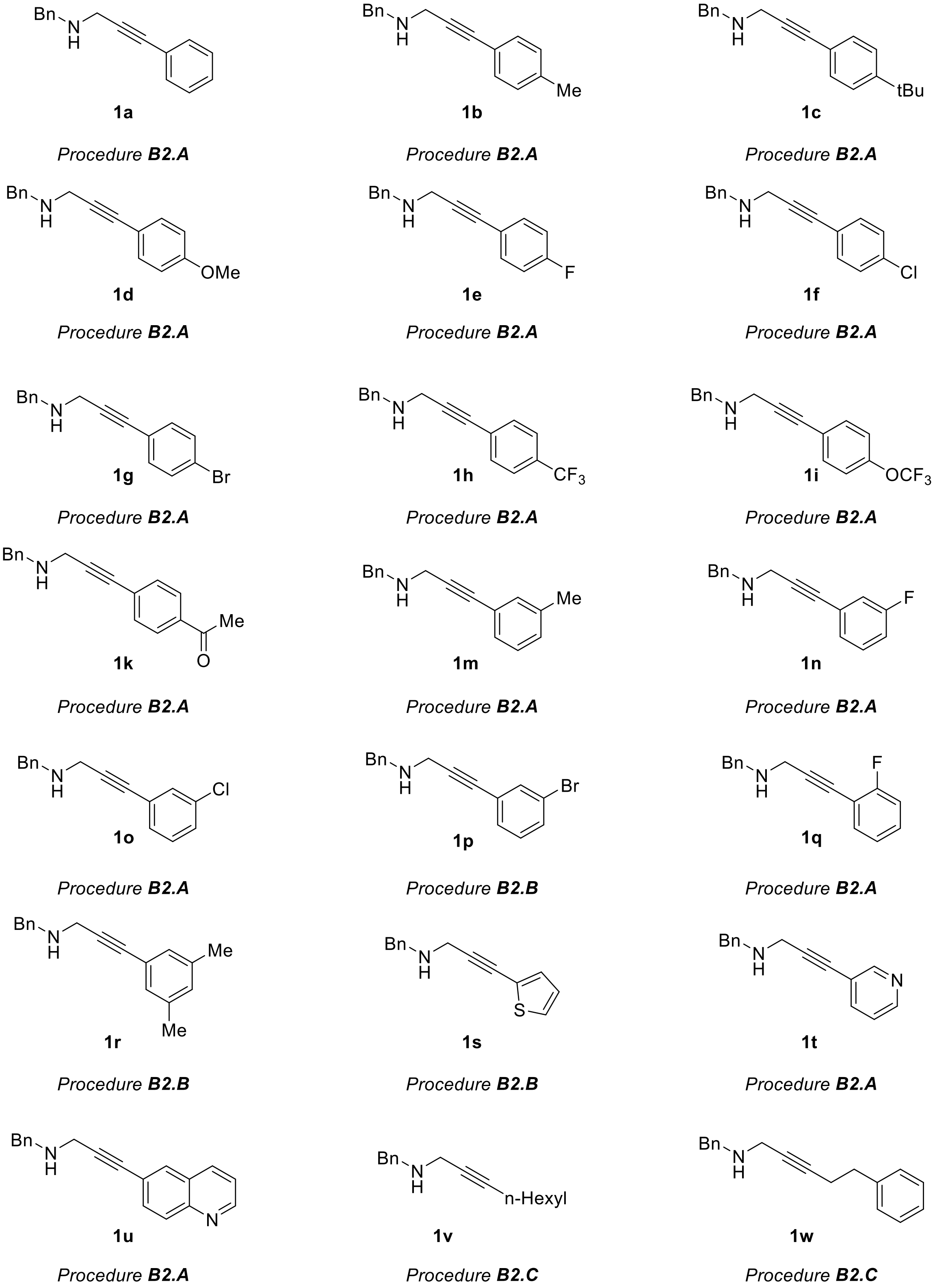

Scheme 3. The propargylic amines synthesized according to the general procedures reported.

General Procedure B2.A

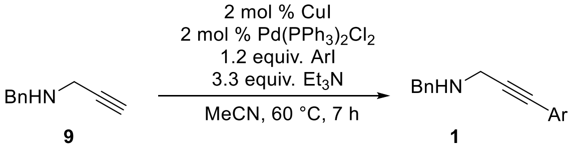

Scheme 4. General Procedure B2.A.

To a flame-dried 100 mL round bottom flask equipped with a Teflon-coated magnetic stirring bar, $Pd(PPh_3)_2Cl_2$ (42 mg, 60 µmol, 2 mol%), CuI (11 mg, 60 µmol, 2 mol%), Et_3N (0.90 g, 1.2 mL, 9.0 mmol, 3.3 equiv.) and degassed (by bubbling dry N_2 for 10 minutes) MeCN (30 mL) were added. Then, the iodoarene (1.1 equiv.) was added and the mixture was heated to 60 °C and stirred for 5 minutes. Benzyl propargyl amine **9** (0.39 g, 2.7 mmol, 1.0 equiv.) was added and the reaction mixture was stirred for 7 hours at 60 °C. Then, the reaction mixture was cooled down to ambient temperature and concentrated *in vacuo*. The resulting crude mixture was dissolved in EtOAc (20 mL), then washed with water (20 mL) and brine (20 mL). The organic layer was dried over Na_2SO_4, filtered, and concentrated *in vacuo*. The crude was purified with Biotage flash chromatography system using Buchi FlashPure cartridge with EcoFlex silica (10% – 40% EtOAc in pentane).

General Procedure B2.B

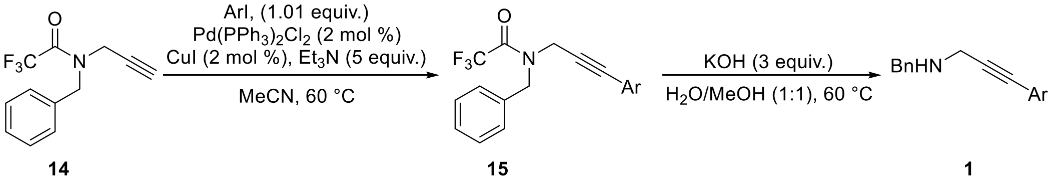

Scheme 5. General Procedure B2.B.

Following a modified version of a reported procedure.[7] To a solution of **14** (0.80 g, 3.3 mmol, 1 equiv.), ArI (1.01 equiv.) and Et_3N (2.3 mL, 17 mmol, 5 equiv.) in acetonitrile (30 mL) was added $PdCl_2(PPh_3)_2$ (47 mg, 0.066 mmol, 2 mol%) and CuI (13 mg, 0.066 mmol, 2 mol%) in a single portion. The resulting mixture was stirred for 7 hours at 60 °C. Water (20 mL) was then added and the reaction mixture extracted with EtOAc (3 x 30 mL); the combined organic layers were dried over $MgSO_4$, filtered, and concentrated by rotary evaporation. The crude material was purified by flash column chromatography (SiO_2, 0-5% EtOAc in pentane).

Hydrolysis: following an adapted version of a reported procedure.[8] To the trifluoroacetamide **15** obtained from the previous step (1 equiv.) was added a solution of KOH (3.0 equiv.) in water (15 mL) and methanol (15 mL) and the resulting mixture was stirred at 60 °C for 3 hours. The reaction was then cooled to room temperature and acidified with aq. HCl (1.0 M; 5 mL) followed by basification with sat. aq. $NaHCO_3$ (pH >7). The resulting mixture was extracted with DCM (3 x 10 mL), dried over $MgSO_4$, filtered, and concentrated by rotary evaporation. The crude material was purified by flash column chromatography (SiO_2, 10-30% EtOAc in pentane).

General Procedure B2.C

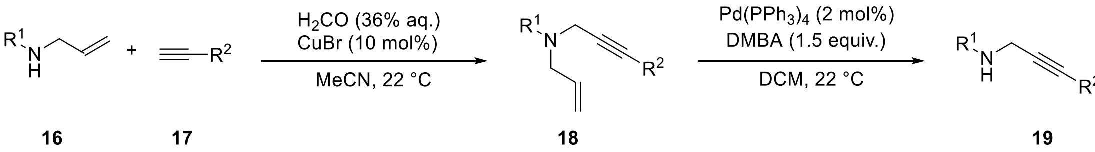

Scheme 6. General Procedure B2.C.

Following an adapted version of a reported procedure. [9] To a solution of CuBr (0.20 g, 1.4 mmol, 13 mol%) in MeCN (c = 0.15 M) was added allyl amine **16** (1.3 equiv.), formaldehyde (3 equiv.) and alkyne **17** (1 equiv.). The reaction mixture was stirred at room temperature for 16 hours after which it was concentrated by rotary evaporation. The residue was diluted with Et_2O (20 mL) and washed with aq. NaOH solution (5.0

M; 3 x 10 mL), dried over $MgSO_4$, filtered, and concentrated by rotary evaporation. The crude material was purified by flash column chromatography (SiO_2, 0-2% EtOAc in pentane).

Deallylation: The tertiary amine **18** obtained from the previous step (1 equiv.) was added to a solution of $Pd(PPh_3)_4$ (2 mol%) and 1,3-dimethylbarbituric acid (1.5 equiv.) in DCM (c = 0.18 M) under an N_2 atmosphere. The reaction mixture was stirred at room temperature for 16 hours. The reaction mixture was concentrated to a quarter of its original volume and diluted with ether (40 mL) and washed with sat. $NaHCO_3$ (3 x 15 mL). The organic layer was extracted with aq. HCl (1.0 M; 3 x 15 mL) after which the combined aqueous layers and any precipitated solids were basified with K_2CO_3 (pH >7) and extracted with DCM (3 x 25 mL). The combined extracts were dried over $MgSO_4$, filtered, and concentrated by rotary evaporation. The crude material was purified by flash column chromatography (SiO_2, 20-50% EtOAc in pentane) to obtain the compound **19**.

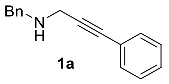

1a

N-Benzyl-3-phenylprop-2-yn-1-amine (1a)

Prepared following an up-scaled general procedure B2.A using *N*-benzylprop-2-yn-1-amine **12** (2.20 g, 13.5 mmol, 1.0 equiv.), iodobenzene (3.1 g, 1.7 mL, 15 mmol, 1.1 equiv.), Et_3N (4.5 g, 6.3 mL, 45 mmol, 3.3 equiv.), $Pd(PPh_3)_2Cl_2$ (211 mg, 300 µmol, 2 mol%) and CuI (57 mg, 300 µmol, 2 mol%). Purification was performed by Biotage flash column chromatography system with a 120 g cartridge (SiO_2, 10 – 40% EtOAc in pentane) to afford *N*-benzyl-3-phenylprop-2-yn-1-amine (**1a**) as an orange oil (2.5 g, 11 mmol, 75% yield).

R_f value: 0.36 (20% Ethyl acetate in Pentane).

^{1}H NMR (400 MHz, Chloroform-*d*) δ 7.52 – 7.20 (m, 9H, Ar*H*), 3.96 (s, 2H, PhCH_2), 3.66 (s, 2H, CH_2C≡C), 1.73 (br. s, 1H, N*H*).

^{13}C{^{1}H} NMR (101 MHz, Chloroform-*d*) δ 139.5, 131.7, 128.5 (2C), 128.3, 128.1, 127.2, 123.2, 87.5, 83.8, 52.5, 38.3.

Spectral data were consistent with the values reported in literature.[9]

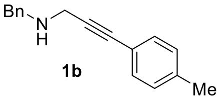

1b

N-Benzyl-3-(*p*-tolyl)prop-2-yn-1-amine (1b)

Prepared following general procedure B2.A using *p*-tolyliodobenzene (667 mg, 3.06 mmol, 1.1 equiv.). Purification was performed by Biotage flash column chromatography system with a 25 g cartridge (SiO_2, 10 – 40% EtOAc in pentane) to afford *N*-benzyl-3-(p-tolyl)prop-2-yn-1amine (**1b**) as an orange oil (512 mg, 2.13 mmol, 79% yield).

R_f value: 0.38 (20% Ethyl acetate in Pentane).

^{1}H NMR (400 MHz, Chloroform-d) δ 7.41 – 7.29 (m, 6H, Ar*H*), 7.29 – 7.22 (m, 1H, Ar*H*), 7.12 (d, *J* = 7.9 Hz, 2H, Ar*H*), 3.95 (s, 2H, PhCH_2), 3.65 (s, 2H, CH_2C-C≡C), 2.35 (s, 3H), 1.68 (br. s., 1H, N*H*)

^{13}C{^{1}H} NMR (101 MHz, $CDCl_3$) δ 139.7, 138.3, 131.7, 129.2, 128.62, 128.59, 127.3, 120.3, 86.7, 84.0, 52.6, 38.4, 21.6.

Spectral data were consistent with the values reported in literature.[9]

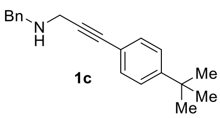

1c

N-benzyl-3-(4-(tert-butyl)phenyl)prop-2-yn-1-amine (1c)

Prepared following an scaled-up general procedure B2.A using *N*-benzylprop-2-yn-1-amine **12** (0.39 g, 2.7 mmol, 1.0 equiv.), 1-*tert*-butyl-4-iodobenzene (0.84 g, 0.57 mL, 3.2 mmol, 1.2 equiv.), Et_3N (0.90 g, 1.3 mL, 8.9 mmol, 3.3 equiv.), $Pd(PPh_3)_2Cl_2$ (38 mg, 54 µmol, 2 mol%) and CuI (11 mg, 54 µmol, 2 mol%). Purification was performed by Biotage flash column chromatography system with a 120 g cartridge (SiO_2, 10 – 40% EtOAc in pentane) to afford *N*-benzyl-3-(4-(tert-butyl)phenyl)prop-2-yn-1-amine **(1c)** as an orange oil (0.53 g, 1.9 mmol, 71% yield).

R_f value: 0.35 (20% Ethyl acetate in Pentane).

^{1}H NMR (400 MHz, Chloroform-*d*) δ 7.41 – 7.30 (m, 8H, Ar*H*), 7.30 – 7.24 (m, 1H, Ar*H*), 3.95 (s, 2H, PhCH_2), 3.65 (s, 2H, CH_2C≡C), 1.62 (br. s, 1H, N*H*), 1.32 (s, 9H, ArC(CH_3)$_3$).

^{13}C{^{1}H} NMR (101 MHz, Chloroform-*d*) δ 151.4, 139.8, 131.5, 128.6, 128.6, 127.3, 125.4, 120.4, 86.9, 83.9, 52.6, 38.4, 34.9, 31.3.

IR (cm^{-1}) 3032 (m), 2962 (s), 1658 (s), 1504 (s), 1458 (s), 1361 (m), 1269 (m), 1115 (m), 837 (m), 741 (s), 702 (s).

HRMS (ESI/QTOF) m/z: $[M + H]^+$ Calcd for $C_{20}H_{24}N^+$ 278.1903; Found 278.1901.

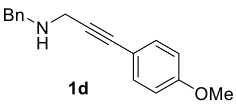

1d

N-Benzyl-3-(4-methoxyphenyl)prop-2-yn-1-amine (1d)

Prepared following modified general procedure B2.A using $Pd(PPh_3)_2Cl_2$ (90 mg, 0.13 mmol, 5 mol%), dppf (86 mg, 0.16 mmol, 6 mol%), CuI (25 mg, 0.13 mmol, 5 mol%), DABCO (0.76 g, 6.8 mmol, 2.6 equiv.) and 4-iodo-anisole (0.79 g, 6.4 mmol, 1.3 mmol) in DMSO (10 mL; degassed by bubbling N_2). The crude material was dry-loaded onto SiO_2 and purified by column chromatography (SiO_2, 15-30% EtOAc in pentane) affording *N*-benzyl-3-(4-methoxyphenyl)prop-2-yn-1-amine (**1d**) as a light orange solid (0.28 g, 1.1 mmol, 43% yield).

R_f value: 0.28 (20% Ethyl acetate in Pentane).

^{1}H NMR (400 MHz, Chloroform-*d*) δ 7.42 – 7.23 (m, 7H, Ar*H*), 6.87 – 6.81 (m, 2H, Ar*H*), 3.95 (s, 2H, ArC*H₂*), 3.81 (s, 3H, C*H₃*), 3.64 (s, 2H, C*H₂*C≡C), 1.64 (bs, 1H, N*H*).

^{13}C NMR (101 MHz, Chloroform-*d*) δ 159.4, 139.6, 133.0, 128.4 (2C), 127.1, 115.3, 113.9, 86.0, 83.5, 55.3, 52.5, 38.3.

Spectral data was consistent with the values reported in literature.[10]

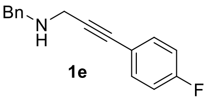

1e

N-Benzyl-3-(4-fluorophenyl)prop-2-yn-1-amine (1e)

Prepared following general procedure B2.A using 4-fluoroiodobenzene (0.68 g, 0.35 mL, 3.1 mmol, 1.1 equiv.). Purification was performed by Biotage flash column chromatography system with a 25 g cartridge (SiO_2, 10 – 40 % EtOAc in pentane) to afford *N*-benzyl-3-(4-fluorophenyl)prop-2-yn-1-amine (**1e**) as an orange oil (512 mg, 2.02 mmol, 79% yield).

R_f value: 0.39 (20% Ethyl acetate in Pentane).

^{1}H NMR (400 MHz, Chloroform-*d*) δ 7.48 – 7.30 (m, 6H, Ar*H*), 7.30 – 7.22 (m, 1H, Ar*H*), 7.07 – 6.91 (m, 2H, *o*-FAr*H*), 3.95 (s, 2H, PhC*H₂*), 3.64 (s, 2H, C*H₂*C≡C), 1.61 (br. s., 1H, N*H*).

^{13}C{^{1}H} NMR (101 MHz, Chloroform-*d*) δ 162.5 (d, J = 249.0 Hz), 139.7, 133.6 (d, J = 8.3 Hz), 128.61, 128.55, 127.3, 119.4 (d, J = 3.5 Hz), 115.7 (d, J = 22.0 Hz), 87.4, 82.8, 52.7, 38.3.
^{19}F NMR (376 MHz, Chloroform-*d*) δ -111.4 (tt, J = 8.7, 5.4 Hz).

Spectral data were consistent with the values reported in literature.[9]

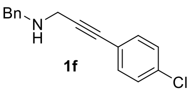

1f

N-Benzyl-3-(4-chlorophenyl)prop-2-yn-1-amine (1f)

Prepared following general procedure B2.A using 4-chloroiodobenzene (730 mg, 3.06 mmol, 1.1 equiv.). Purification was performed by Biotage flash column chromatography system with a 25 g cartridge (SiO_2, 10 – 40% EtOAc in pentane) to afford *N*-benzyl-3-(4-chlorophenyl)prop-2yn-1-amine (**1f**) as an orange oil (540 mg, 2.08 mmol, 77% yield).

R_f value: 0.36 (20% Ethyl acetate in Pentane).

^{1}H NMR (400 MHz, Chloroform-*d*) δ 7.41 – 7.29 (m, 6H, Ar*H*), 7.29 – 7.22 (m, 1H, Ar*H*), 7.12 (d, J = 7.9 Hz, 2H, *o*-MeAr*H*), 3.95 (s, 2H, PhC*H₂*), 3.65 (s, 2H, C*H₂*C≡C), 2.35 (s, 3H, C*H₃*), 1.57 (br. s., 1H, N*H*).

^{13}C{^{1}H} NMR (101 MHz, Chloroform-*d*) δ 139.6, 134.2, 133.0, 129.1, 128.6, 128.6, 127.3, 121.9, 87.7, 82.8, 52.7, 38.3.

IR (cm^{-1}) 3327 (w), 3031 (m), 2921 (m), 2840 (m), 2104 (w), 1727 (m), 1487 (s), 1335 (m), 1254 (m), 1166 (m), 1094 (s).

HRMS (ESI/QTOF) m/z: $[M + H]^+$ Calculated for $C_{16}H_{15}ClN^+$ 256.0888; Found 256.0890.

Spectral data were consistent with the values reported in literature.[13]

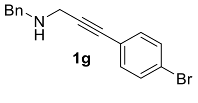

1g

N-benzyl-3-(4-bromophenyl)prop-2-yn-1-amine (1g)

Prepared following an scaled-up general procedure B2.A using *N*-benzylprop-2-yn-1-amine **12** (0.39 g, 2.7 mmol, 1.0 equiv.), 1-bromo-4-iodobenzene (0.92 g, 3.2 mmol, 1.2 equiv.), Et_3N (0.90 g, 1.3 mL, 8.9 mmol, 3.3 equiv.), $Pd(PPh_3)_2Cl_2$ (38 mg, 54 µmol, 2 mol%) and CuI (11 mg, 54 µmol, 2 mol%). Purification was performed by Biotage flash column chromatography system with a 120 g cartridge (SiO_2, 10 – 40% EtOAc in pentane) to afford *N*-benzyl-3-(4-bromophenyl)prop-2-yn-1-amine (**1g**) as an orange oil (0.60 g, 1.9 mmol, 73% yield).

R_f value: 0.38 (20% Ethyl acetate in Pentane).

^{1}H NMR (400 MHz, Chloroform-*d*) δ 7.47 – 7.41 (m, 2H, Ar*H*), 7.39 – 7.31 (m, 4H, Ar*H*), 7.31 – 7.26 (m, 3H, Ar*H*), 3.94 (s, 2H, PhC*H₂*), 3.64 (s, 2H, C*H₂*C≡C), 1.58 (br. s, 1H, N*H*).

$^{13}C\{^1H\}$ NMR (101 MHz, Chloroform-*d*) δ 139.6, 133.3, 131.7, 128.6, 128.5, 127.3, 122.4, 122.3, 88.9, 82.8, 52.7, 38.4.

IR (cm^{-1}) 3032 (w), 2920 (w), 2835 (w), 1485 (s), 1331 (m), 1111 (m), 1072 (m), 1011 (m), 910 (w), 825 (s), 741 (s).

HRMS (ESI/QTOF) m/z: $[M + H]^+$ Calcd for $C_{16}H_{15}{}^{79}BrN^+$ 300.0382; Found 300.0381.

Spectral data were consistent with the values reported in literature.[9]

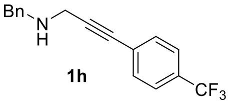

1h

N-Benzyl-3-(4-(trifluoromethyl)phenyl)prop-2-yn-1-amine (1h)

Prepared following modified general procedure B2.A using $Pd(PPh_3)_2Cl_2$ (90 mg, 0.13 mmol, 5 mol%), dppf (86 mg, 0.16 mmol, 6 mol%), CuI (25 mg, 0.13 mmol, 5 mol%), DABCO (0.76 g, 6.8 mmol, 2.6 equiv.) and 4-trifluro-Iodobenzene (0.92 g, 3.4 mmol, 1.3 equiv.) in DMSO (10 mL; degassed by bubbling N_2). The crude material was dry-loaded onto SiO_2 and purified by column chromatography (SiO_2, 10-20% EtOAc in pentane) affording *N*-benzyl-3-(4-(trifluoromethyl)phenyl)prop-2-yn-1-amine (**1h**) as a dark orange oil (0.55 g, 1.9 mmol, 72% yield).

R_f value: 0.34 (20% Ethyl acetate in Pentane).

^1H NMR (400 MHz, Chloroform-*d*) δ 7.61 – 7.24 (m, 9H, Ar*H*), 3.95 (s, 2H, ArC*H*$_2$), 3.67 (s, 2H, C*H*$_2$C≡C), 1.76 (bs, 1H, N*H*).

$^{13}C\{^1H\}$ NMR (101 MHz, Chloroform-*d*) δ 139.3, 131.9, 129.8 (q, *J* = 32.7 Hz), 128.5, 128.4, 127.2, 127.0, 125.2 (q, *J* = 3.9 Hz), 123.91 (q, *J* = 272.2 Hz), 90.2, 82.5, 52.6, 38.2.

^{19}F NMR (376 MHz, Chloroform-*d*) δ -63.2.

Spectral data was consistent with the values reported in literature.[10]

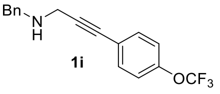

1i

N-benzyl-3-(4-(trifluoromethoxy)phenyl)prop-2-yn-1-amine (1i)

Prepared following an up-scaled general procedure B2.A using *N*-benzylprop-2-yn-1-amine **12** (0.58 g, 4.0 mmol, 1.0 equiv.), 1-iodo-4-(trifluoromethoxy)benzene (1.38 g, 0.751 mL, 4.80 mmol, 1.20 equiv.), Et_3N (1.34 g, 1.84 mL, 13.2 mmol, 3.30 equiv.), $Pd(PPh_3)_2Cl_2$ (56 mg, 80 µmol, 2.0 mol%) and CuI (15 mg, 80 µmol, 2.0 mol%). Purification was performed by flash column chromatography system (SiO_2, 10 – 40% EtOAc in pentane) to afford *N*-benzyl-3-(4-(trifluoromethoxy)phenyl)prop-2-yn-1-amine (**1i**) as an orange oil (1.0 g, 3.4 mmol, 86% yield).

R_f value: 0.39 (20% Ethyl acetate in Pentane).

^1H NMR (400 MHz, Chloroform-*d*) δ 7.50 – 7.41 (m, 2H, Ar*H*), 7.40 – 7.31 (m, 4H, Ar*H*), 7.31 – 7.25 (m, 1H, Ar*H*), 7.16 (dp, *J* = 7.8, 1.1 Hz, 2H, Ar*H*), 3.95 (s, 2H, PhC*H*$_2$), 3.65 (s, 2H, C*H*$_2$C≡C), 1.57 (br. s, 1H, N*H*).

$^{13}C\{^1H\}$ NMR (101 MHz, Chloroform-*d*) δ 148.9, 139.6, 133.3, 128.6, 128.6, 127.4, 122.2, 121.0, 120.5 (q, *J* = 257.6 Hz), 88.7, 82.5, 52.7, 38.3.

^{19}F NMR (376 MHz, Chloroform-*d*) δ -57.8 (s, 3F, ArOC*F*$_3$).

IR (cm^{-1}) 3035 (w), 2916 (w), 2835 (w), 1504 (m), 1454 (w), 1257 (s), 1215 (s), 1169 (s), 849 (w), 741 (m).

HRMS (nanochip-ESI/LTQ-Orbitrap) m/z: $[M + H]^+$ Calcd for $C_{17}H_{15}F_3NO^+$ 306.1100; Found 306.1092.

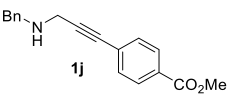

1j

Methyl 4-(3-(benzylamino)prop-1-yn-1-yl)benzoate (1j)

Prepared following an up-scaled general procedure B2.A using *N*-benzylprop-2-yn-1-amine **12** (0.39 g, 2.7 mmol, 1.0 equiv.), methyl 4-iodobenzoate (0.849 g, 3.24 mmol, 1.20 equiv.), Et_3N (0.902 g, 1.24 mL, 8.91 mmol, 3.30 equiv.), $Pd(PPh_3)_2Cl_2$ (38 mg, 54 µmol, 2.0 mol%) and CuI (11 mg, 54 µmol, 2.0 mol%). Purification was performed by flash column chromatography system (SiO_2, 10 – 40% EtOAc in pentane) to afford Methyl 4-(3-(benzylamino)prop-1-yn-1-yl)benzoate (**1j**) as an orange solid (0.58 g, 2.1 mmol, 76% yield).

R_f value: 0.39 (30% Ethyl acetate in Pentane).

Melting point: 45°C.

^1H NMR (400 MHz, Chloroform-*d*) δ 8.02 – 7.94 (m, 2H, Ar*H*), 7.53 – 7.45 (m, 2H, Ar*H*), 7.42 – 7.30 (m, 4H, Ar*H*), 7.30 (s, 1H, Ar*H*), 3.93 (s, 2H, PhC*H*$_2$), 3.92 (s, 3H, CO_2C*H*$_3$), 3.67 (s, 2H, C*H*$_2$C≡C), 1.64 (s, 1H, N*H*).

$^{13}C\{^1H\}$ NMR (101 MHz, Chloroform-*d*) δ 166.7, 139.5, 131.7, 129.6, 129.5, 128.6, 128.5, 128.1, 127.3, 91.0, 83.2, 52.7, 52.3, 38.4.

IR (cm^{-1}) 3029 (w), 2951 (w), 2841 (w), 1719 (s), 1606 (m), 1454 (m), 1435 (m), 1274 (s), 1176 (m), 1107 (s), 1019 (w), 859 (m), 769 (s), 741 (m), 697 (s).

HRMS (ESI/QTOF) m/z: $[M + H]^+$ Calcd for $C_{18}H_{18}NO_2^+$ 280.1332; Found 280.1332.

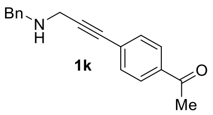

1-(4-(3-(Benzylamino)prop-1-yn-1-yl)phenyl)ethan-1-one (1k)

Prepared following an up-scaled general procedure B2.A using *N*-benzylprop-2-yn-1-amine **12** (0.58 g, 4.0 mmol, 1.0 equiv.), 1-(4-iodophenyl)ethanone (1.2 g, 4.8 mmol, 1.2 equiv.), Et_3N (1.3 g, 1.8 mL, 13 mmol, 3.3 equiv.), $Pd(PPh_3)_2Cl_2$ (56 mg, 80 µmol, 2 mol%) and CuI (15 mg, 80 µmol, 2 mol%). Purification was performed by flash column chromatography system (SiO_2, 10 – 40% EtOAc in pentane) to afford 1-(4-(3-(benzylamino)prop-1-yn-1-yl)phenyl)ethan-1-one (**1k**) as an orange oil (0.80 g, 3.03 mmol, 76% yield).

R_f value: 0.32 (20% Ethyl acetate in Pentane).

^{1}H NMR (400 MHz, Chloroform-*d*) δ 7.94 – 7.87 (m, 2H, Ar*H*), 7.55 – 7.47 (m, 2H, Ar*H*), 7.42 – 7.31 (m, 4H, Ar*H*), 7.30 – 7.23 (m, 1H, Ar*H*), 3.96 (s, 2H, PhC*H*$_2$), 3.68 (s, 2H, C*H*$_2$C≡C), 2.60 (s, 3H, COC*H*$_3$), 1.60 (br. s, 1H, N*H*).

^{13}C{^{1}H} NMR (101 MHz, Chloroform-*d*) δ 197.5, 139.5, 136.3, 131.9, 128.6, 128.6, 128.4, 128.3, 127.4, 91.3, 83.2, 52.7, 38.4, 26.8.

IR (cm^{-1}) 3336 (w), 3035 (w), 2920 (w), 2835 (w), 1682 (s), 1604 (m), 1358 (m), 1265 (s), 841 (m), 741 (m), 702 (m).

HRMS (nanochip-ESI/LTQ-Orbitrap) m/z: $[M + H]^+$ Calcd for $C_{18}H_{18}NO^+$ 264.1383; Found 264.1377.

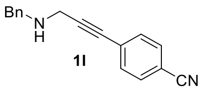

4-(3-(Benzylamino)prop-1-yn-1-yl)benzonitrile (1l)

Prepared following an up-scaled general procedure B2.A using *N*-benzylprop-2-yn-1-amine **12** (0.39 g, 2.7 mmol, 1.0 equiv.), 4-iodobenzonitrile (0.74 g, 3.2 mmol, 1.2 equiv.), Et_3N (0.90 g, 1.2 mL, 8.9 mmol, 3.3 equiv.), $Pd(PPh_3)_2Cl_2$ (38 mg, 54 µmol, 2.0 mol%) and CuI (11 mg, 54 µmol, 2.0 mol%). Purification was performed by Biotage flash column chromatography system with a 120 g cartridge (SiO_2, 10 – 40% EtOAc in pentane) to afford 4-(3-(benzylamino)prop-1-yn-1-yl)benzonitrile (**1m**) as an orange solid (0.48 g, 1.9 mmol, 72% yield).

R_f value: 0.32 (20% Ethyl acetate in Pentane).

Melting point: 48°C.

^{1}H NMR (400 MHz, Chloroform-*d*) δ 7.64 – 7.55 (m, 2H, Ar*H*), 7.55 – 7.46 (m, 2H, Ar*H*), 7.40 – 7.31 (m, 4H, Ar*H*), 7.31 – 7.24 (m, 1H, Ar*H*), 3.94 (s, 2H, PhC*H*$_2$), 3.68 (s, 2H, C*H*$_2$C≡C), 1.63 (s, 1H, N*H*).

^{13}C{^{1}H} NMR (101 MHz, Chloroform-*d*) δ 139.4, 132.3, 132.1, 128.6, 128.5, 128.3, 127.4, 118.6, 111.5, 92.6, 82.4, 52.7, 38.3.

IR (cm^{-1}) 3324 (w), 3028 (m), 2909 (w), 2835 (m), 2227 (s), 1604 (s), 1499 (s), 1454 (m), 1328 (m), 1273 (m), 1177 (m), 1105 (m), 839 (s), 737 (s), 700 (s).

HRMS (ESI/QTOF) m/z: $[M + H]^+$ Calcd for $C_{17}H_{15}N_2^+$ 247.1230; Found 247.1234.

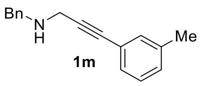

***N*-Benzyl-3-(m-tolyl)prop-2-yn-1-amine (1m)**

Prepared following an up-scaled general procedure B2.A using *N*-benzylprop-2-yn-1-amine **12** (0.39 g, 2.7 mmol, 1.0 equiv.), 1-iodo-3-methylbenzene (0.71 g, 0.42 mL, 3.2 mmol, 1.2 equiv.), Et_3N (0.90 g, 1.3 mL, 8.9 mmol, 3.3 equiv.), $Pd(PPh_3)_2Cl_2$ (38 mg, 54 µmol, 2 mol%) and CuI (11 mg, 54 µmol, 2 mol%). Purification was performed by Biotage flash column chromatography system with a 120 g cartridge (SiO_2, 10 – 40% EtOAc in pentane) to afford *N*-benzyl-3-(m-tolyl)prop-2-yn-1-amine (**1m**) as an orange oil (0.45 g, 1.9 mmol, 71% yield).

R_f value: 0.42 (20% Ethyl acetate in Pentane).

^{1}H NMR (400 MHz, Chloroform-*d*) δ 7.41 – 7.30 (m, 4H, Ar*H*), 7.30 – 7.23 (m, 3H, Ar*H*), 7.20 (td, *J* = 7.5, 0.7 Hz, 1H, Ar*H*), 7.12 (dtd, *J* = 7.4, 1.5, 0.8 Hz, 1H, Ar*H*), 3.95 (s, 2H, PhC*H*$_2$), 3.65 (s, 2H, C*H*$_2$C≡C), 2.33 (d, *J* = 0.8 Hz, 3H, ArC*H*$_3$), 1.58 (br. s, 1H, N*H*).

^{13}C{^{1}H} NMR (101 MHz, Chloroform-*d*) δ 139.7, 138.1, 132.4, 129.1, 128.9, 128.6 (2C), 128.3, 127.3, 123.2, 87.3, 84.0, 52.6, 38.4, 21.4.

IR (cm^{-1}) 3032 (m), 2920 (m), 2850 (m), 1601 (m), 1485 (m), 1454 (m), 1331 (m), 1254 (m), 1107 (m), 910 (m), 787 (s), 737 (s), 698 (s).

HRMS (ESI/QTOF) m/z: $[M + H]^+$ Calcd for $C_{17}H_{18}N^+$ 236.1434; Found 236.1436.

N-benzyl-3-(3-fluorophenyl)prop-2-yn-1-amine (1n)

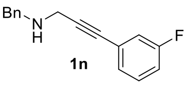

Prepared following an up-scaled general procedure B2.A using *N*-benzylprop-2-yn-1-amine **12** (0.78 g, 5.4 mmol, 1.0 equiv.), 1-fluoro-3-iodobenzene (1.4 g, 0.76 mL, 6.4 mmol, 1.2 equiv.), Et_3N (1.8 g, 2.6 mL, 18 mmol, 3.3 equiv.), $Pd(PPh_3)_2Cl_2$ (0.076 g, 108 µmol, 2.00 mol%) and CuI (0.022 g, 108 µmol, 2.00 mol%). Purification was performed by Biotage flash column chromatography system with a 120 g cartridge (SiO_2, 10 – 40% EtOAc in pentane) to afford *N*-benzyl-3-(3-fluorophenyl)prop-2-yn-1-amine (**1n**) as an orange oil (0.87 g, 3.6 mmol, 67% yield).

R_f value: 0.43 (20% Ethyl acetate in Pentane).

^{1}H NMR (400 MHz, Chloroform-*d*) δ 7.41 – 7.31 (m, 4H, Ar*H*), 7.30 – 7.19 (m, 3H, Ar*H*), 7.13 (ddd, *J* = 9.5, 2.6, 1.4 Hz, 1H, Ar*H*), 7.02 (tdd, *J* = 8.2, 2.7, 1.2 Hz, 1H, Ar*H*), 3.95 (s, 2H, PhCH_2), 3.65 (s, 2H, CH_2C≡C), 1.59 (br. s, 1H, N*H*).

^{13}C{^{1}H} NMR (101 MHz, Chloroform-*d*) δ 162.5 (d, *J* = 246.4 Hz), 139.6, 130.0 (d, *J* = 8.7 Hz), 128.6, 128.6, 127.7 (d, *J* = 3.2 Hz), 127.3, 125.2 (d, *J* = 9.4 Hz), 118.6 (d, *J* = 22.6 Hz), 115.6 (d, *J* = 21.1 Hz), 88.8, 82.7 (d, *J* = 3.4 Hz), 52.7, 38.3.

^{19}F NMR (376 MHz, Chloroform-*d*) δ -113.1 (s, 1F, Ar*F*).

IR (cm^{-1}) 3066 (m), 3032 (m), 2920 (m), 2843 (m), 1577 (s), 1481 (s), 1446 (m), 1331 (m), 1277 (m), 1157 (s), 1107 (m), 991 (m), 872 (m), 787 (s), 737 (s), 690 (s).

HRMS (ESI/QTOF) m/z: $[M + H]^+$ Calcd for $C_{16}H_{15}FN^+$ 240.1183; Found 240.1181.

N-Benzyl-3-(3-chlorophenyl)prop-2-yn-1-amine (1o)

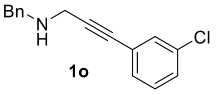

Prepared following general procedure B2.A using 3-chloroiodobenzene (730 mg, 3.06 mmol, 1.1 equiv.). Purification was performed by Biotage flash column chromatography system with a 25 g cartridge (SiO_2, 10 – 40% EtOAc in pentane) to afford *N*-benzyl-3-(3-chlorophenyl)prop-2yn-1-amine (**1o**) as an orange oil (530 mg, 2.08 mmol, 77% yield).

R_f value: 0.36 (20% Ethyl acetate in Pentane).

^{1}H NMR (400 MHz, Chloroform-*d*) δ 7.45 – 7.40 (m, 1H, Ar*H*), 7.40 – 7.18 (m, 8H, Ar*H*), 3.94 (s, 2H, PhCH_2), 3.65 (s, 2H, CH_2C≡C), 1.60 (br. s., 1H. N*H*).

^{13}C{^{1}H} NMR (101 MHz, Chloroform-*d*) δ 139.6, 134.2, 131.7, 129.9, 129.6, 128.62, 128.56, 128.5, 127.4, 125.1, 89.1, 82.5, 52.7, 38.3.

IR (cm^{-1}) 3324 (m), 3030 (m), 2909 (m), 2833 (m), 2357 (w), 1589 (m), 1560 (m), 1465 (m).

HRMS (ESI/QTOF) m/z: $[M + H]^+$ Calculated for $C_{16}H_{15}ClN^+$ 256.0888; Found 256.0886.

Spectral data were consistent with the values reported in literature.[13]

N-benzyl-3-(3-bromophenyl)prop-2-yn-1-amine (1p)

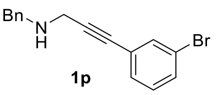

Prepared following general procedure B2.B using $PdCl_2(PPh_3)_2$ (47 mg, 66 µmol, 2 mol%), CuI (13 mg, 66 µmol, 2 mol%, **12** (0.80 g, 3.3 mmol, 1 equiv.), 1-bromo-3-iodobenzene (0.95 g, 3.4 mmol, 1.01 equiv.) and Et_3N (2.3 mL, 17 mmol, 5 equiv.) in acetonitrile (30 mL). The crude material was purified by flash column chromatography (SiO_2, 0-5% EtOAc in pentane) affording *N*-benzyl-*N*-(3-(3-bromophenyl)prop-2-yn-1-yl)-2,2,2-trifluoroacetamide as an yellow oil (1.2 g, 3.0 mmol, 92% yield).

Hydrolysis: the obtained trifluoroacetamide (1.2 g, 3.0 mmol, 1 equiv.) was treated with KOH (0.50 g, 9.0 mmol, 3.0 equiv.) in H_2O (15 mL) and MeOH (15 mL). Purification by column chromatography (SiO_2, 10-30% EtOAc in pentane) afforded *N*-benzyl-3-(3-bromophenyl)prop-2-yn-1-amine (**1p**) as a light yellow oil (0.80 g, 2.7 mmol, 88% yield).

R_f value: 0.36 (20% Ethyl acetate in Pentane).

^{1}H NMR (400 MHz, Chloroform-*d*) δ 7.59 (t, *J* = 1.7 Hz, 1H, Ar*H*), 7.45 (ddd, *J* = 8.0, 2.1, 1.1 Hz, 1H, Ar*H*), 7.43 – 7.24 (m, 6H, Ar*H*), 7.18 (t, *J* = 7.9 Hz, 1H, Ar*H*), 3.96 (s, 2H, ArCH_2), 3.66 (s, 2H, CH_2C≡C), 2.37 (s, 1H, N*H*).

^{13}C{^{1}H} NMR (101 MHz, Chloroform-*d*) δ 138.7, 134.4, 131.3, 130.2, 129.7, 128.5, 128.5, 127.4, 125.1, 122.1, 88.4, 82.6, 52.3, 37.9.

HRMS (ESI/QTOF) m/z: $[M + H]^+$ Calculated for $C_{16}H_{15}{}^{79}BrN^+$300.0382; Found 300.0384.

Spectral data were consistent with the values reported in literature.[13]

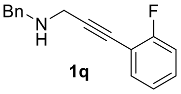

1q

N-Benzyl-3-(2-fluorophenyl)prop-2-yn-1-amine (1q)

Prepared following general procedure B2.A using 2-fluoroiodobenzene (0.80 g, 0.42 mL, 3.6 mmol, 1.2 equiv.). Purification was performed by Biotage flash column chromatography system with a 25 g cartridge (SiO_2, 10 – 40% EtOAc in pentane) to afford *N*-benzyl-3-(2-fluorophenyl)prop-2-yn-1-amine (**1q**) as an orange oil (520 mg, 2.17 mmol, 72% yield).

R_f value: 0.40 (20% Ethyl acetate in Pentane).

^{1}H NMR (400 MHz, Chloroform-*d*) δ 7.34 – 7.19 (m, 5H, Ar*H*), 7.19 – 7.10 (m, 2H, Ar*H*), 7.00 – 6.92 (m, 2H, Ar*H*), 3.86 (s, 2H, PhC*H₂*), 3.58 (s, 2H, C*H₂*C≡C), 1.48 (s, 1H, N*H*).

^{13}C{^{1}H} NMR (101 MHz, Chloroform-*d*) δ 163.0 (d, *J* = 250.9 Hz), 139.6, 133.7, 129.9 (d, *J* = 7.9 Hz), 128.7, 128.6, 127.3, 124.0 (d, *J* = 3.7 Hz), 115.6 (d, *J* = 21.0 Hz), 111.9 (d, *J* = 15.7 Hz), 93.2, 77.3, 52.5, 38.4.

^{19}F NMR (376 MHz, Chloroform-*d*) δ -110.4 (d, 1F, *J* = 5.9 Hz, Ar*F*).

IR (cm^{-1}) 3324 (m), 3032 (m), 2912 (m), 2836 (m), 2104 (w), 1494 (s), 1451 (s), 1327 (m), 1214 (m), 1107 (m).

HRMS (ESI/QTOF) m/z: $[M + H]^+$ Calculated for $C_{16}H_{15}FN^+$ 240.1183; Found 240.1184.

Spectral data were consistent with the values reported in literature.[13]

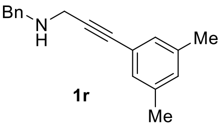

1r

N-benzyl-3-(3,5-dimethylphenyl)prop-2-yn-1-amine (1r)

Prepared following modified general procedure B2.B using $PdCl_2(PPh_3)_2$ (0.14 g, 0.20 mmol, 5 mol%), PPh_3 (0.21 g, 0.80 mmol, 20 mol%) and CuI (76 mg, 0.40 mmol, 10 mol%). **12** (0.97 g, 4.0 mmol, 1 equiv.), 1-iodo-3,5-dimethylbenzene (1.1 g, 4.8 mmol, 1.2 equiv.) in DMF (3.3 mL) and Et_3N (10 mL). The crude material was purified by flash column chromatography (SiO_2, 0-5% EtOAc in pentane) afforded *N*-benzyl-*N*-(3-(3,5-dimethylphenyl)prop-2-ynyl)-trifluoroacetamide as an orange oil (1.2 g, 3.6 mmol, 90% yield).

Hydrolysis: the obtained trifluoroacetamide (0.84 g, 2.4 mmol, 1 equiv.) was treated with KOH (0.15 g, 2.7 mmol, 1.3 equiv.) in H_2O (5 mL) and MeOH (5 mL). Purification by column chromatography (SiO_2, 10-40% EtOAc in pentane) afforded *N*-benzyl-3-(3,5-dimethylphenyl)prop-2-ynylamine (**1r**) as an orange oil (0.49 g, 2.0 mmol, 76% yield).

R_f value: 0.41 (20% Ethyl acetate in Pentane).

^{1}H NMR (400 MHz, Chloroform-*d*) δ 7.42 – 7.24 (m, 5H, Ar*H*), 7.08 (m, 2H, Ar*H*), 6.95 (m, 1H, Ar*H*), 3.96 (s, 2H, ArC*H₂*), 3.65 (s, 2H, C*H₂*C≡C), 2.29 (s, 6H, C*H₃*), 2.09 (bs, 1H, N*H*).

^{13}C{^{1}H} NMR (101 MHz, Chloroform-*d*) δ 139.3, 137.8, 130.0, 129.3, 128.5, 128.4, 127.2, 122.8, 86.5, 84.2, 52.3, 38.1, 21.1.

Spectral data was consistent with the values reported in literature.[11]

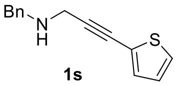

1s

N-benzyl-3-(thiophen-2-yl)prop-2-yn-1-amine (1s)

Prepared following general procedure B2.B using $PdCl_2(PPh_3)_2$ (36 mg, 51 µmol, 2 mol%), CuI (12 mg, 66 µmol, 3 mol%), **12** (0.50 g, 2.0 mmol, 1 equiv.), 2-iodothiophene (0.43 g, 2.0 mmol, 1.01 equiv.) and Et_3N (1.4 mL, 10 mmol, 5 equiv.) in acetonitrile (30 mL). The The crude material was purified by flash column chromatography (SiO_2, 0-5% EtOAc in pentane) afforded *N*-benzyl-2,2,2-trifluoro-*N*-(3-(thiophen-2-yl)prop-2-yn-1-yl)acetamide as an yellow oil (0.58 g, 1.8 mmol, 88% yield).

Hydrolysis: the obtained trifluoroacetamide (0.58 g, 1.8 mmol, 1 equiv.) was treated with KOH (0.30 g, 5.4 mmol, 3.0 equiv.) in H_2O (9 mL) and MeOH (9 mL). Purification by column chromatography (SiO_2, 10-30% EtOAc in pentane) afforded *N*-benzyl-3-(thiophen-2-yl)prop-2-yn-1-amine (**1s**) as an orange amorphous solid (0.38 g, 1.7 mmol, 93% yield).

R_f value: 0.36 (20% Ethyl acetate in Pentane).

^{1}H NMR (400 MHz, Chloroform-*d*) δ 7.40 – 7.27 (m, 5H, Ar*H*), 7.24 (dd, *J* = 5.2, 1.2 Hz, 1H, Ar*H*), 7.20 (dd, *J* = 3.6, 1.1 Hz, 1H, Ar*H*), 6.97 (dd, *J* = 5.2, 3.6 Hz, 1H, Ar*H*), 3.95 (s, 2H, ArC*H₂*), 3.68 (s, 2H, C*H₂*C≡C), 3.00 (s, 1H N*H*).

$^{13}C\{^{1}H\}$ NMR (101 MHz, Chloroform-*d*) δ 138.8, 131.8, 128.5, 128.5, 127.3, 126.9, 126.8, 123.1, 91.0, 77.3, 52.3, 38.2.

HRMS (ESI/QTOF) m/z: $[M + H]^{+}$ Calculated for $C_{14}H_{14}NS^{+}$ 228.0841; Found 228.0844.

Spectral data were consistent with the values reported in literature.[13]

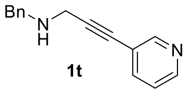

1t

***N*-Benzyl-3-(pyridin-3-yl)prop-2-yn-1-amine (1t)**

Prepared following general procedure B2.A using 3-bromopyridine (0.48 g, 0.30 mL, 3.06 mmol, 1.1 equiv.). Purification was performed by two sequential runs of Biotage flash column chromatography system with a 25 g cartridge (SiO_2, 0 – 10% MeOH in DCM) to afford *N*-benzyl-3-(pyridin-3-yl)prop-2-yn-1-amine (**1t**) as a dark orange oil (401 mg, 1.80 mmol, 60% yield). The material was used without further purification.

R_f value: 0.35 (DCM/EA/MeOH 6:4:0.3).

^{1}H NMR (400 MHz, DMSO-*d6*) δ 8.62 (br. s, 1H, HetAr*H*), 8.55 (br. s, 1H, HetAr*H*), 7.84 (dt, *J* = 7.9, 1.9 Hz, 1H, HetAr*H*), 7.45 – 7.29 (m, 5H, HetAr*H* and Ar*H*), 7.26 – 7.19 (m, 1H, Ar*H*), 3.82 (s, 2H, PhCH_2), 3.56 (s, 2H, CH_2C≡C).

$^{13}C\{^{1}H\}$ NMR (101 MHz, DMSO-*d6*) δ 151.6, 148.6, 140.1, 138.5, 128.1, 128.1, 126.7, 123.6, 119.8, 92.3, 79.8, 51.5, 37.4.

IR (cm^{-1}) 3649 (m), 3276 (m), 3032 (m), 2914 (m), 2831 (m), 2233 (w), 1663 (m), 1465 (m), 1112 (m).

HRMS (ESI/QTOF) m/z: $[M + H]^{+}$ Calculated for $C_{15}H_{15}N_2^{+}$ 223.1230; Found 223.1232.

Spectral data were consistent with the values reported in literature.[13]

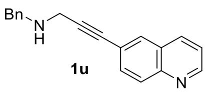

1u

***N*-benzyl-3-(quinolin-6-yl)prop-2-yn-1-amine (1u)**

Prepared following an up-scaled general procedure B2.A using *N*-benzylprop-2-yn-1-amine **12** (0.39 g, 2.7 mmol, 1.0 equiv.), 6-iodoquinoline (0.83 g, 3.2 mmol, 1.2 equiv.), Et_3N (0.90 g, 1.3 mL, 8.9 mmol, 3.3 equiv.), $Pd(PPh_3)_2Cl_2$ (38 mg, 54 µmol, 2 mol%) and CuI (11 mg, 54 µmol, 2 mol%). Purification was performed by Biotage flash column chromatography system with a 120 g cartridge (SiO_2, 10 – 40% EtOAc in pentane) to afford *N*-benzyl-3-(quinolin-6-yl)prop-2-yn-1-amine (**1u**) as an orange oil (0.64 g, 2.3 mmol, 87% yield).

R_f value: 0.26 (DCM/EA/MeOH 6:4:0.3).

^{1}H NMR (400 MHz, Chloroform-*d*) δ 8.91 (dd, *J* = 4.3, 1.7 Hz, 1H, Ar*H*), 8.10 (dt, *J* = 8.1, 1.4 Hz, 1H, Ar*H*), 8.04 (dd, *J* = 8.6, 0.8 Hz, 1H, Ar*H*), 7.92 (d, *J* = 1.8 Hz, 1H, Ar*H*), 7.72 (dd, *J* = 8.7, 1.8 Hz, 1H, Ar*H*), 7.45 – 7.32 (m, 5H, Ar*H*), 7.31 – 7.26 (m, 1H, Ar*H*), 3.99 (s, 2H, PhCH_2), 3.71 (s, 2H, CH_2C≡C), 1.64 (br. s, 1H, N*H*).

$^{13}C\{^{1}H\}$ NMR (101 MHz, Chloroform-*d*) δ 151.0, 147.8, 139.6, 135.8, 132.5, 131.2, 129.7, 128.6, 128.6, 128.1, 127.4, 121.8, 121.7, 89.1, 83.5, 52.8, 38.5.

IR (cm^{-1}) 3309 (w), 3032 (m), 2916 (w), 2835 (w), 1589 (w), 1496 (m), 1454 (m), 1331 (m), 1115 (m), 895 (m), 841 (s), 741 (s).

HRMS (ESI/QTOF) m/z: $[M + H]^{+}$ Calcd for $C_{19}H_{17}N_2^{+}$ 273.1386; Found 273.1390.

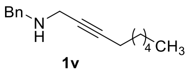

1v

***N*-benzylnon-2-yn-1-amine (1v)**

Prepared following general procedure B2.C using CuBr (54 mg, 0.37 mmol, 12 mol%), allyl benzylamine (0.55 g, 0.59 mL, 3.7 mmol, 1.3 equiv), formaldehyde (0.75 g, 0.70 mL, 9.0 mmol 36% aq. solution, 3.0 equiv) and 1-octyne (0.33 g, 0.44 mL, 3.0 mmol, 1.0 equiv.) in MeCN (25 mL). Purification of the crude product by column chromatography (SiO_2, 0-2% EtOAc in pentane) to afford *N*-allyl-N-benzylnon-2-yn-1-amine as a colourless oil (0.68 g, 2.5 mmol, 84% yield).

Deallylation: the obtained tertiary amine (0.84 g, 3.1 mmol, 1 equiv.) was treated with $Pd(PPh_3)_4$ (72 mg, 63 µmol, 2 mol%) and 1,3-dimethylbarbituric acid (0.73 g, 4.7 mmol, 1.5 equiv.) in DCM (20 mL). Purification by flash column chromatography (SiO_2, 40-60% EtOAc in pentane) to afford *N*-benzylnon-2-yn-1-amine (**1v**) as a straw-coloured oil (0.12 g, 0.5 mmol, 16% yield).

R_f value: 0.46 (40% Ethyl acetate in Pentane).

^{1}H NMR (400 MHz, Chloroform-*d*) δ 7.41 – 7.16 (m, 5H, Ar*H*), 3.86 (s, 2H, PhCH_2), 3.40 (t, *J* = 2.2 Hz, 2H, CH_2C≡C-$CH_2(CH_2)_4CH_3$), 2.21 (tt, *J* = 7.1, 2.2 Hz, 2H, CH_2C≡C-C$H_2(CH_2)_4CH_3$), 1.58 – 1.19 (m, 9H, CH_2C≡C-CH_2(C$H_2)_4CH_3$+ br. s, 1H, N*H*), 0.94 – 0.82 (m, 3H, CH_2C≡C-$CH_2(CH_2)_4$CH_3).

$^{13}C\{^1H\}$ NMR (101 MHz, Chloroform-*d*) δ 139.9, 128.6, 128.5, 127.2, 84.2, 78.1, 52.6, 38.0, 31.5, 29.0, 28.7, 22.7, 18.9, 14.2.

IR (cm^{-1}) 3066 (w), 3032 (w), 2927 (s), 2858 (m), 1581 (m), 1454 (m), 1331 (m), 1277 (m), 1161 (m), 787 (m), 741 (s), 698 (m).

HRMS (ESI/QTOF) m/z: $[M + H]^+$ Calcd for $C_{16}H_{24}N^+$ 230.1903; Found 230.1904.

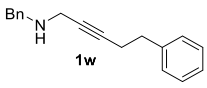

_N_-benzyl-5-phenylpent-2-yn-1-amine (1w)

Prepared following general procedure B2.C using CuBr (0.053 g, 0.37 mmol, 12 mol%), allyl benzylamine (0.55 g, 0.59 mL, 3.8 mmol, 1.3 equiv), formaldehyde (0.7 mL, 9 mmol 36% aq. solution, 3.0 equiv) and but-3-ynylbenzene (0.4 g, 0.4 mL, 3 mmol, 1 equiv.) in MeCN (25 mL). Purification of the crude product by column chromatography (SiO_2, 0-2% EtOAc in pentane) to afford *N*-allyl-N-benzyl-5-phenylpent-2-yn-1-amine as a colourless oil (0.83 g, 2.9 mmol, 96% yield).

Deallylation: the obtained tertiary amine (0.83 g, 2.9 mmol, 1 equiv.) was treated with $Pd(PPh_3)_4$ (67 mg, 57 µmol, 2 mol%) and 1,3-dimethylbarbituric acid (0.67 g, 4.3 mmol, 1.5 equiv.) in DCM (20 mL). Purification by flash column chromatography (SiO_2, 20-30% EtOAc in pentane) to afford *N*-benzyl-5-phenylpent-2-yn-1-amine (**1w**) as a straw-coloured oil (46 mg, 0.18 mmol, 6% yield).

R_f value: 0.16 (20% Ethyl acetate in Pentane).

^1H NMR (400 MHz, Chloroform-*d*) δ 7.39 – 7.17 (m, 10H, Ar*H*), 3.81 (s, 2H, PhCH_2NH), 3.39 (t, *J* = 2.2 Hz, 2H, CH_2C≡C), 2.85 (t, *J* = 7.5 Hz, 2H, PhCH_2), 2.52 (tt, *J* = 7.5, 2.2 Hz, 2H, PhCH_2CH_2C≡C), 1.45 (br. s, 1H, N*H*).

$^{13}C\{^1H\}$ NMR (101 MHz, Chloroform-*d*) δ 140.9, 139.8, 128.6, 128.5, 128.5 (2C), 127.2, 126.4, 83.2, 78.9, 52.5, 37.9, 35.4, 21.0.

IR (cm^{-1}) 3321 (w), 3028 (w), 2924 (m), 2846 (w), 1604 (w), 1581 (w), 1493 (m), 1454 (m), 1331 (w), 1265 (w), 1157 (w), 1107 (w), 702 (s).

HRMS (ESI/QTOF) m/z: $[M + H]^+$ Calcd for $C_{18}H_{20}N^+$ 250.1590; Found 250.1589.

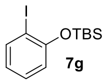

_N_-benzyl-5-phenylpent-2-yn-1-amine (7g)

Prepared following a literature procedure.[14] To a solution of 2-iodophenol (0.50 g, 2.3 mmol) and imidazole (0.32 g, 4.7 mmol) in anhydrous THF (5 mL) was added TBSCl (0.69 g, 4.5 mmol) in one portion and the reaction mixture was stirred at room temperature for 1 h. The mixture was then diluted with CH_2Cl_2 (10 mL) and was filtered through celite. The solvents were removed under reduced pressure, and the residue was purified by column chromatography on silica gel (Pentane) to provide the desired product as a colorless oil (0.69 g, 2.1 mmol, 91 % yield.

R_f value: 0.84 (Pentane).

^1H NMR (400 MHz, Chloroform-*d*) δ 7.76 (dd, *J* = 7.9, 1.6 Hz, 1H, Ar*H*), 7.20 (ddd, *J* = 8.1, 7.3, 1.7 Hz, 1H, Ar*H*), 6.83 (dd, *J* = 8.1, 1.4 Hz, 1H, Ar*H*), 6.68 (ddd, *J* = 7.9, 7.3, 1.4 Hz, 1H, Ar*H*), 1.07 (s, 9H, Si-C-(CH_3)$_3$), 0.28 (s, 6H, Si-(CH_3)$_2$).

$^{13}C\{^1H\}$ NMR (101 MHz, Chloroform-*d*) δ 155.3, 139.7, 129.4, 122.9, 118.7, 90.7, 26.0, 18.5, -3.9.

^1H Spectral data was consistent with the values reported in literature,[15] but ^{13}C spectra wasn't previously reported.

C. Optimization Studies

C.1. Cyclization: Screening of ArI (1.4 eqv)

The optimization reactions were conducted on a 0.1 mmol scale (relative to the propargylic amine). Reactions were performed in 6 mL conical microwave vials equipped with Teflon-coated magnetic stirring bars. The vials were loaded with the palladium source, the base, and the ligand. Part of the solvent (300 μL) was added, and the mixture was stirred at the specified temperature for 10 minutes. The propargylic amine, tether, and the remaining solvent (200 μL) were then added and the reaction mixture was stirred for 16 hours. The crude mixture was filtered through a plug of deactivated silica eluting with 10 mL of pentane/EtOAc 9:1. The solvent was removed, and yields were determined by ^{1}HNMR analysis of the crude mixture using 1 equiv. of trichloroethylene as the internal standard (IS). The enantiomeric excess was determined by HPLC analysis of a pure sample of product obtained by preparative TLC purification (pentane/EtOAc 100:3). HPLC method: Daicel Chiralpak IB N-5 column, 99:1 hexane/IPA, flow rate 1 mL/min.: τ_1 = 7.0 min τ_2 = 8.5 min.

Table 1. Screening of ArI in the hydroalkoxylation of propargylic amine **1**.

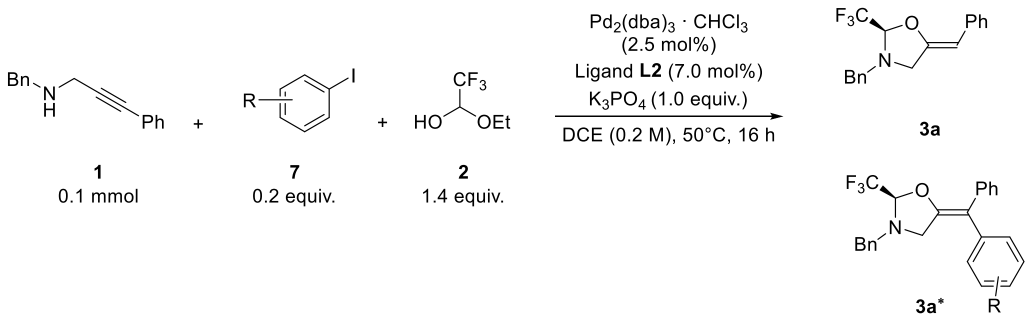

entry	ArI (1.4 eqv)	[%] yield **3a**	ee **3a**	[%] yield of 3**a***
1	p-H	23	94	23
2	p-MeO	14	89	17
2	m-MeO	20	94	20
3	p-CN	-	-	26
4	p-CF_3	5	78	24
5	o-Me	27	86	20
6	o-MeO	90	92	10
7	p-Me	13	90	15
9	2,6-$(MeO)_2$	49	70	13
10	o-F	90	86	10
11	o-CF_3	30	76	

C.2. Cyclization: Stoichiometry of ArI (o-MeO)

Table 2. Screening of the stoichiometry of ArI **7a** in the hydroalkoxylation of propargylic amine **1**.

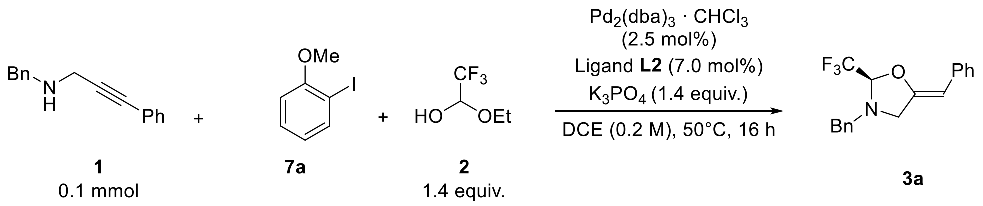

entry	Eqv o-MeOArI	[%] yield **3a**	ee **3a**

1	1.4	80	90
2	1.0	75	90
3	0.5	80	89
4	0.2	77	90
5	0.15	66	90
6	0.10	52	90

C.3. Cyclization: Ligand Screening

Table 3. Screening of ligands in the hydroalkoxylation of propargylic amine **1**.

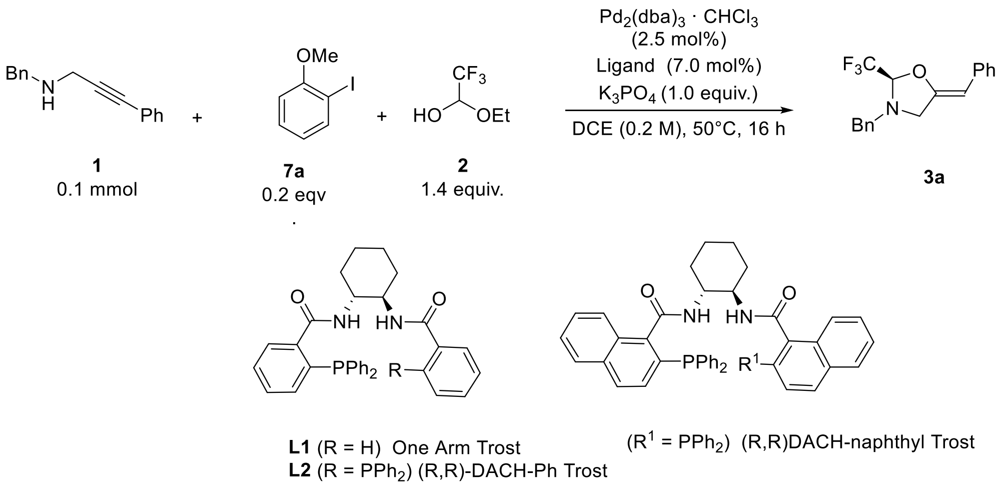

entry	Ligand	[%] yield **3a**	ee **3a**
1	(R,R)-DACH-Ph-Trost	90	90
2	“One arm”	13	38
3	(R)-BINAP	-	-
4	(R,R)-DACHNaphtyl Trost	<5	-
5	(R)-JosiPhos (Cy,tBu)	-	-
6	(R)-JosiPhos (Cy,Cy)	-	-
7	iPr-PHOX	-	-
8	(R)-MOP	80	<5
9	(R)-SIPHOS PE	50	<5
10	(R)-DM SEFPHOS	-	-
11	(R,R)-ADEN-Ph Trost	5	8

C.4. Cyclization: Screening of Solvents and Temperatures

Table 4. Screening of solvents and temperatures in the hydroalkoxylation of propargylic amine **1**.

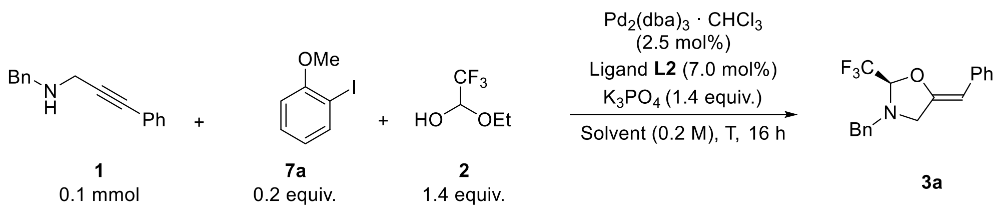

entry	Solvent (Temperature)	[%] yield **3a**	ee **3a**
1	DCE (50°C)	77	90
2	Toluene (50°C)	<5	-
3	THF (50°C)	17	-
4	MeCN (50°C)	8	-
5	PhCl (50°C)	78	86
6	Chloroform (50°C)	62	86
7	Hexane (50°C)	64	56
8	DMSO (50°C)	5	0
9	DMF (50°C)	8	0
10	EtOAc (50°C)	45	86
11	Et_2O (50°C)	34	72
12	DCM (35°C)	63	92
13	DCM (50°C)	80	90

C.5. Cyclization: Stoichiometry of K_3PO_4

Table 5. Screening of the stoichiometry of K_3PO_4 in the hydroalkoxylation of propargylic amine **1**.

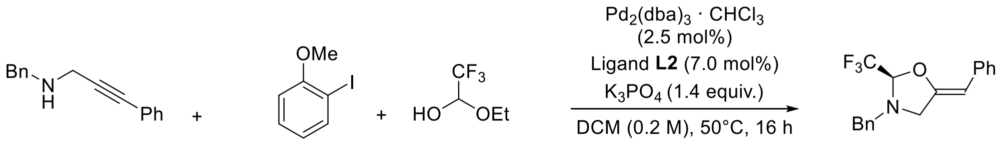

1 0.1 mmol; **7a** 0.2 equiv.; **2** 1.4 equiv.; **3a**

entry	Eqv K_3PO_4	[%] yield **3a**	ee **3a**
1	1.4	80	90
2	1	92	90
3	2	54	90
4	0.3	>95	87

C.6. Asymmetric Hydrogenation: Optimization Studies

The optimization reactions were performed in 25 mL round-bottom flask equipped with Teflon-coated magnetic stir bars. The flasks were loaded with the palladium catalyst and the olefin substrate closed with a septum and purged with nitrogen. The solvent mixture was added, and the suspension was stirred under a nitrogen flow for 10 minutes. Then, a balloon of hydrogen was connected to the flask with a needle and the reaction was stirred for 16 h at room temperature. The crude mixture was degassed bubbling nitrogen for 10 minutes and filtered through a plug of celite eluting with 10 mL of MeOH. The crude extract was washed with saturated $NaHCO_3$ and extracted with DCM (3x20 mL). The combined organic layers were dried over sodium sulfate, filtered, and concentrated in vacuum. Yields were determined by ^{1}HNMR analysis of the crude mixture using 1 equiv. of trichloroethylene as the internal standard (IS). The enantiomeric excess was determined by HPLC analysis of a pure sample obtained by preparative TLC purification (pentane/EtOAc 100:15). HPLC method: Daicel Chiralpak IA column, 95:5 hexane/IPA, flow rate 1 mL/min. τ_1 = 8.5 min, τ_2 = 10.8 min.

Table 6. Optimization of the hydrogenation of enol **3a**.

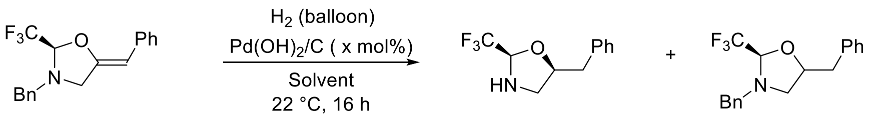

3a **4a** **4a***

entry	Solvent (0.05M)	[Pd] loading	M	additive	[%] yield **4a**	[%] yield **4a***	SM	ee of 4a[a]
1	MeOH/AcOH (2:1)	10	0.05	-	-	15	68	-
2	MeOH/AcOH (2:1)	20	0.05	-	-	60	25	-
3	MeOH/AcOH (2:1)	20	0.05	EtOAc (100 µL)	82	-	-	90%

a: ee starting material: 90%

D. Stereoselective Tethered Cyclization of Propargylic Amines

D.1. General Procedure for the Enantioselective Cyclization of Propargylic Amines

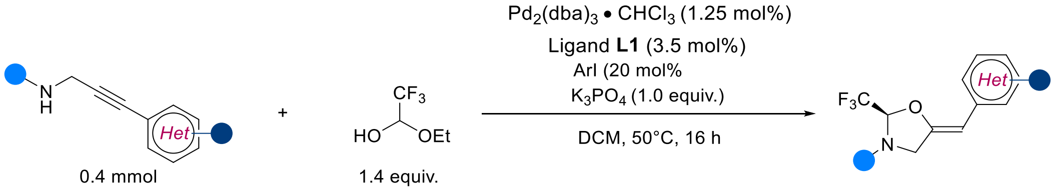

1 2 3

Scheme 7. Enantioselevtive Carboetherification of Propargylic Amines

An oven-dried 8 mL microwave vial equipped with a Teflon coated stirring bar was charged with $Pd_2(dba)_3$ • $CHCl_3$ (5.2 mg, 5.0 µmol, 1.25 mol%), the ligand (9.7 mg, 14 µmol, 3.5 mol%), K_3PO_4 (85 mg, 0.40 mmol, 1.0 equiv.) and, if solid, the propargylic amine (0.40 mmol, 1.0 equiv.). The vial was then sealed, purged with N_2 and placed in a heating metal block. 1.5 mL of DCM were added, and the suspension was stirred at room temperature for 10 minutes. 1-Iodo-2-methoxybenzene (**7a**, 10.5 µL, 800 µmol, 0.200 equiv.) was then added, followed by 0.5 mL of DCM and the mixture was stirred at room temperature for extra 10 minutes. 1-Ethoxy-2,2,2-trifluoroethanol (85% in EtOH, 76 uL, 0.56 mmol 1.4 equiv.) and, if liquid, the propargylic amine (0.40 mmol, 1.0 equiv.) were added, and the resulting suspension was stirred at 50 °C for 16 hours. Next, the reaction mixture was filtered through a plug of deactivated silica gel eluting with 15 mL of pentane/EtOAc 8:2 and concentrated in vacuo. The crude material was purified by flash column chromatography on silica gel to afford the corresponding product.

D.2. Characterization of Products of the Enantioselective Cyclization of Propargyl amines

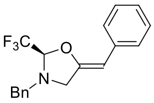

3a

(R,Z)-3-Benzyl-5-benzylidene-2-(trifluoromethyl)oxazolidine (3a)

Prepared according to the general procedure D1 using N-benzyl-3-phenylprop-2-yn-1-amine **1a** (87 µL, 0.40 mmol, 1.0 equiv.). The crude material was purified by flash column chromatography (pentane/EtOAc gradient 100:0 to 100:3) to give the corresponding olefin **3a** (106 mg, 0.332 mmol, 83% yield) as a white amorphous solid. The enantiomeric excess was determined to be 90% by HPLC analysis on a Daicel Chiralpak IB N-5 column: 99:1 hexane/IPA, flow rate 1 mL/min, λ = 254 nm: τ_{Minor} = 19.6 min τ_{Major} = 11.2 min. Absolute configuration was determined by X-Ray diffraction analysis of a single crystal of **3a** (details in section F).

R_f value: 0.56 (5% Ethyl acetate in Pentane).

$[\alpha]D^{20}$ = +25.7 (c = 0.49, $CHCl_3$, 90% ee).

^{1}H NMR (400 MHz, Chloroform-*d*) δ 7.59 – 7.54 (m, 2H, Ar*H*), 7.42 – 7.30 (m, 7H, Ar*H*), 7.21 – 7.15 (m, 1H, Ar*H*), 5.36 (br.s., 1H, vinyl C*H*), 5.17 (q, *J* = 5.3 Hz, 1H, C*H*CF_3), 4.11 – 4.03 (d, *J* = 15.5 Hz, 1H, NCH_aH_bC=C), 3.99 (d, *J* = 13.1 Hz, 1H, $PhCH_aH_b$), 3.91 (d, *J* = 13.1 Hz, 1H, $PhCH_aH_b$), 3.61 (d, *J* = 15.5 Hz, 1H, NCH_aH_bC=C).

$^{13}C\{^1H\}$ NMR (101 MHz, Chloroform-*d*) δ 150.2, 137.0, 135.4, 128.9, 128.8, 128.5, 128.1, 127.8, 126.0, 122.7 (q, *J* = 283.5 Hz) 98.8, 95.0 (q, *J* = 34.5 Hz), 60.5, 55.5.

$^{19}F\{^1H\}$ NMR (376 MHz, Chloroform-*d*) δ -80.3.

IR (cm^{-1}) 3028 (w), 1693 (m), 1496 (w), 1450 (w), 1369 (w), 1296 (m), 1173 (s), 1146 (s).

HRMS (ESI/QTOF) m/z: $[M + H]^+$ Calculated for $C_{18}H_{17}F_3NO^+$ 320.1257; Found 320.1248.

3 mmol scale reaction. The model reaction was repeated on 3 mmol scale. An oven dried 25 mL sealed tube equipped with a Teflon stir bar was charged with $Pd_2(dba)_3$ • $CHCl_3$ (39 mg, 38 µmol, 1.25 mol%), the ligand (73 mg, 0.11 mmol, 3.5 mol%) and K_3PO_4 (637 mg, 3.00 mmol, 1.00 equiv.). The tube was then purged with N_2.and sealed 11 mL of DCM were added under a nitrogen flow and the suspension was stirred at room temperature for 10 minutes. 1-Iodo-2-methoxybenzene **(7a)** (78 µL, 0.60 mmol, 0.20 equiv.) was then added under nitrogen flow, followed by 4 mL of DCM. The mixture was stirred at room temperature for extra 10 minutes. 1-ethoxy-2,2,2-trifluoroethanol **2** (85% in EtOH, 575 uL, 4.20 mmol 1.40 equiv.) and N-benzyl-3-phenylprop-2-yn-1-amine **1a** (650 µL, 3.00 mmol, 1.00 equiv.) were added under a nitrogen flow, the tube was sealed, and the resulting suspension was stirred at 50 °C for 16 hours. Then, the reaction mixture was

filtered through a plug of silica gel eluting with 150 mL of pentane/EtOAc 8:2 and concentrated in vacuo and analyzed by ^{1}H NMR with an internal standard (trichloroethylene, 0.33 equiv., NMR yield: =90%). The crude material was purified by flash column chromatography (pentane/EtOAc gradient 100:0 to 100:3) to give the corresponding product **3a** (783 mg, 2.45 mmol, 82% yield) as a white solid. The enantiomeric excess was determined to be 92% by HPLC analysis on a Daicel Chiralpak IB N-5 column: 99:1 hexane/IPA, flow rate 1 mL/min, λ = 254 nm: τ_{Minor} = 19.6 min τ_{Major} = 11.2 min.

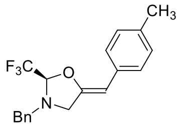

3b

(R,Z)-3-Benzyl-5-(4-methylbenzylidene)-2-(trifluoromethyl)oxazolidine (3b)

Prepared according to the general procedure D1 using N-benzyl-3-(p-tolyl)prop-2-yn-1-amine **1b** (94 mg, 0.40 mmol, 1.0 equiv.). The crude material was purified by flash column chromatography (pentane/EtOAc gradient 100:0 to 97:3) to give the corresponding olefin **3b** (107 mg, 0.321 mmol, 80% yield) as pale yellow amorphous solid. The enantiomeric excess was determined to be 90% by HPLC analysis on a Daicel Chiralpak IB N-5 column: 99:1 hexane/IPA, flow rate 1 mL/min, λ = 254 nm: τ_{Minor} = 14.5 min, τ_{Major} = 11.1 min. Absolute configuration determined in comparison to compound **3a.**

R_f value: 0.58 (5% Ethyl acetate in Pentane).

[α]D^{20} = 21.8 (c = 0.49, $CHCl_3$, 90% ee).

^{1}H NMR (400 MHz, Chloroform-d) δ 7.47 – 7.41 (m, 2H, Ar*H*), 7.41 – 7.28 (m, 5H, Ar*H*), 7.13 (d, *J* = 8.0 Hz, 2H, Ar*H*), 5.32 (s, 1H, vinyl C*H*), 5.15 (q, *J* = 5.4 Hz, 1H, C*H*CF_3), 4.04 (d, *J* = 15.4 Hz, 1H, NCH_aH_bC=C), 3.98 (d, *J* = 13.1 Hz, 1H, PhCH_aH_b), 3.90 (d, *J* = 13.1 Hz, 1H, PhCH_aH_b), 3.59 (d, *J* = 15.3, 1H, NCH_aH_bC=C), 2.34 (s, 3H, CH_3).

^{13}C{^{1}H} NMR (101 MHz, Chloroform-d) δ 149.5, 137.1, 135.7, 132.5, 129.2, 128.9, 128.8, 128.07, 127.7, 122.7 (q, *J* = 283.5 Hz), 98.7, 94.8 (q, *J* = 34.4 Hz), 60.5, 55.4, 21.3.

^{19}F{^{1}H} NMR (376 MHz, Chloroform-*d*) δ -80.4 (s, 3F, CHCF_3).

IR (cm^{-1}) 3028 (w), 2927 (w), 1693 (m), 1512 (w), 1454 (w), 1369 (w), 1296 (m), 1173 (s), 1146 (s), 1018 (m), 976 (m), 837 (m), 752 (w), 702 (m).

HRMS (ESI/QTOF) m/z: [M + H]$^+$ Calcd for $C_{19}H_{19}F_3NO^+$ 334.1413; Found 334.1417.

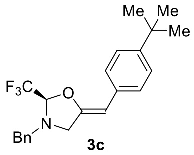

3c

(R,Z)-3-Benzyl-5-(4-(tert-butyl)benzylidene)-2-(trifluoromethyl)oxazolidine (3c)

Prepared according to the general procedure D1 using N-benzyl-3-(4-(tert-butyl)phenyl)prop-2-yn-1-amine **1c** (111 mg, 0.400 mmol, 1.00 equiv.). The crude material was purified by flash column chromatography (pentane/EtOAc gradient 100:0 to 97:3) to give the corresponding olefin **3c** (122 mg, 0.325 mmol, 81% yield) as a dark red liquid. The enantiomeric excess was determined to be 84% by HPLC analysis on a Daicel Chiralpak IB N-5 column: 99:1 hexane/IPA, flow rate 1 mL/min, λ = 254 nm: τ_{Minor} = 12.2 min, τ_{Major} = 9.2 min. Absolute configuration determined in comparison to compound **3a.**

R_f value: 0.60 (5% Ethyl acetate in Pentane).

[α]D^{20} = 2.85 (c = 0.48, $CHCl_3$, 84% ee).

^{1}H NMR (400 MHz, $CDCl_3$) δ 7.52 – 7.47 (m, 2H, Ar*H*), 7.40 – 7.35 (m, 5H, Ar*H*), 7.35 – 7.29 (m, 2H, Ar*H*), 5.34 (s, 1H, vinyl C*H*), 5.14 (q, *J* = 5.4 Hz, 1H, C*H*CF_3), 4.05 (d, *J* = 15.3, 1H, NCH_aH_bC=C), 3.97 (d, *J* = 13.1 Hz, 1H, PhCH_aH_b), 3.89 (d, *J* = 13.1 Hz, 1H, PhCH_aH_b), 3.59 (d, *J* = 15.4, 1H, NCH_aH_bC=C), 1.32 (s, 9H, C(CH_3)$_3$).

^{13}C{^{1}H} NMR (101 MHz, Chloroform-d) δ 149.7, 148.9, 137.1, 132.6, 128.9, 128.8, 128.1, 127.5, 125.4, 122.7 (q, *J* = 283.6 Hz), 98.6, 94.8 (q, *J* = 34.3 Hz), 60.5, 55.4, 34.6, 31.5.

^{19}F{^{1}H} NMR (376 MHz, Chloroform-*d*) δ -80.3 (s, 3F, CHCF_3).

IR (cm^{-1}) 2958 (m), 2866 (w), 1693 (m), 1458 (w), 1369 (m), 1296 (m), 1173 (s), 1149 (s), 1014 (w), 972 (m), 849 (w), 756 (w), 702 (m).

HRMS (ESI/QTOF) m/z: [M + H]$^+$ Calcd for $C_{22}H_{25}F_3NO^+$ 376.1883; Found 376.1879.

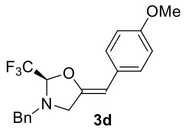

3d

(R,Z)-3-Benzyl-5-(4-methoxybenzylidene)-2-(trifluoromethyl)oxazolidine (3d)

Prepared according to the general procedure D1 using N-benzyl-3-(4-(methoxy)phenyl)prop-2-yn-1-amine **1d** (101 mg, 0.400 mmol, 1.0 equiv.). The crude material was purified by flash column chromatography (pentane/EtOAc gradient 100:0 to 97:3) to give the corresponding olefin **3d** (100 mg, 0.286 mmol, 72% yield) as an

orange oil. The enantiomeric excess was determined to be 84% by HPLC analysis on a Daicel Chiralpak IB N-5 column: 99:1 hexane/IPA, flow rate 1 mL/min, λ = 254 nm: τ_{Minor} = 15.1 min, τ_{Major} = 10.7 min. Absolute configuration determined in comparison to compound **3a.**

R_f value: 0.42 (5% Ethyl acetate in Pentane).

[α]D^{20} = 13.9 (c = 0.48, $CHCl_3$, 84% ee).

^{1}H NMR (400 MHz, Chloroform-d) δ 7.49 (d, *J* = 8.8 Hz, 2H, Ar*H*), 7.43 – 7.29 (m, 5H, Ar*H*), 6.88 (d, *J* = 8.8 Hz, 2H, Ar*H*), 5.30 (s, 1H, vinyl C*H*), 5.14 (q, *J* = 5.4 Hz, 1H, C*H*CF_3), 4.04 (d, *J* = 15.3 Hz, 1H, NCH_aH_bC=C), 3.98 (d, *J* = 13.1 Hz, 1H, PhCH_aH_b), 3.90 (d, *J* = 13.1 Hz, 1H, PhCH_aH_b), 3.81 (s, 3H, OCH_3), 3.58 (d, *J* = 15.3 Hz, 1H, NCH_aH_bC=C).

^{13}C{^{1}H} NMR (101 MHz, Chloroform-d) δ 157.8, 148.5, 137.1, 129.0, 128.8, 128.8, 128.2, 128.1, 122.7 (q, *J* = 283.5 Hz), 114.0, 98.3, 94.7 (q, *J* = 34.3 Hz), 60.4, 55.4, 55.3.

^{19}F{^{1}H} NMR (376 MHz, Chloroform-*d*) δ -80.4.

IR (cm^{-1}) 2947 (w), 1693 (m), 1608 (m), 1512 (m), 1454 (m), 1292 (m), 1250 (s), 1173 (s), 1149 (s).

HRMS (nanochip-ESI/LTQ-Orbitrap) m/z: $[M + H]^+$ Calculated for $C_{19}H_{19}F_3NO_2^+$ 350.1362; Found 350.1356.

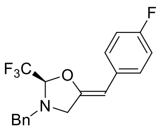

3e

(R,Z)-3-Benzyl-5-(4-fluorobenzylidene)-2-(trifluoromethyl)oxazolidine (3e)

Prepared according to the general procedure D1 using N-benzyl-3-(4-fluorophenyl)prop-2-yn-1-amine **1e** (96 mg, 0.40 mmol, 1.0 equiv.). The crude material was purified by flash column chromatography (pentane/EtOAc gradient 100:0 to 100:3) to give the corresponding olefin **3e** (112 mg, 0.332 mmol, 83% yield) as a pale yellow amorphous solid. The enantiomeric excess was determined to be 92% by HPLC analysis on a Daicel Chiralpak IB N-5 column: 99:1 hexane/IPA, flow rate 1 mL/min, λ = 254 nm: τ_{Minor} = 16.4 min τ_{Major} = 12.3 min. Absolute configuration was determined by X-Ray diffraction analysis of a single crystal of **3a**.

R_f value: 0.62 (5% Ethyl acetate in Pentane).

[α]D^{20} = +13.6 (c = 0.39, $CHCl_3$, 92% ee).

^{1}H NMR (400 MHz, Chloroform-*d*) δ 7.56 – 7.46 (m, 2H, Ar*H*), 7.43 – 7.27 (m, 5H, Ar*H*), 7.08 – 6.95 (m, 2H, Ar*H*), 5.32 (s, 1H, vinyl C*H*), 5.16 (q, *J* = 5.4 Hz, 1H, C*H*CF_3), 4.04 (d, *J* = 15.4 Hz, 1H, NCH_aH_bC=C), 3.99 (d, *J* = 13.1 Hz, 1H, PhCH_aH_b), 3.90 (d, *J* = 13.1 Hz, 1H, PhCH_aH_b), 3.59 (d, *J* = 15.5, 1H, NCH_aH_bC=C).

^{13}C{^{1}H} NMR (101 MHz, Chloroform-*d*) δ 162.3, 159.9, 149.8 (d, *J* = 2.5 Hz), 137.0, 131.5 (d, *J* = 3.3 Hz), 129.3 (d, *J* = 7.7 Hz), 128.8 (d, *J* = 1.6 Hz), 128.1, 122.7 (q, *J* = 283.3 Hz), 115.3 (d, *J* = 21.3 Hz), 97.8, 94.9 (q, *J* = 34.4 Hz), 60.5, 55.3.

^{19}F{^{1}H} NMR (376 MHz, Chloroform-*d*) δ -80.4 (s, 3F, CHCF_3), -116.3 (s, 1F, Ar*F*).

IR (cm^{-1}) 3035 (w), 2846 (w), 1693 (m), 1508 (m), 1296 (m), 1227 (m), 1146 (s), 1014 (m), 976 (m), 845 (m), 702 (m).

HRMS (nanochip-ESI/LTQ-Orbitrap) m/z: [M + H]+ Calcd for C18H16F4NO+ 338.1163; Found 338.1165.

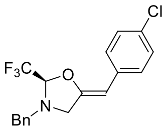

3f

(R,Z)-3-Benzyl-5-(4-chlorobenzylidene)-2-(trifluoromethyl)oxazolidine (3f)

Prepared according to the general procedure D1 using N-benzyl-3-(4-chlorophenyl)prop-2-yn-1-amine **1f** (102 mg, 0.400 mmol, 1.00 equiv.). The crude material was purified by flash column chromatography (pentane/EtOAc gradient 100:0 to 100:3) to give the corresponding olefin **3f** (123 mg, 0.348 mmol, 87% yield) as an orange liquid. The enantiomeric excess was determined to be 90% by HPLC analysis on a Daicel Chiralpak IB N-5 column: 99:1 hexane/IPA, flow rate 1 mL/min, λ = 254 nm: τ_{Minor} = 16.8 min τ_{Major} = 13.3 min. Absolute configuration was determined by X-Ray diffraction analysis of a single crystal of **3a**.

R_f value: 0.58 (5% Ethyl acetate in Pentane).

[α]D^{20} = +36.4 (c = 0.6, $CHCl_3$, 90% ee).

^{1}H NMR (400 MHz, Chloroform-*d*) δ 7.51 – 7.42 (m, 2H, Ar*H*), 7.42 – 7.30 (m, 5H, Ar*H*), 7.30 – 7.24 (m, 2H, Ar*H*), 5.31 (s, 1H, vinyl C*H*), 5.18 (q, *J* = 5.3 Hz, 1H, C*H*CF_3), 4.05 (d, *J* = 15.6 Hz, 1H, NCH_aH_bC=C), 3.99 (d, *J* = 13.1 Hz, 1H, PhCH_aH_b), 3.90 (d, *J* = 13.1 Hz, 1H, PhCH_aH_b), 3.60 (d, *J* = 15.6, 1H, NCH_aH_bC=C).

^{13}C{^{1}H} NMR (101 MHz, Chloroform-*d*) δ 150.8, 136.9, 133.9, 131.4, 129.0, 128.8 (2C), 128.6, 128.2, 122.6 (q, *J* = 283.5 Hz), 97.8, 95.1 (q, *J* = 34.5 Hz), 60.5, 55.5.

^{19}F{^{1}H} NMR (376 MHz, Chloroform-*d*) δ -80.4 (s, 3F, CHCF_3).

IR (cm^{-1}) 3035 (w), 2935 (w), 1689 (m), 1493 (m), 1369 (w), 1296 (m), 1176 (s), 1146 (s), 1088 (m), 1014 (m), 972 (m), 845 (m), 702 (m), 752 (w).

HRMS (ESI/QTOF) m/z: $[M + H]^+$ Calcd for $C_{18}H_{16}ClF_3NO^+$ 354.0867; Found 354.0862.

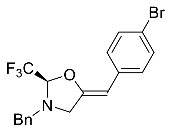

3g

(R,Z)-3-Benzyl-5-(4-bromobenzylidene)-2-(trifluoromethyl)oxazolidine (3g)

Prepared according to the general procedure D1 using N-benzyl-3-(4-bromophenyl)prop-2-yn-1-amine **1g** (120 mg, 0.400 mmol, 1.00 equiv.). The crude material was purified by flash column chromatography (pentane/EtOAc gradient 100:0 to 100:3) to give the corresponding olefin **3g** (138 mg, 0.346 mmol, 87% yield) as a white amorphous solid. The enantiomeric excess was determined to be 94% by HPLC analysis on a Daicel Chiralpak IB N-5 column: 99:1 hexane/IPA, flow rate 1 mL/min, λ = 254 nm: τ_{Minor} = 18.1 min τ_{Major} = 14.4 min. Absolute configuration was determined by X-Ray diffraction analysis of a single crystal of **3a**.

R_f value: 0.57 (5% Ethyl acetate in Pentane).

$[\alpha]D^{20}$ = +29.98 (c = 0.54, $CHCl_3$, 94% ee).

^{1}H NMR (400 MHz, Chloroform-*d*) δ 7.48 – 7.27 (m, 9H, Ar*H*), 5.29 (s, 1H, vinyl C*H*), 5.18 (q, *J* = 5.3 Hz, 1H, C*H*CF$_3$), 4.07 – 4.01 (d, *J* = 15.6, 1H, NCH_aH_bC=C), 3.99 (d, *J* = 13.1 Hz, 1H, PhCH_aH_b), 3.90 (d, *J* = 13.1 Hz, 1H, PhCH_aH_b), 3.60 (d, *J* = 15.6, 1H, NCH_aH_bC=C).

^{13}C{^{1}H} NMR (101 MHz, Chloroform-*d*) δ 151.0, 136.9, 134.3, 131.5, 129.4, 128.8 (2C), 128.2, 122.6 (q, *J* = 283.4 Hz), 119.5, 97.8, 95.1 (q, *J* = 34.6 Hz), 60.5, 55.5.

^{19}F{^{1}H} NMR (376 MHz, Chloroform-*d*) δ -80.4 (s, 3F, CHCF_3).

IR (cm^{-1}) 2931 (w), 2854 (w), 1689 (m), 1489 (m), 1296 (m), 1176 (s), 1146 (s), 1076 (m), 1011 (m), 972 (m), 841 (m), 702 (m).

HRMS (ESI/QTOF) m/z: $[M + H]^+$ Calcd for $C_{18}H_{16}BrF_3NO^+$ 398.0362; Found 398.0348.

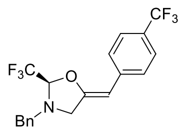

3h

(R,Z)-3-Benzyl-2-(trifluoromethyl)-5-(4-(trifluoromethyl)benzylidene)oxazolidine (3h)

Prepared according to the general procedure D1 using N-benzyl-3-(4-(trifluoromethyl)phenyl)prop-2-yn-1-amine **1h** (116 mg, 0.400 mmol, 1.0 equiv.). The crude material was purified by flash column chromatography (pentane/EtOAc gradient 100:0 to 97:3) to give the corresponding olefin **3h** (128 mg, 0.330 mmol, 83% yield) as colorless oil. The enantiomeric excess was determined to be 92% by HPLC analysis on a Daicel Chiralpak IB N-5 column: 99:1 hexane/IPA, flow rate 1 mL/min, λ = 254 nm: τ_{Minor} = 19.0 min, τ_{Major} = 11.2 min. Absolute configuration determined in comparison to compound **3a.**

R_f value: 0.65 (5% Ethyl acetate in Pentane).

$[\alpha]D^{20}$ = 34.5 (c = 0.50, $CHCl_3$, 92% ee).

^{1}H NMR (400 MHz, Chloroform-d) δ 7.63 (d, *J* = 8.2 Hz, 2H, Ar*H*), 7.56 (d, *J* = 8.3 Hz, 2H, Ar*H*), 7.41 – 7.30 (m, 5H, Ar*H*), 5.39 (s, 1H, vinyl C*H*), 5.22 (q, *J* = 5.3 Hz, 1H, C*H*CF$_3$), 4.09 (d, *J* = 15.7 Hz, 1H, NCH_aH_bC=C), 4.00 (d, *J* = 13.1 Hz, 1H, PhCH_aH_b), 3.92 (d, *J* = 13.1 Hz, 1H, PhCH_aH_b), 3.65 (d, *J* = 15.8 Hz, 1H, NCH_aH_bC=C).

^{13}C{^{1}H} NMR (101 MHz, Chloroform-d) δ 152.5, 139.0, 136.8, 128.88, 128.86, 128.2, 127.8, 127.5, 125.40 (q, *J* = 3.7 Hz), 124.37 (q, *J* = 271.6 Hz), 122.42 (q, *J* = 283.5 Hz), 97.8, 95.2 (q, *J* = 34.7 Hz), 60.5, 55.7.

^{19}F{^{1}H} NMR (376 MHz, Chloroform-*d*) δ -62.3 (s, 3F, ArCF_3), -80.4 (s, 3F, CHCF_3).

IR (cm^{-1}) 1689 (m), 1616 (w), 1454 (w), 1415 (w), 1369 (w), 1327 (s), 1146 (s).

HRMS (nanochip-ESI/LTQ-Orbitrap) m/z: $[M + H]^+$ Calculated for $C_{19}H_{16}F_6NO^+$ 388.1131; Found 388.1126.

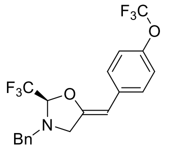

3i

(R,Z)-3-benzyl-5-(4-(trifluoromethoxy)benzylidene)-2-(trifluoromethyl)oxazolidine (3i)

Prepared according to the general procedure D1 using N-benzyl-3-(4-(trifluoromethoxy)phenyl)prop-2-yn-1-amine **1i** (122 mg, 0.400 mmol, 1.00 equiv.). The crude material was purified by flash column chromatography (pentane/EtOAc gradient 100:0 to 100:3) to give the corresponding olefin **3i** (136 mg, 0.337 mmol, 84% yield) as an orange oil. The enantiomeric excess was determined to be 90% by HPLC analysis on a Daicel Chiralpak IB

N-5 column: 99:1 hexane/IPA, flow rate 1 mL/min, λ = 254 nm: τ_{Minor} = 14.5 min τ_{Major} = 9.6 min. Absolute configuration was determined by X-Ray diffraction analysis of a single crystal of **3a**.

R_f value: 0.56 (5% Ethyl acetate in Pentane).

[α]D^{20} = +1.26 (c = 0.49, $CHCl_3$, 92% ee).

^{1}H NMR (400 MHz, Chloroform-*d*) δ 7.59 – 7.51 (m, 2H, Ar*H*), 7.42 – 7.28 (m, 5H, Ar*H*), 7.20 – 7.12 (m, 2H, Ar*H*), 5.34 (s, 1H, vinyl C*H*), 5.18 (q, *J* = 5.4 Hz, 1H, C*H*CF_3), 4.06 (d, *J* = 15.7 Hz, 1H, NCH_aH_bC=C), 3.99 (d, *J* = 13.1 Hz, 1H, PhCH_aH_b), 3.90 (d, *J* = 13.1 Hz, 1H, PhCH_aH_b), 3.62 (d, *J* = 15.7 Hz, 1H, NCH_aH_bC=C).

^{13}C{^{1}H} NMR (101 MHz, Chloroform-*d*) δ 150.9, 147.1, 136.9, 134.2, 129.0, 128.9 (2C), 128.2, 122.6 (q, *J* = 283.4 Hz), 121.1, 120.7 (q, *J* = 260 Hz), 97.6, 95.1 (q, *J* = 34.6 Hz), 60.5, 55.5.
^{19}F{^{1}H} NMR (376 MHz, Chloroform-*d*) δ -57.9 (s, 3F, ArOCF_3), -80.4 (s, 3F, CHCF_3).
IR (cm^{-1}) 2931 (w), 2854 (w), 1689 (w), 1508 (w), 1261 (s), 1173 (s), 972 (w), 856 (w), 702 (w).

HRMS (ESI/QTOF) m/z: $[M + H]^+$ Calcd for $C_{19}H_{16}F_6NO_2^+$ 404.1080; Found 404.1079.

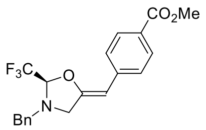

3j

Methyl (R,Z)-4-((3-benzyl-2-(trifluoromethyl)oxazolidin-5-ylidene)methyl) benzoate (3j)

Prepared according to the general procedure D1 using methyl 4-(3-(benzylamino)prop-1-yn-1-yl)benzoate (112 mg, 0.400 mmol, 1.0 equiv.). The crude material was purified by flash column chromatography (pentane/EtOAc gradient 100:0 to 90:10) to give the corresponding olefin **3j** (117 mg, 0.311 mmol, 78% yield) as a pale-yellow solid. The enantiomeric excess was determined to be 92% by HPLC analysis on a Daicel Chiralpak IA column: 95:5 hexane/IPA, flow rate 1 mL/min, λ = 254 nm: τ_{Minor} = 10.0 min, τ_{Major} = 8.2 min. Absolute configuration determined in comparison to compound **3a.**

R_f value: 0.35 (5% Ethyl acetate in Pentane).

[α]D^{20} = 46.9 (c = 0.49, $CHCl_3$, 92% ee).

^{1}H NMR (400 MHz, Chloroform-d) δ 7.98 (d, *J* = 8.5 Hz, 2H, Ar*H*), 7.59 (d, *J* = 8.6 Hz, 2H, Ar*H*), 7.48 – 7.27 (m, 5H, Ar*H*), 5.40 (s, 1H, vinyl C*H*), 5.23 (q, *J* = 5.3 Hz, 1H, C*H*CF_3), 4.09 (d, *J* = 14.8 Hz, 1H, NCH_aH_bC=C), 4.00 (d, *J* = 13.1 Hz, 1H, PhCH_aH_b), 3.92 (s + d, *J* = 9.3 Hz, 4H, PhCH_aH_b + OCH_3), 3.65 (d, *J* = 15.9 Hz, 1H, NCH_aH_bC=C).

^{13}C{^{1}H} NMR (101 MHz, Chloroform-d) 167.2, 152.6, 140.1, 136.8, 129.9, 128.9 (2 x C), 128.2, 127.6, 127.2, 122.6 (q, *J* = 283.5 Hz), 98.2, 95.4 (q, *J* = 34.6 Hz), 60.5, 55.8, 52.1.

^{19}F{^{1}H} NMR (376 MHz, Chloroform-*d*) δ -80.3.

IR (cm^{-1}) 1716 (s), 1608 (m), 1442 (m), 1373 (w), 1284 (s), 2951 (w), 3028 (w), 1180 (s), 1146 (s).

HRMS (ESI/QTOF) m/z: $[M + H]^+$ Calculated for $C_{20}H_{19}F_3NO_3^+$ 378.1312; Found 378.1320.

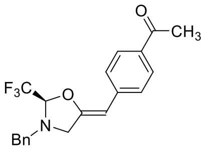

3k

(R,Z)-1-(4-((3-Benzyl-2-(trifluoromethyl)oxazolidin-5-ylidene)methyl)phenyl)ethan-1-one (3k)

Prepared according to the general procedure D1 using 1-(4-(3-(benzylamino)prop-1-yn-1-yl)phenyl)ethan-1-one **1k** (105 mg, 0.400 mmol, 1.00 equiv.)**.** The crude material was purified by flash column chromatography (pentane/EtOAc gradient 100:0 to 100:3) to give the corresponding olefin **3k** (105 mg, 0.291 mmol, 73% yield) as an orange liquid. The enantiomeric excess was determined to be 88% by HPLC analysis on a Daicel Chiralpak IB N-5 column: 95:5 hexane/IPA, flow rate 1 mL/min, λ = 214 nm: τ_{Minor} = 26.7 min τ_{Major} = 19.4 min. Absolute configuration was determined by X-Ray diffraction analysis of a single crystal of **3a**.

R_f value: 0.48 (5% Ethyl acetate in Pentane).

[α]D^{20} = +12.86 (c = 0.48, $CHCl_3$, 88% ee).

^{1}H NMR (400 MHz, Chloroform-*d*) δ 7.96 – 7.87 (m, 2H, Ar*H*), 7.65 – 7.57 (m, 2H, Ar*H*), 7.42 – 7.36 (m, 4H, Ar*H*), 7.34 (ddt, *J* = 8.9, 5.2, 2.5 Hz, 1H, Ar*H*), 5.41 (s, 1H, vinyl C*H*), 5.23 (q, *J* = 5.3 Hz, 1H, C*H*CF_3), 4.09 (d, *J* = 15.9, 1H, NCH_aH_bC=C), 4.00 (d, *J* = 13.1 Hz, 1H, PhCH_aH_b), 3.92 (d, *J* = 13.1 Hz, 1H, PhCH_aH_b), 3.66 (d, *J* = 15.9, 1H, NCH_aH_bC=C), 2.59 (s, 3H, COCH_3).

^{13}C{^{1}H} NMR (101 MHz, Chloroform-*d*) δ 197.7, 152.9, 140.4, 136.8, 134.4, 128.9, 128.9, 128.8, 128.2, 127.7, 122.5 (q, *J* = 283.5 Hz), 98.2, 95.4 (q, *J* = 34.8 Hz), 60.5, 55.8, 26.7.
^{19}F{^{1}H} NMR (376 MHz, Chloroform-*d*) δ -80.3 (s, 3F, CHCF_3).

IR (cm^{-1}) 2927 (w), 2854 (w), 1678 (s), 1604 (m), 1277 (s), 1176 (s), 1149 (s), 968 (m), 856 (m), 706 (m).

HRMS (ESI/QTOF) m/z: $[M + H]^+$ Calcd for $C_{20}H_{19}F_3NO_2^+$ 362.1362; Found 362.1360.

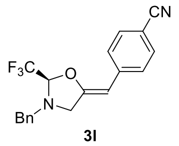

3l

(R,Z)-4-((3-Benzyl-2-(trifluoromethyl)oxazolidin-5-ylidene)methyl)benzonitrile (3l)

Prepared according to the general procedure D1 using 4-(3-(benzylamino)prop-1-yn-1-yl)benzonitrile (99 mg, 0.40 mmol, 1.0 equiv.). The crude material was purified by flash column chromatography (pentane/EtOAc gradient 100:0 to 90:10) to give the corresponding olefin **3l** (120 mg, 0.349 mmol, 87% yield) as a pale yellow oil. The enantiomeric excess was determined to be 90% by HPLC analysis on a Daicel Chiralpak IA column: 95:5 hexane/IPA, flow rate 1 mL/min, λ = 254 nm: τ_{Minor} = 11.9 min, τ_{Major} = 9.3 min. Absolute configuration determined in comparison to compound **3a.**

R_f value: 0.45 (5% Ethyl acetate in Pentane).

$[\alpha]D^{20}$ = 49.0 (c = 0.54, $CHCl_3$, 90% ee).

^{1}H NMR (400 MHz, Chloroform-d) δ 7.71 – 7.51 (m, 4H, Ar*H*), 7.44 – 7.29 (m, 5H, Ar*H*), 5.37 (s, 1H, vinyl C*H*), 5.24 (q, *J* = 5.3 Hz, 1H, C*H*CF$_3$), 4.09 (d, *J* = 16.0 Hz, 1H, NCH_aH$_b$C=C), 4.00 (d, *J* = 13.1 Hz, 1H, PhCH_aH$_b$), 3.92 (d, *J* = 13.0 Hz, 1H, PhCH$_a$$H_b$), 3.67 (d, *J* = 16.0 Hz, 1H, NCH$_a$$H_b$C=C).

^{13}C{^{1}H} NMR (101 MHz, Chloroform-d) δ 153.6, 140.1, 136.6, 132.3, 128.9, 128.8, 128.3, 128.1, 122.5 (q, *J* = 283.4 Hz), 119.5, 108.8, 97.7, 95.6 (q, *J* = 34.7 Hz), 60.5, 55.8.

^{19}F{^{1}H} NMR (376 MHz, Chloroform-*d*) -80.3.

IR (cm^{-1}) 2225 (w), 1685 (m), 1604 (m), 1504 (w), 1450 (w), 1373 (w), 1296 (m), 1176 (s), 1149 (s).

HRMS (ESI/QTOF) m/z: $[M + H]^+$ Calculated for $C_{19}H_{16}F_3N_2O^+$ 345.1209; Found 345.1213.

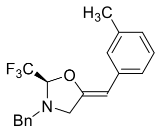

3m

(R,Z)-3-Benzyl-5-(3-methylbenzylidene)-2-(trifluoromethyl)oxazolidine (3m)

Prepared according to the general procedure D1 using N-benzyl-3-(m-tolyl)prop-2-yn-1-amine **1m** (94 mg, 0.40 mmol, 1.0 equiv.). The crude material was purified by flash column chromatography (pentane/EtOAc gradient 100:0 to 100:3) to give the corresponding olefin **3m** (106 mg, 0.318 mmol, 79% yield) as an orange liquid. The enantiomeric excess was determined to be 88% by HPLC analysis on a Daicel Chiralpak IB N-5 column: 99:1 hexane/IPA, flow rate 1 mL/min, λ = 254 nm: τ_{Minor} = 15.9 min τ_{Major} = 9.5 min. Absolute configuration was determined by X-Ray diffraction analysis of a single crystal of **3a**.

R_f value: 0.63 (5% Ethyl acetate in Pentane).

$[\alpha]D^{20}$ = +1.46 (c = 0.63, $CHCl_3$, 88% ee).

^{1}H NMR (400 MHz, Chloroform-*d*) δ 7.42 – 7.29 (m, 7H, Ar*H*), 7.22 (t, *J* = 7.6 Hz, 1H, Ar*H*), 6.99 (d, *J* = 7.5, 1H, Ar*H*), 5.32 (s, 1H, vinyl C*H*), 5.17 (q, *J* = 5.4 Hz, 1H, C*H*CF$_3$), 4.05 (d, *J* = 15.5, 1H, NCH_aH$_b$C=C), 3.98 (d, *J* = 13.1 Hz, 1H, PhCH_aH$_b$), 3.90 (d, *J* = 13.1 Hz, 1H, PhCH$_a$$H_b$), 3.60 (d, *J* = 15.6, 1H, NCH$_a$$H_b$C=C), 2.35 (s, 3H, C$H_3$).

^{13}C{^{1}H} NMR (101 MHz, Chloroform-*d*) δ 150.1, 138.0, 137.1, 135.3, 128.8, 128.5, 128.1, 126.8, 125.0, 122.7 (q, *J* = 283.3 Hz), 98.9, 94.9 (q, *J* = 34.5 Hz), 60.5, 55.5, 21.7.

^{19}F{^{1}H} NMR (376 MHz, Chloroform-*d*) δ -80.3 (s, 3F, CHCF_3).

IR (cm^{-1}) 3028 (m), 2951 (m), 2110 (s), 1697 (s), 1377 (s), 1142 (s), 1018 (s), 976 (s), 760 (s), 698 (s), 629 (s), 1604 (s).

HRMS (nanochip-ESI/LTQ-Orbitrap) m/z: $[M + H]^+$ Calcd for $C_{19}H_{19}F_3NO^+$ 334.1413; Found 334.1419.

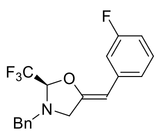

3n

(R,Z)-3-Benzyl-5-(3-fluorobenzylidene)-2-(trifluoromethyl)oxazolidine (3n)

Prepared according to the general procedure D1 using N-benzyl-3-(3-fluorophenyl)prop-2-yn-1-amine **1n** (96 mg, 0.40 mmol, 1.0 equiv.). The crude material was purified by flash column chromatography (pentane/EtOAc gradient 100:0 to 100:3) to give the corresponding olefin **3n** (120 mg, 0.356 mmol, 89% yield) as a white amorphous solid. The enantiomeric excess was determined to be 90% by HPLC analysis on a Daicel Chiralpak IB N-5 column: 99:1 hexane/IPA, flow rate 1 mL/min, λ = 254 nm: τ_{Minor} = 19.0 min τ_{Major} = 11.1 min. Absolute configuration was determined by X-Ray diffraction analysis of a single crystal of **3a**.

R_f value: 0.59 (5% Ethyl acetate in Pentane).

$[\alpha]D^{20}$ = +19.8 (c = 0.52, $CHCl_3$, 90% ee).

^{1}H NMR (400 MHz, Chloroform-*d*) δ 7.43 – 7.29 (m, 6H, Ar*H*), 7.29 – 7.19 (m, 2H, Ar*H*), 6.86 (ddt, *J* = 9.2, 5.2, 2.1 Hz, 1H, Ar*H*), 5.33 (s, 1H, vinyl C*H*), 5.20 (q, *J* = 5.4 Hz, 1H, C*H*CF$_3$), 4.06 (d, *J* = 15.7, 1H, NCH_aH$_b$C=C), 3.99 (d, *J* = 13.1 Hz, 1H, PhCH_aH$_b$), 3.91 (d, *J* = 13.1 Hz, 1H, PhCH$_a$$H_b$), 3.62 (d, *J* = 15.7, 1H, NCH$_a$$H_b$C=C).

^{13}C{^{1}H} NMR (101 MHz, Chloroform-*d*) δ 163.1 (d, *J* = 243.7 Hz), 151.4, 137.6 (d, *J* = 8.4 Hz), 136.9, 129.8 (d, *J* = 8.7 Hz), 128.9 (2C), 128.2, 123.5 (d, *J* = 2.8 Hz), 122.6 (q, *J* = 283.6 Hz), 114.4 (d, *J* = 22.5 Hz), 112.8 (d, *J* = 21.5 Hz), 98.0 (d, *J* = 2.7 Hz), 95.2 (q, *J* = 34.6 Hz), 60.5, 55.1.

^{19}F{^{1}H} NMR (376 MHz, Chloroform-*d*) δ -80.3 (s, 3F, CHC*F*$_3$), -113.7 (s, 1F, Ar*F*).

IR (cm^{-1}) 3035 (w), 2850 (w), 1689 (m), 1612 (m), 1581 (w), 1485 (w), 1446 (m), 1373 (w), 1292 (m), 1149 (s), 1014 (m), 968 (m), 879 (m), 698 (m), 752 (m).

HRMS (ESI/QTOF) m/z: [M + H]$^+$ Calcd for $C_{18}H_{16}F_4NO^+$ 338.1163; Found 338.1168.

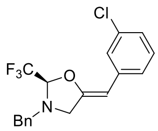

3o

(R,Z)-3-Benzyl-5-(3-chlorobenzylidene)-2-(trifluoromethyl)oxazolidine (3o)

Prepared according to the general procedure D1 using N-benzyl-3-(3-chlorophenyl)prop-2-yn-1-amine **1o** (102 mg, 0.400 mmol, 1.00 equiv.). The crude material was purified by flash column chromatography (pentane/EtOAc gradient 100:0 to 100:3) to give the corresponding olefin **3o** (116 mg, 0.328 mmol, 82% yield) as an orange liquid. The enantiomeric excess was determined to be 90% by HPLC analysis on a Daicel Chiralpak IB N-5 column: 99:1 hexane/IPA, flow rate 1 mL/min, λ = 254 nm: τ_{Minor} = 22.3 min τ_{Major} = 12.4 min. Absolute configuration was determined by X-Ray diffraction analysis of a single crystal of **3a**.

R$_f$ value: 0.63 (5% Ethyl acetate in Pentane).

[α]D^{20} = +28.4 (c = 0.5, CHCl$_3$, 90% ee).

^{1}H NMR (400 MHz, Chloroform-*d*) δ 7.55 (t, *J* = 1.9 Hz, 1H, Ar*H*), 7.42 – 7.36 (m, 5H, Ar*H*), 7.36 – 7.29 (m, 1H, Ar*H*), 7.23 (t, *J* = 7.9 Hz, 1H, Ar*H*), 7.13 (ddd, *J* = 8.0, 2.1, 1.1 Hz, 1H, Ar*H*), 5.30 (s, 1H, vinyl C*H*), 5.20 (q, *J* = 5.4 Hz, 1H, C*H*CF$_3$), 4.06 (d, *J* = 15.7 Hz, 1H, NCH_aH$_b$C=C), 3.98 (d, *J* = 13.1 Hz, 1H, PhCH_aH$_b$), 3.90 (d, *J* = 13.1 Hz, 1H, PhCH$_a$$H_b$), 3.62 (d, *J* = 15.7, 1H, NCH$_a$$H_b$C=C).

^{13}C{^{1}H} NMR (101 MHz, Chloroform-*d*) δ 151.5, 137.2, 136.9, 134.3, 129.7, 128.9 (2C), 128.2, 127.7, 125.9 (2C), 122.6 (q, *J* = 283.6 Hz), 97.7, 95.2 (q, *J* = 34.6 Hz), 60.5, 55.5.

^{19}F{^{1}H} NMR (376 MHz, Chloroform-*d*) δ -80.3 (s, 3F, CHC*F*$_3$).

IR (cm^{-1}) 3066 (w), 2939 (w), 1689 (m), 1593 (w), 1296 (m), 1176 (s), 1146 (s), 972 (m), 698 (m), 887 (m), 1466 (w), 1369 (w), 1084 (m).

HRMS (ESI/QTOF) m/z: [M + H]$^+$ Calcd for $C_{18}H_{16}ClF_3NO^+$ 354.0867; Found 354.0865.

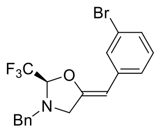

3p

(R,Z)-3-Benzyl-5-(3-bromobenzylidene)-2-(trifluoromethyl)oxazolidine (3p)

Prepared according to the general procedure D1 using N-benzyl-3-(3-bromophenyl)prop-2-yn-1-amine **1p** (120 mg, 0.400 mmol, 1.00 equiv.). The crude material was purified by flash column chromatography (pentane/EtOAc gradient 100:0 to 100:3) to give the corresponding olefin **3p** (137 mg, 0.344 mmol, 86% yield) as a dark red liquid. The enantiomeric excess was determined to be 86% by HPLC analysis on a Daicel Chiralpak IB N-5 column: 99:1 hexane/IPA, flow rate 1 mL/min, λ = 254 nm: τ_{Minor} = 26.0 min τ_{Major} = 13.8 min. Absolute configuration was determined by X-Ray diffraction analysis of a single crystal of **3a**.

R$_f$ value: 0.62 (5% Ethyl acetate in Pentane).

[α]D^{20} = +3.9 (c = 0.46, CHCl$_3$, 86% ee).

^{1}H NMR (400 MHz, Chloroform-*d*) δ 7.70 (t, *J* = 1.8 Hz, 1H, Ar*H*), 7.46 (dt, *J* = 7.8, 1.4 Hz, 1H, Ar*H*), 7.41 – 7.36 (m, 4H, Ar*H*), 7.36 – 7.27 (m, 2H, Ar*H*), 7.18 (t, *J* = 7.9 Hz, 1H, Ar*H*), 5.28 (s, 1H, vinyl C*H*), 5.20 (q, *J* = 5.3 Hz, 1H, C*H*CF$_3$), 4.07 (d, *J* = 15.7, 1H, NCH_aH$_b$C=C), 3.98 (d, *J* = 13.1 Hz, 1H, PhCH_aH$_b$), 3.90 (d, *J* = 13.1 Hz, 1H, PhCH$_a$$H_b$), 3.62 (d, *J* = 15.7, 1H, NCH$_a$$H_b$C=C).

^{13}C{^{1}H} NMR (101 MHz, Chloroform-*d*) δ 151.5, 137.5, 136.8, 130.6, 130.0, 128.8 (3C), 128.2, 126.3, 122.6, 122.6 (q, *J* = 283.5 Hz), 97.6, 95.2 (q, *J* = 34.6 Hz), 60.5, 55.5.

^{19}F{^{1}H} NMR (376 MHz, Chloroform-*d*) δ -80.3 (s, 3F, CHC*F*$_3$).

IR (cm^{-1}) 2927 (m), 2858 (w), 1689 (m), 1589 (m), 1466 (m), 1296 (m), 1176 (s), 1146 (s), 972 (m), 694 (m).

HRMS (ESI/QTOF) m/z: [M + H]$^+$ Calcd for $C_{18}H_{16}BrF_3NO^+$ 398.0362; Found 398.0354.

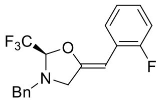

3q

(R,Z)-3-Benzyl-5-(2-fluorobenzylidene)-2-(trifluoromethyl)oxazolidine (3q)

Prepared according to the general procedure D1 using N-benzyl-3-(2-fluorophenyl)prop-2-yn-1-amine **1q** (96 mg, 0.40 mmol, 1.0 equiv.). The crude material was purified by flash column chromatography (pentane/EtOAc gradient 100:0 to 97:3) to give the corresponding olefin **3q** (60 mg, 0.18 mmol, 45% yield) as pale yellow oil. The enantiomeric excess was determined to be 84% by HPLC analysis on a Daicel Chiralpak IB N-5 column: 99:1 hexane/IPA, flow rate 1 mL/min, λ = 254 nm: τ_{Minor} = 17.3 min, τ_{Major} = 10.7 min. Absolute configuration determined in comparison to compound **3a.**

R_f value: 0.58 (5% Ethyl acetate in Pentane).

$[\alpha]D^{20}$ = 16.5 (c = 0.22, $CHCl_3$, 84% ee).

<u>^{1}H NMR</u> (400 MHz, Chloroform-d) δ 8.08 – 7.92 (m, 1H, Ar*H*), 7.46 – 7.28 (m, 5H, Ar*H*), 7.17 – 7.08 (m, 2H, Ar*H*), 7.06 – 6.96 (m, 1H, Ar*H*), 5.62 (s, 1H, vinyl C*H*), 5.18 (q, *J* = 5.3 Hz, 1H, C*H*CF_3), 4.09 (d, *J* = 15.7 Hz, 1H, NCH_aH_bC=C), 3.99 (d, *J* = 13.1 Hz, 1H, PhCH_aH), 3.92 (d, *J* = 13.1 Hz, 1H, PhCH_aH_b), 3.66 (d, *J* = 15.7 Hz, 1H, NCH_aH_bC=C).

<u>^{13}C{^{1}H}</u> NMR (101 MHz, Chloroform-d) δ 159.1 (d, *J* = 247.4 Hz), 151.64 (d, *J* = 2.2 Hz), 136.9, 129.4 (d, *J* = 3.1 Hz), 128.9, 128.8, 128.2, 127.2 (d, *J* = 8.4 Hz), 124.1 (d, *J* = 3.6 Hz), 123.2 (d, *J* = 12.0 Hz), 122.6 (q, *J* = 283.5 Hz), 115.0 (d, *J* = 22.1 Hz), 95.1 (q, *J* = 34.6 Hz), 89.9 (d, *J* = 8.1 Hz), 60.5, 55.7.

<u>^{19}F{^{1}H} NMR</u> (376 MHz, Chloroform-*d*) δ -80.3 (s, 3F, CHCF_3), -119.1 (s, 1F, Ar*F*)

<u>IR</u> (cm^{-1}) 1697 (m), 1658 (m), 1489 (m), 1454 (m), 1292 (m), 1176 (s), 1149 (s).

<u>HRMS</u> (ESI/QTOF) m/z: $[M + H]^+$ Calculated for $C_{18}H_{16}F_4NO^+$ 338.1163; Found 338.1170.

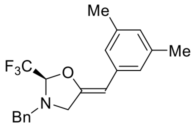

3r

(R,Z)-3-Benzyl-5-(3,5-dimethylbenzylidene)-2-(trifluoromethyl)oxazolidine (3r)

Prepared according to the general procedure D1 using N-benzyl-3-(3,5-dimethylphenyl)prop-2-yn-1-amine **1r** (100 mg, 0.400 mmol, 1.00 equiv.). The crude material was purified by flash column chromatography (pentane/EtOAc gradient 100:0 to 97:3) to give the corresponding olefin **3r** (107 mg, 0.307 mmol, 77% yield) as colorless oil. The enantiomeric excess was determined to be 86% by HPLC analysis on a Daicel Chiralpak IB N-5 column: 99:1 hexane/IPA, flow rate 1 mL/min, λ = 254 nm: τ_{Minor} = 12.8 min, τ_{Major} = 8.5 min. Absolute configuration determined in comparison to compound **3a.**

R_f value: 0.60 (5% Ethyl acetate in Pentane).

$[\alpha]D^{20}$ = 24.1 (c = 0.53, $CHCl_3$, 86% ee).

<u>^{1}H NMR</u> (400 MHz, Chloroform-d) δ 7.41 – 7.30 (m, 5H, Ar*H*), 7.18 (d, *J* = 1.5 Hz, 2H, Ar*H*), 6.83 (s, 1H, Ar*H*), 5.29 (s, 1H, vinyl C*H*), 5.17 (q, *J* = 5.4 Hz, 1H, C*H*CF_3), 4.1 (d, *J* = 15.4 Hz, 1H, NCH_aH_bC=C), 3.98 (d, *J* = 13.1 Hz, 1H, PhCH_aH), 3.90 (d, *J* = 13.1 Hz, 1H, PhCH_aH_b), 3.59 (d, *J* = 15.4 Hz, 1H, NCH_aH_bC=C), 2.32 (s, 6H, 2×ArCH_3).

<u>^{13}C{^{1}H}</u> NMR (101 MHz, Chloroform-d) δ 149.9, 137.9, 137.1, 135.2, 128.86, 128.79, 128.1, 127.8, 125.7, 122.71 (q, *J* = 283.6 Hz), 98.9, 94.87 (q, *J* = 34.5 Hz), 60.5, 55.5, 21.6.

<u>^{19}F{^{1}H} NMR</u> (376 MHz, Chloroform-*d*) δ -80.3.

<u>IR</u> (cm^{-1}) 3024 (w), 2924 (w), 2862 (w), 1689 (m), 1601 (w), 1458 (w), 1369 (m), 1300 (m), 1173 (s), 1146 (s).

<u>HRMS</u> (ESI/QTOF) m/z: $[M + H]^+$ Calculated for $C_{20}H_{21}F_3NO^+$ 348.1570; Found 348.1572.

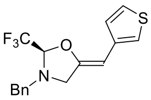

3s

(R,Z)-3-Benzyl-5-(thiophen-3-ylmethylene)-2-(trifluoromethyl)oxazolidine (3s)

Prepared according to the general procedure D1 using N-benzyl-3-(thiophen-3-yl)prop-2-yn-1-amine **1s** (91 mg, 0.40 mmol, 1.0 equiv.). The crude material was purified by flash column chromatography (pentane/EtOAc gradient 100:0 to 100:3) to give the corresponding olefin **3s** (112 mg, 0.344 mmol, 86% yield) as a black oil. The enantiomeric excess was determined to be 86% by HPLC analysis on a Daicel Chiralpak IB N-5 column: 99:1 hexane/IPA, flow rate 1 mL/min, λ = 254 nm: τ_{Minor} = 24.6 min τ_{Major} = 12.5 min. Absolute configuration was determined by X-Ray diffraction analysis of a single crystal of **3a**.

R_f value: 0.56 (5% Ethyl acetate in Pentane).

$[\alpha]D^{20}$ = +25.3 (c = 0.48, $CHCl_3$, 86% ee).

^{1}H NMR (400 MHz, Chloroform-*d*) δ 7.42 – 7.36 (m, 4H, Ar*H*), 7.36 – 7.29 (m, 2H, Ar*H*), 7.29 – 7.24 (m, 2H, Ar*H*), 5.47 (s, 1H, vinyl C*H*), 5.14 (q, *J* = 5.3 Hz, 1H, C*H*CF$_3$), 4.03 (d, *J* = 15.5, 1H, NC*H$_a$*H$_b$C=C), 3.97 (d, *J* = 13.1 Hz, 1H, PhC*H$_a$*H$_b$), 3.90 (d, *J* = 13.1 Hz, 1H, PhCH$_a$*H$_b$*), 3.57 (d, *J* = 15.5, 1H, NCH$_a$*H$_b$*C=C).

^{13}C{^{1}H} NMR (101 MHz, Chloroform-*d*) δ 149.5, 137.0, 136.0, 128.8, 128.8, 128.1, 128.0, 125.0, 122.7 (q, *J* = 283.4 Hz), 121.0, 94.6 (q, *J* = 34.4 Hz), 93.7, 60.4, 54.8.
^{19}F{^{1}H} NMR (376 MHz, Chloroform-*d*) δ -80.4 (s, 3F, CHC*F*$_3$).

IR (cm^{-1}) 3035 (w), 2939 (w), 2846 (w), 1693 (m), 1296 (m), 1173 (s), 1142 (s), 976 (m), 768 (m), 702 (m).

HRMS (ESI/QTOF) m/z: [M + H]$^+$ Calcd for $C_{16}H_{15}F_3NOS^+$ 326.0821; Found 326.0820.

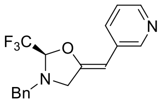

3t

(R,Z)-3-Benzyl-5-(pyridin-3-ylmethylene)-2-(trifluoromethyl)oxazolidine (3t)

Prepared according to the general procedure D1 using N-benzyl-3-(pyridin-3-yl)prop-2-yn-1-amine **1t** (89 mg, 0.40 mmol, 1.0 equiv.). The crude material was purified by flash column chromatography (pentane/EtOAc gradient 100:0 to 100:3) to give the corresponding olefin **3t** (52 mg, 0.16 mmol, 41% yield) as an orange liquid. The enantiomeric excess was determined to be 80% by HPLC analysis on a Daicel Chiralpak IB N-5 column: 80:20 hexane/IPA, flow rate 1 mL/min, λ = 254 nm: τ_{Minor} = 23.3 min τ_{Major} = 17.9 min. Absolute configuration was determined by X-Ray diffraction analysis of a single crystal of **3a**.

R$_f$ value: 0.12 (40% Ethyl acetate in Pentane).

[α]D^{20} = -0.06 (c = 0.46, CHCl$_3$, 80% ee).

^{1}H NMR (400 MHz, Chloroform-*d*) δ 8.63 (dd, *J* = 2.4, 0.8 Hz, 1H, (Hetero)Ar*H*), 8.38 (dd, *J* = 4.8, 1.6 Hz, 1H, (Hetero)Ar*H*), 7.97 (dt, *J* = 8.0, 2.0 Hz, 1H, (Hetero)Ar*H*), 7.43 – 7.28 (m, 5H, Ar*H*), 7.24 (ddd, *J* = 8.0, 4.8, 0.9 Hz, 1H, (Hetero)Ar*H*), 5.33 (s, 1H, vinyl C*H*), 5.20 (q, *J* = 5.3 Hz, 1H, C*H*CF$_3$), 4.13 – 4.03 (d, *J* = 15.7, 1H, NC*H$_a$*H$_b$C=C), 4.00 (d, *J* = 13.1 Hz, 1H, PhC*H$_a$*H$_b$), 3.91 (d, *J* = 13.1 Hz, 1H, PhCH$_a$*H$_b$*), 3.65 (d, *J* = 15.7, 1H, NCH$_a$*H$_b$*C=C).

^{13}C{^{1}H} NMR (101 MHz, Chloroform-*d*) δ 152.4, 149.1, 146.9, 136.8, 134.4, 131.4, 128.9, 128.9, 128.2, 123.4, 122.6 (q, *J* = 283.6 Hz), 95.3, 95.2 (q, *J* = 34.7 Hz), 60.5, 55.5.
^{19}F{^{1}H} NMR (376 MHz, Chloroform-*d*) δ -80.4 (s, 3F, CHC*F*$_3$).
IR (cm^{-1}) 2931 (m), 2858 (w), 2110 (w), 1693 (m), 1377 (m), 1292 (m), 1176 (s), 1149 (s), 972 (m), 706 (m).

HRMS (ESI/QTOF) m/z: [M + H]$^+$ Calcd for $C_{17}H_{16}F_3N_2O^+$ 321.1209; Found 321.1208.

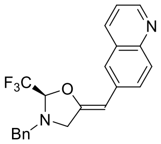

3u

(R,Z)-3-benzyl-5-(quinolin-6-ylmethylene)-2-(trifluoromethyl)oxazolidine (3u)

Prepared according to the general procedure D1 using N-benzyl-3-(quinolin-6-yl)prop-2-yn-1-amine **1u** (109 mg, 0.400 mmol, 1.00 equiv.). The crude material was purified by flash column chromatography (pentane/EtOAc gradient 100:0 to 100:3) to give the corresponding olefin **3u** (123 mg, 0.332 mmol, 83% yield) as an orange liquid. The enantiomeric excess was determined to be 90% by HPLC analysis on a Daicel Chiralpak IB N-5 column: 90:10 hexane/IPA, flow rate 1 mL/min, λ = 254 nm: τ_{Minor} = 26.8 min τ_{Major} = 20.4 min. Absolute configuration was determined by X-Ray diffraction analysis of a single crystal of **3a**.

R$_f$ value: 0.12 (40% Ethyl acetate in Pentane).

[α]D^{20} = +27.4 (c = 0.75, CHCl$_3$, 90% ee).

^{1}H NMR (400 MHz, Chloroform-*d*) δ 8.84 (dd, *J* = 4.3, 1.7 Hz, 1H, (Hetero)Ar*H*), 8.11 (ddd, *J* = 8.2, 1.8, 0.7 Hz, 1H, (Hetero)Ar*H*), 8.07 – 8.00 (m, 1H, (Hetero)Ar*H*), 7.98 – 7.88 (m, 2H, (Hetero)Ar*H*), 7.44 – 7.28 (m, 6H, Ar*H*), 5.52 (s, 1H, vinyl C*H*), 5.25 (q, *J* = 5.3 Hz, 1H, C*H*CF$_3$), 4.13 (d, *J* = 15.6, 1H, NC*H$_a$*H$_b$C=C), 4.02 (d, *J* = 13.1 Hz, 1H, PhC*H$_a$*H$_b$), 3.94 (d, *J* = 13.1 Hz, 1H, PhCH$_a$*H$_b$*), 3.69 (d, *J* = 15.6, 1H, NCH$_a$*H$_b$*C=C).

^{13}C{^{1}H} NMR (101 MHz, Chloroform-*d*) δ 151.6, 149.9, 147.0, 136.9, 136.0, 133.8, 130.3, 129.4, 128.9 (2C), 128.6, 128.2, 125.7, 122.6 (q, *J* = 283.3 Hz), 121.3, 98.3, 95.2 (q, *J* = 34.6 Hz), 60.5, 55.7.
^{19}F{^{1}H} NMR (376 MHz, Chloroform-*d*) δ -80.2 (s, 3F, CHC*F*$_3$).

IR (cm^{-1}) 3032 (w), 2931 (w), 2850 (w), 1685 (m), 1500 (w), 1296 (m), 1176 (s), 1146 (s), 972 (m), 756 (m).

HRMS (ESI/QTOF) m/z: [M + H]$^+$ Calcd for $C_{21}H_{18}F_3N_2O^+$ 371.1366; Found 371.1364.

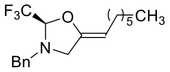

3v

(R,Z)-3-Benzyl-2-(trifluoromethyl)-5-(3,3,3-trimethyl-3l8-butylidene)oxazolidine (3v)

Prepared according to the general procedure D1 using N-benzylnon-2-yn-1-amine **1v** (92 mg, 0.40 mmol, 1.0 equiv.). The crude material was purified by flash column

chromatography (pentane/EtOAc gradient 100:0 to 100:3) to give the corresponding olefin **3v** (20 mg, 0.061 mmol, 15% yield) as a colorless oil. The enantiomeric excess was determined to be 56% by HPLC analysis on a Daicel Chiralpak IB N-5 column: 99:1 hexane/IPA, flow rate 1 mL/min, λ = 214 nm: τ_{Minor} = 5.2 min τ_{Major} = 4.5 min. Absolute configuration was determined by X-Ray diffraction analysis of a single crystal of **3a**.

R_f value: 0.45 (5% Ethyl acetate in Pentane).

$[\alpha]D^{20}$ = +0.45 (c = 0.50, $CHCl_3$, 54% ee).

^{1}H NMR (400 MHz, Chloroform-*d*) δ 7.40 – 7.27 (m, 5H, Ar*H*), 4.91 (q, *J* = 5.3 Hz, 1H, C*H*CF_3), 4.31 (tt, *J* = 7.4, 1.6 Hz, 1H, NCH_2(O)C=C*H*(CH_2)(CH_2)$_4$$CH_3$), 3.95 (d, *J* = 13.1 Hz, 1H, PhCH_aH_b), 3.83 (d, *J* = 13.1 Hz, 1H, PhCH$_a$$H_b$), 3.78 (d, *J* = 14.6 Hz, 1H, NCH_aH$_b$C=C), 3.39 – 3.29 (d, 14.6 Hz, 1H, NCH$_a$$H_b$C=C), 2.18 – 2.05 (m, 2H, C=CH(CH_2)(CH_2)$_4$$CH_3$), 1.39 – 1.22 (m, 8H, C=CH(CH_2)(CH_2)$_4$$CH_3$), 0.94 – 0.79 (m, 3H, C=CH(CH_2)(CH_2)$_4$CH_3).

^{13}C{^{1}H} NMR (101 MHz, Chloroform-*d*) δ 148.9, 137.4, 128.8, 128.7, 127.9, 122.9 (q, *J* = 283.6 Hz), 98.3, 93.1 (q, *J* = 33.9 Hz), 60.3, 53.5, 31.8, 29.9, 28.9, 25.4, 22.8, 14.2.

^{19}F{^{1}H} NMR (376 MHz, Chloroform-*d*) δ -80.6 (s, 3F, CHCF_3).

IR (cm^{-1}) 3321 (w), 2927 (s), 2858 (s), 1454 (m), 1331 (m), 1111 (m), 741 (s), 702 (m).

HRMS (ESI/QTOF) m/z: $[M + H]^+$ Calcd for $C_{18}H_{25}F_3NO^+$ 328.1883; Found 328.1879.

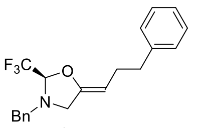
3w

(R,Z)-3-Benzyl-5-(3-phenylpropylidene)-2-(trifluoromethyl)oxazolidine (3w)

Prepared according to the general procedure D1 using N-benzyl-5-phenylpent-2-yn-1-amine **1w** (100 mg, 0.400 mmol, 1.00 equiv.). The crude material was purified by flash column chromatography (pentane/EtOAc gradient 100:0 to 100:3) to give the corresponding olefin **3w** (50 mg, 0.14 mmol, 36% yield) as a colorless oil. The enantiomeric excess was determined to be 74% by HPLC analysis on a Daicel Chiralpak IB N-5 column: 99:1 hexane/IPA, flow rate 1 mL/min, λ = 214 nm: τ_{Minor} = 8.6 min τ_{Major} = 7.6 min. Absolute configuration was determined by X-Ray diffraction analysis of a single crystal of **3a**.

R_f value: 0.48 (5% Ethyl acetate in Pentane).

$[\alpha]D^{20}$ = +3.9 (c = 0.49, $CHCl_3$, 72% ee).

^{1}H NMR (400 MHz, Chloroform-*d*) δ 7.35 (d, *J* = 4.4 Hz, 4H, Ar*H*), 7.33 – 7.24 (m, 3H), Ar*H*, 7.24 – 7.15 (m, 3H, Ar*H*), 4.89 (q, *J* = 5.3 Hz, 1H, C*H*CF_3), 4.34 (tt, *J* = 7.2, 1.5 Hz, 1H, C=C*H*(CH_2)(CH_2)Ph), 3.89 (d, *J* = 13.1 Hz, 1H, PhCH_aH$_b$), 3.81 – 3.72 (m, 2H, PhCH$_a$$H_b$, NCH_aH$_b$C=C), 3.32 (ddd, *J* = 14.7, 2.6, 1.3 Hz, 1H, NCH$_a$$H_b$C=C), 2.79 – 2.61 (m, 2H, C=CH(CH_2)(CH_2)Ph), 2.51 – 2.41 (m, 2H, C=CH(CH_2)(CH_2)Ph).

^{13}C{^{1}H} NMR (101 MHz, Chloroform-*d*) δ 149.5, 142.2, 137.4, 128.8, 128.7, 128.6, 128.3, 127.9, 125.9, 122.9 (q, *J* = 283.7 Hz), 97.1, 93.2 (q, *J* = 34.1 Hz), 60.2, 53.5, 36.0, 27.0.

^{19}F{^{1}H} NMR (376 MHz, Chloroform-*d*) δ -80.6 (s, 3F, CHCF_3).

IR (cm^{-1}) 3032 (w), 2927 (w), 2854 (w), 1712 (w), 1296 (m), 1169 (s), 1146 (s), 1030 (m), 980 (m), 744 (m), 702 (m).

HRMS (ESI/QTOF) m/z: $[M + H]^+$ Calcd for $C_{20}H_{21}F_3NO^+$ 348.1570; Found 348.1573.

D.3. General Procedure for the Asymmetric Hydrogenation of the Trisubstituted Olefins.

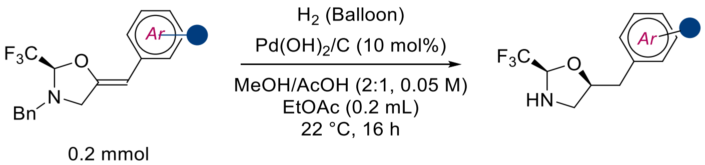

3 4

Scheme 8. Palladium-catalyzed asymmetric hydrogenation of olefins.

An oven-dried 25 mL round-bottom flask equipped with a Teflon coated stirring bar was charged with $Pd(OH)_2/C$ (20% Pd on C) (14 mg, 0.020 mmol, 10 mol%) and the olefin **3**. The flask was sealed and evacuated and back-filled with N_2 three times. MeOH (2.7 mL), AcOH (1.3 mL) and EtOAc (0.2 mL) were added and the suspension was stirred at room temperature for 10 minutes under a nitrogen flow. Then, a hydrogen balloon was connected to the flask through a needle and the mixture was vigorously stirred at room

temperature for 16 hours. Then, the reaction mixture was degassed by bubbling nitrogen for 10 minutes and filtered through a plug of celite eluting with 10 mL of MeOH. The crude extract was washed with saturated $NaHCO_3$ and extracted with DCM (3 x 25 mL). The combined organic layer was dried over sodium sulfate, filtered and concentrated in vacuum. The crude material was purified by flash column chromatography on silica gel to afford the corresponding product **4** as a single diastereoisomer.

D.4. Characterization of Hydrogenated Products

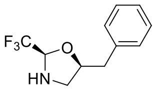

(R,S)-**4a**

(2R,5S)-5-Benzyl-2-(trifluoromethyl)oxazolidine ((*R,S*)-4a)

Prepared according to the general procedure D3 using **3a** (64 mg, 0.20 mmol, 1.0 equiv., 90% ee). The crude material was purified by flash column chromatography (pentane/EtOAc gradient 100:0 to 80:20) to give the corresponding product *(R,S)*-**4a** (39 mg, 0.17 mmol, 85% yield) as a colorless oil. The enantiomeric excess was determined to be 90% by HPLC analysis on a Daicel Chiralpak IA column: 95:5 hexane/IPA, flow rate 1 mL/min, λ = 210 nm: τ_{Major} = 10.8 min, τ_{Minor} = 8.5 min. Absolute and relative configuration were determined by X-Ray diffraction analysis of a single crystal of *(R,S)*-**4b** (Details in section F).

R_f value: 0.31 (20% Ethyl acetate in Pentane).

$[\alpha]D^{20}$ = 16.5 (c = 0.64, $CHCl_3$, 90% ee).

^{1}H NMR (400 MHz, Chloroform-*d*) δ 7.34 – 7.28 (m, 2H, Ar*H*), 7.27 – 7.21 (m, 3H, Ar*H*), 4.93 (dq, *J* = 8.6, 5.5 Hz, 1H, C*H*CF_3), 4.11 (dq, *J* = 9.1, 6.3 Hz, 1H, OC*H*), 3.27 (dddd, *J* = 11.7, 7.3, 5.6, 1.4 Hz, 1H, NCH_aH_b), 3.08 (dd, *J* = 13.7, 6.8 Hz, 1H, ArCH_aH_b), 2.89 – 2.75 (m, 2H, ArCH_aH_b + NCH_aH_b), 2.63 (q, *J* = 10.1, 9.3 Hz, 1H, N*H*).

^{13}C{^{1}H} NMR (101 MHz, Chloroform-*d*) δ 137.7, 129.2, 128.7, 126.8, 123.5 (q, *J* = 282.6 Hz), 88.0 (q, *J* = 33.9 Hz), 80.7, 50.8, 39.9.

^{19}F{^{1}H} NMR (376 MHz, Chloroform-*d*) δ -81.4.

IR (cm^{-1}) 3352 (w), 3032 (w), 2939 (w), 1709 (w), 1608 (w), 1496 (w), 1454 (w), 1292 (m), 1161 (s).

HRMS (ESI/QTOF) m/z: $[M + H]^+$ Calculated for $C_{11}H_{13}F_3NO^+$ 232.0944; Found 232.0939.

1.0 mmol scale reduction. The model reduction was repeated on 1.0 mmol scale. An oven dried 50 mL round-bottom flask equipped with a Teflon stirring bar was charged with $Pd(OH)_2$ (5.0 wt%, 70 mg, 0.10 mmol, 10 mol%) and olefin **3a** (319 mg, 1.00 mmol, 1.00 equiv.). MeOH (13 mL), AcOH (7 mL) and EtOAc (1 mL) were added and the suspension was stirred at ambient temperature for 10 minutes under a nitrogen flow. Then, a hydrogen balloon was connected to the flask through a needle and the mixture was vigorously stirred at ambient temperature for 16 hours. Then, the reaction mixture was degassed by bubbling nitrogen for 10 minutes and filtered through a plug of celite eluting with 20 mL of MeOH. The crude extract was washed with saturated $NaHCO_3$ and extracted with DCM (3×50 mL). The combined organic layer was dried over sodium sulfate, filtered and concentrated *in vacuo*. The crude material was purified by flash column chromatography (pentane/EtOAc gradient 100:0 to 80:20) to give the corresponding product *(R,S)*-**4a** (284 mg, 0.884 mmol, 72% yield) as a colorless oil, which solidified upon vigorous scratching with a spatula. The enantiomeric excess was determined to be 90% by HPLC analysis on a Daicel Chiralpak IA column: 95:5 hexane/IPA, flow rate 1 mL/min, λ = 210 nm: τ_{Major} = 10.8 min, τ_{Minor} = 8.5 min.

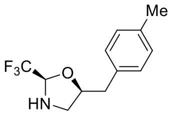

(R,S)-**4b**

(2R,5S)-5-(4-Methylbenzyl)-2-(trifluoromethyl)oxazolidine ((*R,S*)-4b)

Prepared according to the general procedure D3 using **3b** (67 mg, 0.20 mmol, 1.0 equiv., 90% ee). The crude material was purified by flash column chromatography (pentane/EtOAc gradient 100:0 to 80:20) to give the corresponding product *(R,S)*-**4b** (38 mg, 0.15 mmol, 77% yield) as a white amorphous solid. The enantiomeric excess was determined to be 90% by HPLC analysis on a Daicel Chiralpak IA column: 95:5 hexane/IPA, flow rate 1 mL/min, λ = 214 nm: τ_{Major} = 10.1 min, τ_{Minor} = 7.5 min. Absolute and relative configuration were determined by X-Ray diffraction analysis of a single crystal of *(R,S)*-**4b** (Details in section F).

R_f value: 0.34 (20% Ethyl acetate in Pentane).

$[\alpha]D^{20}$ = +14.8 (c = 0.48, $CHCl_3$, 90% ee).

^{1}H NMR (400 MHz, Chloroform-*d*) δ 7.11 (s, 4H, Ar*H*), 4.93 (q, *J* = 5.5 Hz, 1H, C*H*CF$_3$), 4.08 (dq, *J* = 9.0, 6.4 Hz, 1H, OC*H*), 3.26 (dd, *J* = 11.4, 5.5 Hz, 1H, NCH_aH$_b$), 3.04 (dd, *J* = 13.7, 6.8 Hz, 1H, ArCH_aH$_b$), 2.86 – 2.71 (m, 2H, ArCH$_a$$H_b$ + NCH$_a$$H_b$), 2.64 (s, 1H, N*H*), 2.33 (s, 3H, ArCH_3).

^{13}C{^{1}H} NMR (101 MHz, Chloroform-*d*) δ 136.4, 134.6, 129.4, 129.1, 123.5 (q, *J* = 282.6 Hz), 88.0 (q, *J* = 33.9 Hz), 80.9, 50.8, 39.5, 21.2.

^{19}F{^{1}H} NMR (376 MHz, Chloroform-*d*) δ -81.4 (s, 3F, CHCF_3).

IR (cm^{-1}) 3352 (w), 2931 (w), 1516 (w), 1450 (w), 1292 (m), 1161 (s), 1103 (m).

HRMS (ESI/QTOF) m/z: [M + H]$^+$ Calcd for $C_{12}H_{15}F_3NO^+$ 246.1100; Found 246.1103.

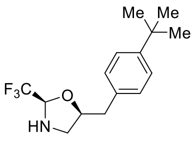

(*R*,*S*)-**4c**

(2R,5S)-5-(4-(Tert-butyl)benzyl)-2-(trifluoromethyl)oxazolidine ((*R*,*S*)-4c)

Prepared according to the general procedure D3 using **3c** (75 mg, 0.20 mmol, 1.0 equiv., 90% ee). The crude material was purified by flash column chromatography (pentane/EtOAc gradient 100:0 to 80:20) to give the corresponding product *(R,S)*-**4c** (46 mg, 0.16 mmol, 80% yield) as a white amorphous solid. The enantiomeric excess was determined to be 84% by HPLC analysis on a Daicel Chiralpak IA column: 95:5 hexane/IPA, flow rate 1 mL/min, λ = 214 nm: τ_{Major} = 7.9 min, τ_{Minor} = 6.7 min. Absolute and relative configuration were determined by X-Ray diffraction analysis of a single crystal of *(R,S)*-**4b**.

R$_f$ value: 0.36 (20% Ethyl acetate in Pentane).

[α]D^{20} = +10.5 (c = 0.51, CHCl$_3$, 84% ee).

^{1}H NMR (400 MHz, Chloroform-*d*) δ 7.37 – 7.29 (m, 2H, Ar*H*), 7.20 – 7.12 (m, 2H, Ar*H*), 4.94 (q, *J* = 5.7 Hz, 1H, C*H*CF$_3$), 4.10 (dq, *J* = 8.8, 6.3 Hz, 1H, OC*H*), 3.28 (dd, *J* = 12.0, 5.4 Hz, 1H, NCH_aH$_b$), 3.05 (dd, *J* = 13.8, 6.8 Hz, 1H, ArCH_aH$_b$), 2.84 (d, *J* = 10.4 Hz, 1H, NCH$_a$$H_b$), 2.76 (dd, *J* = 13.8, 6.5 Hz, 1H, ArCH$_a$$H_b$), 2.64 (s, 1H, N*H*), 1.31 (s, 9H, C(CH_3)$_3$).

^{13}C{^{1}H} NMR (101 MHz, Chloroform-*d*) δ 149.6, 134.6, 128.8, 125.6, 123.5 (q, *J* = 282.7 Hz), 88.0 (q, *J* = 33.9 Hz), 80.9, 50.9, 39.4, 34.6, 31.5.

^{19}F{^{1}H} NMR (376 MHz, Chloroform-*d*) δ -81.4 (s, 3F, CHCF_3).

IR (cm^{-1}) 3352 (w), 2962 (m), 2904 (w), 1516 (w), 1462 (w), 1288 (m), 1165 (s), 1103 (m).

HRMS (ESI/QTOF) m/z: [M + H]$^+$ Calcd for $C_{15}H_{21}F_3NO^+$ 288.1570; Found 288.1566.

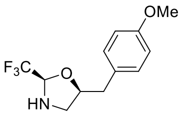

(*R*,*S*)-**4d**

(2R,5S)-5-(4-Methoxybenzyl)-2-(trifluoromethyl)oxazolidine ((*R*,*S*)-4d)

Prepared according to the general procedure D3 using **3d** (70 mg, 0.20 mmol, 1.0 equiv., 84% ee). The crude material was purified by flash column chromatography (pentane/EtOAc gradient 100:0 to 80:20) to give the corresponding product *(R,S)*-**4d** (31 mg, 0.12 mmol, 59% yield) as colorless oil The enantiomeric excess was determined to be 84% by HPLC analysis on a Daicel Chiralpak IA column: 95:5 hexane/IPA, flow rate 1 mL/min, λ = 210 nm: τ_{Major} = 14.7 min, τ_{Minor} = 10.6 min. Absolute configuration was determined in comparison to compound *(R,S)*-**4b**.

R$_f$ value: 0.26 (20% Ethyl acetate in Pentane).

[α]D^{20} = 10.4 (c = 0.53, CHCl$_3$, 84% ee).

^{1}H NMR (400 MHz, Chloroform-*d*) δ 7.14 (d, *J* = 8.6 Hz, 2H, Ar*H*), 6.85 (d, *J* = 8.6 Hz, 2H, Ar*H*), 4.93 (dq, *J* = 8.4, 5.5 Hz, 1H, C*H*CF$_3$), 4.06 (dq, *J* = 9.2, 6.3 Hz, 1H, OC*H*), 3.79 (s, 3H, OCH_3), 3.25 (dt, *J* = 11.7, 6.0 Hz, 1H, NCH_aH$_b$), 3.01 (dd, *J* = 13.8, 6.8 Hz, 1H, ArCH_aH$_b$), 2.87 – 2.69 (m, 2H, ArCH$_a$$H_b$ + NCH$_a$$H_b$), 2.63 (t, *J* = 9.3 Hz, 1H, N*H*).

^{13}C{^{1}H} NMR (101 MHz, Chloroform-*d*) δ 158.5, 130.2, 129.7, 123.5 (q, *J* = 282.6 Hz), 114.1, 88.0 (q, *J* = 33.9 Hz), 81.0, 55.4, 50.7, 39.0.

^{19}F{^{1}H} NMR (376 MHz, Chloroform-*d*) δ -81.4.

IR (cm^{-1}) 3352 (w), 2943 (w), 2843 (w), 1701 (w), 1612 (w), 1516 (m), 1458 (w), 1292 (m), 1250 (s), 1165 (s).

HRMS (ESI/QTOF) m/z: [M + H]$^+$ Calculated for $C_{12}H_{15}F_3NO_2^+$ 262.1049; Found 262.1053.

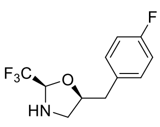

(*R,S*)-**4e**

(2R,5S)-5-(4-Fluorobenzyl)-2-(trifluoromethyl)oxazolidine ((*R,S*)-4e)

Prepared according to the general procedure D3 using **3e** (68 mg, 0.20 mmol, 1.0 equiv., 90% ee). The crude material was purified by flash column chromatography (pentane/EtOAc gradient 100:0 to 80:20) to give the corresponding product *(R,S)*-**4e** (42 mg, 0.17 mmol, 84% yield) as a white amorphous solid. The enantiomeric excess was determined to be 92% by HPLC analysis on a Daicel Chiralpak IA column: 95:5 hexane/IPA, flow rate 1 mL/min, λ = 254 nm: τ_{Major} = 11.1 min, τ_{Minor} = 9.6 min. Absolute and relative configuration were determined by X-Ray diffraction analysis of a single crystal of *(R,S)*-**4b**.

R_f value: 0.38 (20% Ethyl acetate in Pentane).

$[\alpha]D^{20}$ = +7.6 (c = 0.48, $CHCl_3$, 92% ee).

<u>^{1}H NMR</u> (400 MHz, Chloroform-*d*) δ 7.23 – 7.13 (m, 2H, Ar*H*), 7.04 – 6.94 (m, 2H, Ar*H*), 4.94 (q, *J* = 5.5 Hz, 1H, C*H*CF_3), 4.07 (dq, *J* = 8.7, 6.0 Hz, 1H, OC*H*), 3.28 (ddd, *J* = 11.8, 5.6, 1.5 Hz, 1H, NCH_aH_b), 3.01 (dd, *J* = 13.9, 7.1 Hz, 1H, ArCH_aH_b), 2.86 – 2.75 (m, 2H, ArCH_aH_b + NCH_aH_b), 2.73 – 2.48 (m, 1H, N*H*).

<u>^{13}C{^{1}H} NMR</u> (101 MHz, Chloroform-*d*) δ 162.0 (d, *J* = 244.6 Hz), 133.4 (d, *J* = 3.3 Hz), 130.7 (d, *J* = 7.9 Hz), 123.4 (q, *J* = 282.6 Hz), 115.5 (d, *J* = 21.3 Hz), 88.0 (q, *J* = 34.0 Hz), 80.6, 50.7, 39.0.
<u>^{19}F{^{1}H} NMR</u> (376 MHz, Chloroform-*d*) δ -81.4 (s, 3F, CHC*F*$_3$), -116.5 (s, 1F, Ar*F*).

<u>IR</u> (cm^{-1}) 3363 (w), 2931 (w), 1512 (m), 1292 (m), 1223 (m), 1161 (s), 1107 (m), 852 (m).

<u>HRMS</u> (ESI/QTOF) m/z: $[M + H]^+$ Calcd for $C_{11}H_{12}F_4NO^+$ 250.0850; Found 250.0858.

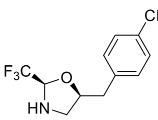

(*R,S*)-**4h**

(2R,5S)-2-(Trifluoromethyl)-5-(4-(trifluoromethyl)benzyl)oxazolidine ((*R,S*)-4h)

Prepared according to the general procedure D3 using **3h** (77 mg, 0.20 mmol, 1.0 equiv., 92% ee). The crude material was purified by flash column chromatography (pentane/EtOAc gradient 100:0 to 80:20) to give the corresponding product *(R,S)*-**4h** (51 mg, 0.17 mmol, 84% yield) as colorless oil The enantiomeric excess was determined to be 92% by HPLC analysis on a Daicel Chiralpak IA column: 95:5 hexane/IPA, flow rate 1 mL/min, λ = 210 nm: τ_{Major} = 6.8 min, τ_{Minor} = 7.3 min. Absolute configuration was determined in comparison to compound *(R,S)*-**4b**.

R_f value: 0.42 (20% Ethyl acetate in Pentane).

$[\alpha]D^{20}$ = 1.1 (c = 0.45, $CHCl_3$, 92% ee).

<u>^{1}H NMR</u> (400 MHz, Chloroform-*d*) δ 7.57 (d, *J* = 8.0 Hz, 2H, Ar*H*), 7.35 (d, *J* = 8.0 Hz, 2H, Ar*H*), 4.94 (dq, *J* = 8.6, 5.5 Hz, 1H, C*H*CF_3), 4.11 (ddd, *J* = 13.8, 8.4, 5.7 Hz, 1H, OC*H*), 3.33 (dddd, *J* = 11.7, 7.3, 5.7, 1.5 Hz, 1H, NCH_aH_b), 3.06 (dd, *J* = 13.9, 7.5 Hz, 1H, ArCH_aH_b), 2.98 – 2.75 (m, 2H, ArCH_aH_b + NCH_aH_b), 2.67 (q, *J* = 9.5, 9.0 Hz, 1H, N*H*).

<u>^{13}C{^{1}H} NMR</u> (101 MHz, Chloroform-*d*) δ 141.9, 129.6, 129.2 (q, *J* = 29.9), 125.6 (m), 124.4 (q, *J* = 271.6 Hz), 123.41 (q, *J* = 282.6 Hz), 88.1 (q, *J* = 34.0 Hz), 80.1, 50.7, 39.7.
<u>^{19}F{^{1}H} NMR</u> (376 MHz, Chloroform-*d*) δ -62.5 (s, 3F, ArC*F*$_3$), -81.4 (s, 3F, CHC*F*$_3$).

<u>IR</u> (cm^{-1}) 3348 (w), 2943 (w), 1705 (w), 1624 (w), 1423 (w), 1327 (s), 1292 (m), 1165 (s), 1126 (s), 1072 (m).

<u>HRMS</u> (ESI/QTOF) m/z: $[M + H]^+$ Calculated for $C_{12}H_{12}F_6NO^+$ 300.0818; Found 300.0816.

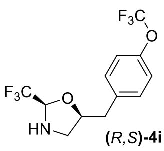

(*R,S*)-**4i**

(2R,5S)-5-(4-(Trifluoromethoxy)benzyl)-2-(trifluoromethyl)oxazolidine ((*R,S*)-4i)

Prepared according to the general procedure D3 using **3i** (81 mg, 0.20 mmol, 1.0 equiv., 90% ee). The crude material was purified by flash column chromatography (pentane/EtOAc gradient 100:0 to 80:20) to give the corresponding product *(R,S)*-**4i** (46 mg, 0.15 mmol, 73% yield) as a colorless liquid. The enantiomeric excess was determined to be 90% by HPLC analysis on a Daicel Chiralpak IB column: 95:5 hexane/IPA, flow rate 1 mL/min, λ = 254 nm: τ_{Major} = 13.1 min, τ_{Minor} = 10.3 min. Absolute and relative configuration were determined by X-Ray diffraction analysis of a single crystal of *(R,S)*-**4b**.

R_f value: 0.34 (20% Ethyl acetate in Pentane).

$[\alpha]D^{20}$ = -0.76 (c = 0.51, $CHCl_3$, 92% ee).

<u>^{1}H NMR</u> (400 MHz, Chloroform-*d*) δ 7.30 – 7.21 (m, 2H, Ar*H*), 7.20 – 7.11 (m, 2H, Ar*H*), 4.94 (s, 1H, C*H*CF_3), 4.15 – 4.03 (m, 1H, OC*H*), 3.31 (d, *J* = 11.0 Hz, 1H, NCH_aH_b), 3.02 (dd, *J* = 13.9, 7.3 Hz, 1H, ArCH_aH_b), 2.88 – 2.79 (m, 2H, ArCH_aH_b + NCH_aH_b), 2.66 (s, 1H, N*H*).

$^{13}C\{^1H\}$ NMR (101 MHz, Chloroform-*d*) δ 148.2, 136.5, 130.6, 123.4 (q, *J* = 282.7 Hz), 121.2, 120.6 (q, *J* = 255.7 Hz), 88.1 (q, *J* = 34.0 Hz), 80.3, 50.7, 39.2.
$^{19}F\{^1H\}$ NMR (376 MHz, Chloroform-*d*) δ -57.9 (s, 3F, ArOC*F₃*), -81.4 (s, 3F, CHC*F₃*).

IR (cm^{-1}) 2931 (m), 3340 (w), 2862 (w), 1504 (w), 1454 (w), 1265 (s), 1169 (s).

HRMS (ESI/QTOF) m/z: $[M + H]^+$ Calcd for $C_{12}H_{12}F_6NO_2^+$ 316.0767; Found 316.0768.

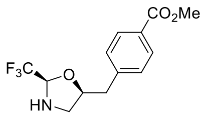

(*R,S*)-**4j**

Methyl 4-(((2R,5S)-2-(trifluoromethyl)oxazolidin-5-yl)methyl)benzoate ((*R,S*)-4j)

Prepared according to the general procedure D3 using **3j** (75 mg, 0.20 mmol, 1.0 equiv., 92% ee). The crude material was purified by flash column chromatography (pentane/EtOAc gradient 100:0 to 80:20) to give the corresponding product *(R,S)*-**4j** (48 mg, 0.16 mmol, 82% yield) as colorless oil. The enantiomeric excess was determined to be 92% by HPLC analysis on a Daicel Chiralpak IA column: 95:5 hexane/IPA, flow rate 1 mL/min, λ = 230 nm: τ_{Major} = 26.8 min, τ_{Minor} = 17.8 min. Absolute configuration was determined in comparison to compound *(R,S)*-**4b**.

R_f value: 0.35 (20% Ethyl acetate in Pentane).

$[\alpha]D^{20}$ = 6.5 (c = 0.44, $CHCl_3$, 92% ee).

^1H NMR (400 MHz, Chloroform-*d*) δ 7.91 (d, *J* = 8.2 Hz, 2H, Ar*H*), 7.23 (d, *J* = 8.3 Hz, 2H, Ar*H*), 4.87 (dq, *J* = 8.6, 5.6 Hz, 1H, C*H*CF₃), 4.05 (dq, *J* = 8.8, 6.0 Hz, 1H, OC*H*), 3.84 (s, 3H, COOC*H₃*) 3.22 (dddd, *J* = 11.7, 7.3, 5.7, 1.5 Hz, 1H, NCH_aH$_b$), 3.01 (dd, *J* = 13.8, 7.2 Hz, 1H, ArCH_aH$_b$), 2.86 – 2.69 (m, 2H, NCH$_a$$H_b$ + ArCH$_a$$H_b$), 2.64 – 2.53 (m, 1H, N*H*).

$^{13}C\{^1H\}$ NMR (101 MHz, Chloroform-*d*) δ 167.1, 143.1, 130.0, 129.0, 128.8, 123.4 (q, *J* = 282.5 Hz), 88.1 (q, *J* = 33.9 Hz), 80.1, 52.2, 50.7, 39. 9.
$^{19}F\{^1H\}$ NMR (376 MHz, Chloroform-*d*) δ -81.4.

IR (cm^{-1}) 3352 (w), 2951 (w), 1716 (s), 1612 (w), 1442 (m), 1288 (s), 1165 (s), 1115 (s).
HRMS (ESI/QTOF) m/z: $[M + H]^+$ Calculated for $C_{13}H_{15}F_3NO_3^+$ 290.0999; Found 290.0998.

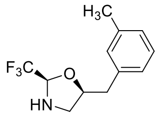

(*R,S*)-**4m**

(2R,5S)-5-(3-Methylbenzyl)-2-(trifluoromethyl)oxazolidine ((*R,S*)-4m)

Prepared according to the general procedure D3 using **3m** (67 mg, 0.20 mmol, 1.0 equiv., 90% ee). The crude material was purified by flash column chromatography (pentane/EtOAc gradient 100:0 to 80:20) to give the corresponding product *(R,S)*-**4m** (36 mg, 0.15 mmol, 73% yield) as a colorless liquid. The enantiomeric excess was determined to be 88% by HPLC analysis on a Daicel Chiralpak IA column: 95:5 hexane/IPA, flow rate 1 mL/min, λ = 214 nm: τ_{Major} = 10.1 min, τ_{Minor} = 7.5 min. Absolute and relative configuration were determined by X-Ray diffraction analysis of a single crystal of *(R,S)*-**4b**.

R_f value: 0.33 (20% Ethyl acetate in Pentane).

$[\alpha]D^{20}$ = +11.9 (c = 0.47, $CHCl_3$, 88% ee).

^1H NMR (400 MHz, Chloroform-*d*) δ 7.20 (td, *J* = 7.4, 1.0 Hz, 1H, Ar*H*), 7.08 – 6.98 (m, 3H, Ar*H*), 5.03 – 4.83 (m, 1H, C*H*CF₃), 4.10 (dq, *J* = 9.1, 6.3 Hz, 1H, OC*H*), 3.26 (dd, *J* = 12.1, 5.5 Hz, 1H, NCH_aH$_b$), 3.05 (dd, *J* = 13.7, 6.6 Hz, 1H, ArCH_aH$_b$), 2.87 – 2.70 (m, 2H, ArCH$_a$$H_b$ + NCH$_a$$H_b$), 2.63 (s, 1H, N*H*), 2.34 (s, 3H, C*H₃*).

$^{13}C\{^1H\}$ NMR (101 MHz, Chloroform-*d*) δ 138.3, 137.5, 130.0, 128.6, 127.5, 126.2, 123.5 (q, *J* = 282.6 Hz), 88.0 (q, *J* = 33.9 Hz), 80.8, 50.8, 39.8, 21.5.
$^{19}F\{^1H\}$ NMR (376 MHz, Chloroform-*d*) δ -81.37 (s, 3F, CHC*F₃*).

IR (cm^{-1}) 3348 (w), 3024 (w), 2935 (w), 1454 (m), 1288 (m), 1149 (s), 1099 (s), 787 (m).

HRMS (ESI/QTOF) m/z: $[M + H]^+$ Calcd for $C_{12}H_{15}F_3NO^+$ 246.1100; Found 246.1110.

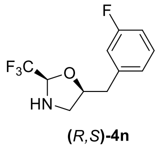

(*R,S*)-**4n**

(2R,5S)-5-(3-fluorobenzyl)-2-(trifluoromethyl)oxazolidine ((*R,S*)-4n)

Prepared according to the general procedure D3 using **3n** (68 mg, 0.20 mmol, 1.0 equiv., 90% ee). The crude material was purified by flash column chromatography (pentane/EtOAc gradient 100:0 to 80:20) to give the corresponding product *(R,S)*-**4n** (41 mg, 0.16 mmol, 82% yield) as a colorless liquid. The enantiomeric excess was determined to be 90% by HPLC analysis on a Daicel Chiralpak IA column: 95:5 hexane/IPA, flow rate 1 mL/min, λ = 254 nm: τ_{Major} = 12.2 min, τ_{Minor} = 9.8 min. Absolute and relative configuration were determined by X-Ray diffraction analysis of a single crystal of *(R,S)*-**4b**.

R_f value: 0.37 (20% Ethyl acetate in Pentane).

[α]D^{20} = +5.1 (c = 0.51, $CHCl_3$, 90% ee).

^{1}H NMR (400 MHz, Chloroform-*d*) δ 7.33 – 7.21 (m, 1H, Ar*H*), 7.00 (dt, *J* = 7.6, 1.3 Hz, 1H, Ar*H*), 6.97 – 6.89 (m, 2H, Ar*H*), 4.94 (q, *J* = 5.3 Hz, 1H, C*H*CF_3), 4.10 (dq, *J* = 8.8, 6.0 Hz, 1H, OC*H*), 3.29 (t, *J* = 8.3 Hz, 1H, NCH_aH$_b$), 3.04 (dd, *J* = 13.9, 7.1 Hz, 1H, ArCH_aH$_b$), 2.81 (dd, *J* = 13.9, 5.9 Hz, 2H, ArCH$_a$$H_b$ + NCH$_a$$H_b$), 2.66 (s, 1H, N*H*).

^{13}C{^{1}H} NMR (101 MHz, Chloroform-*d*) δ 163.0 (d, *J* = 245.9 Hz), 140.2 (d, *J* = 7.3 Hz), 130.1 (d, *J* = 8.3 Hz), 124.9 (d, *J* = 2.9 Hz), 123.4 (q, *J* = 282.7 Hz), 116.1 (d, *J* = 21.2 Hz), 113.7 (d, *J* = 21.0 Hz), 88.1 (q, *J* = 34.0 Hz), 80.3, 50.7, 39.6 (d, *J* = 1.8 Hz).

^{19}F{^{1}H} NMR (376 MHz, Chloroform-*d*) δ -81.4 (s, 3F, CHCF_3), -113.3 (s, 1F, Ar*F*).

IR (cm^{-1}) 3356 (w), 2931 (w), 1593 (w), 1450 (w), 1288 (m), 791 (m), 1254 (m), 1489 (w), 868 (m), 941 (w).

HRMS (ESI/QTOF) m/z: [M + H]$^+$ Calcd for $C_{11}H_{12}F_4NO^+$ 250.0850; Found 250.0855.

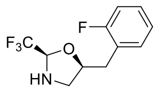

(*R*,*S*)-**4q**

(2R,5S)-5-(2-Fluorobenzyl)-2-(trifluoromethyl)oxazolidine ((*R*,*S*)-4q)

Prepared according to the general procedure D3 using **3q** (34 mg, 0.20 mmol, 1.0 equiv., 84% ee). The crude material was purified by flash column chromatography (pentane/EtOAc gradient 100:0 to 80:20) to give the corresponding product *(R,S)*-**4q** (18 mg, 0.072 mmol, 72% yield) as colorless oil. The enantiomeric excess was determined to be 84% by HPLC analysis on a Daicel Chiralpak IA column: 95:5 hexane/IPA, flow rate 1 mL/min, λ = 210 nm: τ_{Major} = 12.2 min, τ_{Minor} = 7.6 min. Absolute configuration was determined in comparison to compound *(R,S)*-**4b**.

R_f value: 0.38 (20% Ethyl acetate in Pentane).

[α]D^{20} = 11.1 (c = 0.48, $CHCl_3$, 84% ee).

^{1}H NMR (400 MHz, Chloroform-*d*) δ 7.30 – 7.18 (m, 2H, Ar*H*), 7.13 – 6.98 (m, 2H, Ar*H*), 4.93 (d, *J* = 5.7 Hz, 1H, C*H*CF_3), 4.16 (dq, *J* = 9.0, 6.3 Hz, 1H, OC*H*), 3.38 – 3.24 (m, 1H, NCH_aH$_b$), 3.02 (ddd, *J* = 13.9, 6.8, 1.3 Hz, 1H, ArCH_aH$_b$), 2.94 (ddd, *J* = 13.9, 6.2, 1.3 Hz, 1H, ArCH$_a$$H_b$), 2.84 (q, *J* = 8.9, 8.4 Hz, 1H, NCH$_a$$H_b$), 2.66 (s, 1H, N*H*).

^{13}C{^{1}H} NMR (101 MHz, Chloroform-*d*) δ 161.2 (d, *J* = 245.0 Hz), 131.8 (d, *J* = 4.7 Hz), 128.7 (d, *J* = 8.1 Hz), 124.5 (d, *J* = 15.9 Hz), 124.3 (d, *J* = 3.6 Hz), 123.5 (q, *J* = 283.1 Hz), 115.4 (d, *J* = 22.0 Hz), 88.0 (q, *J* = 34.0 Hz), 79.3, 50.7, 33.0 (d, *J* = 1.7 Hz).

^{19}F{^{1}H} NMR (376 MHz, Chloroform-*d*) δ -81.4 (s, 3F, CHCF_3), -118.2 (s, 1F, Ar*F*).

IR (cm^{-1}) 3348 (w), 2939 (w), 1585 (w), 1493 (m), 1454 (w), 1292 (m), 1230 (m), 1165 (s).

HRMS (ESI/QTOF) m/z: [M + H]$^+$ Calculated for $C_{11}H_{12}F_4NO^+$ 250.0850; Found 250.0852.

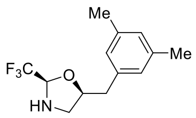

(*R*,*S*)-**4r**

(2R,5S)-5-(3,5-Dimethylbenzyl)-2-(trifluoromethyl)oxazolidine ((*R*,*S*)-4r)

Prepared according to the general procedure D3 using **3r** (70 mg, 0.20 mmol, 1.0 equiv., 86% ee). The crude material was purified by flash column chromatography (pentane/EtOAc gradient 100:0 to 80:10) to give the corresponding product *(R,S)*-**4r** (43 mg, 0.17 mmol, 83% yield) as colorless oil. The enantiomeric excess was determined to be 84% by HPLC analysis on a Daicel Chiralpak IA column: 95:5 hexane/IPA, flow rate 1 mL/min, λ = 210 nm: τ_{Major} = 8.8 min, τ_{Minor} = 6.5 min. Absolute configuration was determined in comparison to compound *(R,S)*-**4b**.

R_f value: 0.40 (20% Ethyl acetate in Pentane).

[α]D^{20} = 14.6 (c = 0.49, $CHCl_3$, 84% ee).

^{1}H NMR (400 MHz, Chloroform-*d*) δ 6.88 (s, 1H, Ar*H*), 6.84 (s, 2H, Ar*H*), 4.93 (dq, *J* = 8.6, 5.6 Hz, 1H, C*H*CF_3), 4.10 (dq, *J* = 9.2, 6.4 Hz, 1H, OC*H*), 3.25 (dddd, *J* = 11.8, 7.3, 5.7, 1.4 Hz, 1H, NCH_aH$_b$), 3.02 (dd, *J* = 13.6, 6.6 Hz, 1H, ArCH_aH$_b$), 2.86 – 2.76 (m, 1H, NCH$_a$$H_b$), 2.72 (dd, *J* = 13.6, 6.7 Hz, 1H, ArCH$_a$$H_b$), 2.61 (q, *J* = 9.5, 8.9 Hz, 1H, N*H*), 2.30 (s, 6H, 2 x ArCH_3).

^{13}C{^{1}H} NMR (101 MHz, Chloroform-*d*) δ 138.2, 137.5, 127.0, 123.5 (q, *J* = 282.8 Hz), 88.0 (q, *J* = 33.8 Hz), 80.8, 50.8, 39.7, 21.4.

^{19}F{^{1}H} NMR (376 MHz, Chloroform-*d*) δ -81.4.

IR (cm^{-1}) 3348 (w), 3012 (w), 2931 (w), 1709 (w), 1608 (w), 1458 (w), 1292 (m), 1165 (s), 1103 (m).

HRMS (ESI/QTOF) m/z: [M + H]$^+$ Calculated for $C_{13}H_{17}F_3NO^+$ 260.1257; Found 260.1262.

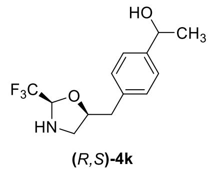

1-(4-(((2R,5S)-2-(Trifluoromethyl)oxazolidin-5-yl)methyl)phenyl)ethan-1-ol ((*R,S)*-4k)

Prepared according to the general procedure D3 using **3k** (72 mg, 0.20 mmol, 1.0 equiv., 90% ee). The crude material was purified by flash column chromatography (pentane/EtOAc gradient 100:0 to 80:20) to give the corresponding product *(R,S)*-**4k** (45 mg, 0.16 mmol, 82% yield) as a colorless amorphous solid as a mixture of diasteteisomers in equal amounts. The enantiomeric excess was determined to be 88% by HPLC analysis on a Daicel Chiralpak IB column: 80:20 hexane/IPA, flow rate 1 mL/min, λ = 210 nm: τ_{Major} = 17.1 min, 15.5 min, τ_{Minor} = 11.3 min, 10.4 min. Absolute and relative configuration were determined by X-Ray diffraction analysis of a single crystal of *(R,S)*-**4b**.

R_f value: 0.25 (20% Ethyl acetate in Pentane).

$[\alpha]D^{20}$ = +8.2 (c = 0.46, $CHCl_3$, 88% ee).

^{1}H NMR (400 MHz, Chloroform-*d*) δ 7.32 – 7.25 (m, 2H, Ar*H*), 7.24 – 7.16 (m, 2H, Ar*H*), 4.94 (dq, *J* = 8.5, 5.9 Hz, 1H, C*H*CF_3), 4.77 (qd, *J* = 6.5, 4.1 Hz, 1H, CH_3C*H*(OH)), 4.02 (dq, *J* = 12.9, 5.7 Hz, 1H, OC*H*), 3.30 – 3.15 (m, 2H, NC$H_a$$H_b$+ NC$H_a$$H_b$), 3.13 (d, *J* = 4.2 Hz, 1H, CH_3CH(O*H*)), 2.89 (dd, *J* = 13.9, 7.4 Hz, 1H, ArC$H_a$$H_b$), 2.81 (dd, *J* = 13.8, 5.7 Hz, 1H, ArC$H_a$$H_b$), 2.68 – 2.56 (m, 1H, N*H*), 1.37 (d, *J* = 6.5 Hz, 3H, CH_3CH(OH)).

^{13}C{^{1}H} NMR (101 MHz, Chloroform-*d*) δ 144.3, 136.9, 129.3, 125.8, 123.5 (q, *J* = 282.7 Hz), 88.0 (q, *J* = 33.8 Hz), 80.7, 70.3, 50.8, 39.6, 25.2.

^{19}F{^{1}H} NMR (376 MHz, Chloroform-*d*) δ -81.4 (s, 3F, CHC*F*$_3$).

IR (cm^{-1}) 3351 (w), 3344 (m), 2931 (w), 1666 (w), 1446 (w), 1292 (m), 1157 (s), 1095 (s).

HRMS (ESI/QTOF) m/z: $[M + Na]^+$ Calcd for $C_{13}H_{16}F_3NNaO_2^+$ 298.1025; Found 298.1030.

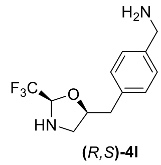

(4-(((2R,5S)-2-(Trifluoromethyl)oxazolidin-5-yl)methyl)phenyl)methanamine ((*R,S)*-4l)

Prepared according to the general procedure D3 using **3l** (69 mg, 0.20 mmol, 1.0 equiv., 90% ee). The crude material was purified by flash column chromatography (DCM/MeOH gradient 100:0 to 90:10) to give the corresponding product *(R,S)*-**4l** (29 mg, 0.11 mmol, 56% yield) as colorless oil. The enantiomeric excess was determined to be 88% by HPLC analysis on a Daicel Chiralpak IC column: 80:20 hexane/IPA, flow rate 1 mL/min, λ = 210 nm: τ_{Major} = 13.5 min, τ_{Minor} = 10.6 min. Absolute configuration was determined in comparison to compound *(R,S)*-**4b**.

R_f value: 0.28 (20% Ethyl acetate in Pentane).

$[\alpha]D^{20}$ = 54.5 (c = 0.40, $CHCl_3$, 89% ee).

^{1}H NMR (400 MHz, Chloroform-*d*) δ 7.25 (d, *J* = 6.8 Hz, 2H, Ar*H*), 7.19 (d, *J* = 8.1 Hz, 2H, Ar*H*), 4.92 (q, *J* = 5.6 Hz, 1H, C*H*CF_3), 4.09 (dq, *J* = 9.0, 6.3 Hz, 1H, OC*H*), 3.85 (s, 2H, C$H_2$$NH_2$), 3.26 (ddd, *J* = 11.9, 5.6, 1.5 Hz, 1H, NC$H_a$$H_b$), 3.04 (dd, *J* = 13.8, 6.9 Hz, 1H, ArC$H_a$$H_b$), 2.88 – 2.73 (m, 2H, NC$H_a$$H_b$ + ArC$H_a$$H_b$), 2.44 – 1.72 (br. s., 3H, N*H* + NH_2).

^{13}C{^{1}H} NMR (101 MHz, Chloroform-*d*) δ 141.6, 136.3, 129.4, 127.5,123.5 (q, *J* = 282.5 Hz), 88.0 (q, *J* = 33.9 Hz), 80.7, 50.8, 46.2, 39.6.

^{19}F{^{1}H} NMR (376 MHz, Chloroform-*d*) δ -81.4.

IR (cm^{-1}) 3344 (w), 3020 (w), 2935 (w), 1589 (w), 1454 (w), 1292 (m), 1161 (s)

HRMS (ESI/QTOF) m/z: $[M + H_{-2}N_{-1}]^+$ Calculated for $C_{12}H_{13}F_3NO^+$ 244.0944; Found 244.0947.

(S)-1-Amino-3-phenylpropan-2-ol 2,2,2-trifluoroacetic acid salt (11)

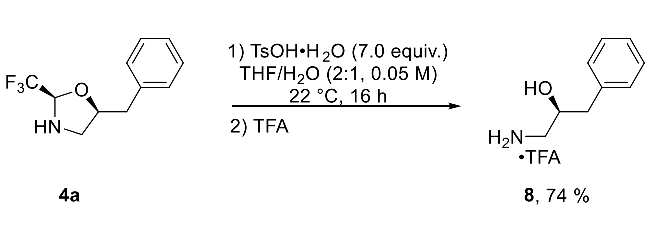

Scheme 9. Acidic hydrolysis of the hemiaminal, synthesis of **8**

In 5 mL round bottom flask **4a** (69 mg, 0.30 mmol, 90% ee) was dissolved in a mixture of THF (5.4 mL) and H_2O (0.6 mL). Tosylsulfonic acid (400 mg, 2.10 mmol, 7.0 equiv) was added and the mixture was stirred at

room temperature for 16 hours. The reaction was diluted with DCM (10 mL) and quenched by adding 1 M NaOH (6 mL). The layers were separated, and the aqueous layer was extracted with DCM (2 x 10 mL). The combined organic layers were washed with brine, dried over Na_2SO_4, filtered and concentrated. The crude material was purified by preparative RP-HPLC on an Agilent 1260 HPLC system with a G2260A 1260 Prep ALS Autosampler, a G1361a 1260 Prep Pump, a G1365C 1260 MWD detector and a G1364B 1260 FC-PS collector, coupled with a Waters XBridge semi-preparative C18 column (19 x 150 mm, 5 µm). Water (solvent A) and water:acetonitrile 5:95 (solvent B), each containing 0.1% TFA, were used as the mobile phase at a flow rate of 20 mL.min-1. The following method was used: 100% A to 100% B in 20 minutes. The desired product (S)-1-amino-3-phenylpropan-2-ol 2,2,2-trifluoroacetic acid salt **8** was obtained as gummy solid (62 mg, 0.23 mmol, 74%).

$[\alpha]D^{20}$ = -0.45 (c = 0.40, $CHCl_3$).

^{1}H NMR (400 MHz, MeOD) δ 7.35 – 7.18 (m, 5H, Ar*H*), 3.99 (dtd, *J* = 9.7, 6.7, 3.0 Hz, 1H, HOC*H*), 2.98 (dd, *J* = 12.8, 3.0 Hz, 1H, $H_2NCH_aH_b$), 2.89 – 2.72 (m, 3H, $H_2NCH_aH_b$+ ArCH_2).

^{13}C{^{1}H} NMR (101 MHz, Chloroform-*d*) δ 161.7 (q, *J* = 34.3 Hz), 137.2, 129.0, 128.2, 126.3, 116.8 (q, *J* = 292.9 Hz), 68.6, 44.03, 41.3.

^{19}F NMR (376 MHz, MeOD) δ -76.9 (s, 3F, $^-OOCCF_3$).

IR (cm^{-1}) 3398 (w), 2933 (m), 1676 (s), 1137 (s), 840 (m), 801 (m), 748 (m), 724 (m), 702 (m).

HRMS (APCI/QTOF) m/z: $[M]^+$ Calcd for $C_9H_{14}NO^+$ 152.1070; Found 152.1072.

D.5. Unsuccessful Substrates

Unreactive propargylic amines, aryl iodides and failed hydrogenations are reported in the following scheme. Yields are reported in the case of low conversions.

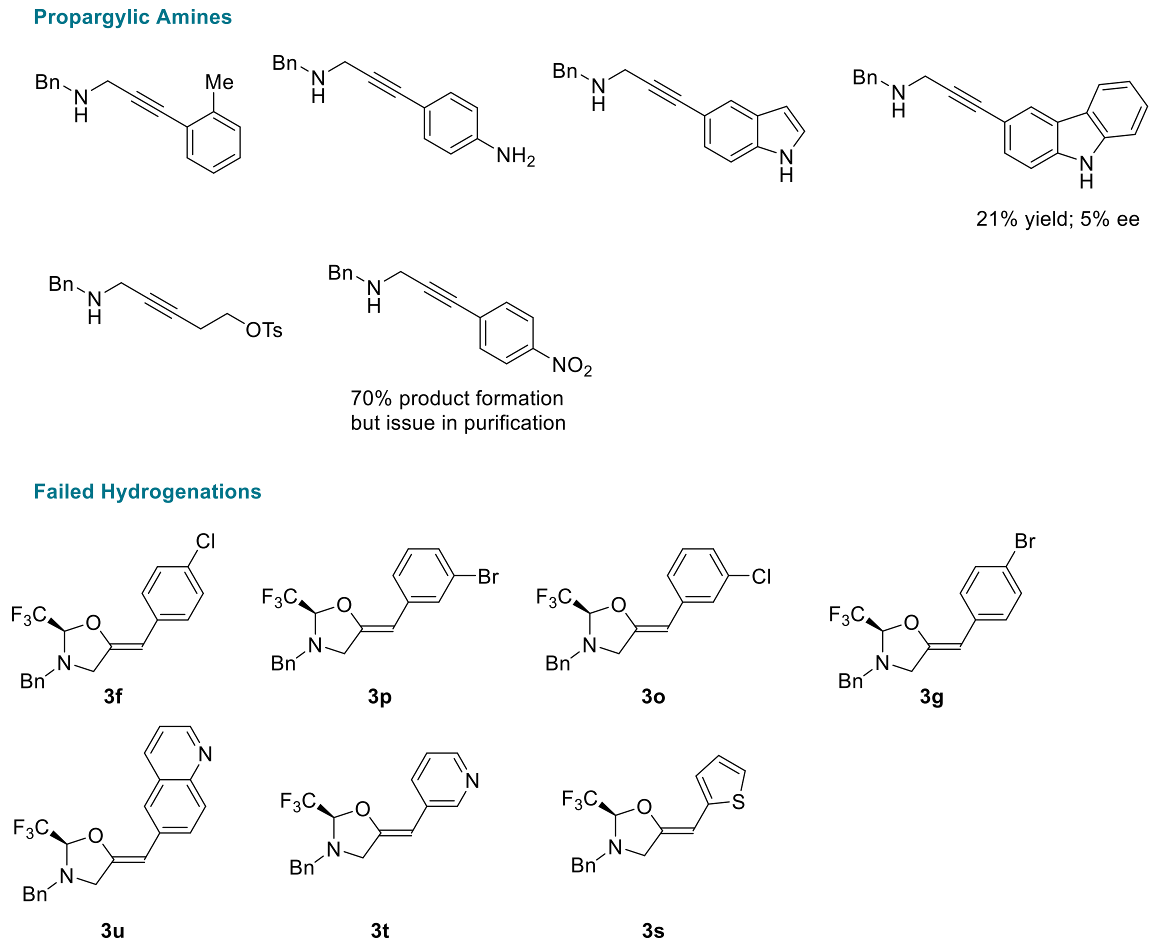

Scheme 10. Unsuccessful substrates and scope limitations.

E. NMR studies

In order to gain more insight on the reaction mechanism, we performed some NMR studies (Figure 1). First, according to Trost et. al.[16] we mixed Pd_2dba_3•$CHCl_3$ with **L1** in THF/C_6D_6 (4:1 v/v). Two doublets appeared δ 24.44 (d, J = 14.7 Hz), 22.02 (d, J = 14.7 Hz) ppm in the $^{31}P\{^1H\}$ NMR characteristic of bidentate Pd(0)L•dba complex (Figure 1, spectra 1). Then, *ortho*-iodoanisole (**7a**) was added. Interestingly, the two doublets disappeared and two new singlets appeared at δ 18.14 and 17.94 ppm (Figure 1, spectra 2). The same species was observed in a filtered reaction mixture before the addition of the propargylic amine **1a** and the tether **2** (Figure 1, spectra 3). The reaction mixture was also probed after full conversion. In this case only two new unidentified singlets at δ 30.10 and 25.67 ppm were observed (Figure 1, spectra 4). However, since during the reaction the ArI additive is slowly being consumed, it is possible that after the reaction no more intermediate **III** would be present. Therefore, the reaction was run with high loading of the ArI additive (1.0 equiv.). In this case along with the aforementioned new peaks at δ 30.10 and 25.67 ppm, the characteristic signals of ArI adducts **III** at δ 18.14 and 17.94 ppm were observed (Figure 1, spectra 5). These experiments indicate that indeed an ArI oxidative addition complex is present in the reaction mixture and may be the active catalyst of the reaction. What remains unclear is the structure of this complex, since no coupling in $^{31}P\{^1H\}$ NMR was observed. This means that the structure of this complex is not the classic tetrasubstituted square planar bidentate complex with the two phosphines in *cis* position.[17]

Figure 1. NMR studies of the *trans*-hydroalkoxylation reaction.[a]

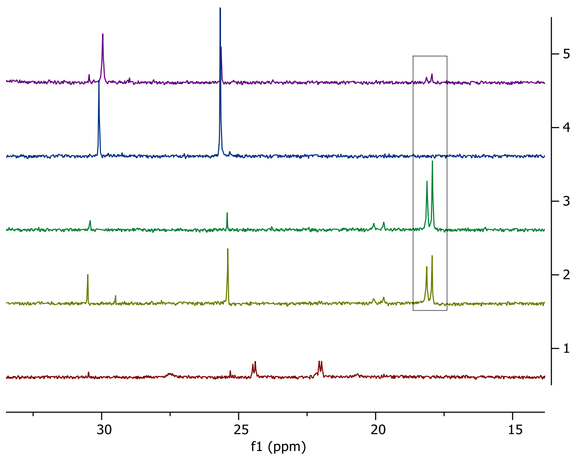

[a]NMR studies. 1 – in situ prepared Pd(0)L•dba; 2 – in situ prepared intermediate **III.** 3 – filtered reaction mixture before heating. 4 – filtered reaction mixture after heating; 5 – filtered reaction mixture with high **7a** loading (1.0 equiv.) after heating.

31P{1H} NMR spectra were recorded in a mixture of THF/C_6D_6 (4:1 v/v, degassed by freeze-pump-thaw). ^1H was referenced by $Si(CH_3)_4$ internal standard (δ 0 ppm) and $^{31}P\{^1H\}$ was referenced using Ξ-scales with 85% H_3PO_4 (Ξ=40.480747 MHz, ^{31}P) as secondary reference.

$^{31}P\{^1H\}$ spectra of **L1**:

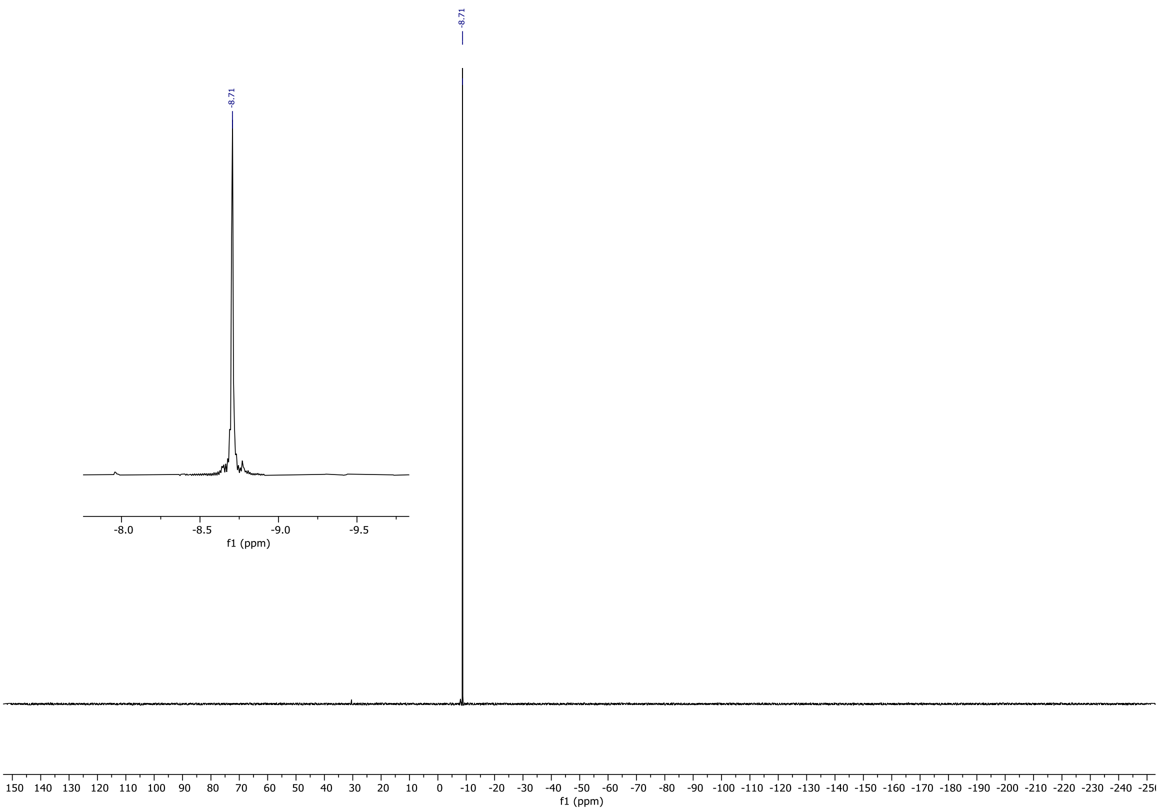

$^{31}P\{^1H\}$ spectra of **L1** + Pd_2dba_3•$CHCl_3$ (approx. 10 minutes after mixing):

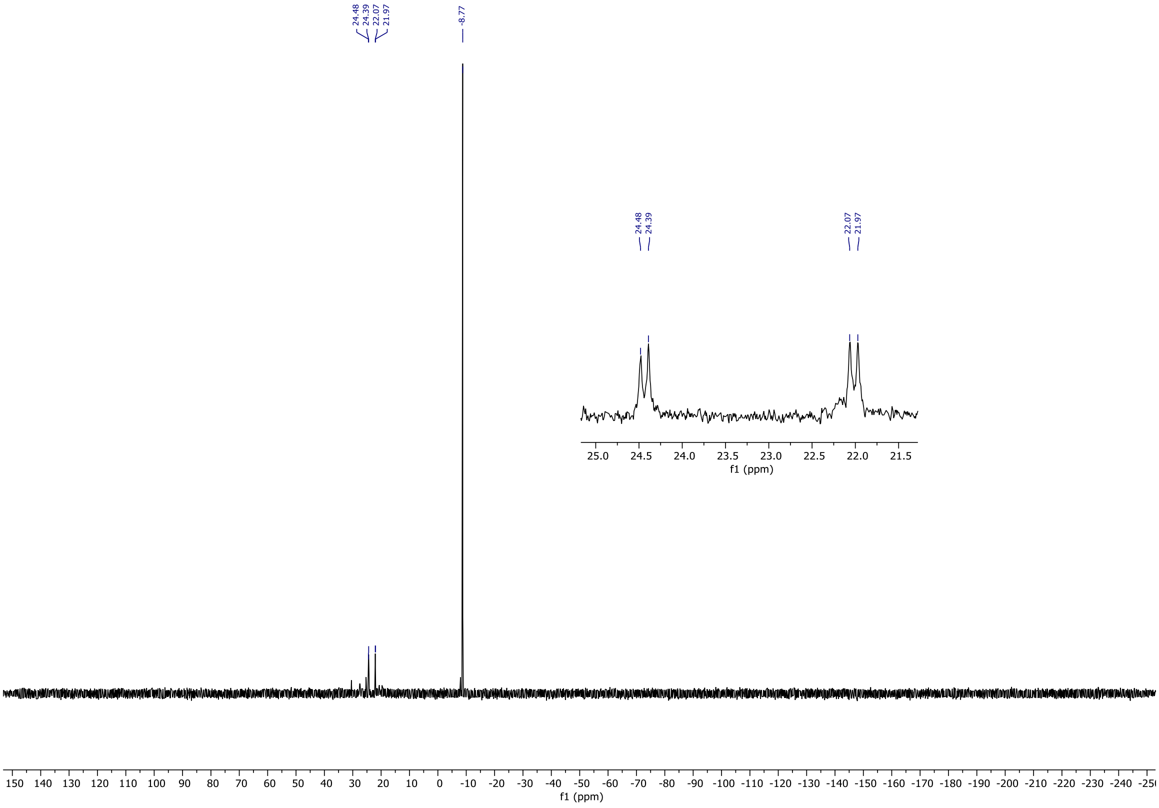

$^{31}P\{^{1}H\}$ spectra of **L1** + $Pd_2dba_3 \bullet CHCl_3$ + ortho-iodoanisole:

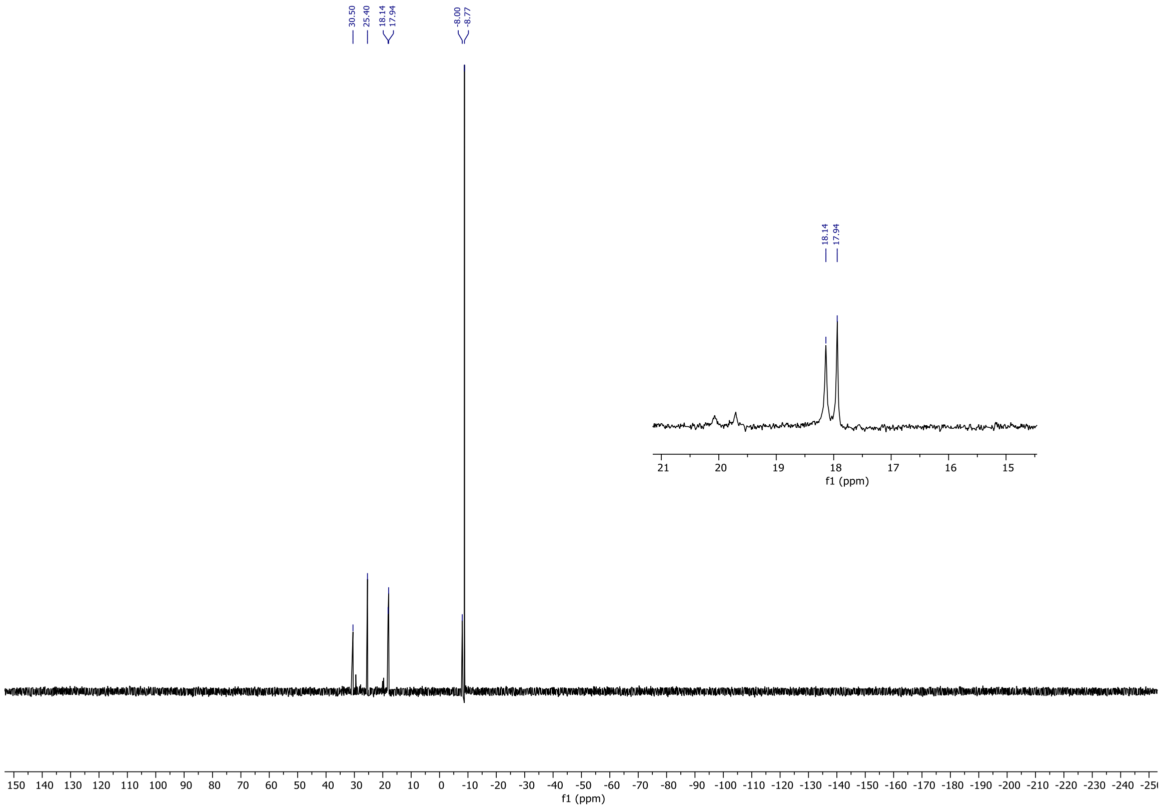

$^{31}P\{^{1}H\}$ spectra of reaction mixture before the start of the reaction:

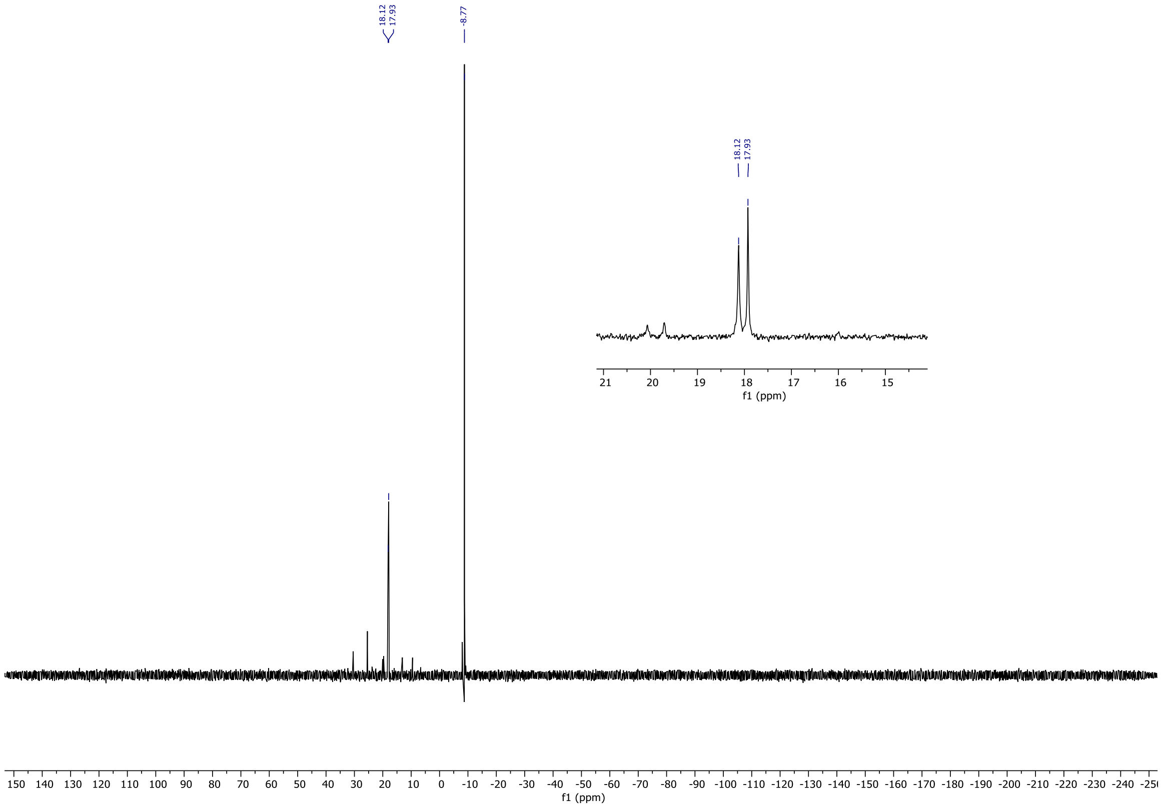

$^{31}P\{^{1}H\}$ spectra of reaction after heating the reaction mixture for 16h:

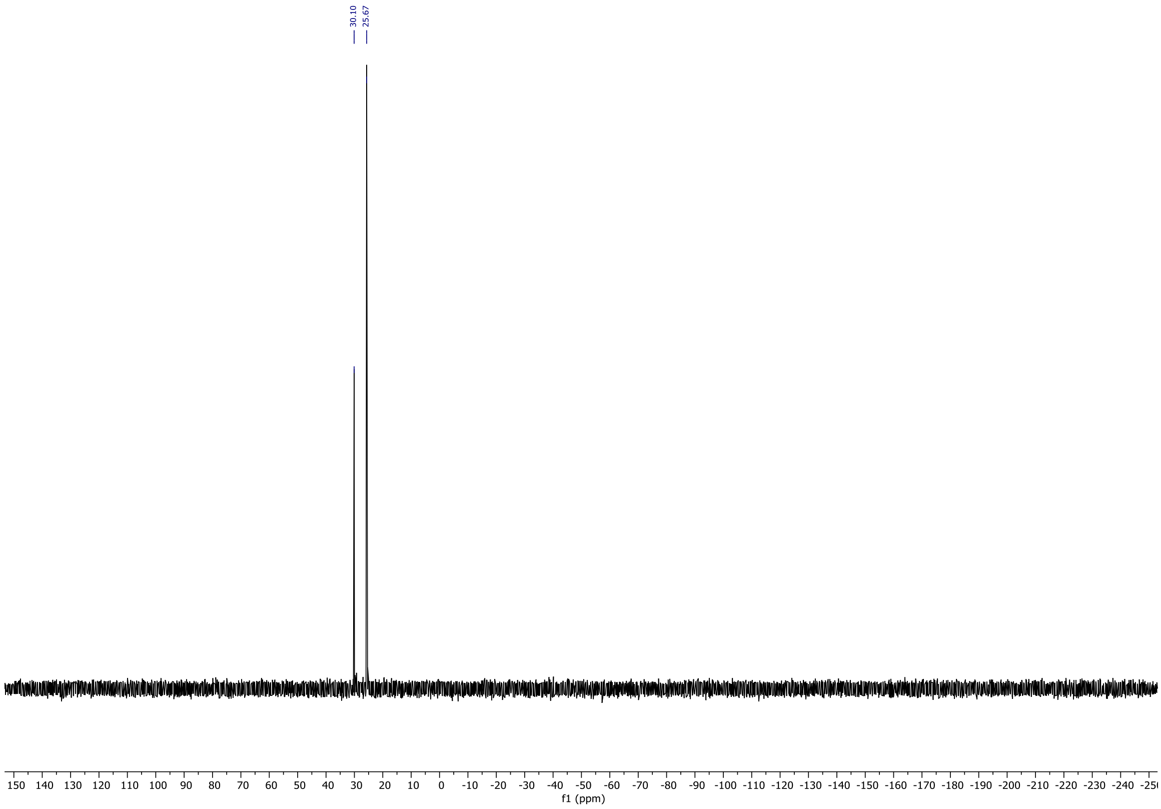

$^{31}P\{^{1}H\}$ spectra of reaction after heating the reaction mixture for 16h with high ortho-iodoanisole loading (1.0 equiv.):

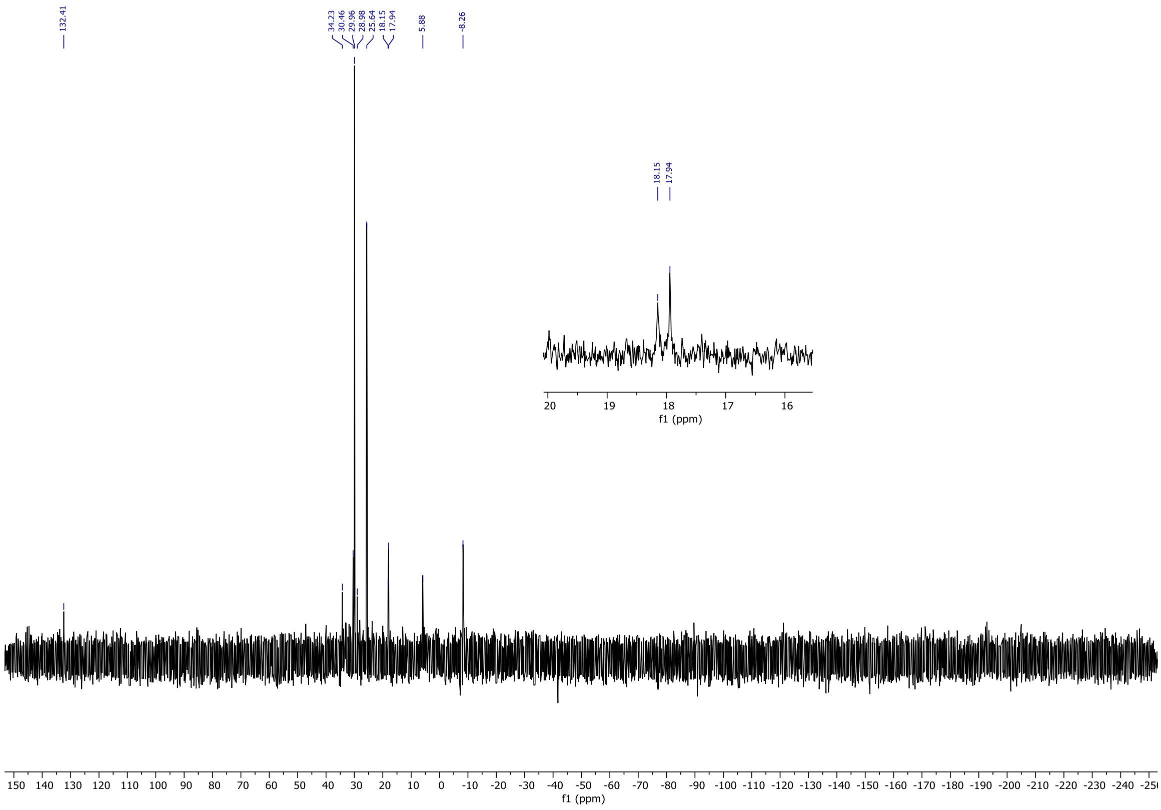

F. X-Ray Crystallographic Data

F.1. Single Crystal X-Ray Diffraction for the chiral compound *(S)*-3a

Crystals of the compound *(S)*-**4** were obtained by slow evaporation of a hexane/isopropanol solution.

Data acquisition: Single clear pale colourless prism crystals of **(*S*)-3a** were used as supplied. A suitable crystal with dimensions 0.60 × 0.48 × 0.35 mm^3 was selected and mounted on a SuperNova, Dual, Cu at home/near, Atlas diffractometer. The crystal was kept at a steady T = 140.00(10) K during data collection. The structure was solved with the ShelXS (Sheldrick, 2008) solution program using direct methods and by using Olex2 (Dolomanov et al., 2009) as the graphical interface. The model was refined with ShelXL 2018/3 (Sheldrick, 2015) using full matrix least squares minimisation on $\boldsymbol{F}^2$

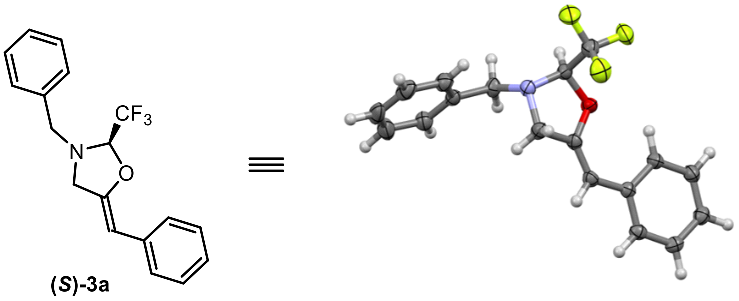

Scheme 11: Crystal data and structure refinement for ***(S)*-3a. CCDC 2126130**

Crystal Data. $C_{18}H_{16}F_3NO$, M_r = 319.32, orthorhombic, $P2_12_12_1$ (No. 19), a = 8.36428(10) Å, b = 10.91132(12) Å, c = 17.10096(18) Å, $\alpha = \beta = \gamma = 90^\circ$, V = 1560.72(3) Å^3, T = 140.00(10) K, Z = 4, Z' = 1, μ(Cu K_α) = 0.923, 16378 reflections measured, 3257 unique (R_{int} = 0.0120) which were used in all calculations. The final wR_2 was 0.0607 (all data) and R_1 was 0.0231 (I≥2 σ(I)).

**Compound**	**3a**
Formula	$C_{18}H_{16}F_3NO$
$D_{calc.}$/ g cm^{-3}	1.359
μ/mm^{-1}	0.923
Formula Weight	319.32
Colour	clear pale colourless
Shape	prism
Size/mm^3	0.60×0.48×0.35
T/K	140.00(10)
Crystal System	orthorhombic
Flack Parameter	0.022(14)
Hooft Parameter	0.048(12)
Space Group	$P2_12_12_1$
a/Å	8.36428(10)
b/Å	10.91132(12)
c/Å	17.10096(18)
α/°	90
β/°	90
γ/°	90
V/Å^3	1560.72(3)
Z	4
Z'	1
Wavelength/Å	1.54184
Radiation type	Cu K_α
Θ_{min}/°	4.808
Θ_{max}/°	76.213
Measured Refl's.	16378
Indep't Refl's	3257
Refl's I≥2 σ(I)	3238
R_{int}	0.0120
Parameters	273
Restraints	0
Largest Peak	0.138
Deepest Hole	-0.114
GooF	1.043
wR_2 (all data)	0.0607
wR_2	0.0606
R_1 (all data)	0.0233
R_1	0.0231

F.2. Single Crystal X-Ray Diffraction for the chiral compound 4b

Crystals of the compound **5** were obtained by slow evaporation of a hexane/isopropanol (10:1) solution.

Data Acquisition: Single colourless plate crystals of **4b** were used as supplied. A suitable crystal with dimensions 0.40 × 0.10 × 0.05 mm^3 was selected and mounted on a SuperNova, Dual, Cu at home/near, Atlas diffractometer. The crystal was kept at a steady T = 140.00(10) K during data collection. The structure was solved with the ShelXT 2018/2 (Sheldrick, 2015) solution program using dual methods and by using Olex2 (Dolomanov et al., 2009) as the graphical interface. The model was refined with ShelXL 2018/3 (Sheldrick, 2015) using full matrix least squares minimisation on $\boldsymbol{F}^2$.

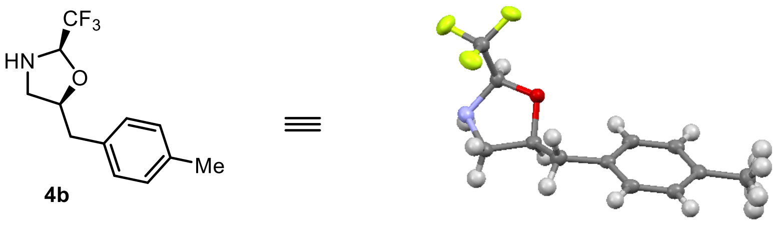

Scheme 12: Crystal data and structure refinement for **4b. CCDC 2126132**

**Compound**	**4b**
Formula	$C_{12}H_{14}F_3NO$
$D_{calc.}$/ g cm^{-3}	1.419
μ/mm^{-1}	1.066
Formula Weight	245.24
Colour	colourless
Shape	plate
Size/mm^3	0.40×0.10×0.05
T/K	140.00(10)
Crystal System	orthorhombic
Flack Parameter	-0.04(3)
Space Group	$P2_12_12_1$
a/Å	5.65596(10)
b/Å	7.72749(12)
c/Å	26.2606(4)
α/°	90
β/°	90
γ/°	90
V/Å^3	1147.76(3)
Z	4
Z'	1
Wavelength/Å	1.54184
Radiation type	Cu$K\alpha$
Θ_{min}/°	3.366
Θ_{max}/°	72.464
Measured Refl's.	18872
Indep't Refl's	2256
Refl's I≥2σ(I)	2187
R_{int}	0.0269
Parameters	161
Restraints	0
Largest Peak/e Å^{-3}	0.144
Deepest Hole/e Å^{-3}	-0.173
GooF	1.058
wR_2 (all data)	0.0585
wR_2	0.0575
R_1 (all data)	0.0246
R_1	0.0231

Crystal Data. $C_{12}H_{14}F_3NO$, M_r = 245.24, orthorhombic, $P2_12_12_1$ (No. 19), a = 5.65596(10) Å, b = 7.72749(12) Å, c = 26.2606(4) Å, $\alpha = \beta = \gamma = 90°$, V = 1147.76(3) Å^3, T = 140.00(10) K, Z = 4, Z' = 1, μ(Cu K_α) = 1.066, 18872 reflections measured, 2256 unique (R_{int} = 0.0269) which were used in all calculations. The final wR_2 was 0.0585 (all data) and R_1 was 0.0231 (I≥2 σ(I)).

G. References

1. Sheldrick, G. M. SHELXT – Integrated space-group and crystal-structure determination. *Acta Cryst A* **71**, 3–8 (2015).
2. Sheldrick, G. M. Crystal structure refinement with SHELXL. *Acta Cryst C* **71**, 3–8 (2015).
3. Zalesskiy, S. S. & Ananikov, V. P. $Pd_2(dba)_3$ as a Precursor of Soluble Metal Complexes and Nanoparticles: Determination of Palladium Active Species for Catalysis and Synthesis. *Organometallics* **31**, 2302–2309 (2012).
4. Greenwood, P. D. G. & Waser, J. Palladium-Catalyzed Carboxy-Alkynylation of Propargylic Amines Using Carbonate Salts as Carbon Dioxide Source: Palladium-Catalyzed Carboxy-Alkynylation of Propargylic Amines Using Carbonate Salts as Carbon Dioxide Source. *Eur. J. Org. Chem.*, 5183–5186 (2019).
5. Althuon, D.; Rönicke, F.; Fürniss, D.; Quan, J.; Wellhöfer, I.; Jung, N.; Schepers, U.; Bräse, S. *Org. Biomol. Chem.* **13**, 4226–4230 (2015).
6. Hashmi, A. S. K. *et al.* Gold catalysis: Non-spirocyclic intermediates in the conversion of furanynes by the formal insertion of an alkyne into an aryl-alkyl C-C single bond. *Chem. Eur. J.* **18**, 10480–10486 (2012).
7. Kundu, N. G. & Chaudhuri, G. Heteroannulation through copper catalysis : a novel and highly regio- and stereoselective cyclisation of alkynes leading to (E) -2- (2-arylvinyl) quinazolinones. *Tetrahedron Lett.* **42**, 2883–2886 (2001).
8. Reis, O., Koyuncu, H., Esiringu, I., Sahin, Y. & Gulcan, O. H. A New Method for the Synthesis of Rasagiline. U.S. Patent No US 8,901,352 B2, 2014
9. Shao, Y., Zhang, F., Zhang, J. & Zhou, X. Lanthanide-Catalyzed Reversible Alkynyl Exchange by Carbon–Carbon Single-Bond Cleavage Assisted by a Secondary Amino Group. *Angew. Chem. Int. Ed.* **55**, 11485–11489 (2016).
10. García-Domínguez, P., Fehr, L., Rusconi, G. & Nevado, C. Palladium-catalyzed incorporation of atmospheric CO2: Efficient synthesis of functionalized oxazolidinones. *Chem. Sci.* **7**, 3914–3918 (2016).
11. Greenwood, P. D. G., Grenet, E. & Waser, J. Palladium-Catalyzed Carbo-Oxygenation of Propargylic Amines using in Situ Tether Formation. *Chem. Eur. J.* **2019**, 3010–3013 (2019)

12. Xu, H.-H., Zhang, X.-H. & Zhang, X.-G. Copper-Catalyzed Tandem Sulfuration/Annulation of Propargylamines with Sulfur via C–N Bond Cleavage. *J. Org. Chem.* **84**, 7894–7900 (2019).

13. Buzzetti, L., Purins, M., Greenwood, P. D. G., Waser, J. Enantioselective Carboetherification/hydrogenation for the Synthesis of Amino Alcohols via a Catalytically Formed Chiral Auxiliary. *J. Am. Chem. Soc*. **142**, 17334-17339 (2020).

14. Tang, C.-Y., Wu, X.-Y., Sha, F., Rhodium(III)-Catalyzed Direct C-2 Olefination of Unactivated Indoles Utilizing OH/NH2 as Directing Group *Adv. Synth. Catal* . **2014**, *356*, 609-615.

15. Bobileva, O., Ikaunieks, M., Duburs, G., Mandrika, I., Petrovska, R., Klovins, J., Loza, E., Synthesis and evaluation of (*E*)-2-(5-phenylpent-2-en-4-ynamido)cyclohex-1-ene-1-carboxylate derivatives as HCA2 receptor agonists *Bioorg. Med. Chem.* **2017**, *25*, 4314-4329.

16. Trost, B. M., Breit, B., Organ, M. G., On the nature of the asymmetric induction in a palladium catalyzed allylic alkylation. *Tetrahedron Lett.* **1994**, *35*, 5817-5820.

17. Monccarz, J. R., Brunker, T. J., Jewett, J. C.; Orchowski, M.; Glueck, D. S., Sommer, R. D., Lam, K.-C., Incarvito, C. D.; Concolino, T. E., Ceccarelli, C., Zakharov, L. N., Rheingold, A. L., Palladium-Catalyzed Asymmetric Phosphination. Enantioselective Synthesis of PAMP−BH3, Ligand Effects on Catalysis, and Direct Observation of the Stereochemistry of Transmetalation and Reductive Elimination. *Organometallics* **2003**, *22*, 3205-3221.

H. HPLC Spectra

HPLC Spectra for the Enantioselective Cyclization of propargylic amines

Chiral HPLC Daicel Chiralpak IB N-5 column: 99:1 hexane/IPA, flow rate 1 mL/min, λ = 254 nm

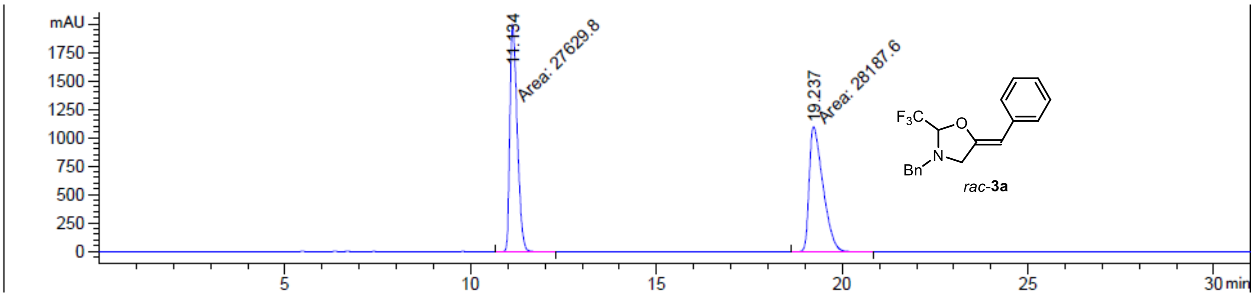

Signal 1: DAD1 A, Sig=254,4 Ref=360,100

Peak #	RetTime [min]	Type	Width [min]	Area [mAU*s]	Height [mAU]	Area %
1	11.134	MM	0.2310	2.76298e4	1993.85388	49.5003
2	19.237	MM	0.4288	2.81876e4	1095.56396	50.4997

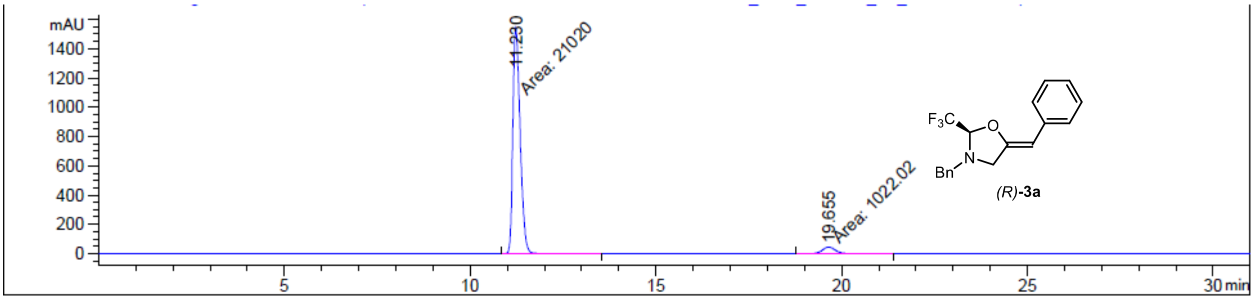

Signal 1: DAD1 A, Sig=254,4 Ref=360,100

Peak #	RetTime [min]	Type	Width [min]	Area [mAU*s]	Height [mAU]	Area %
1	11.230	MM	0.2264	2.10200e4	1547.46667	95.3633
2	19.655	MM	0.3855	1022.02258	44.18879	4.6367

Chiral HPLC Daicel Chiralpak IB N-5 column: 99:1 hexane/IPA, flow rate 1 mL/min, λ = 254 nm

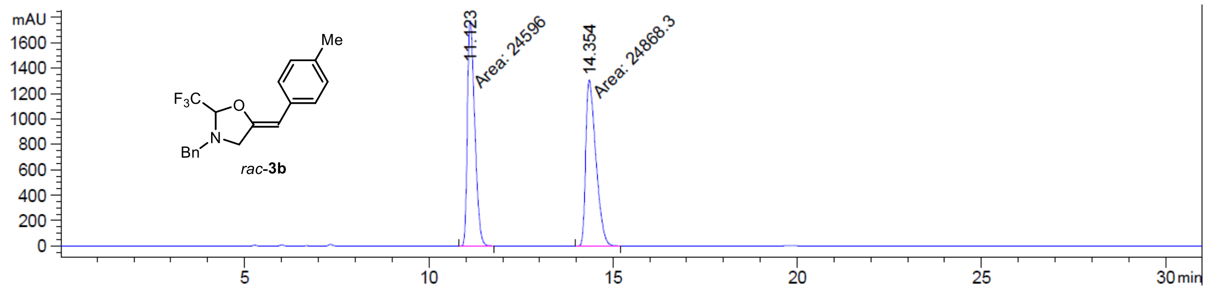

Signal 1: DAD1 A, Sig=254,4 Ref=360,100

Peak #	RetTime [min]	Type	Width [min]	Area [mAU*s]	Height [mAU]	Area %
1	11.123	MM	0.2314	2.45960e4	1771.71655	49.7247
2	14.354	MM	0.3174	2.48683e4	1305.88171	50.2753

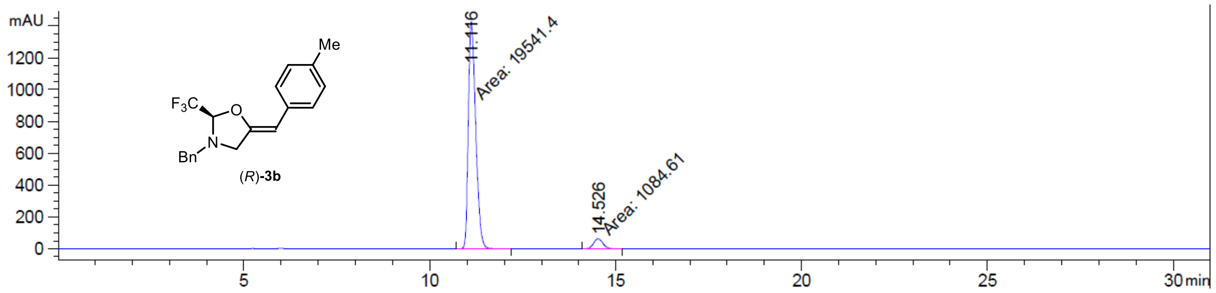

Signal 1: DAD1 A, Sig=254,4 Ref=360,100

Peak #	RetTime [min]	Type	Width [min]	Area [mAU*s]	Height [mAU]	Area %
1	11.116	MM	0.2285	1.95414e4	1425.06104	94.7415
2	14.526	MM	0.2889	1084.60938	62.56993	5.2585

Chiral HPLC Daicel Chiralpak IB N-5 column: 99:1 hexane/IPA, flow rate 1 mL/min, λ = 254 nm

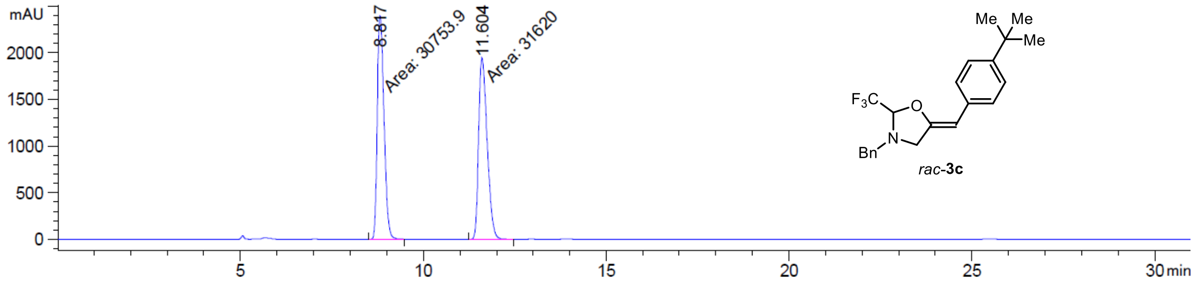

Signal 1: DAD1 A, Sig=254,4 Ref=360,100

Peak #	RetTime [min]	Type	Width [min]	Area [mAU*s]	Height [mAU]	Area %
1	8.817	MM	0.2139	3.07539e4	2396.16968	49.3058
2	11.604	MM	0.2702	3.16200e4	1950.65271	50.6942

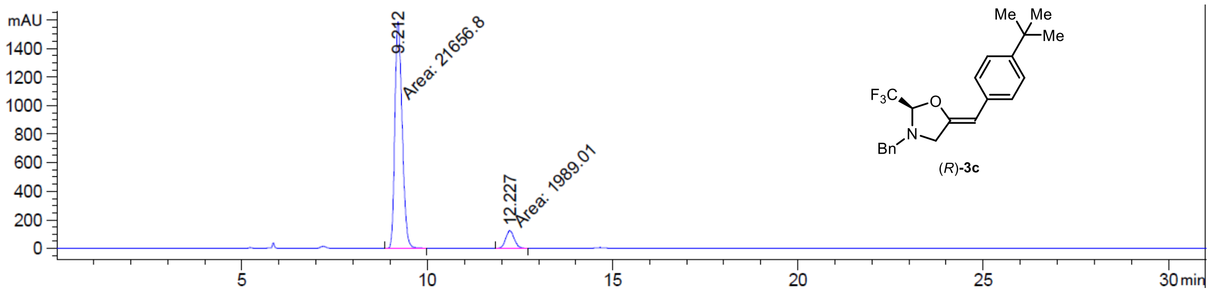

Signal 1: DAD1 A, Sig=254,4 Ref=360,100

Peak #	RetTime [min]	Type	Width [min]	Area [mAU*s]	Height [mAU]	Area %
1	9.212	MM	0.2277	2.16568e4	1585.24792	91.5883
2	12.227	MM	0.2692	1989.00696	123.15968	8.4117

Chiral HPLC Daicel Chiralpak IB N-5 column: 95:5 hexane/IPA, flow rate 1 mL/min, λ = 254 nm

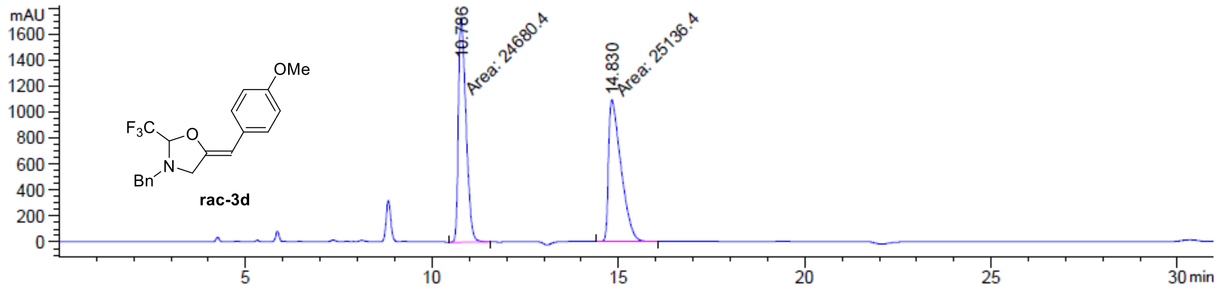

Signal 1: DAD1 A, Sig=254,4 Ref=360,100

Peak #	RetTime [min]	Type	Width [min]	Area [mAU*s]	Height [mAU]	Area %
1	10.786	MM	0.2376	2.46804e4	1731.27991	49.5423
2	14.830	MM	0.3840	2.51364e4	1090.97107	50.4577

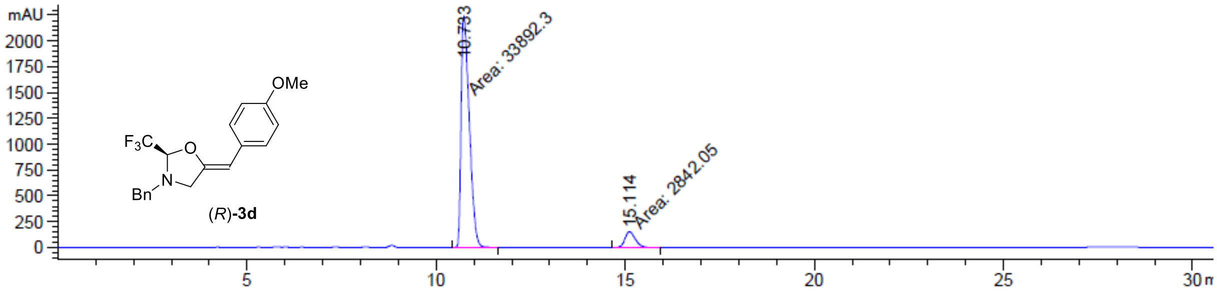

Signal 1: DAD1 A, Sig=254,4 Ref=360,100

Peak #	RetTime [min]	Type	Width [min]	Area [mAU*s]	Height [mAU]	Area %
1	10.733	MM	0.2532	3.38923e4	2230.70264	92.2632
2	15.114	MM	0.3123	2842.05029	151.65312	7.7368

Chiral HPLC Daicel Chiralpak IB N-5 column: 99:1 hexane/IPA, flow rate 1 mL/min, λ = 254 nm

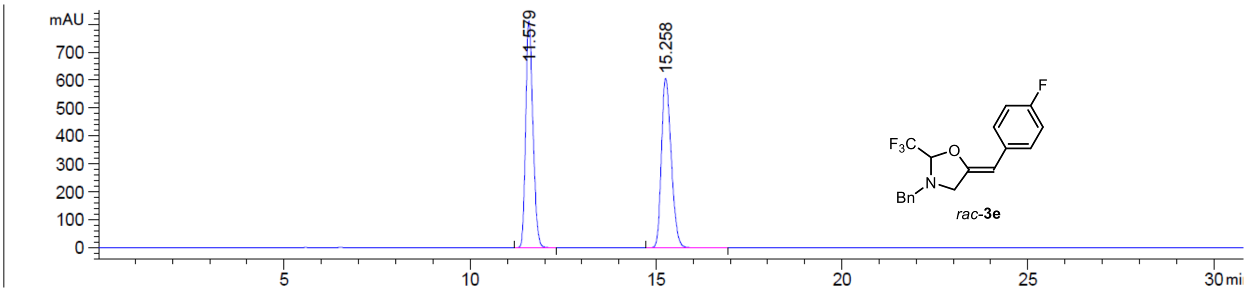

Signal 1: DAD1 A, Sig=254,4 Ref=360,100

Peak #	RetTime [min]	Type	Width [min]	Area [mAU*s]	Height [mAU]	Area %
1	11.579	BB	0.2127	1.10505e4	812.13593	49.9285
2	15.258	BB	0.2830	1.10822e4	607.11145	50.0715

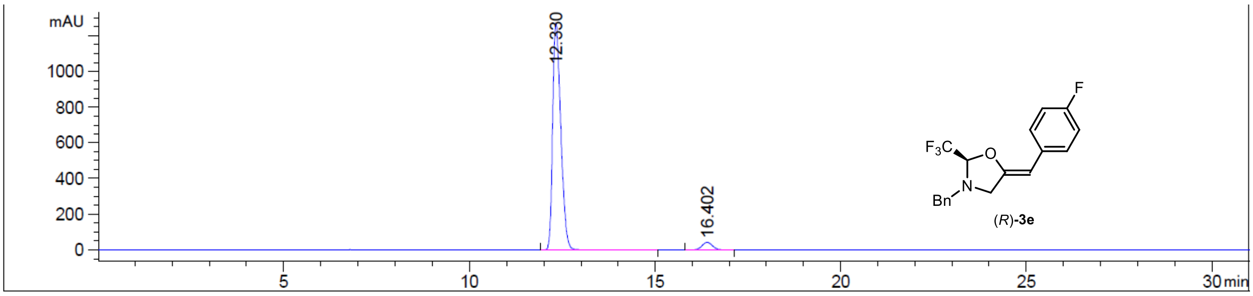

Signal 1: DAD1 A, Sig=254,4 Ref=360,100

Peak #	RetTime [min]	Type	Width [min]	Area [mAU*s]	Height [mAU]	Area %
1	12.330	BB	0.2327	1.92389e4	1270.44849	95.9878
2	16.402	BB	0.2968	804.17584	42.11253	4.0122

Chiral HPLC Daicel Chiralpak IB N-5 column: 99:1 hexane/IPA, flow rate 1 mL/min, λ = 254 nm

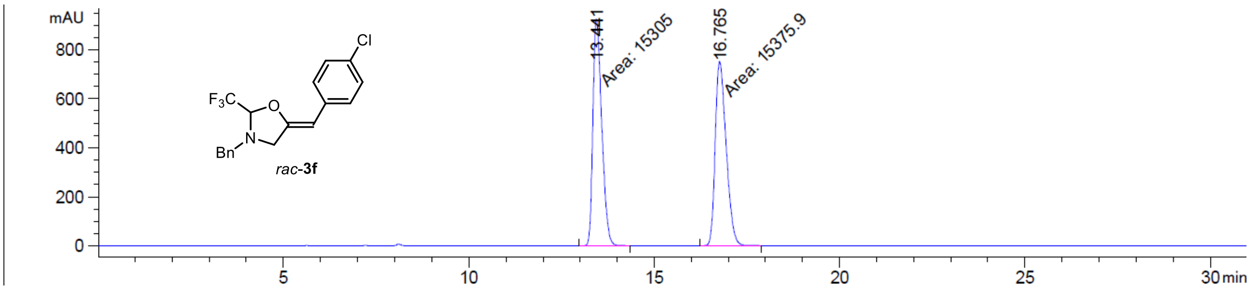

Signal 1: DAD1 A, Sig=254,4 Ref=360,100

Peak #	RetTime [min]	Type	Width [min]	Area [mAU*s]	Height [mAU]	Area %
1	13.441	MM	0.2761	1.53050e4	923.77667	49.8845
2	16.765	MM	0.3408	1.53759e4	752.04340	50.1155

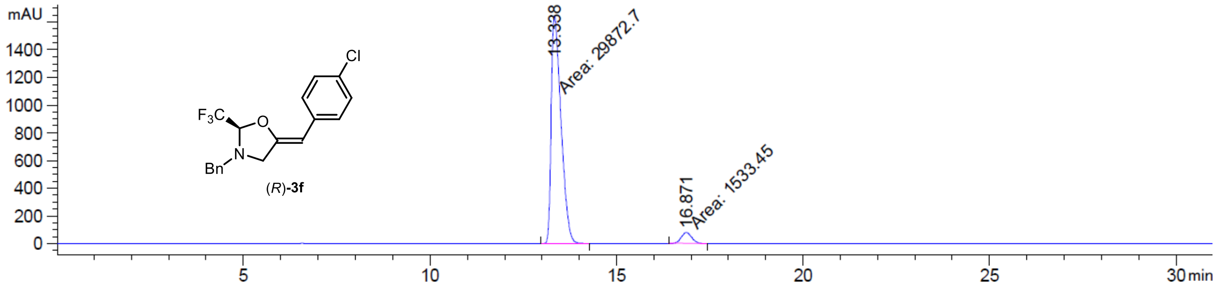

Signal 1: DAD1 A, Sig=254,4 Ref=360,100

Peak #	RetTime [min]	Type	Width [min]	Area [mAU*s]	Height [mAU]	Area %
1	13.338	MM	0.3025	2.98727e4	1645.81787	95.1173
2	16.871	MM	0.3246	1533.45422	78.74245	4.8827

Chiral HPLC Daicel Chiralpak IB N-5 column: 99:1 hexane/IPA, flow rate 1 mL/min, λ = 254 nm

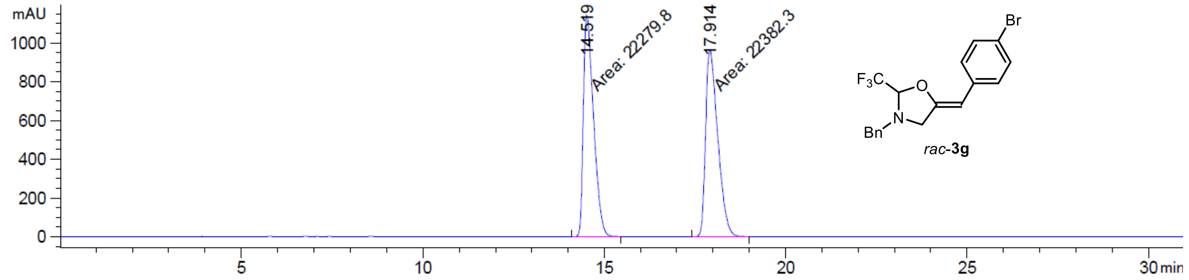

Signal 1: DAD1 A, Sig=254,4 Ref=360,100

Peak #	RetTime [min]	Type	Width [min]	Area [mAU*s]	Height [mAU]	Area %
1	14.519	MM	0.3249	2.22798e4	1142.76660	49.8853
2	17.914	MM	0.3875	2.23823e4	962.68378	50.1147

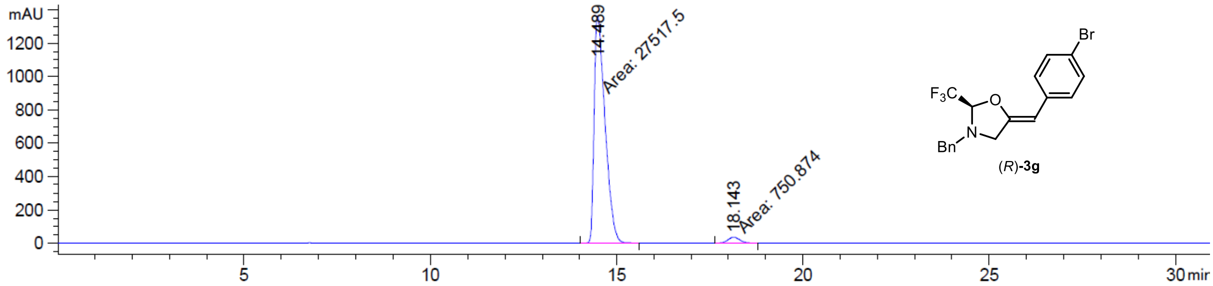

Signal 1: DAD1 A, Sig=254,4 Ref=360,100

Peak #	RetTime [min]	Type	Width [min]	Area [mAU*s]	Height [mAU]	Area %
1	14.489	MM	0.3363	2.75175e4	1363.69739	97.3438
2	18.143	MM	0.3569	750.87433	35.06561	2.6562

Chiral HPLC Daicel Chiralpak IB N-5 column: 99:1 hexane/IPA, flow rate 1 mL/min, λ = 254 nm

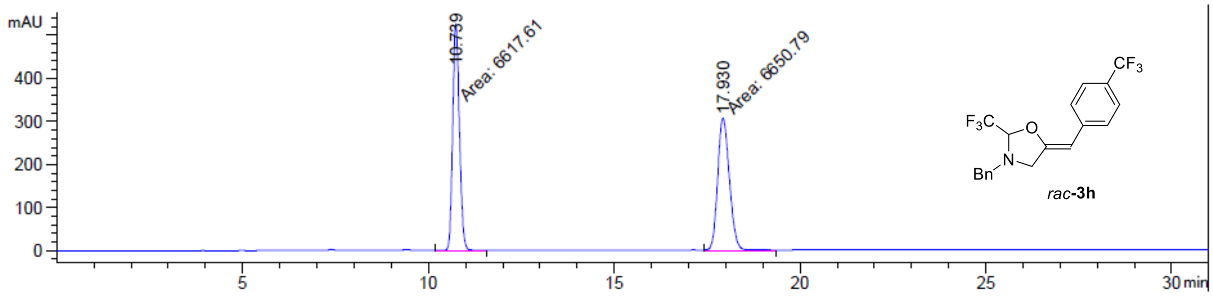


```
Signal 1: DAD1 A, Sig=254,4 Ref=360,100
```

Peak #	RetTime [min]	Type	Width [min]	Area [mAU*s]	Height [mAU]	Area %
1	10.739	MM	0.2108	6617.60645	523.32312	49.8749
2	17.930	MM	0.3617	6650.79199	306.46542	50.1251

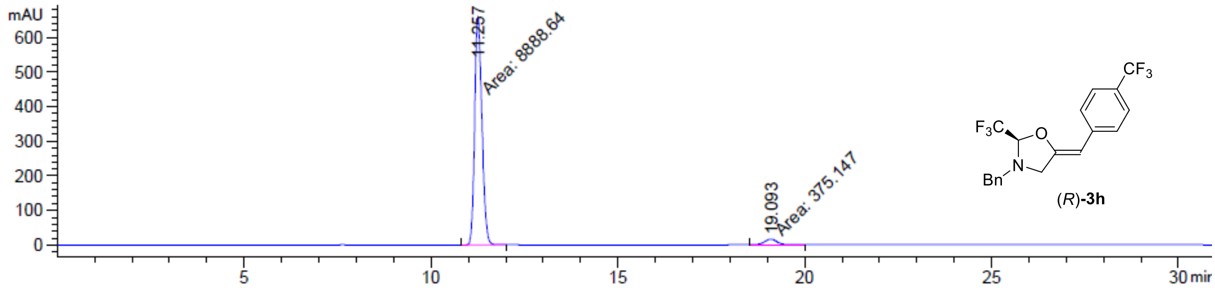


```
Signal 1: DAD1 A, Sig=254,4 Ref=360,100
```

Peak #	RetTime [min]	Type	Width [min]	Area [mAU*s]	Height [mAU]	Area %
1	11.257	MM	0.2252	8888.64355	657.96155	95.9504
2	19.093	MM	0.3858	375.14651	16.20700	4.0496

Chiral HPLC Daicel Chiralpak IB N-5 column: 99:1 hexane/IPA, flow rate 1 mL/min, λ = 254 nm

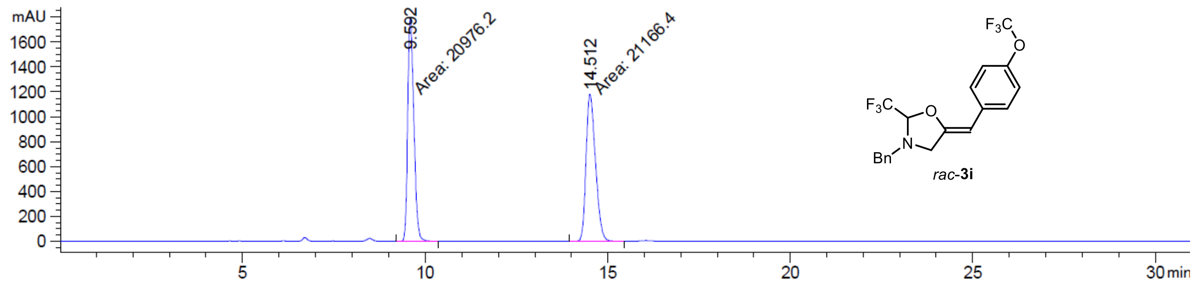

Signal 1: DAD1 A, Sig=254,4 Ref=360,100

Peak #	RetTime [min]	Type	Width [min]	Area [mAU*s]	Height [mAU]	Area %
1	9.592	MM	0.1948	2.09762e4	1795.05945	49.7743
2	14.512	MM	0.2986	2.11664e4	1181.28857	50.2257

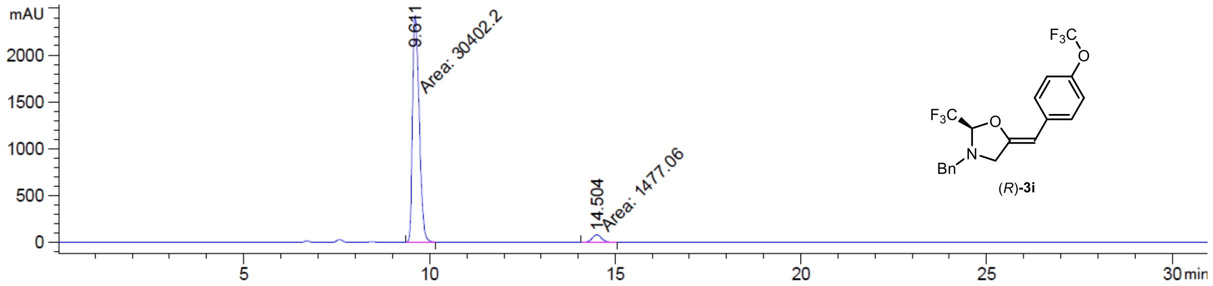

Signal 1: DAD1 A, Sig=254,4 Ref=360,100

Peak #	RetTime [min]	Type	Width [min]	Area [mAU*s]	Height [mAU]	Area %
1	9.611	MM	0.2091	3.04022e4	2423.45947	95.3667
2	14.504	MM	0.3019	1477.05957	81.53121	4.6333

Chiral HPLC Daicel Chiralpak IA column: 95:5 hexane/IPA, flow rate 1 mL/min, λ = 254 nm

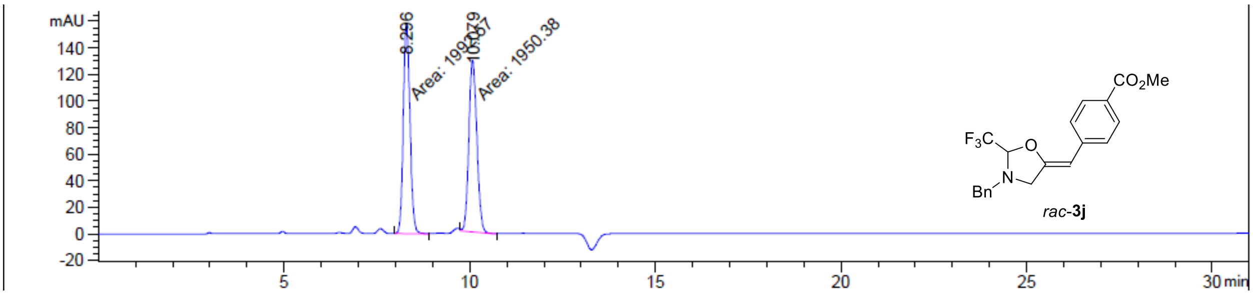

Signal 1: DAD1 A, Sig=254,4 Ref=360,100

Peak #	RetTime [min]	Type	Width [min]	Area [mAU*s]	Height [mAU]	Area %
1	8.296	MM	0.2095	1992.56763	158.55391	50.5349
2	10.079	MM	0.2515	1950.38452	129.24408	49.4651

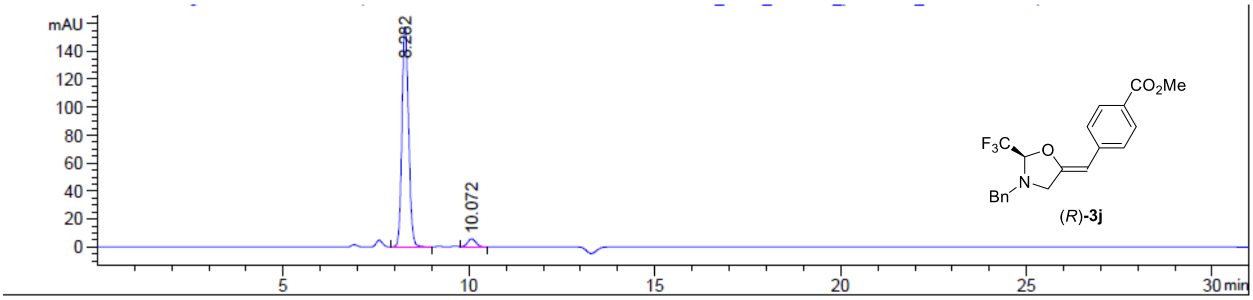

Signal 1: DAD1 A, Sig=254,4 Ref=360,100

Peak #	RetTime [min]	Type	Width [min]	Area [mAU*s]	Height [mAU]	Area %
1	8.282	BB	0.1958	1965.86572	157.32161	95.9784
2	10.072	BB	0.2341	82.37238	5.51916	4.0216

Chiral HPLC Daicel Chiralpak IB N-5 column: 95:5 hexane/IPA, flow rate 1 mL/min, λ = 214 nm

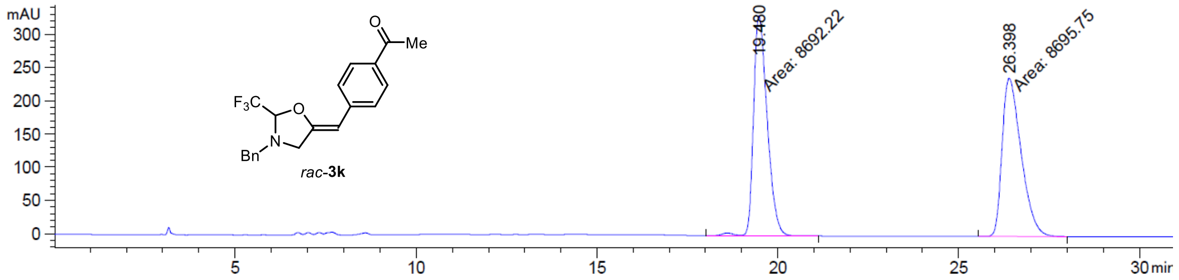

Signal 3: DAD1 C, Sig=214,4 Ref=360,100

Peak #	RetTime [min]	Type	Width [min]	Area [mAU*s]	Height [mAU]	Area %
1	19.480	MM	0.4378	8692.21777	330.92984	49.9898
2	26.398	MM	0.6097	8695.74805	237.68831	50.0102

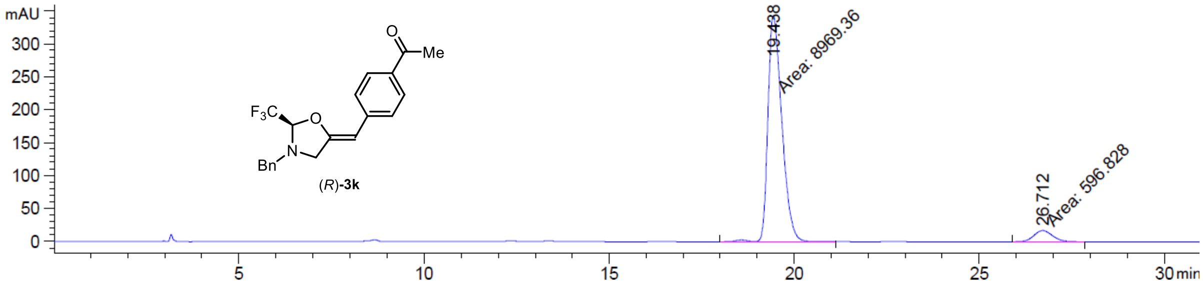

Signal 3: DAD1 C, Sig=214,4 Ref=360,100

Peak #	RetTime [min]	Type	Width [min]	Area [mAU*s]	Height [mAU]	Area %
1	19.438	MM	0.4354	8969.35938	343.30438	93.7611
2	26.712	MM	0.5858	596.82806	16.97925	6.2389

Chiral HPLC Daicel Chiralpak IA column: 95:5 hexane/IPA, flow rate 1 mL/min, λ = 254 nm

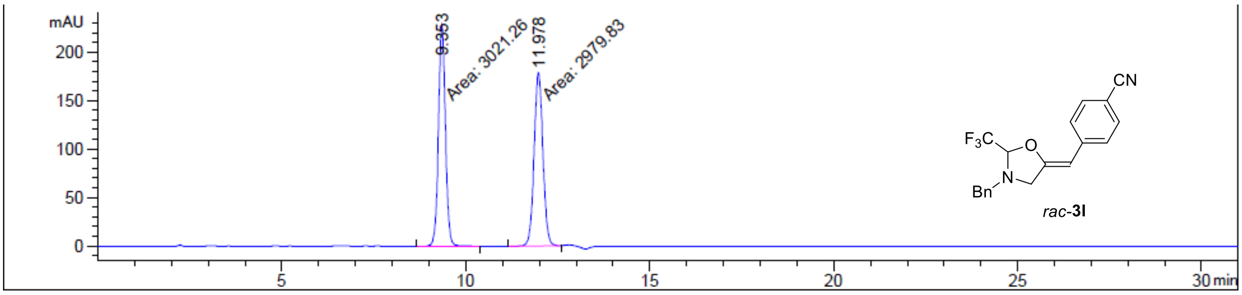

Signal 1: DAD1 A, Sig=254,4 Ref=360,100

Peak #	RetTime [min]	Type	Width [min]	Area [mAU*s]	Height [mAU]	Area %
1	9.353	MM	0.2205	3021.25659	228.40935	50.3452
2	11.978	MM	0.2786	2979.82617	178.24309	49.6548

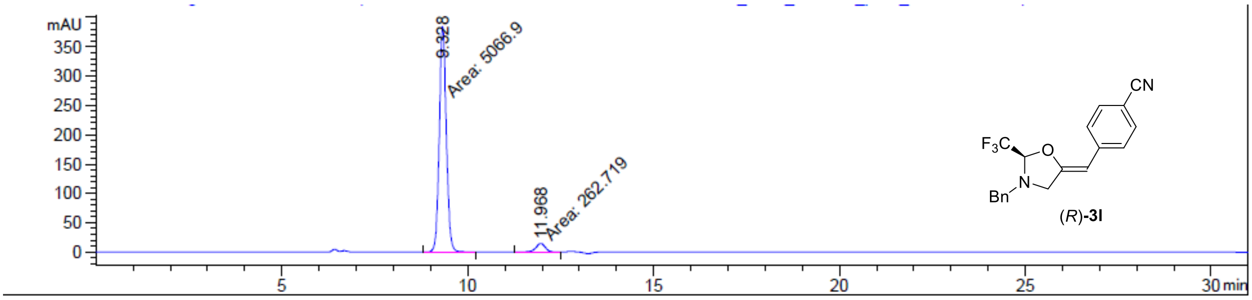

Signal 1: DAD1 A, Sig=254,4 Ref=360,100

Peak #	RetTime [min]	Type	Width [min]	Area [mAU*s]	Height [mAU]	Area %
1	9.328	MM	0.2203	5066.90479	383.38580	95.0706
2	11.968	MM	0.2960	262.71887	14.79262	4.9294

Chiral HPLC Daicel Chiralpak IB N-5 column: 99:1 hexane/IPA, flow rate 1 mL/min, λ = 254 nm

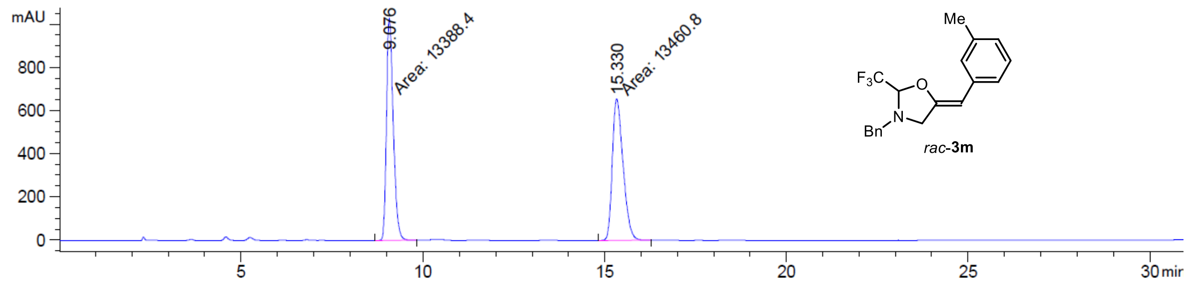

Signal 1: DAD1 A, Sig=254,4 Ref=360,100

Peak #	RetTime [min]	Type	Width [min]	Area [mAU*s]	Height [mAU]	Area %
1	9.076	MM	0.2169	1.33884e4	1028.96338	49.8652
2	15.330	MM	0.3414	1.34608e4	657.15070	50.1348

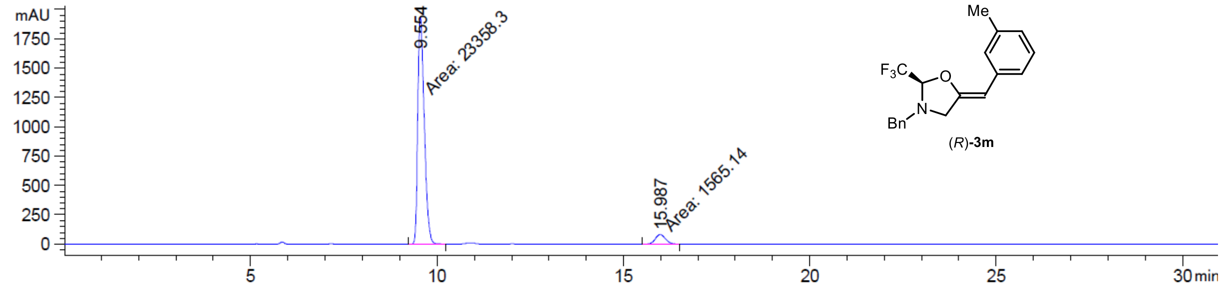

Signal 1: DAD1 A, Sig=254,4 Ref=360,100

Peak #	RetTime [min]	Type	Width [min]	Area [mAU*s]	Height [mAU]	Area %
1	9.554	MM	0.2009	2.33583e4	1938.15588	93.7202
2	15.987	MM	0.3169	1565.13513	82.30818	6.2798

Chiral HPLC Daicel Chiralpak IB N-5 column: 99:1 hexane/IPA, flow rate 1 mL/min, λ = 254 nm

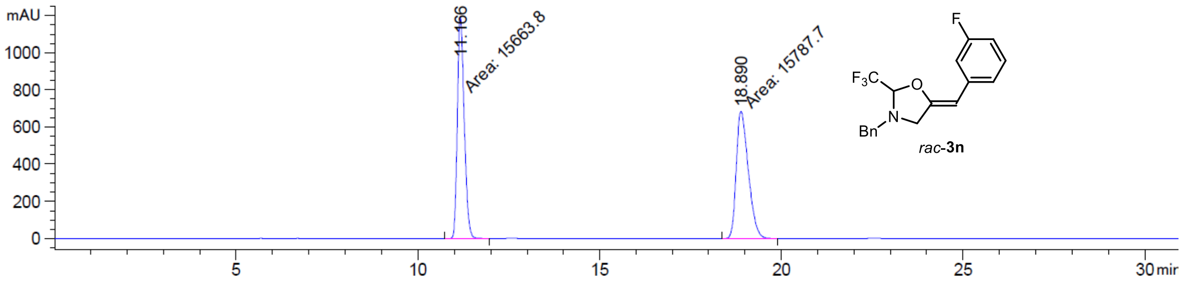

Signal 1: DAD1 A, Sig=254,4 Ref=360,100

Peak #	RetTime [min]	Type	Width [min]	Area [mAU*s]	Height [mAU]	Area %
1	11.166	MM	0.2188	1.56638e4	1193.26343	49.8030
2	18.890	MM	0.3849	1.57877e4	683.56555	50.1970

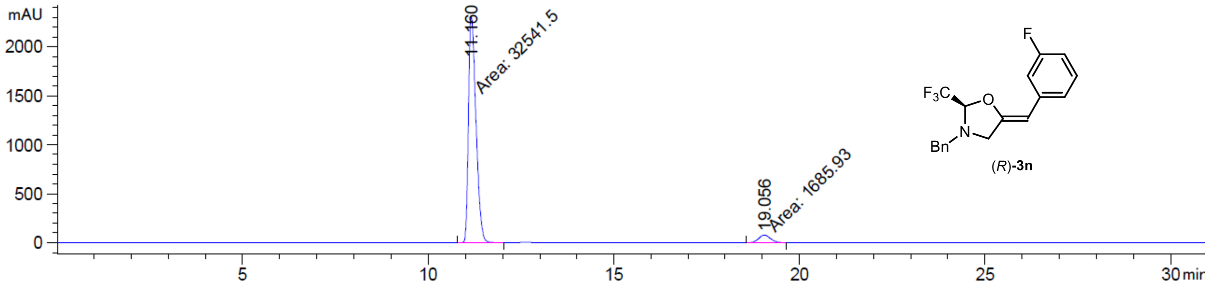

Signal 1: DAD1 A, Sig=254,4 Ref=360,100

Peak #	RetTime [min]	Type	Width [min]	Area [mAU*s]	Height [mAU]	Area %
1	11.160	MM	0.2344	3.25415e4	2313.83179	95.0743
2	19.056	MM	0.3648	1685.93262	77.01691	4.9257

Chiral HPLC Daicel Chiralpak IB N-5 column: 99:1 hexane/IPA, flow rate 1 mL/min, λ = 254 nm

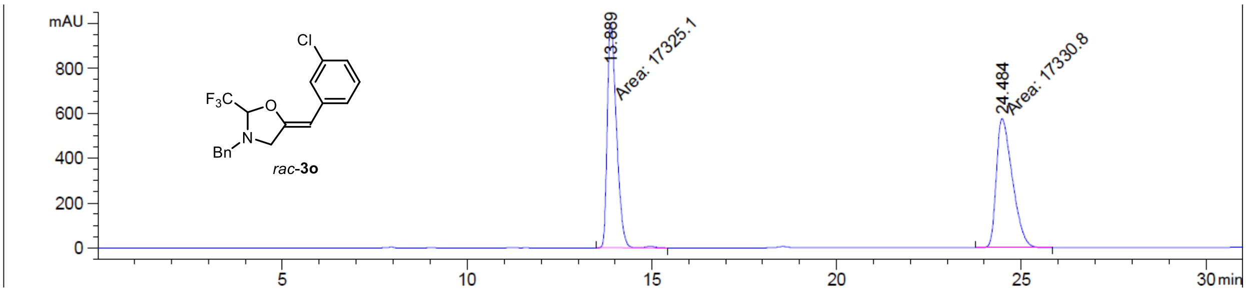

Signal 1: DAD1 A, Sig=254,4 Ref=360,100

Peak #	RetTime [min]	Type	Width [min]	Area [mAU*s]	Height [mAU]	Area %
1	13.889	MM	0.2886	1.73251e4	1000.61200	49.9917
2	24.484	MM	0.5024	1.73308e4	574.92786	50.0083

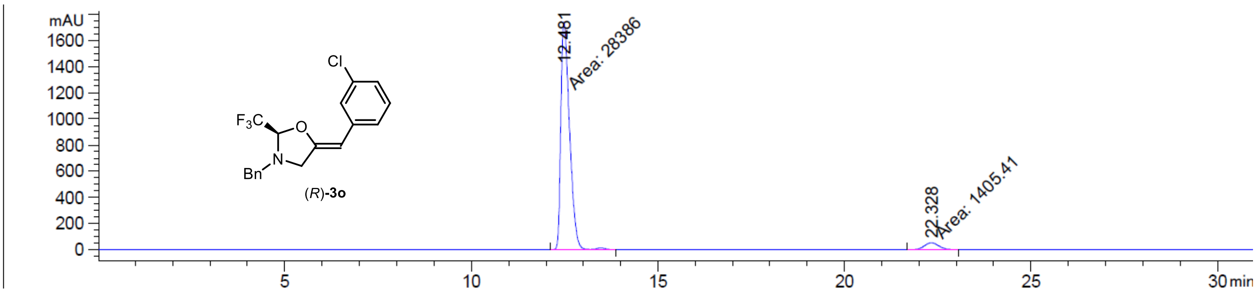

Signal 1: DAD1 A, Sig=254,4 Ref=360,100

Peak #	RetTime [min]	Type	Width [min]	Area [mAU*s]	Height [mAU]	Area %
1	12.481	MM	0.2716	2.83860e4	1741.63745	95.2825
2	22.328	MM	0.4488	1405.40979	52.18882	4.7175

Chiral HPLC Daicel Chiralpak IB N-5 column: 99:1 hexane/IPA, flow rate 1 mL/min, λ = 254 nm

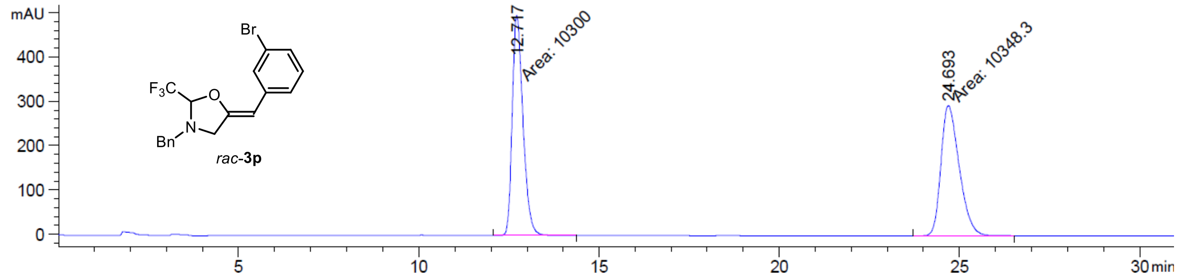

Signal 1: DAD1 A, Sig=254,4 Ref=360,100

Peak #	RetTime [min]	Type	Width [min]	Area [mAU*s]	Height [mAU]	Area %
1	12.717	MM	0.3458	1.03000e4	496.45453	49.8832
2	24.693	MM	0.5874	1.03483e4	293.64285	50.1168

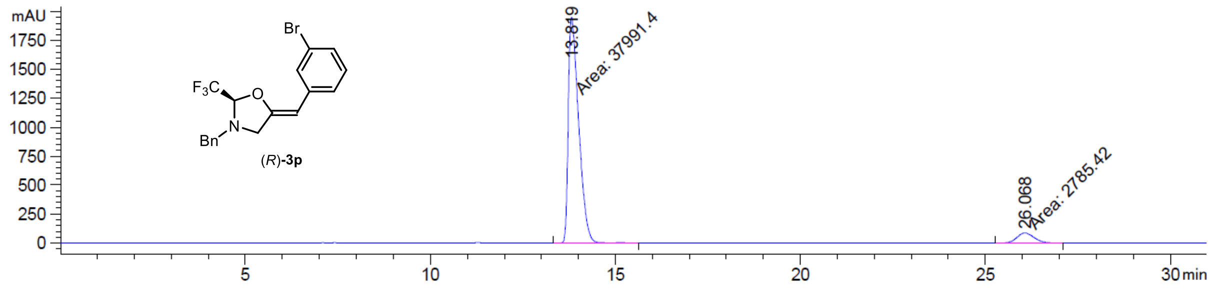

Signal 1: DAD1 A, Sig=254,4 Ref=360,100

Peak #	RetTime [min]	Type	Width [min]	Area [mAU*s]	Height [mAU]	Area %
1	13.819	MM	0.3249	3.79914e4	1948.92444	93.1691
2	26.068	MM	0.5393	2785.41504	86.08337	6.8309

Chiral HPLC Daicel Chiralpak IB N-5 column: 99:1 hexane/IPA, flow rate 1 mL/min, λ = 254 nm

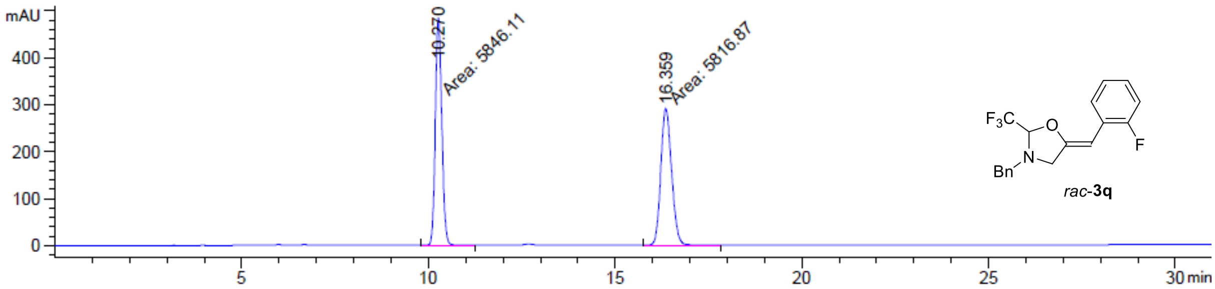

Signal 1: DAD1 A, Sig=254,4 Ref=360,100

Peak #	RetTime [min]	Type	Width [min]	Area [mAU*s]	Height [mAU]	Area %
1	10.270	MM	0.2012	5846.11475	484.25104	50.1254
2	16.359	MM	0.3336	5816.87305	290.60568	49.8746

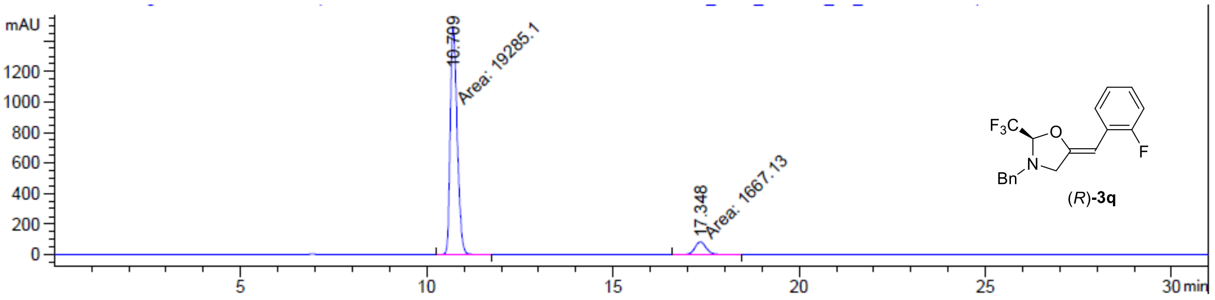

Signal 1: DAD1 A, Sig=254,4 Ref=360,100

Peak #	RetTime [min]	Type	Width [min]	Area [mAU*s]	Height [mAU]	Area %
1	10.709	MM	0.2156	1.92851e4	1490.61804	92.0432
2	17.348	MM	0.3380	1667.13147	82.21236	7.9568

Chiral HPLC Daicel Chiralpak IB N-5 column: 99:1 hexane/IPA, flow rate 1 mL/min, λ = 254 nm

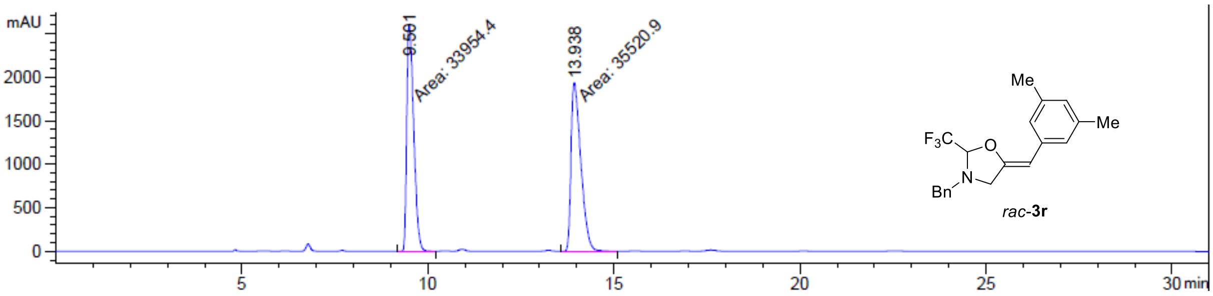

Signal 1: DAD1 A, Sig=254,4 Ref=360,100

Peak #	RetTime [min]	Type	Width [min]	Area [mAU*s]	Height [mAU]	Area %
1	9.501	MM	0.2173	3.39544e4	2603.97998	48.8726
2	13.938	MM	0.3060	3.55209e4	1934.65637	51.1274

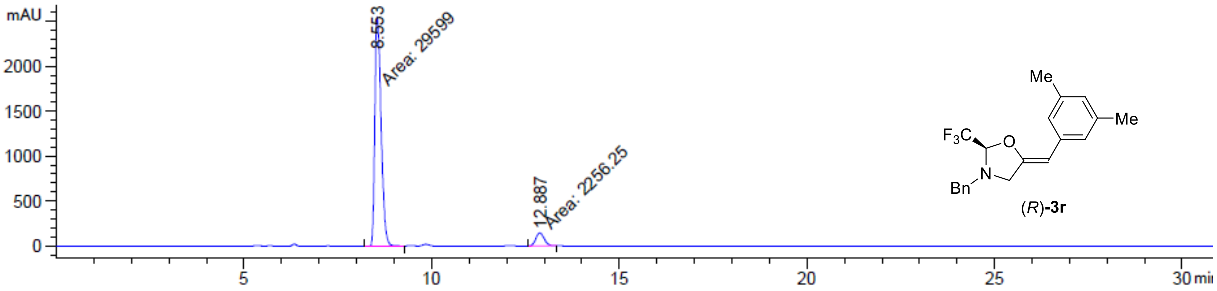

Signal 1: DAD1 A, Sig=254,4 Ref=360,100

Peak #	RetTime [min]	Type	Width [min]	Area [mAU*s]	Height [mAU]	Area %
1	8.553	MM	0.1941	2.95990e4	2541.51367	92.9172
2	12.887	MM	0.2599	2256.25171	144.68677	7.0828

Chiral HPLC Daicel Chiralpak IB N-5 column: 99:1 hexane/IPA, flow rate 1 mL/min, λ = 254 nm

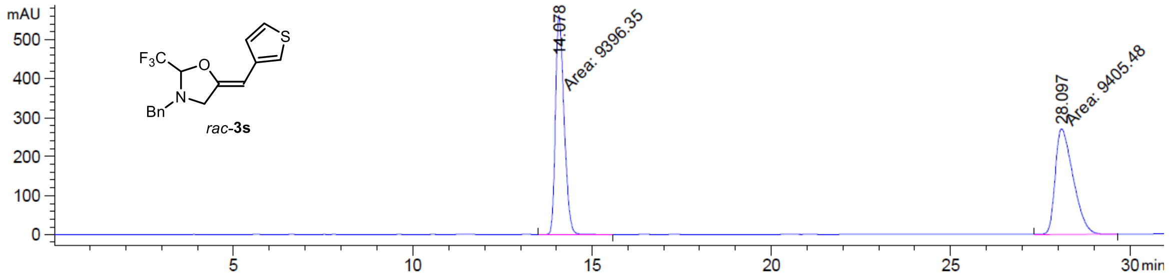

Signal 1: DAD1 A, Sig=254,4 Ref=360,100

Peak #	RetTime [min]	Type	Width [min]	Area [mAU*s]	Height [mAU]	Area %
1	14.078	MM	0.2789	9396.34570	561.53802	49.9757
2	28.097	MM	0.5797	9405.47949	270.42789	50.0243

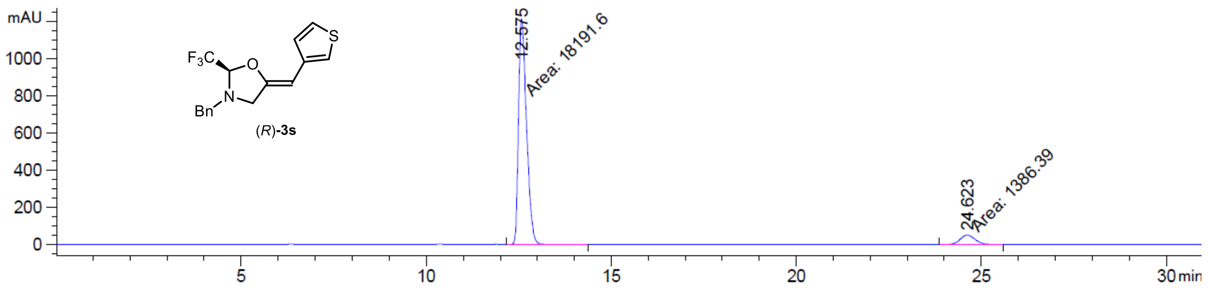

Signal 1: DAD1 A, Sig=254,4 Ref=360,100

Peak #	RetTime [min]	Type	Width [min]	Area [mAU*s]	Height [mAU]	Area %
1	12.575	MM	0.2498	1.81916e4	1213.70837	92.9186
2	24.623	MM	0.4625	1386.38916	49.96437	7.0814

Chiral HPLC Daicel Chiralpak IB N-5 column: 80:20 hexane/IPA, flow rate 1 mL/min, λ = 254 nm

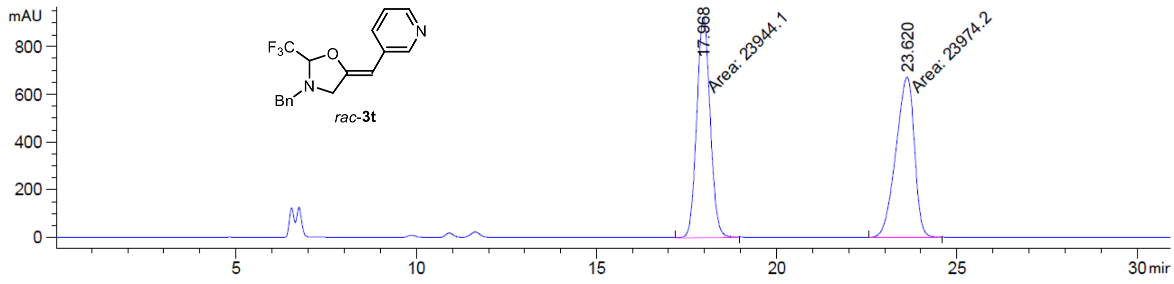

Signal 1: DAD1 A, Sig=254,4 Ref=360,100

Peak #	RetTime [min]	Type	Width [min]	Area [mAU*s]	Height [mAU]	Area %
1	17.968	MM	0.4326	2.39441e4	922.59119	49.9686
2	23.620	MM	0.5939	2.39742e4	672.78363	50.0314

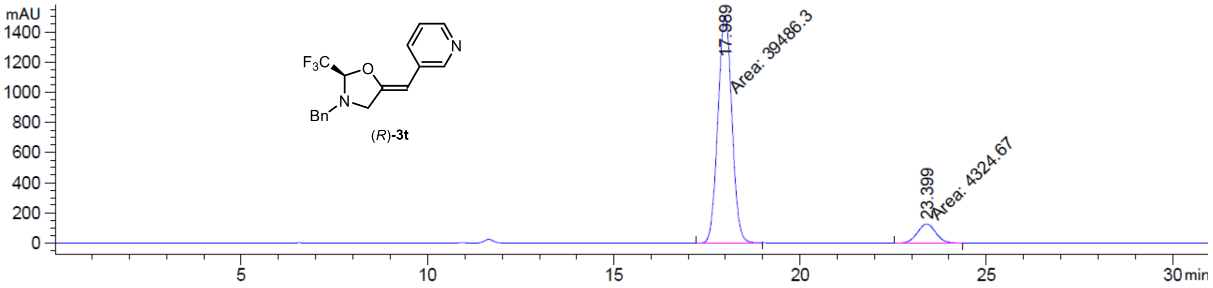

Signal 1: DAD1 A, Sig=254,4 Ref=360,100

Peak #	RetTime [min]	Type	Width [min]	Area [mAU*s]	Height [mAU]	Area %
1	17.989	MM	0.4368	3.94863e4	1506.74780	90.1288
2	23.399	MM	0.5700	4324.67334	126.45943	9.8712

Chiral HPLC Daicel Chiralpak IB N-5 column: 90:10 hexane/IPA, flow rate 1 mL/min, λ = 254 nm

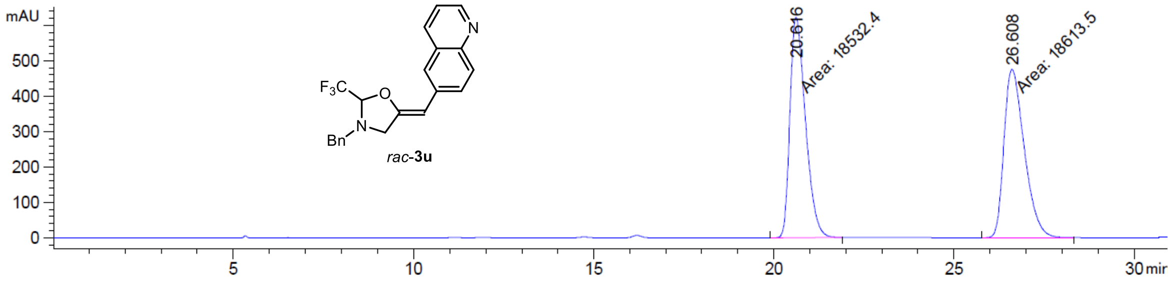

Signal 1: DAD1 A, Sig=254,4 Ref=360,100

Peak #	RetTime [min]	Type	Width [min]	Area [mAU*s]	Height [mAU]	Area %
1	20.616	MM	0.4975	1.85324e4	620.85510	49.8908
2	26.608	MM	0.6527	1.86135e4	475.32925	50.1092

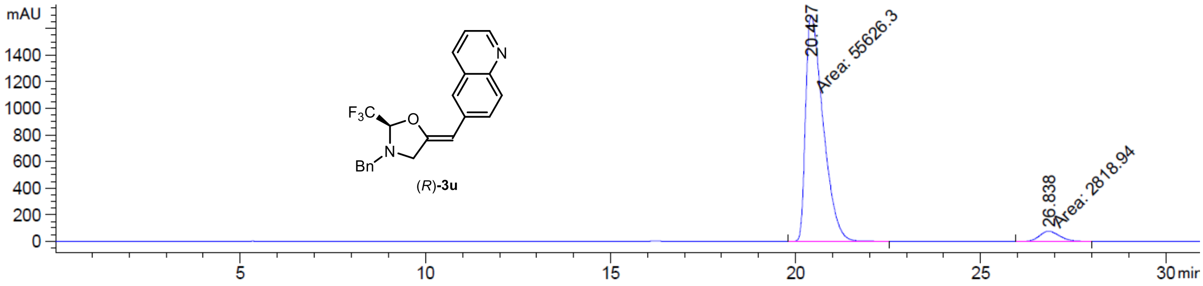

Signal 1: DAD1 A, Sig=254,4 Ref=360,100

Peak #	RetTime [min]	Type	Width [min]	Area [mAU*s]	Height [mAU]	Area %
1	20.427	MM	0.5467	5.56263e4	1695.74573	95.1768
2	26.838	MM	0.6202	2818.94067	75.75125	4.8232

Chiral HPLC Daicel Chiralpak IB N-5 column: 99:1 hexane/IPA, flow rate 1 mL/min, λ = 214 nm

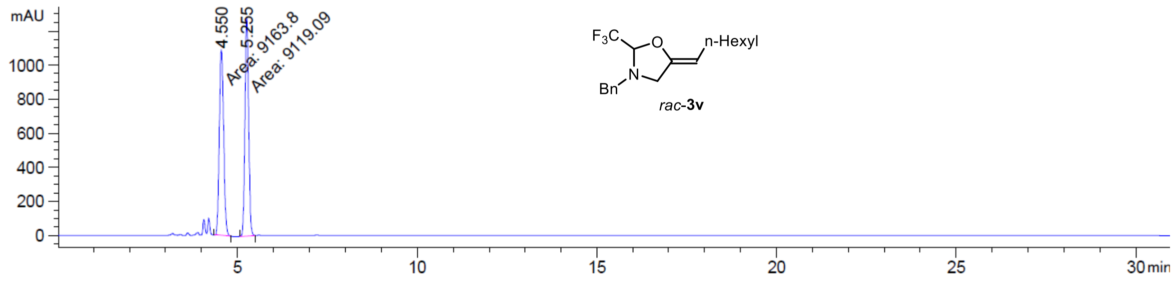

Signal 3: DAD1 C, Sig=214,4 Ref=360,100

Peak #	RetTime [min]	Type	Width [min]	Area [mAU*s]	Height [mAU]	Area %
1	4.550	MM	0.1407	9163.80176	1085.68994	50.1223
2	5.255	MM	0.1183	9119.09375	1285.23889	49.8777

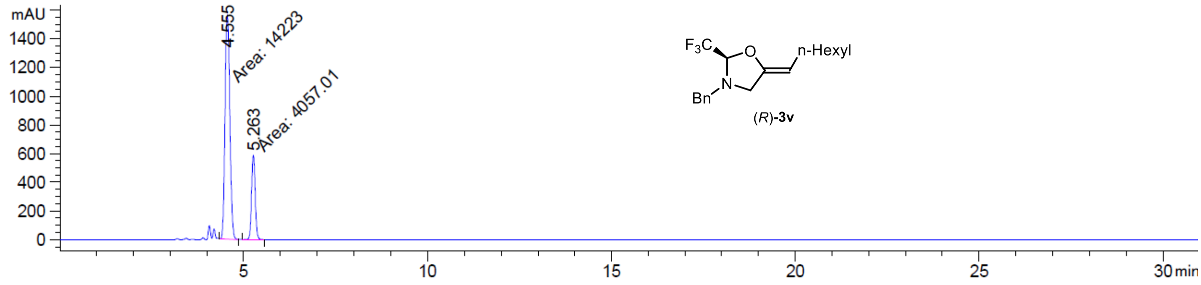

Signal 3: DAD1 C, Sig=214,4 Ref=360,100

Peak #	RetTime [min]	Type	Width [min]	Area [mAU*s]	Height [mAU]	Area %
1	4.555	MM	0.1524	1.42230e4	1555.19885	77.8063
2	5.263	MM	0.1152	4057.00537	587.09900	22.1937

Chiral HPLC Daicel Chiralpak IB N-5 column: 99:1 hexane/IPA, flow rate 1 mL/min, λ = 214 nm

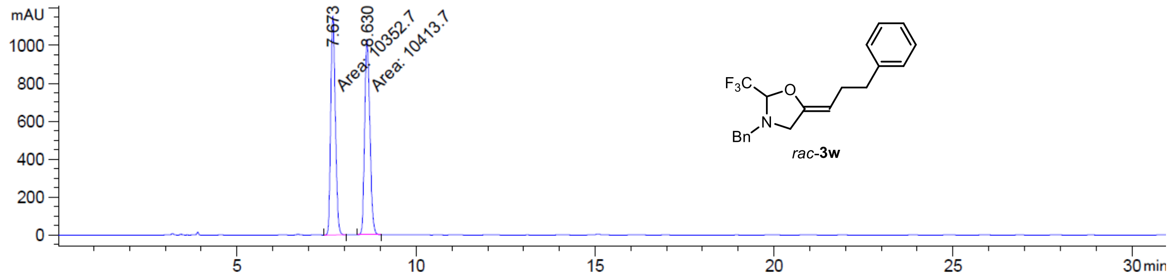

Signal 3: DAD1 C, Sig=214,4 Ref=360,100

Peak #	RetTime [min]	Type	Width [min]	Area [mAU*s]	Height [mAU]	Area %
1	7.673	MM	0.1495	1.03527e4	1154.22253	49.8529
2	8.630	MM	0.1684	1.04137e4	1030.36694	50.1471

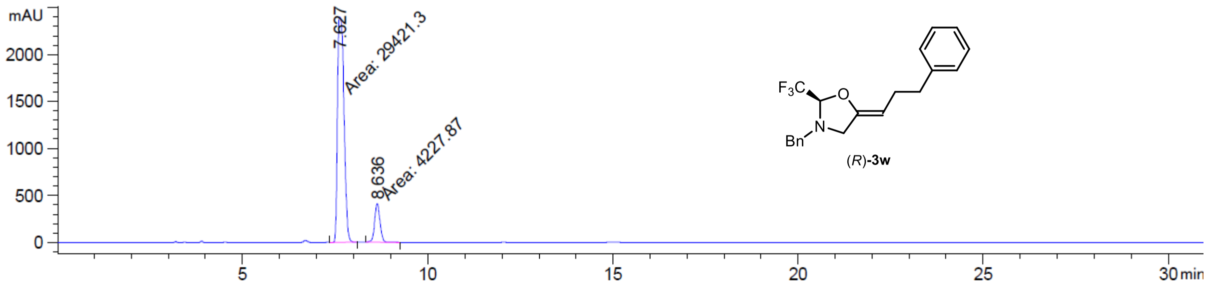

Signal 3: DAD1 C, Sig=214,4 Ref=360,100

Peak #	RetTime [min]	Type	Width [min]	Area [mAU*s]	Height [mAU]	Area %
1	7.627	MM	0.2044	2.94213e4	2398.91064	87.4354
2	8.636	MM	0.1718	4227.87012	410.09567	12.5646

HPLC Spectra for Hydrogenation of enantioenriched trisubstituted olefins

Chiral HPLC Daicel Chiralpak IA column: 95:5 hexane/IPA, flow rate 1 mL/min, λ = 210 nm

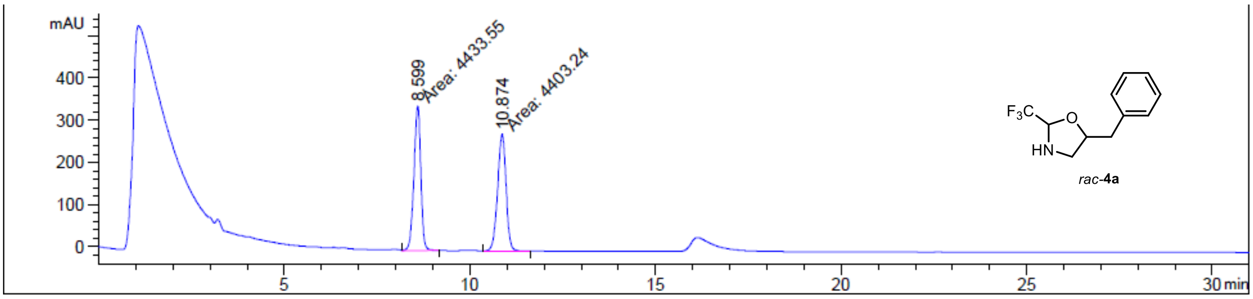

Signal 2: DAD1 B, Sig=210,4 Ref=360,100

Peak #	RetTime [min]	Type	Width [min]	Area [mAU*s]	Height [mAU]	Area %
1	8.599	MM	0.2156	4433.54688	342.71069	50.1715
2	10.874	MM	0.2644	4403.24463	277.55420	49.8285

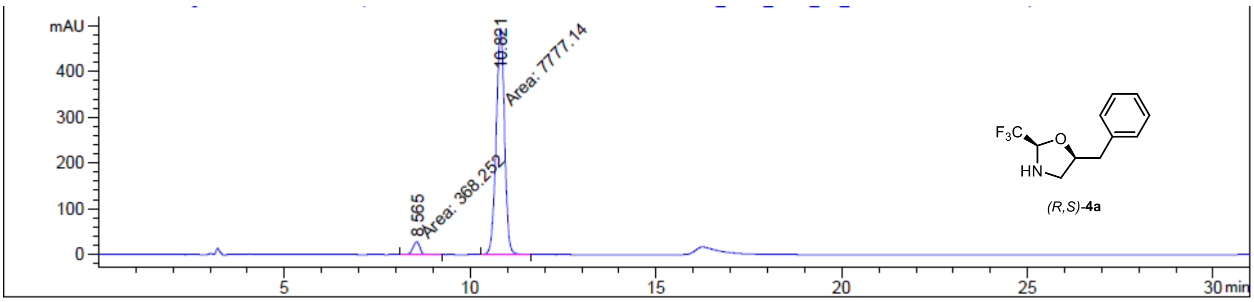

Signal 2: DAD1 B, Sig=210,4 Ref=360,100

Peak #	RetTime [min]	Type	Width [min]	Area [mAU*s]	Height [mAU]	Area %
1	8.565	MM	0.2148	368.25174	28.57014	4.5210
2	10.821	MM	0.2625	7777.13770	493.87759	95.4790

Chiral HPLC Daicel Chiralpak IA column: 95:5 hexane/IPA, flow rate 1 mL/min, λ = 214 nm

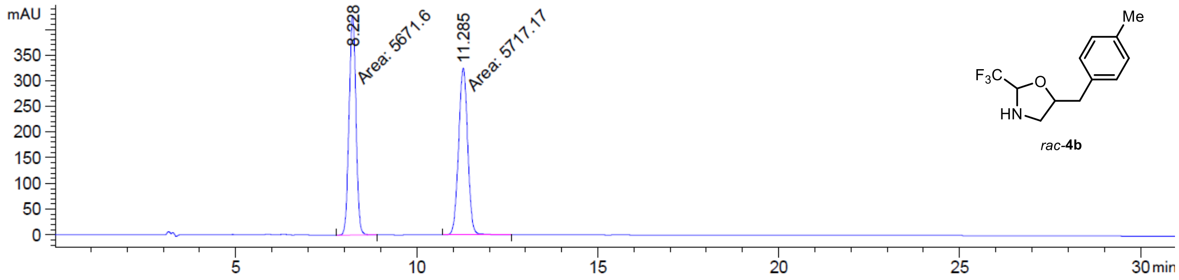

rac-**4b**

Signal 3: DAD1 C, Sig=214,4 Ref=360,100

Peak #	RetTime [min]	Type	Width [min]	Area [mAU*s]	Height [mAU]	Area %
1	8.228	MM	0.2208	5671.59668	428.05231	49.7999
2	11.285	MM	0.2929	5717.16748	325.35431	50.2001

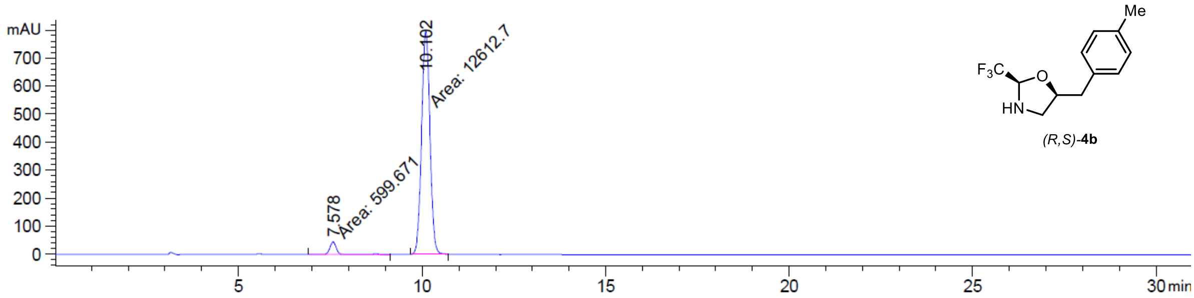

(R,S)-**4b**

Signal 3: DAD1 C, Sig=214,4 Ref=360,100

Peak #	RetTime [min]	Type	Width [min]	Area [mAU*s]	Height [mAU]	Area %
1	7.578	MM	0.2219	599.67145	45.03690	4.5387
2	10.102	MM	0.2632	1.26127e4	798.61334	95.4613

Chiral HPLC Daicel Chiralpak IA column: 95:5 hexane/IPA, flow rate 1 mL/min, λ = 214 nm

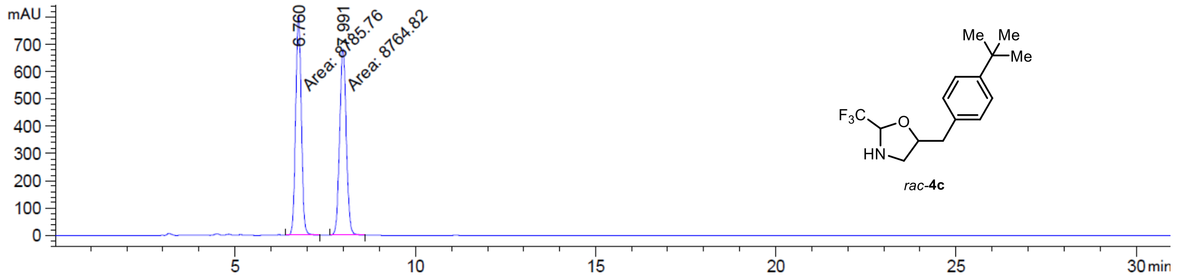

Signal 3: DAD1 C, Sig=214,4 Ref=360,100

Peak #	RetTime [min]	Type	Width [min]	Area [mAU*s]	Height [mAU]	Area %
1	6.760	MM	0.1817	8785.75586	805.70569	50.0596
2	7.991	MM	0.2139	8764.82324	682.97974	49.9404

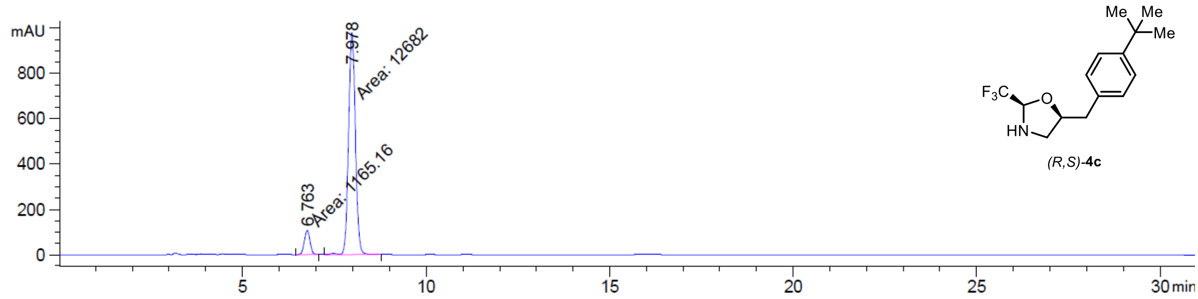

Signal 3: DAD1 C, Sig=214,4 Ref=360,100

Peak #	RetTime [min]	Type	Width [min]	Area [mAU*s]	Height [mAU]	Area %
1	6.763	MM	0.1807	1165.16150	107.48259	8.4144
2	7.978	MM	0.2156	1.26820e4	980.21130	91.5856

Chiral HPLC Daicel Chiralpak IA column: 95:5 hexane/IPA, flow rate 1 mL/min, λ = 210 nm

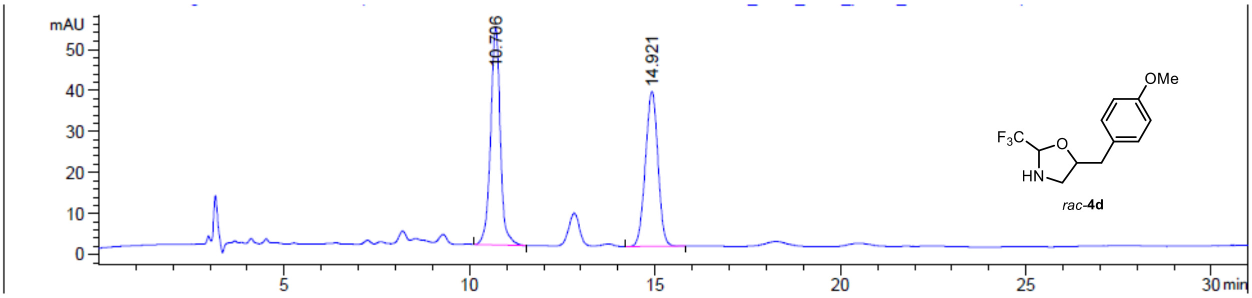

Signal 2: DAD1 B, Sig=210,4 Ref=360,100

Peak #	RetTime [min]	Type	Width [min]	Area [mAU*s]	Height [mAU]	Area %
1	10.706	BB	0.2779	964.28308	53.10142	51.9818
2	14.921	BB	0.3693	890.75610	37.75410	48.0182

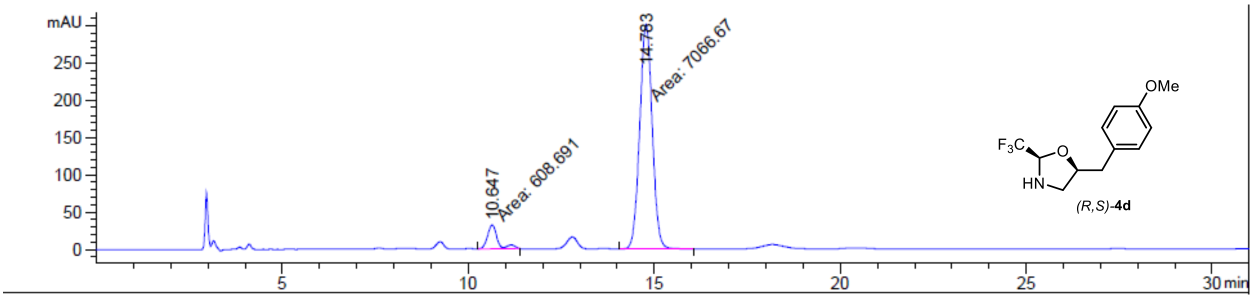

Signal 2: DAD1 B, Sig=210,4 Ref=360,100

Peak #	RetTime [min]	Type	Width [min]	Area [mAU*s]	Height [mAU]	Area %
1	10.647	MM	0.3158	608.69067	32.12711	7.9305
2	14.783	MM	0.3907	7066.66943	301.44839	92.0695

Chiral HPLC Daicel Chiralpak IA column: 95:5 hexane/IPA, flow rate 1 mL/min, λ = 254 nm

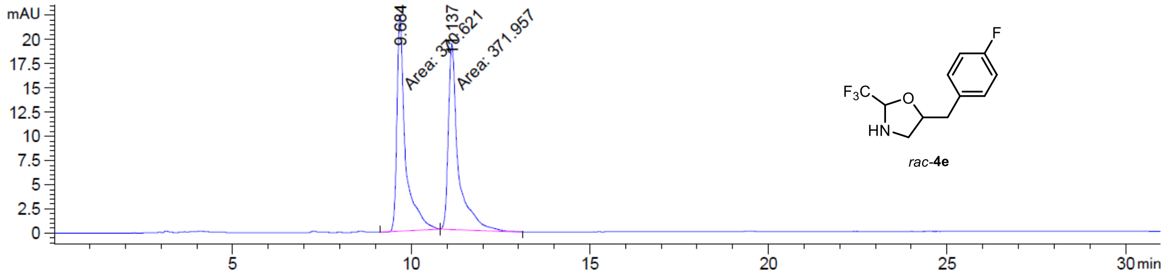

Signal 1: DAD1 A, Sig=254,4 Ref=360,100

Peak #	RetTime [min]	Type	Width [min]	Area [mAU*s]	Height [mAU]	Area %
1	9.684	MM	0.2763	370.62122	22.35606	49.9101
2	11.137	MM	0.3226	371.95660	19.21385	50.0899

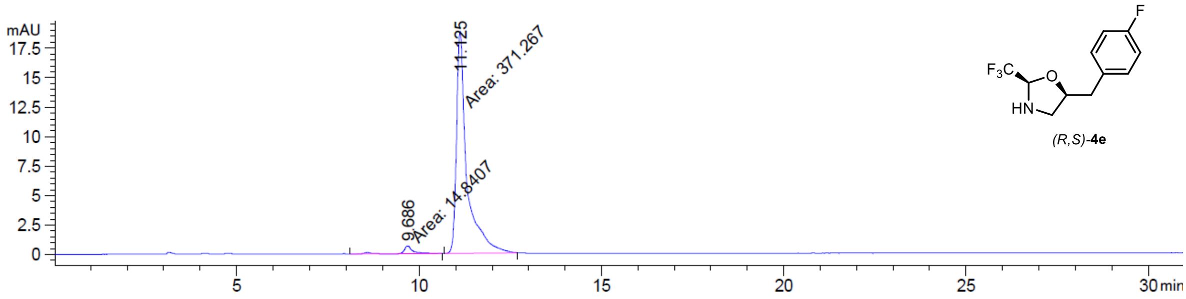

Signal 1: DAD1 A, Sig=254,4 Ref=360,100

Peak #	RetTime [min]	Type	Width [min]	Area [mAU*s]	Height [mAU]	Area %
1	9.686	MM	0.3700	14.84073	6.68512e-1	3.8437
2	11.125	MM	0.3290	371.26715	18.80507	96.1563

Chiral HPLC Daicel Chiralpak IA column: 95:5 hexane/IPA, flow rate 1 mL/min, λ = 210 nm

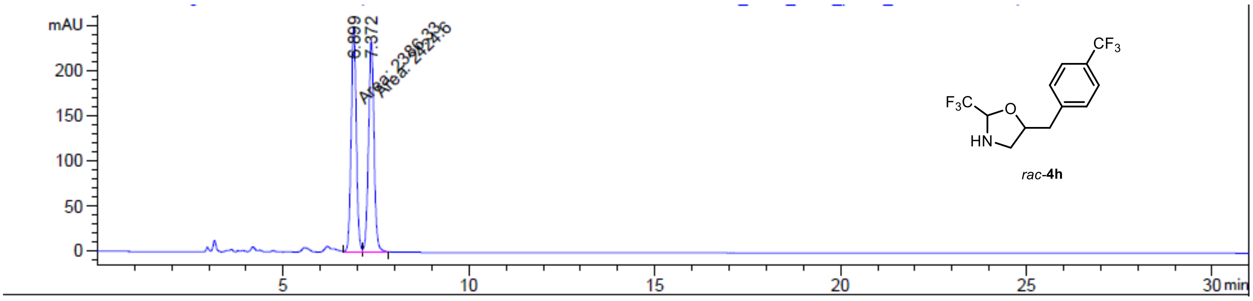

Signal 2: DAD1 B, Sig=210,4 Ref=360,100

Peak #	RetTime [min]	Type	Width [min]	Area [mAU*s]	Height [mAU]	Area %
1	6.899	MF	0.1594	2386.32935	249.53067	49.6023
2	7.372	FM	0.1727	2424.59790	233.94901	50.3977

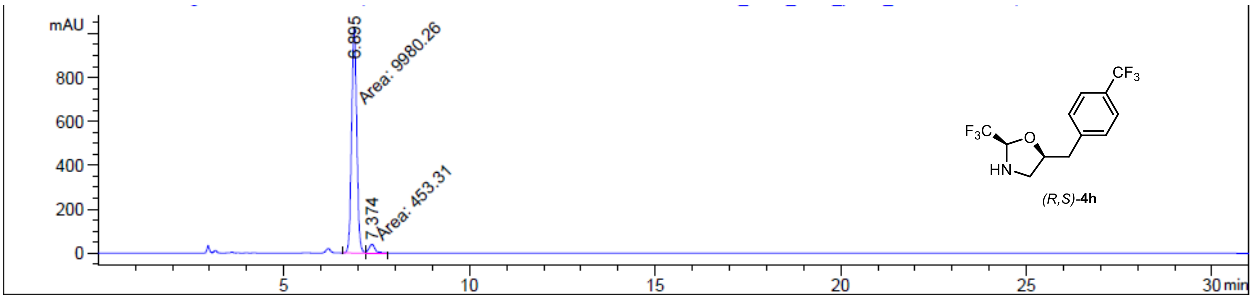

Signal 2: DAD1 B, Sig=210,4 Ref=360,100

Peak #	RetTime [min]	Type	Width [min]	Area [mAU*s]	Height [mAU]	Area %
1	6.895	MF	0.1615	9980.26367	1029.93982	95.6553
2	7.374	FM	0.1868	453.30981	40.44212	4.3447

Chiral HPLC Daicel Chiralpak IB column: 95:5 hexane/IPA, flow rate 1 mL/min, λ = 254 nm

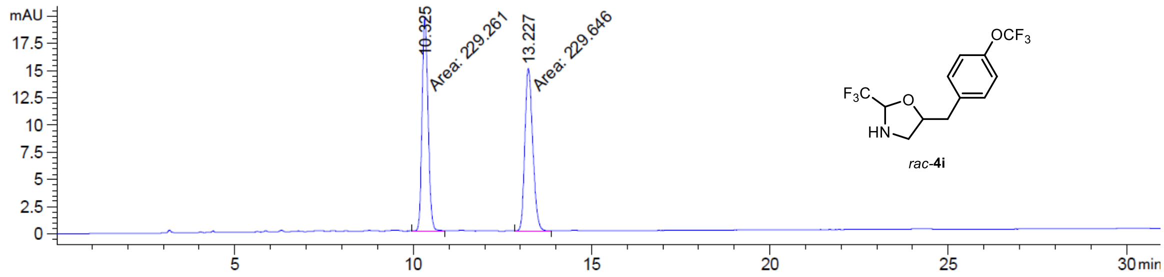

Signal 1: DAD1 A, Sig=254,4 Ref=360,100

Peak #	RetTime [min]	Type	Width [min]	Area [mAU*s]	Height [mAU]	Area %
1	10.325	MM	0.1939	229.26141	19.70669	49.9581
2	13.227	MM	0.2564	229.64565	14.92715	50.0419

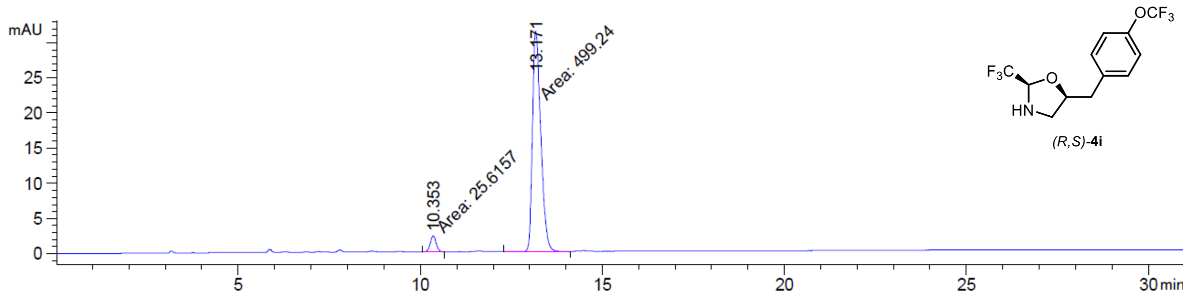

Signal 1: DAD1 A, Sig=254,4 Ref=360,100

Peak #	RetTime [min]	Type	Width [min]	Area [mAU*s]	Height [mAU]	Area %
1	10.353	MM	0.1894	25.61572	2.25459	4.8805
2	13.171	MM	0.2656	499.23953	31.33227	95.1195

Chiral HPLC Daicel Chiralpak IA column: 95:5 hexane/IPA, flow rate 1 mL/min, λ = 230 nm

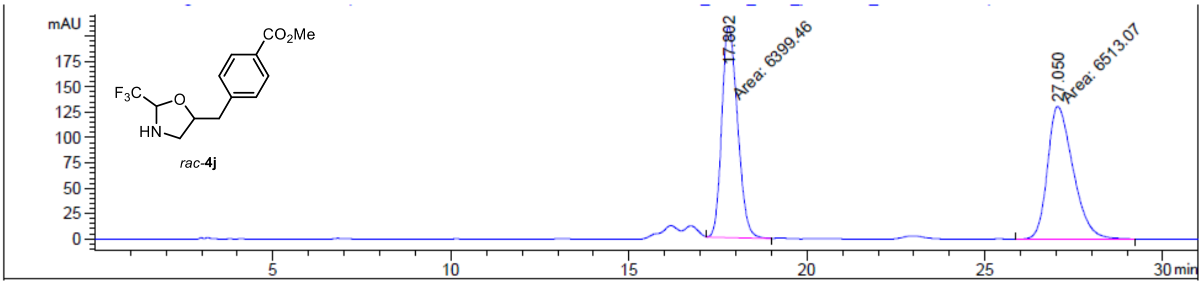

Signal 2: DAD1 B, Sig=210,4 Ref=360,100

Peak #	RetTime [min]	Type	Width [min]	Area [mAU*s]	Height [mAU]	Area %
1	6.572	MM	0.1857	4216.05859	378.37189	50.2142
2	8.921	MM	0.2470	4180.08252	282.03046	49.7858

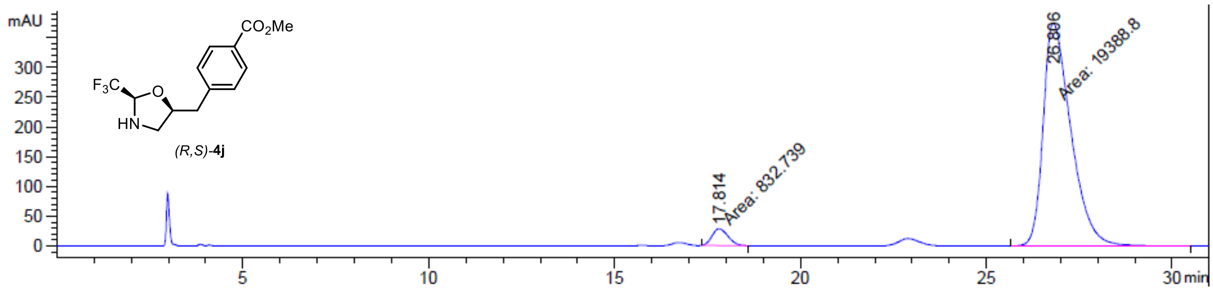

Signal 4: DAD1 D, Sig=230,4 Ref=360,100

Peak #	RetTime [min]	Type	Width [min]	Area [mAU*s]	Height [mAU]	Area %
1	17.814	MM	0.4950	832.73920	28.04005	4.1181
2	26.806	MM	0.8624	1.93888e4	374.69000	95.8819

Chiral HPLC Daicel Chiralpak IB column: 80:20 hexane/IPA, flow rate 1 mL/min, λ = 210 nm

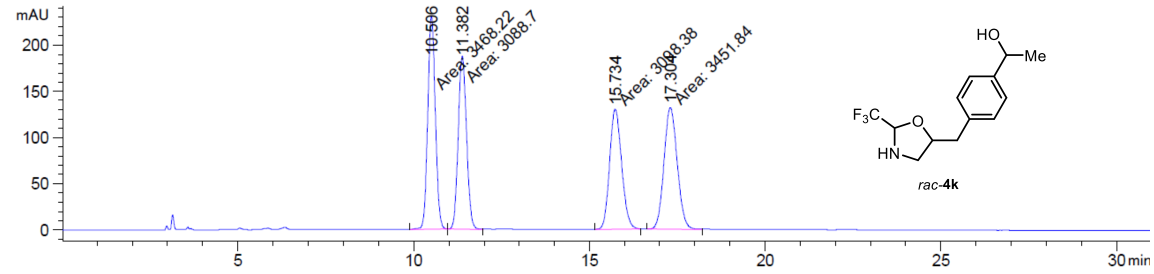

Signal 2: DAD1 B, Sig=210,4 Ref=360,100

Peak #	RetTime [min]	Type	Width [min]	Area [mAU*s]	Height [mAU]	Area %
1	10.506	MM	0.2506	3468.21899	230.69753	26.4605
2	11.382	MM	0.2744	3088.69849	187.59995	23.5650
3	15.734	MM	0.3977	3098.37988	129.84946	23.6389
4	17.304	MM	0.4375	3451.84424	131.49629	26.3356

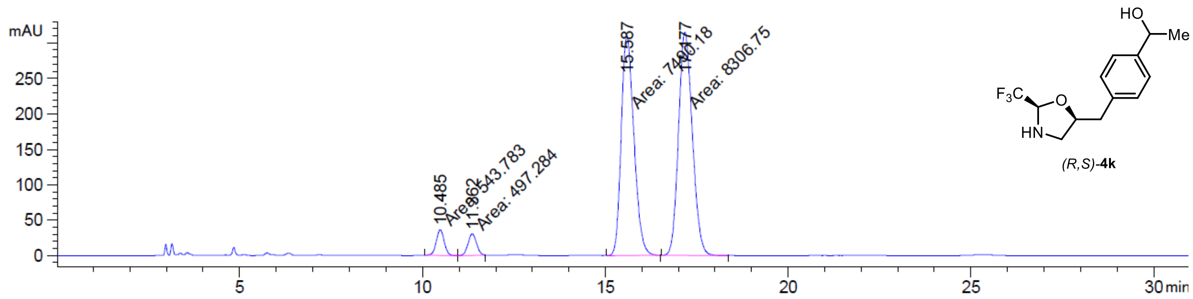

Signal 2: DAD1 B, Sig=210,4 Ref=360,100

Peak #	RetTime [min]	Type	Width [min]	Area [mAU*s]	Height [mAU]	Area %
1	10.485	MM	0.2505	543.78259	36.17344	3.2295
2	11.362	MM	0.2717	497.28378	30.50812	2.9533
3	15.587	MM	0.4023	7490.17871	310.32324	44.4838
4	17.177	MM	0.4397	8306.74902	314.88586	49.3334

Chiral HPLC Daicel Chiralpak IC column: 80:20 hexane/IPA, flow rate 1 mL/min, λ = 210 nm

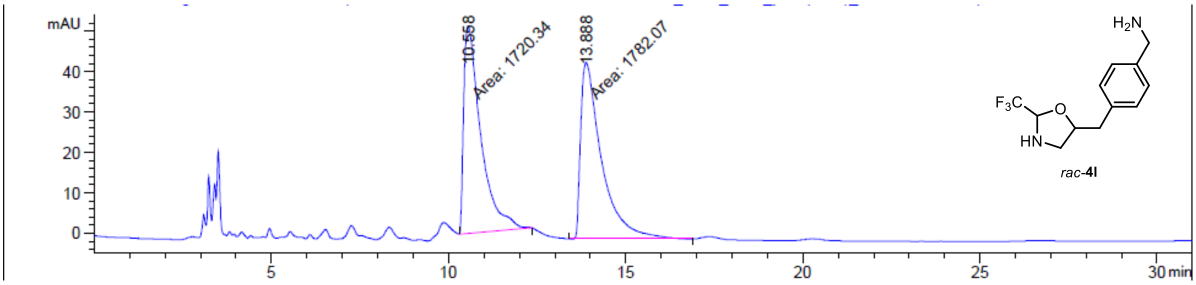

Signal 2: DAD1 B, Sig=210,4 Ref=360,100

Peak #	RetTime [min]	Type	Width [min]	Area [mAU*s]	Height [mAU]	Area %
1	10.558	MM	0.5597	1720.33533	51.23055	49.1187
2	13.888	MM	0.6824	1782.07166	43.52325	50.8813

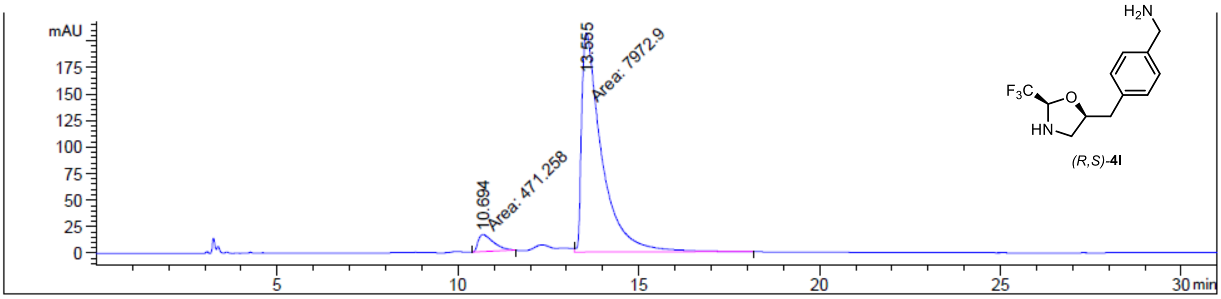

Signal 2: DAD1 B, Sig=210,4 Ref=360,100

Peak #	RetTime [min]	Type	Width [min]	Area [mAU*s]	Height [mAU]	Area %
1	10.694	MM	0.4935	471.25787	15.91644	5.5809
2	13.555	MM	0.6458	7972.90039	205.74698	94.4191

Chiral HPLC Daicel Chiralpak IA column: 95:5 hexane/IPA, flow rate 1 mL/min, λ = 214 nm

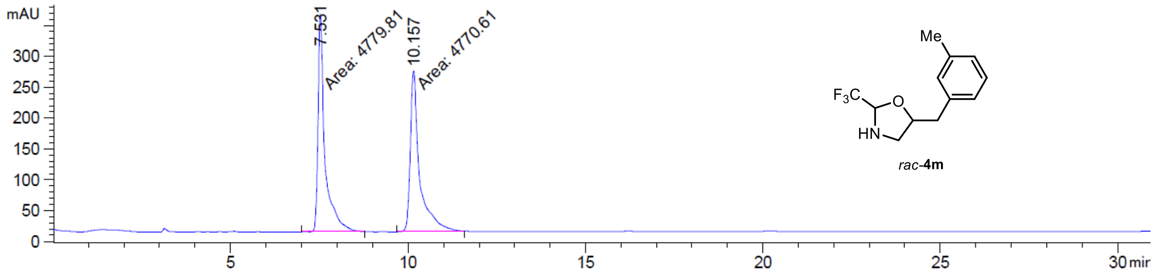

rac-**4m**

Signal 3: DAD1 C, Sig=214,4 Ref=360,100

Peak #	RetTime [min]	Type	Width [min]	Area [mAU*s]	Height [mAU]	Area %
1	7.531	MM	0.2271	4779.81445	350.79733	50.0482
2	10.157	MM	0.3056	4770.61035	260.14420	49.9518

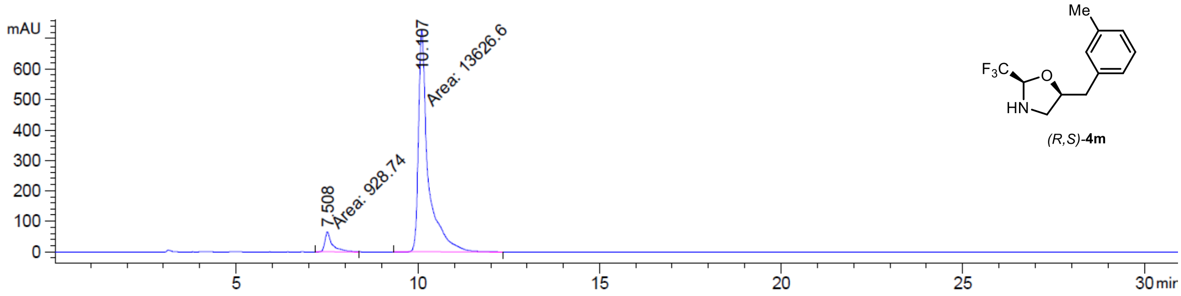

(R,S)-**4m**

Signal 3: DAD1 C, Sig=214,4 Ref=360,100

Peak #	RetTime [min]	Type	Width [min]	Area [mAU*s]	Height [mAU]	Area %
1	7.508	MM	0.2381	928.73999	64.99720	6.3807
2	10.107	MM	0.3124	1.36266e4	727.08881	93.6193

Chiral HPLC Daicel Chiralpak IA column: 95:5 hexane/IPA, flow rate 1 mL/min, λ = 254 nm

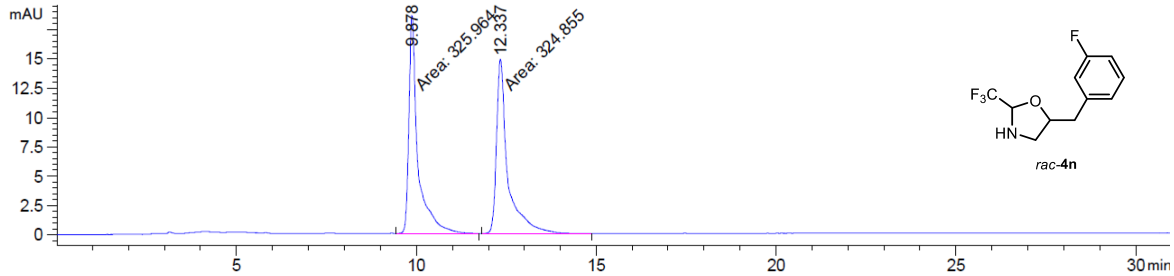

Signal 1: DAD1 A, Sig=254,4 Ref=360,100

Peak #	RetTime [min]	Type	Width [min]	Area [mAU*s]	Height [mAU]	Area %
1	9.878	MM	0.2923	325.96429	18.58866	50.0852
2	12.337	MM	0.3644	324.85541	14.85608	49.9148

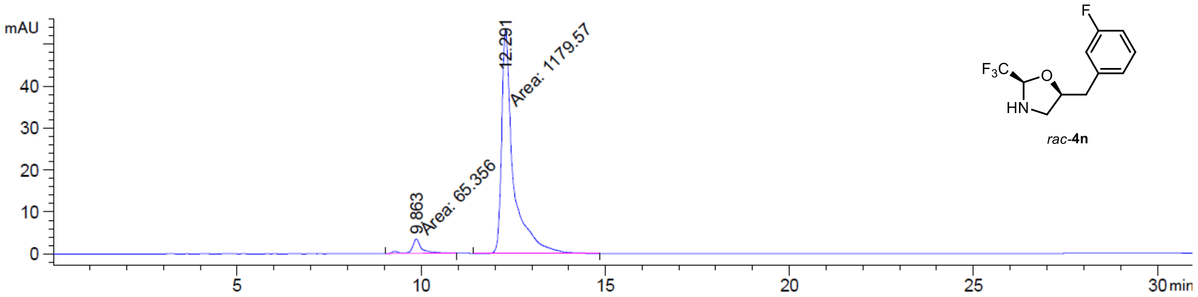

Signal 1: DAD1 A, Sig=254,4 Ref=360,100

Peak #	RetTime [min]	Type	Width [min]	Area [mAU*s]	Height [mAU]	Area %
1	9.863	MM	0.3170	65.35597	3.43638	5.2498
2	12.291	MM	0.3685	1179.56860	53.34912	94.7502

Chiral HPLC Daicel Chiralpak IA column: 95:5 hexane/IPA, flow rate 1 mL/min, λ = 210 nm

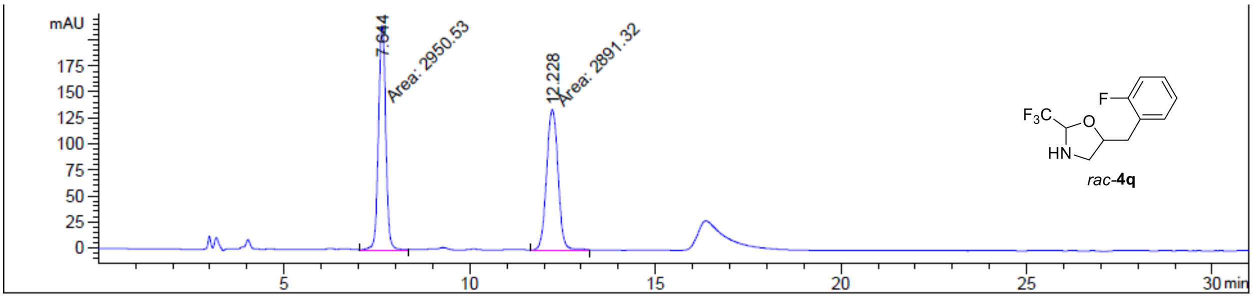

Signal 2: DAD1 B, Sig=210,4 Ref=360,100

Peak #	RetTime [min]	Type	Width [min]	Area [mAU*s]	Height [mAU]	Area %
1	7.644	MM	0.2280	2950.53345	215.72734	50.5068
2	12.228	MM	0.3568	2891.31934	135.06398	49.4932

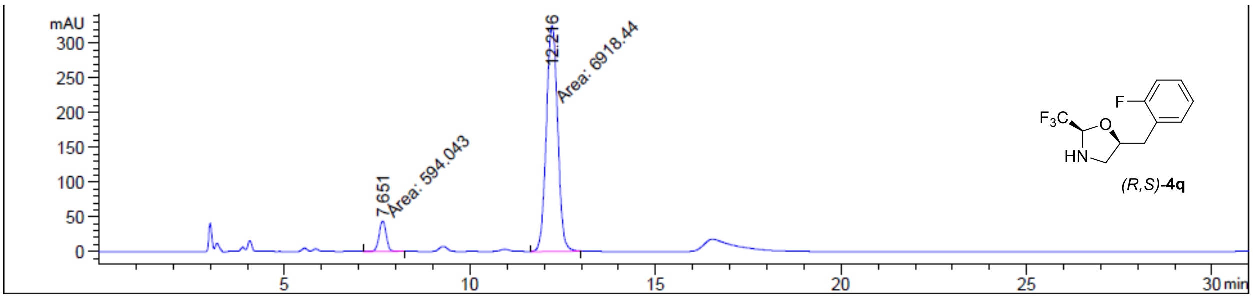

Signal 2: DAD1 B, Sig=210,4 Ref=360,100

Peak #	RetTime [min]	Type	Width [min]	Area [mAU*s]	Height [mAU]	Area %
1	7.651	MM	0.2268	594.04315	43.65479	7.9074
2	12.216	MM	0.3549	6918.43506	324.85623	92.0926

Chiral HPLC Daicel Chiralpak IA column: 95:5 hexane/IPA, flow rate 1 mL/min, λ = 210 nm

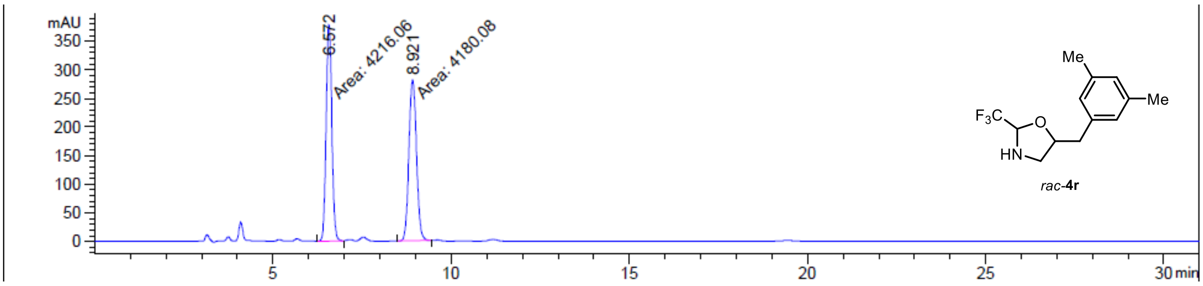

Signal 2: DAD1 B, Sig=210,4 Ref=360,100

Peak #	RetTime [min]	Type	Width [min]	Area [mAU*s]	Height [mAU]	Area %
1	6.572	MM	0.1857	4216.05859	378.37189	50.2142
2	8.921	MM	0.2470	4180.08252	282.03046	49.7858

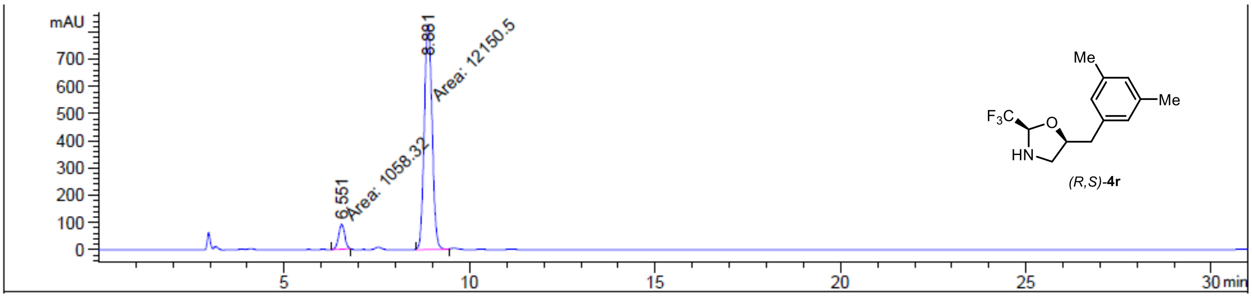

Signal 2: DAD1 B, Sig=210,4 Ref=360,100

Peak #	RetTime [min]	Type	Width [min]	Area [mAU*s]	Height [mAU]	Area %
1	6.551	MM	0.1929	1058.31970	91.42654	8.0122
2	8.881	MM	0.2449	1.21505e4	826.82660	91.9878

I. NMR Spectra

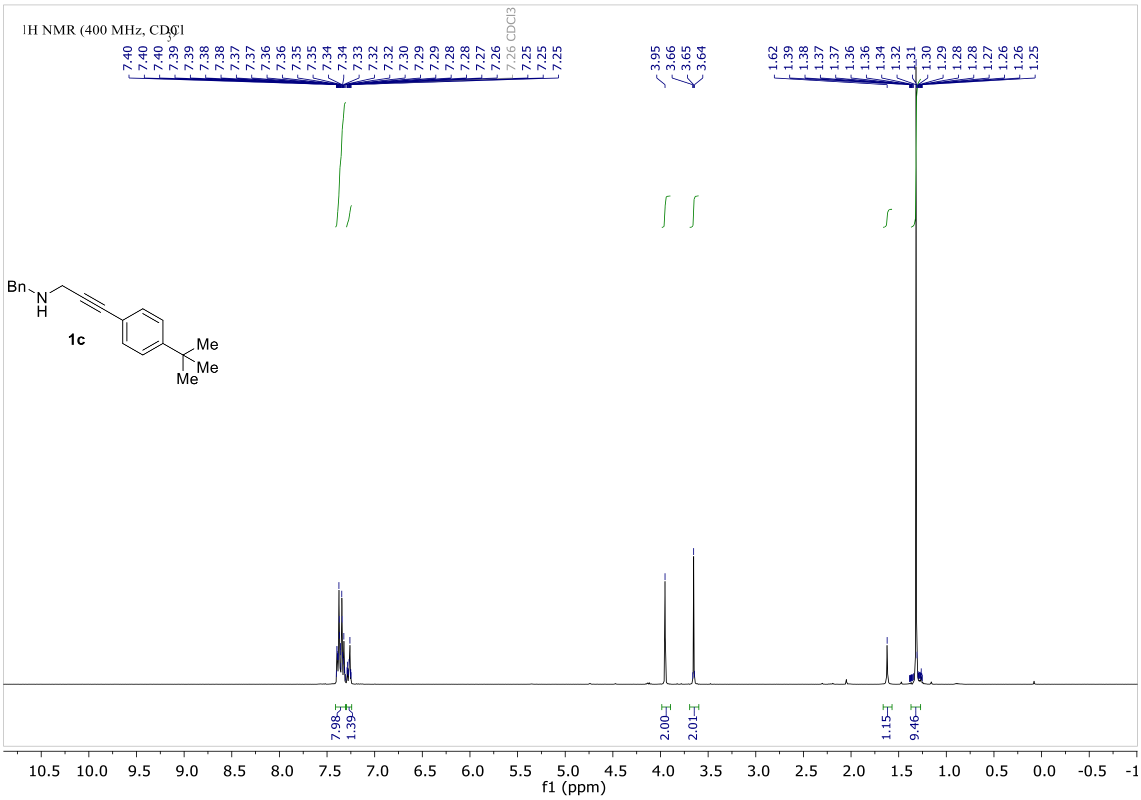

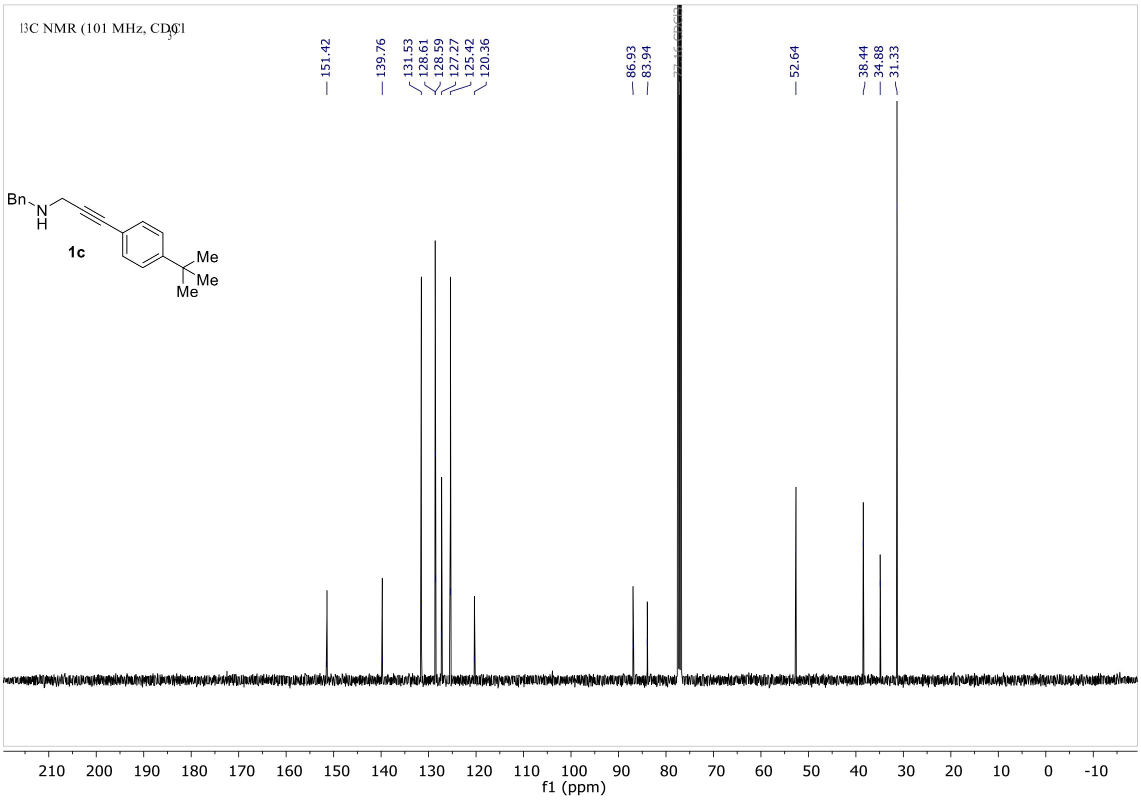

1H NMR (400 MHz, CDCl3)

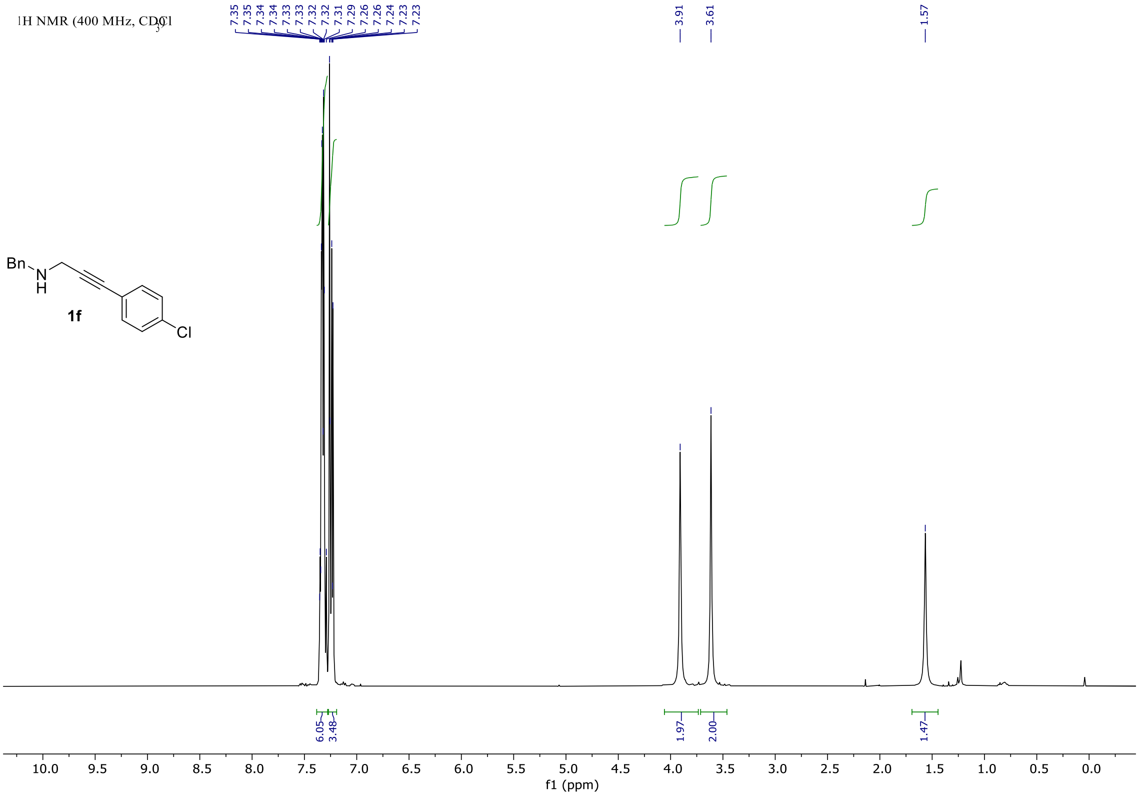

13C NMR (101 MHz, CDCl3)

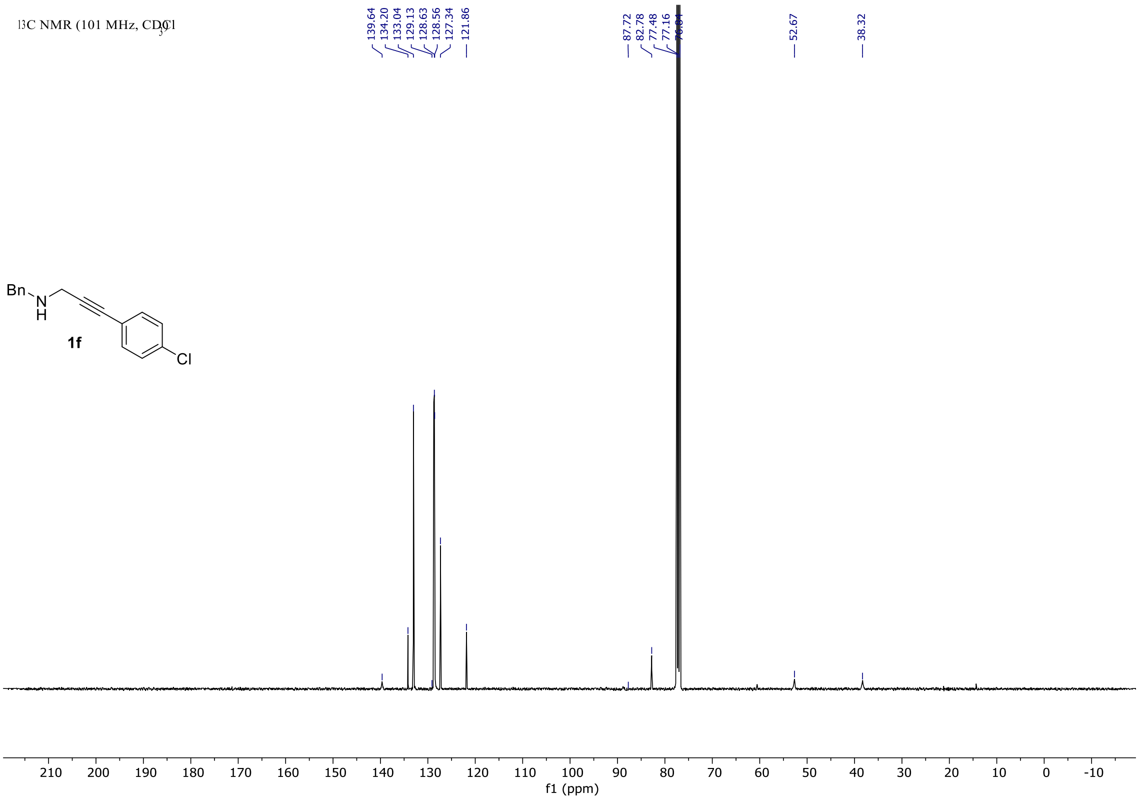

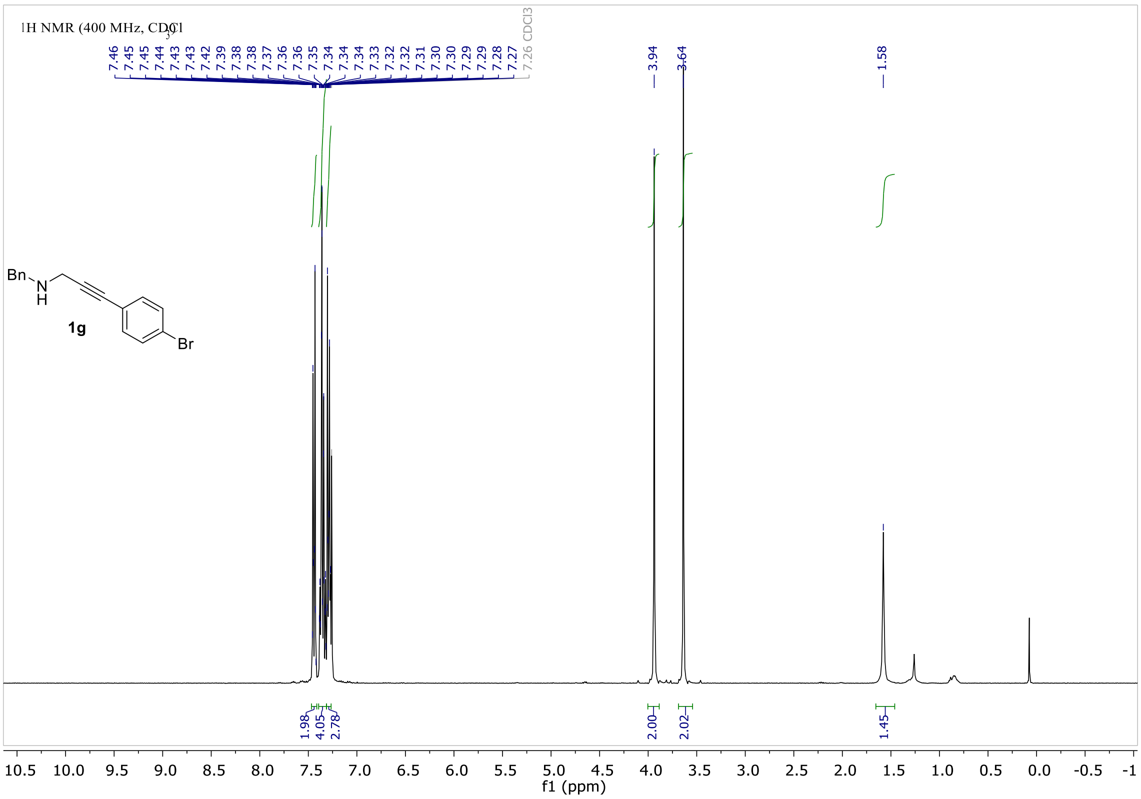
1H NMR (400 MHz, CDCl3)
7.46 7.45 7.45 7.44 7.43 7.43 7.42 7.39 7.38 7.38 7.37 7.36 7.36 7.35 7.34 7.34 7.34 7.33 7.32 7.32 7.31 7.30 7.29 7.29 7.28 7.27
7.26 CDCl3
3.94
3.64
1.58
Bn
N
H
1g
Br
1.98
4.05
2.78
2.00
2.02
1.45
10.5 10.0 9.5 9.0 8.5 8.0 7.5 7.0 6.5 6.0 5.5 5.0 4.5 4.0 3.5 3.0 2.5 2.0 1.5 1.0 0.5 0.0 -0.5 -1
f1 (ppm)

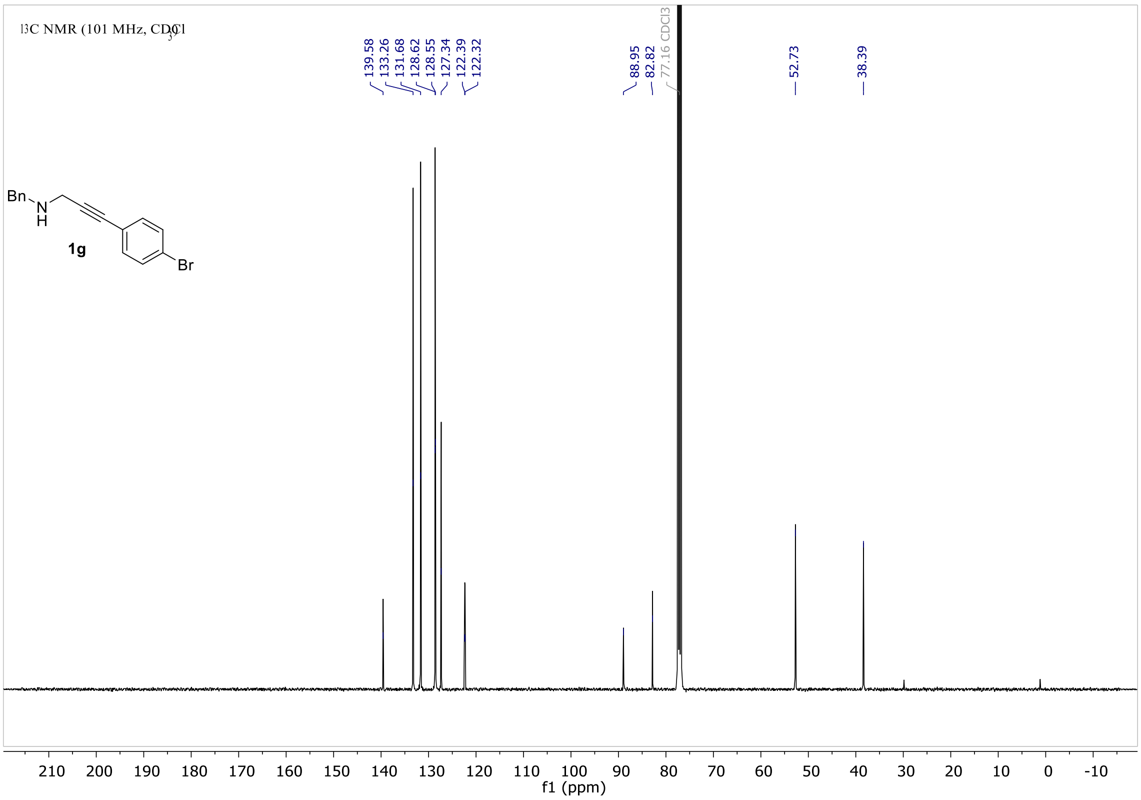
13C NMR (101 MHz, CDCl3)
139.58 133.26 131.68 128.62 128.55 127.34 122.39 122.32
88.95 82.82
77.16 CDCl3
52.73
38.39
Bn
N
H
1g
Br
210 200 190 180 170 160 150 140 130 120 110 100 90 80 70 60 50 40 30 20 10 0 -10
f1 (ppm)

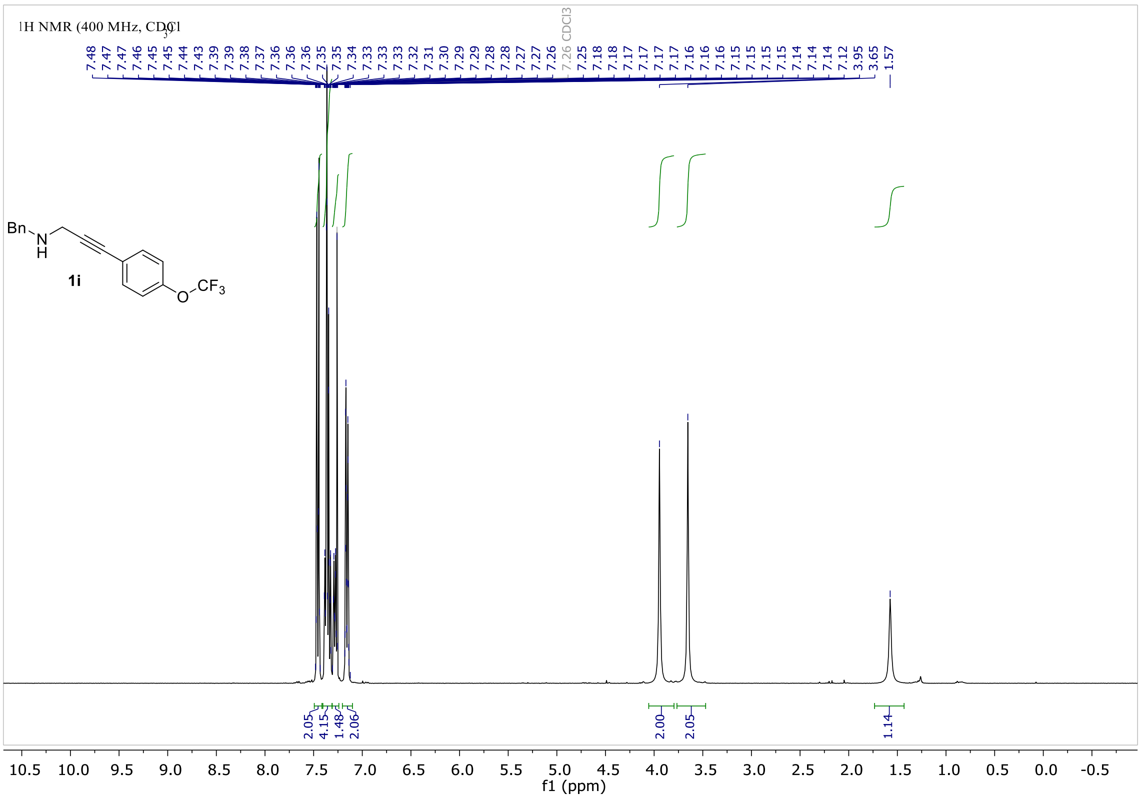
1H NMR (400 MHz, CDCl3)
7.48 7.47 7.46 7.45 7.45 7.44 7.43 7.39 7.39 7.38 7.37 7.36 7.36 7.35 7.35 7.34 7.33 7.33 7.33 7.32 7.31 7.30 7.29 7.29 7.28 7.28 7.27 7.27 7.26 CDCl3 7.25 7.18 7.18 7.17 7.17 7.17 7.16 7.16 7.15 7.15 7.15 7.15 7.14 7.14 7.14 7.12 3.95 3.65 1.57
Bn
N
H
1i
O
CF3
2.05 4.15 1.48 2.06 2.00 2.05 1.14
f1 (ppm)

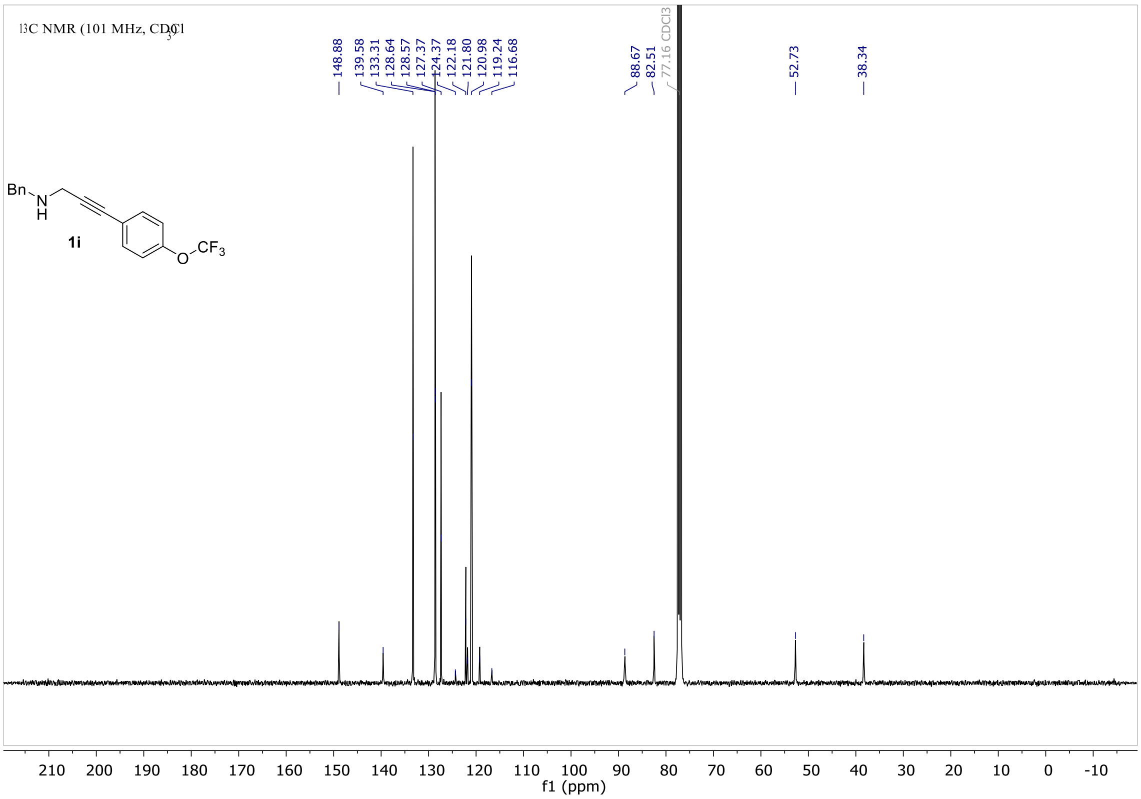
13C NMR (101 MHz, CDCl3)
148.88 139.58 133.31 128.64 128.57 127.37 124.37 122.18 121.80 120.98 119.24 116.68 88.67 82.51 77.16 CDCl3 52.73 38.34
Bn
N
H
1i
O
CF3
f1 (ppm)

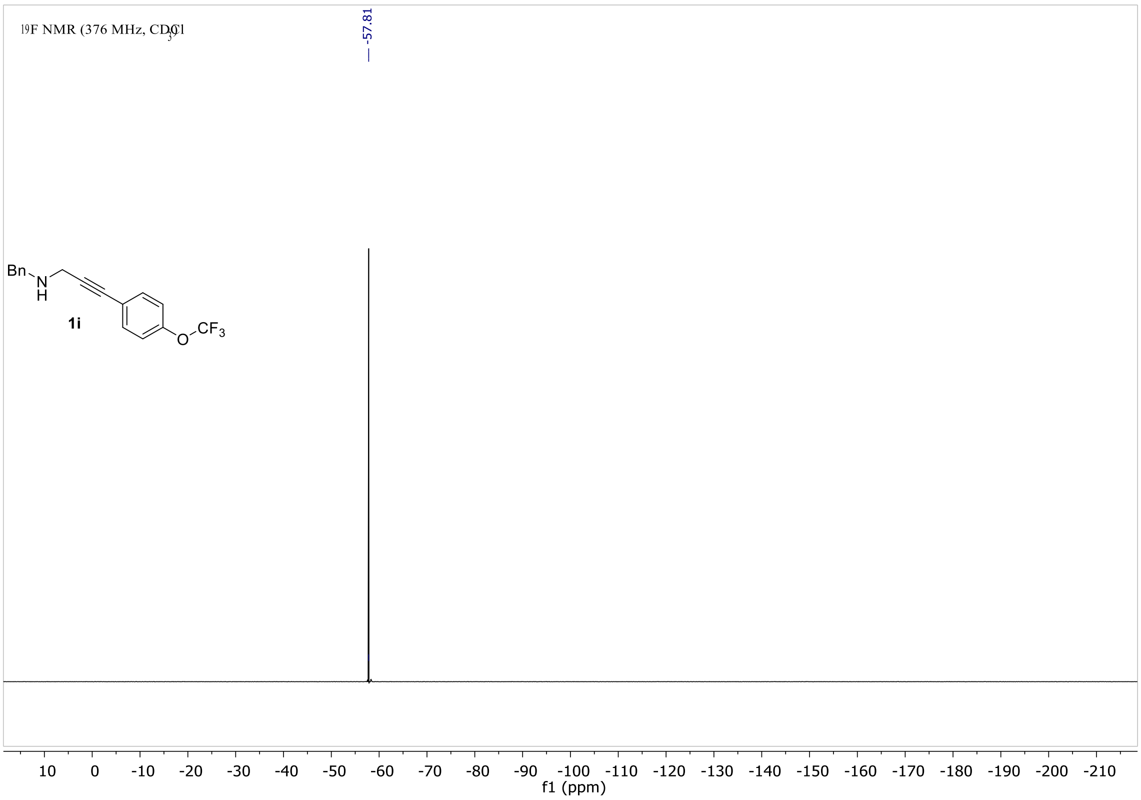
19F NMR (376 MHz, CDCl3)
-57.81
Bn
N
H
1i
O
CF3
f1 (ppm)

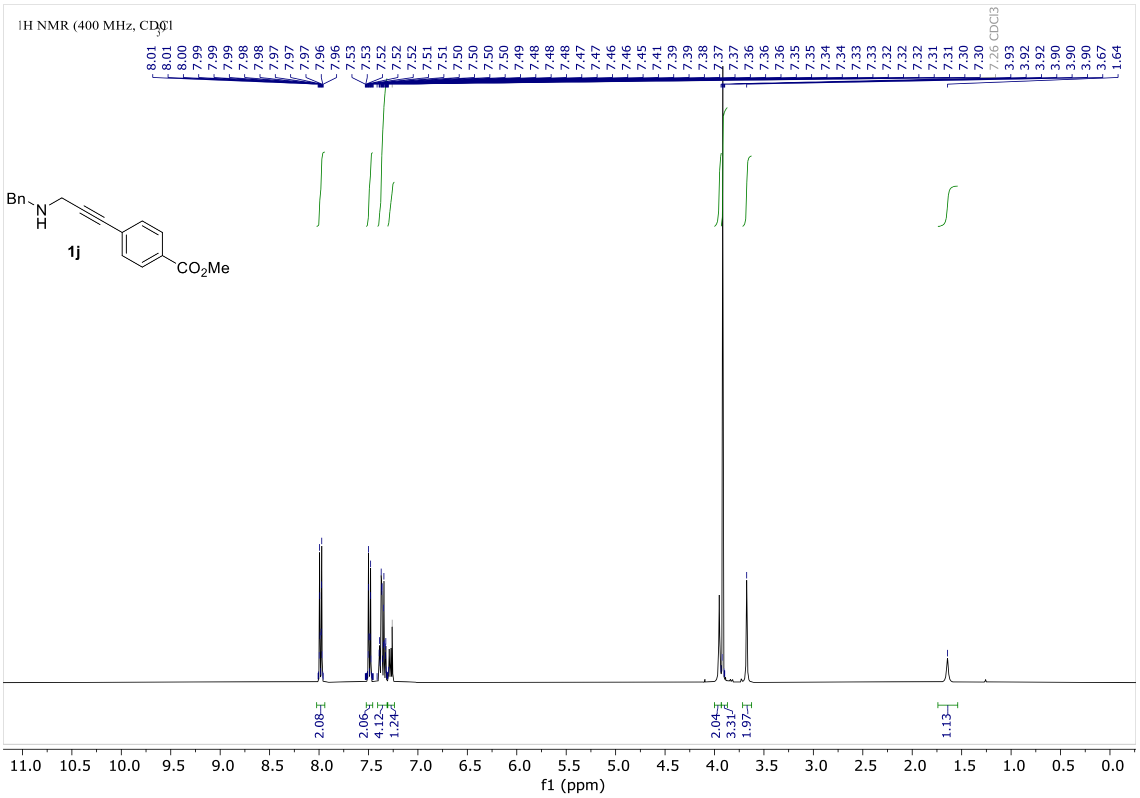
1H NMR (400 MHz, CDCl3)
7.26 CDCl3
Bn
N
H
1j
CO2Me
2.08
2.06
4.12
1.24
2.04
3.31
1.97
1.13
f1 (ppm)

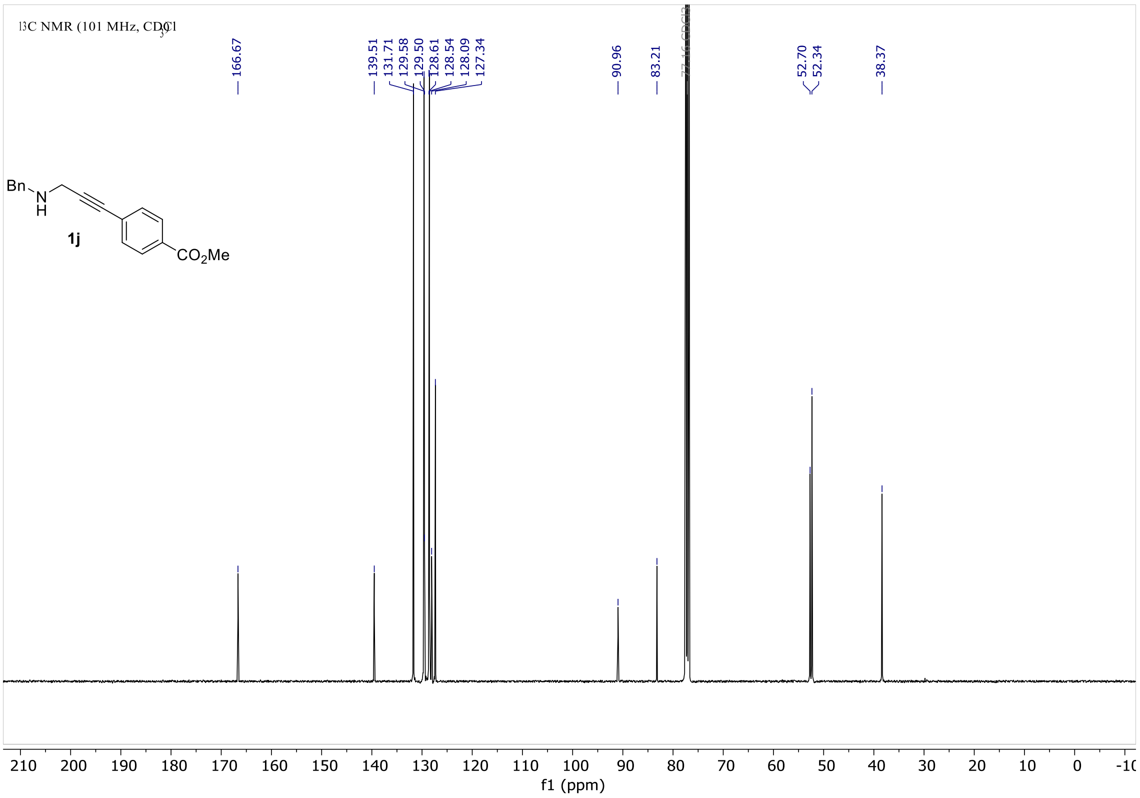
13C NMR (101 MHz, CDCl3)
166.67
139.51
131.71
129.58
129.50
128.61
128.54
128.09
127.34
90.96
83.21
52.70
52.34
38.37
Bn
N
H
1j
CO2Me
f1 (ppm)

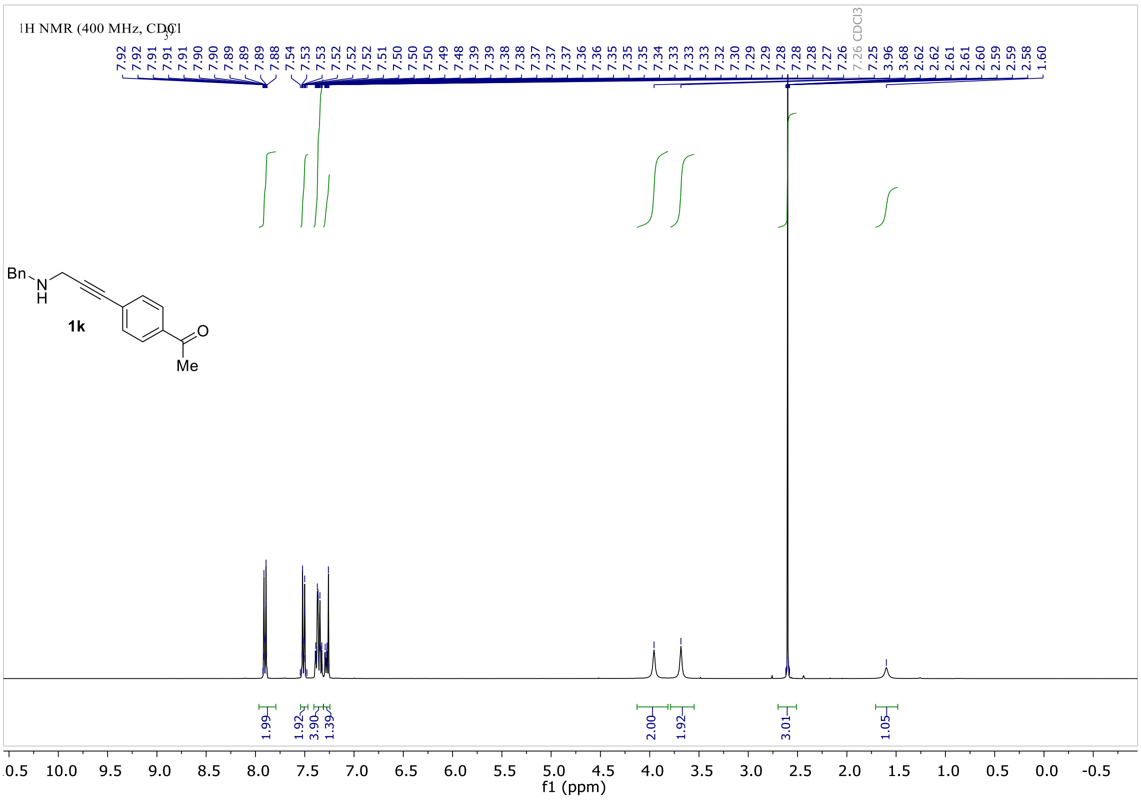
1H NMR (400 MHz, CDCl3)
7.92 7.92 7.91 7.91 7.91 7.90 7.90 7.89 7.89 7.89 7.88 7.54 7.53 7.53 7.52 7.52 7.52 7.51 7.50 7.50 7.50 7.49 7.48 7.39 7.39 7.38 7.38 7.37 7.37 7.37 7.36 7.36 7.35 7.35 7.35 7.34 7.33 7.33 7.32 7.32 7.30 7.29 7.29 7.28 7.28 7.28 7.27 7.26 7.26 CDCl3 7.25 3.96 3.68 2.62 2.62 2.61 2.61 2.60 2.59 2.59 2.58 1.60
Bn
N
H
1k
O
Me
1.99
1.92
3.90
1.39
2.00
1.92
3.01
1.05
f1 (ppm)

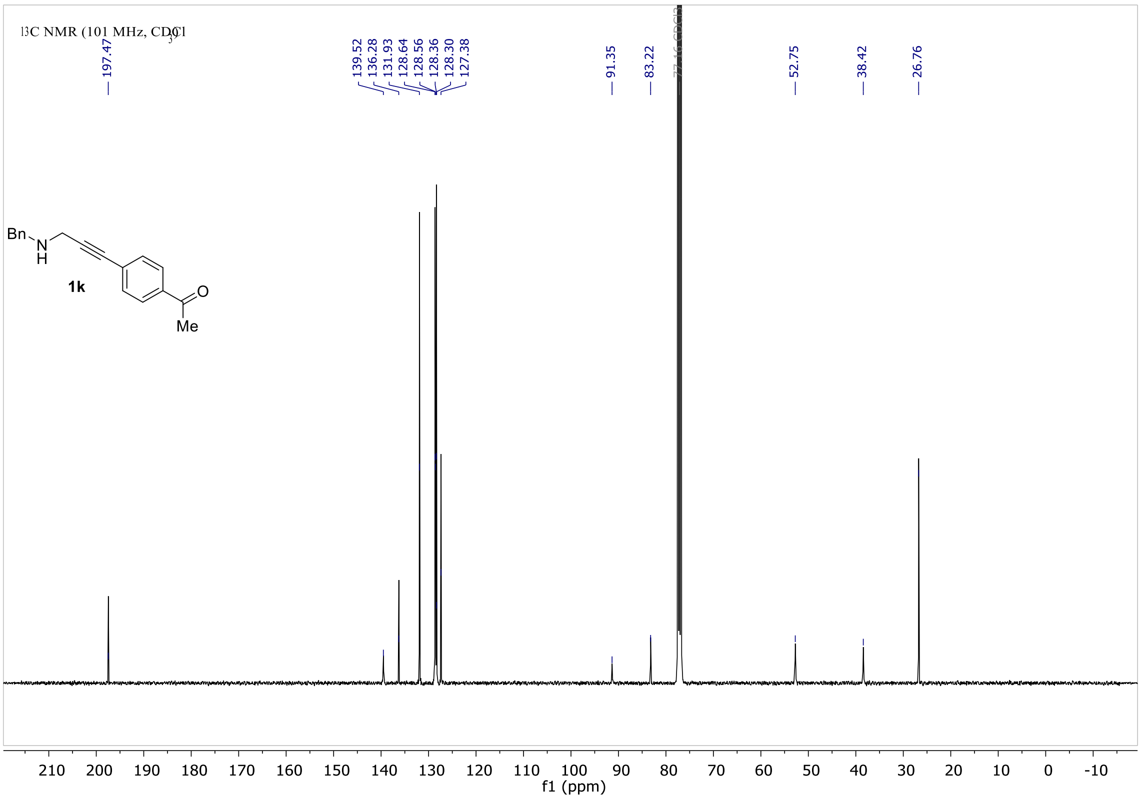
13C NMR (101 MHz, CDCl3)
197.47
139.52
136.28
131.93
128.64
128.56
128.36
128.30
127.38
91.35
83.22
52.75
38.42
26.76
Bn
N
H
1k
O
Me
210 200 190 180 170 160 150 140 130 120 110 100 90 80 70 60 50 40 30 20 10 0 -10
f1 (ppm)

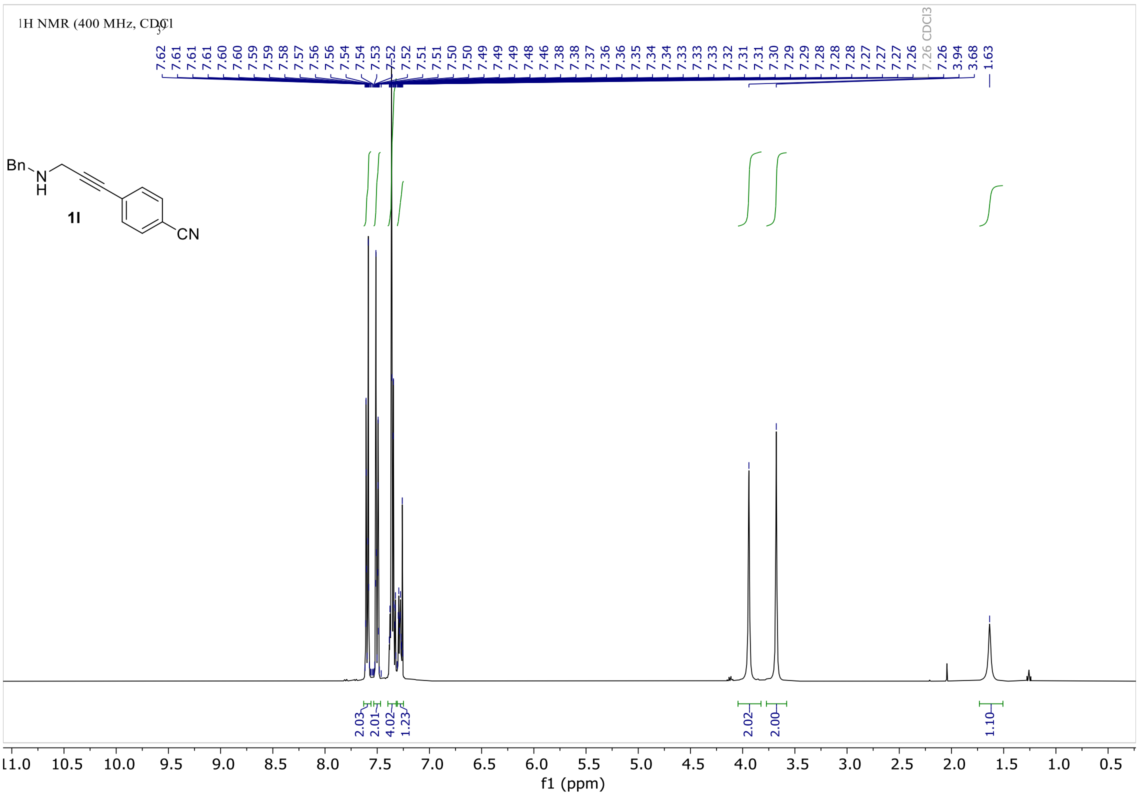
1H NMR (400 MHz, CDCl3)
7.62 7.61 7.61 7.60 7.59 7.59 7.58 7.57 7.56 7.56 7.54 7.54 7.53 7.52 7.52 7.51 7.51 7.50 7.49 7.49 7.48 7.46 7.38 7.38 7.37 7.36 7.36 7.35 7.34 7.34 7.33 7.33 7.33 7.32 7.31 7.31 7.30 7.29 7.29 7.28 7.28 7.28 7.27 7.27 7.26 7.26 CDCl3 7.26 3.94 3.68 1.63
Bn
N
H
1l
CN
2.03 2.01 4.02 1.23 2.02 2.00 1.10
11.0 10.5 10.0 9.5 9.0 8.5 8.0 7.5 7.0 6.5 6.0 5.5 5.0 4.5 4.0 3.5 3.0 2.5 2.0 1.5 1.0 0.5
f1 (ppm)

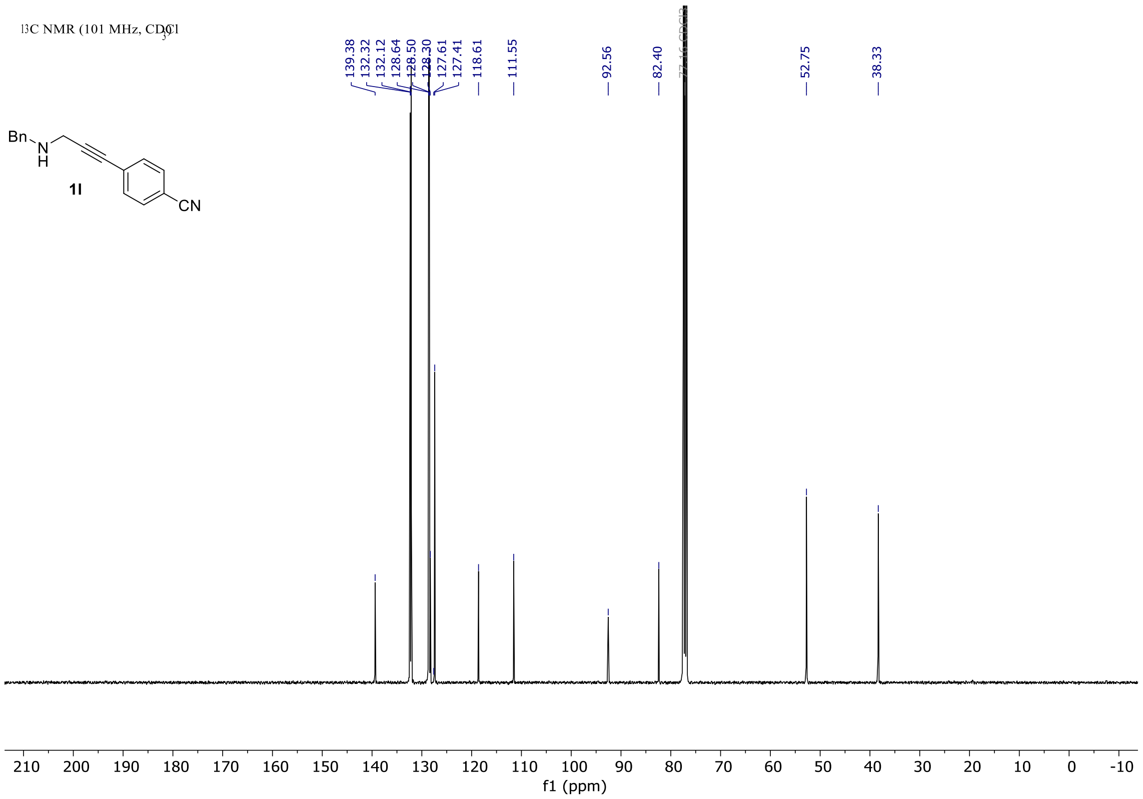
13C NMR (101 MHz, CDCl3
Bn
N
H
1l
CN
139.38
132.32
132.12
128.64
128.50
128.30
127.61
127.41
118.61
111.55
92.56
82.40
52.75
38.33
210 200 190 180 170 160 150 140 130 120 110 100 90 80 70 60 50 40 30 20 10 0 -10
f1 (ppm)

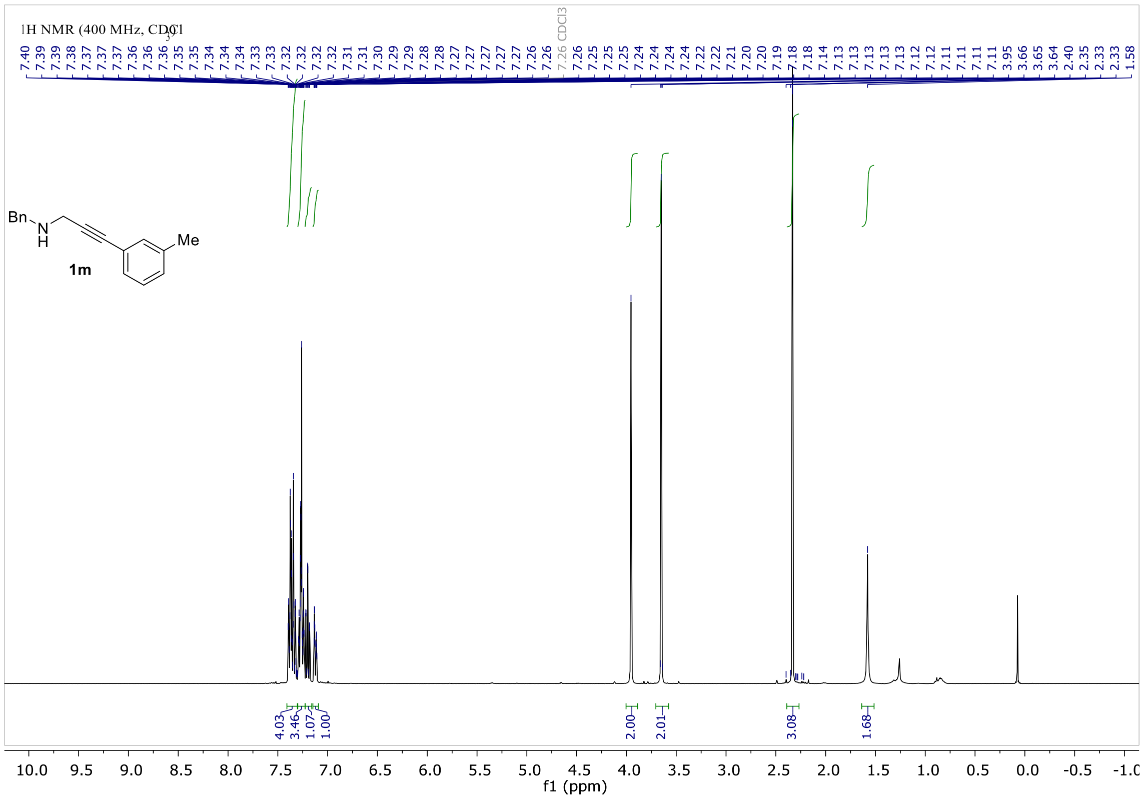
1H NMR (400 MHz, CDCl
Bn
N
H
Me
1m
7.40 7.39 7.39 7.38 7.37 7.37 7.37 7.36 7.36 7.35 7.35 7.34 7.34 7.33 7.33 7.32 7.32 7.32 7.31 7.30 7.29 7.29 7.28 7.28 7.27 7.27 7.27 7.27 7.26 7.26 7.26 7.25 7.25 7.25 7.24 7.24 7.24 7.24 7.22 7.22 7.21 7.20 7.19 7.18 7.14 7.13 7.13 7.13 7.13 7.12 7.12 7.11 7.11 7.11 3.95 3.66 3.65 3.64 2.40 2.35 2.33 2.33 1.58
7.26 CDCl3
4.03
3.46
1.07
1.00
2.00
2.01
3.08
1.68
10.0 9.5 9.0 8.5 8.0 7.5 7.0 6.5 6.0 5.5 5.0 4.5 4.0 3.5 3.0 2.5 2.0 1.5 1.0 0.5 0.0 -0.5 -1.0
f1 (ppm)

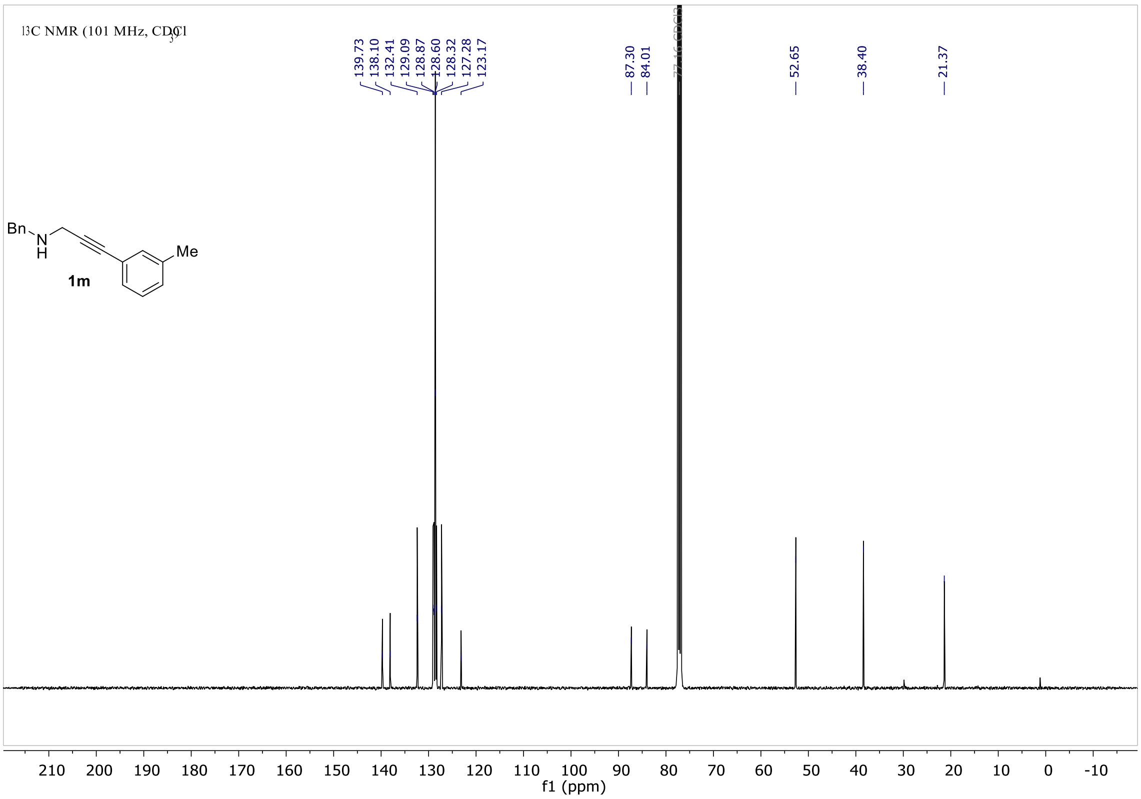
13C NMR (101 MHz, CDCl3)
139.73
138.10
132.41
129.09
128.87
128.60
128.32
127.28
123.17
87.30
84.01
52.65
38.40
21.37
Bn
N
H
Me
1m
f1 (ppm)

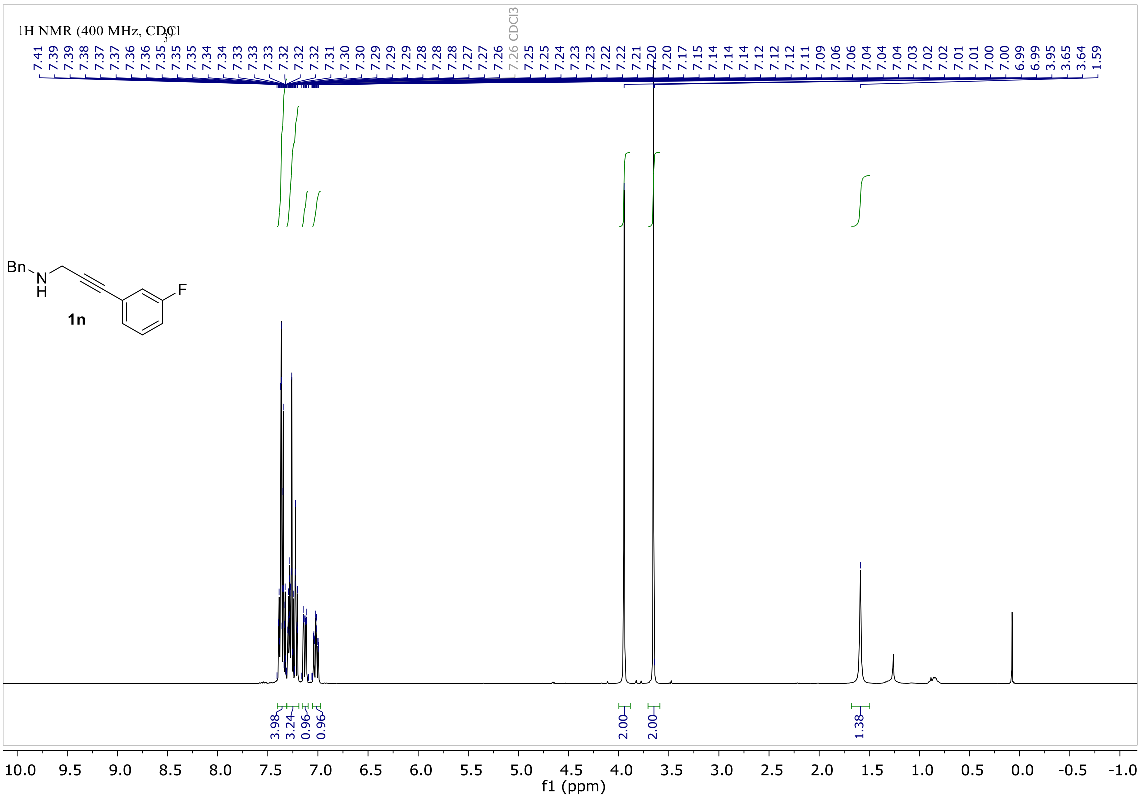
1H NMR (400 MHz, CDCl3)
7.41 7.39 7.38 7.37 7.37 7.36 7.36 7.35 7.35 7.35 7.34 7.34 7.33 7.33 7.33 7.32 7.32 7.32 7.31 7.30 7.30 7.29 7.29 7.29 7.28 7.28 7.28 7.27 7.27 7.26 7.25 7.25 7.24 7.23 7.23 7.22 7.22 7.21 7.20 7.20 7.17 7.15 7.14 7.14 7.12 7.12 7.12 7.11 7.09 7.06 7.04 7.04 7.03 7.02 7.02 7.01 7.00 7.00 6.99 6.99 3.95 3.65 3.64 1.59
Bn
N
H
F
1n
3.98
3.24
0.96
0.96
2.00
2.00
1.38
f1 (ppm)

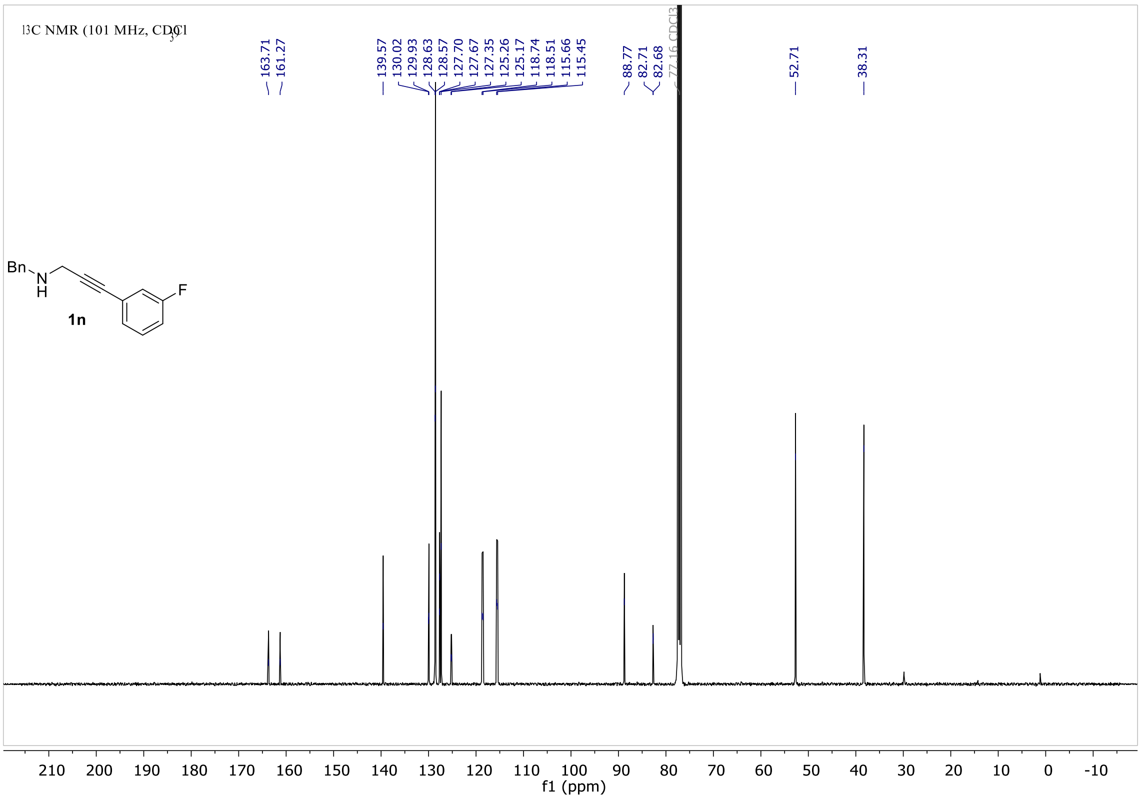
13C NMR (101 MHz, CDCl3)
163.71
161.27
139.57
130.02
129.93
128.63
128.57
127.70
127.67
127.35
125.26
125.17
118.74
118.51
115.66
115.45
88.77
82.71
82.68
77.16 CDCl3
52.71
38.31
Bn
N
H
F
1n
210 200 190 180 170 160 150 140 130 120 110 100 90 80 70 60 50 40 30 20 10 0 -10
f1 (ppm)

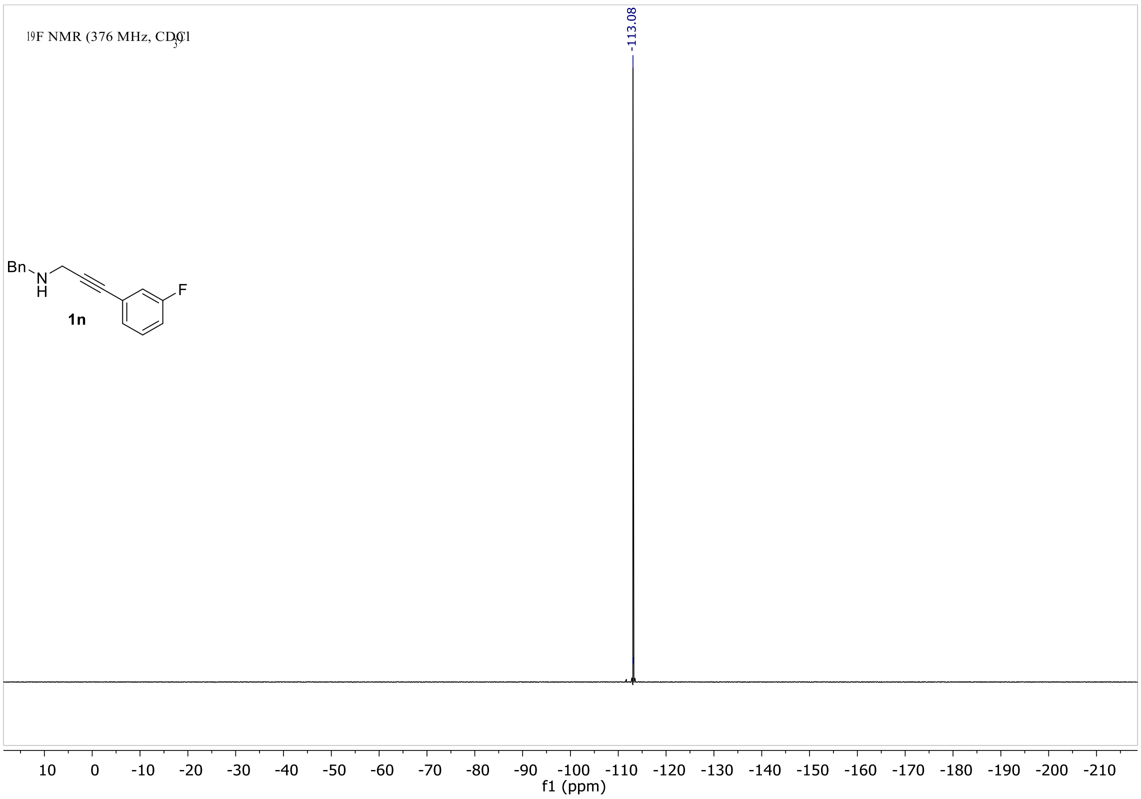
19F NMR (376 MHz, CDCl3)
-113.08
Bn
N
H
F
1n
10 0 -10 -20 -30 -40 -50 -60 -70 -80 -90 -100 -110 -120 -130 -140 -150 -160 -170 -180 -190 -200 -210
f1 (ppm)

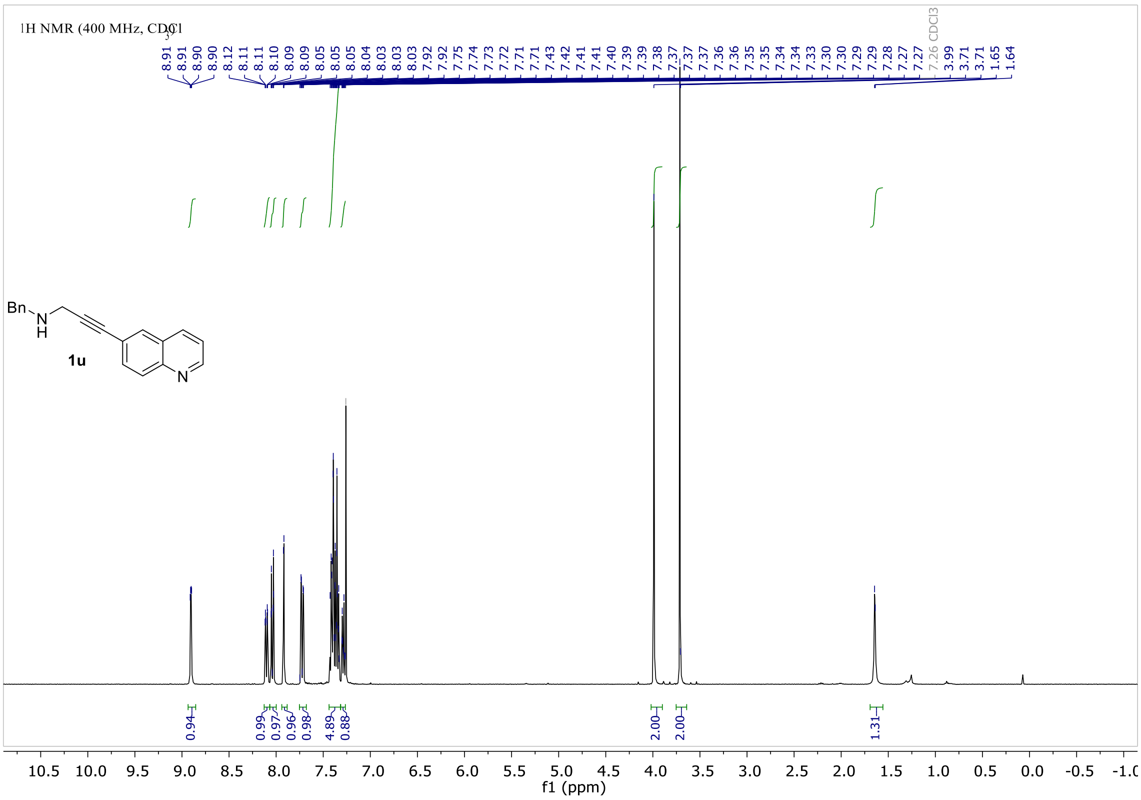
1H NMR (400 MHz, CDCl3)
8.91 8.91 8.90 8.90 8.12 8.11 8.11 8.10 8.09 8.09 8.05 8.05 8.04 8.03 8.03 8.03 7.92 7.92 7.75 7.74 7.73 7.72 7.71 7.71 7.43 7.42 7.41 7.40 7.39 7.39 7.38 7.37 7.37 7.37 7.36 7.36 7.35 7.35 7.34 7.33 7.30 7.29 7.29 7.28 7.27 7.26 CDCl3 3.99 3.71 3.71 1.65 1.64
Bn
N
H
1u
N
0.94 0.99 0.97 0.96 0.98 4.89 0.88 2.00 2.00 1.31
f1 (ppm)

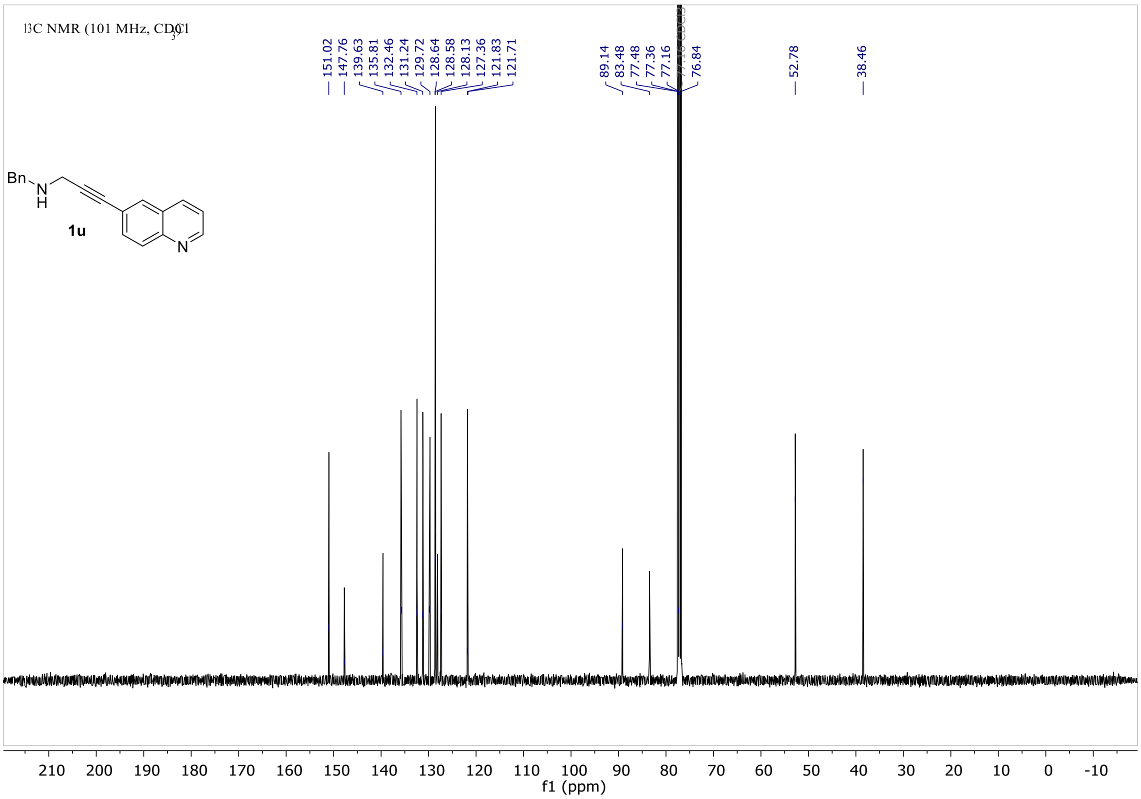
13C NMR (101 MHz, CDCl3)
151.02 147.76 139.63 135.81 132.46 131.24 129.72 128.64 128.58 128.13 127.36 121.83 121.71
89.14 83.48 77.48 77.36 77.16 76.84
52.78
38.46
Bn
N
H
1u
N
f1 (ppm)

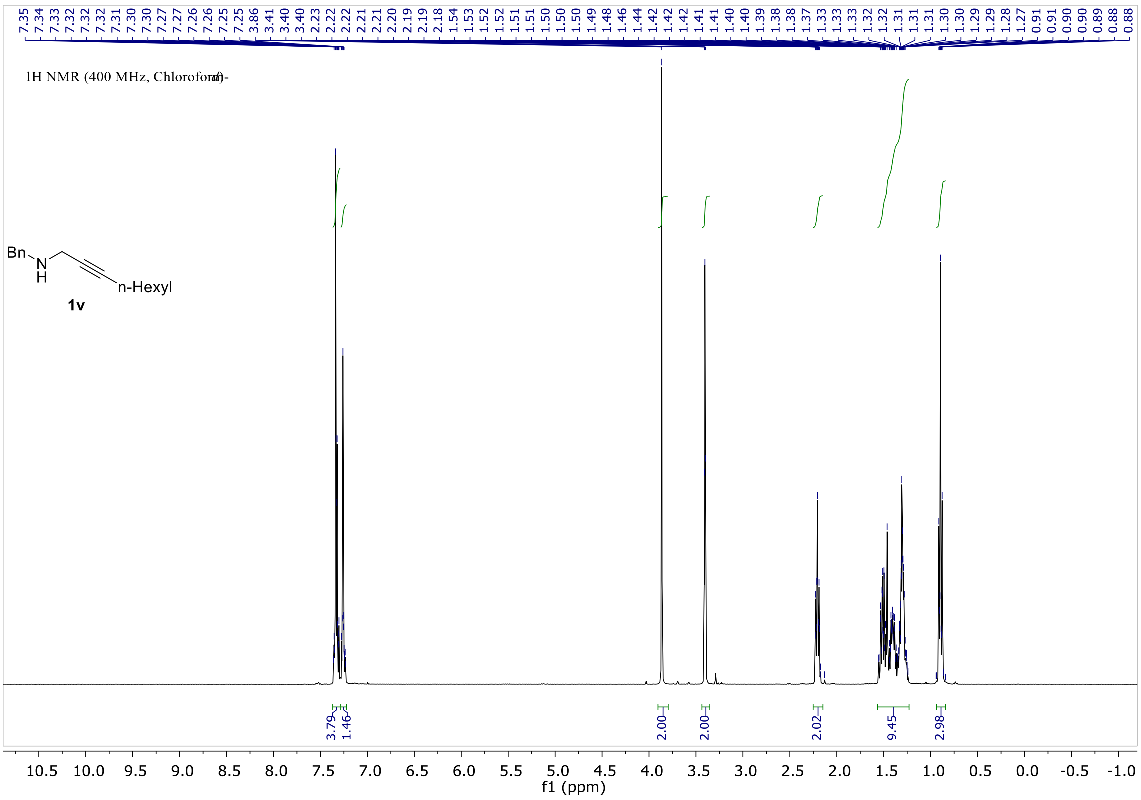
1H NMR (400 MHz, Chloroform-d)
Bn
N
H
n-Hexyl
1v
f1 (ppm)

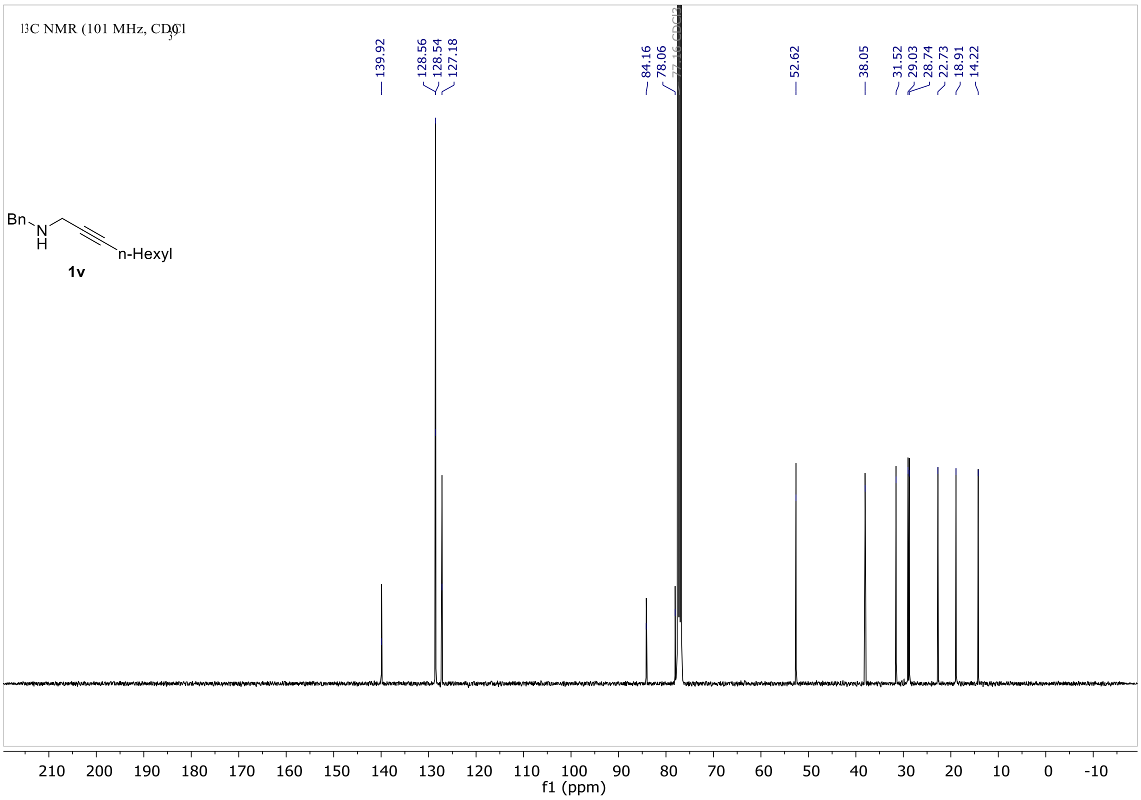
13C NMR (101 MHz, CDCl3)
Bn
N
H
n-Hexyl
1v
139.92
128.56
128.54
127.18
84.16
78.06
52.62
38.05
31.52
29.03
28.74
22.73
18.91
14.22
f1 (ppm)

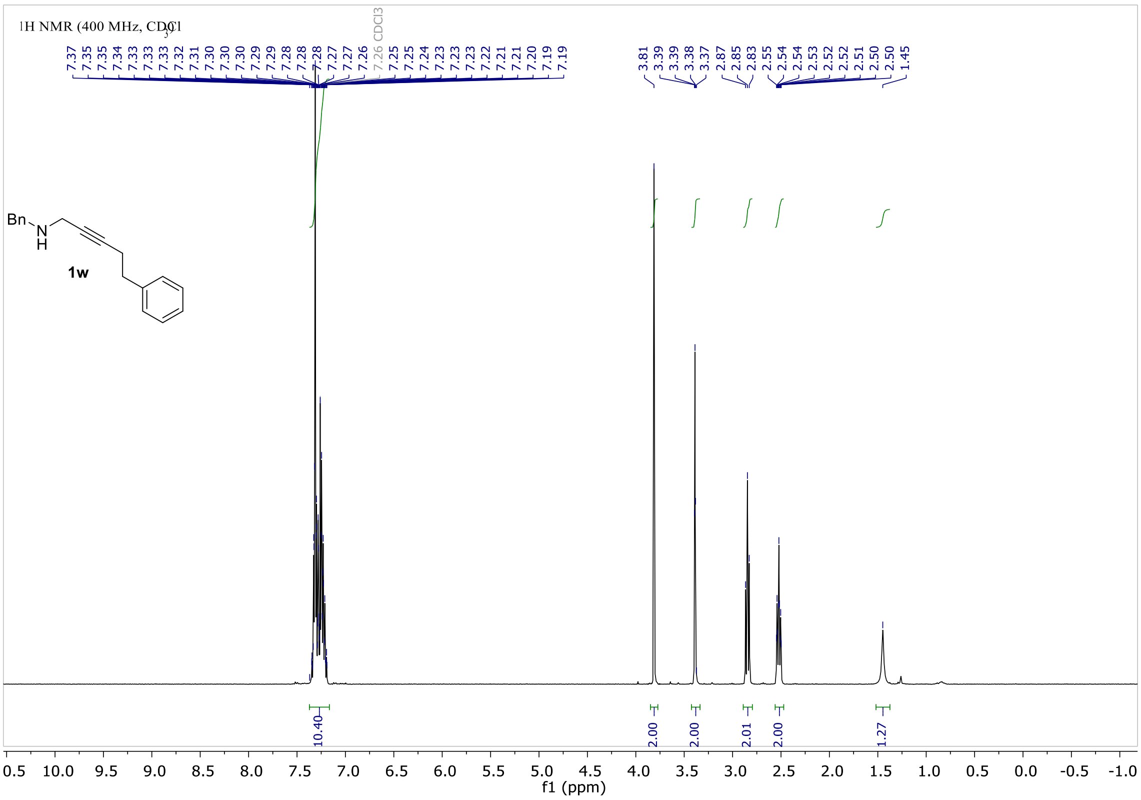
1H NMR (400 MHz, CDCl3
7.37 7.35 7.34 7.33 7.33 7.33 7.32 7.31 7.30 7.30 7.29 7.29 7.28 7.28 7.28 7.27 7.27 7.26 7.26 CDCl3 7.25 7.25 7.24 7.23 7.23 7.23 7.22 7.21 7.21 7.20 7.19 7.19
3.81 3.39 3.39 3.38 3.37 2.87 2.85 2.83 2.55 2.54 2.54 2.53 2.52 2.52 2.51 2.50 2.50 1.45
Bn
N
H
1w
10.40
2.00
2.00
2.01
2.00
1.27
f1 (ppm)

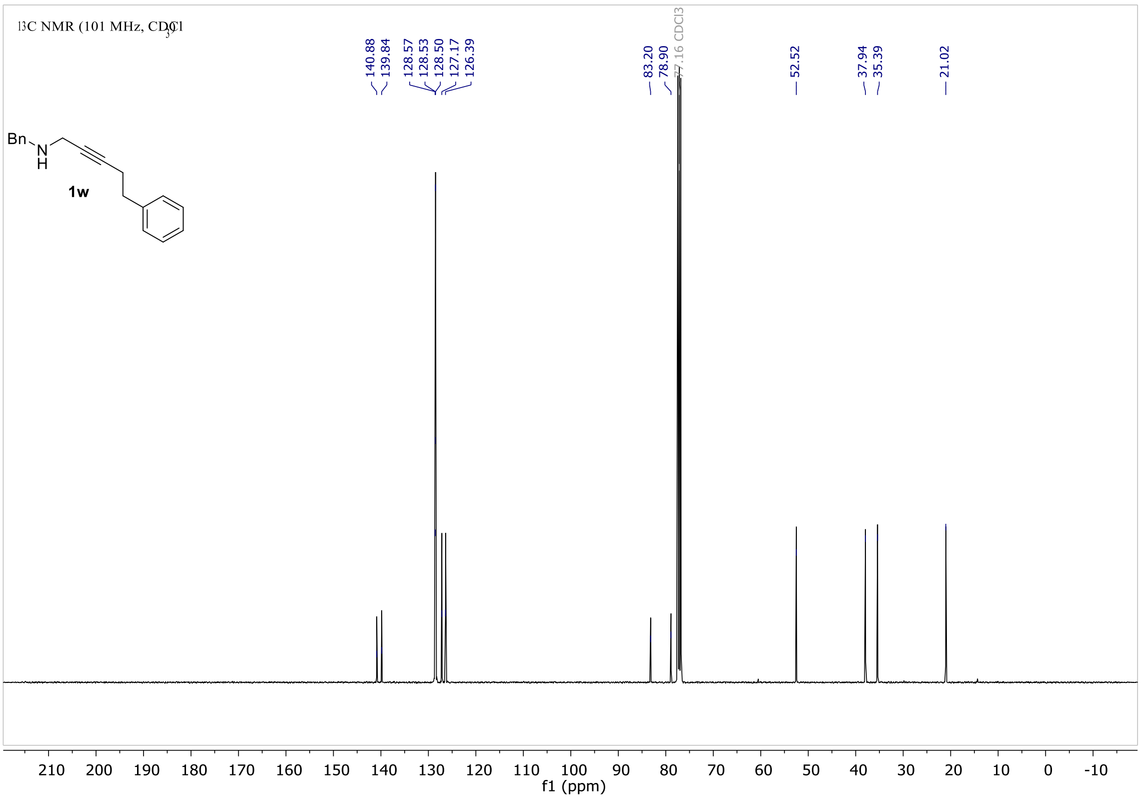
13C NMR (101 MHz, CDCl3
140.88 139.84 128.57 128.53 128.50 127.17 126.39
83.20 78.90 77.16 CDCl3
52.52 37.94 35.39 21.02
Bn
N
H
1w
f1 (ppm)

1H NMR (400 MHz, Chloroform-*d*)

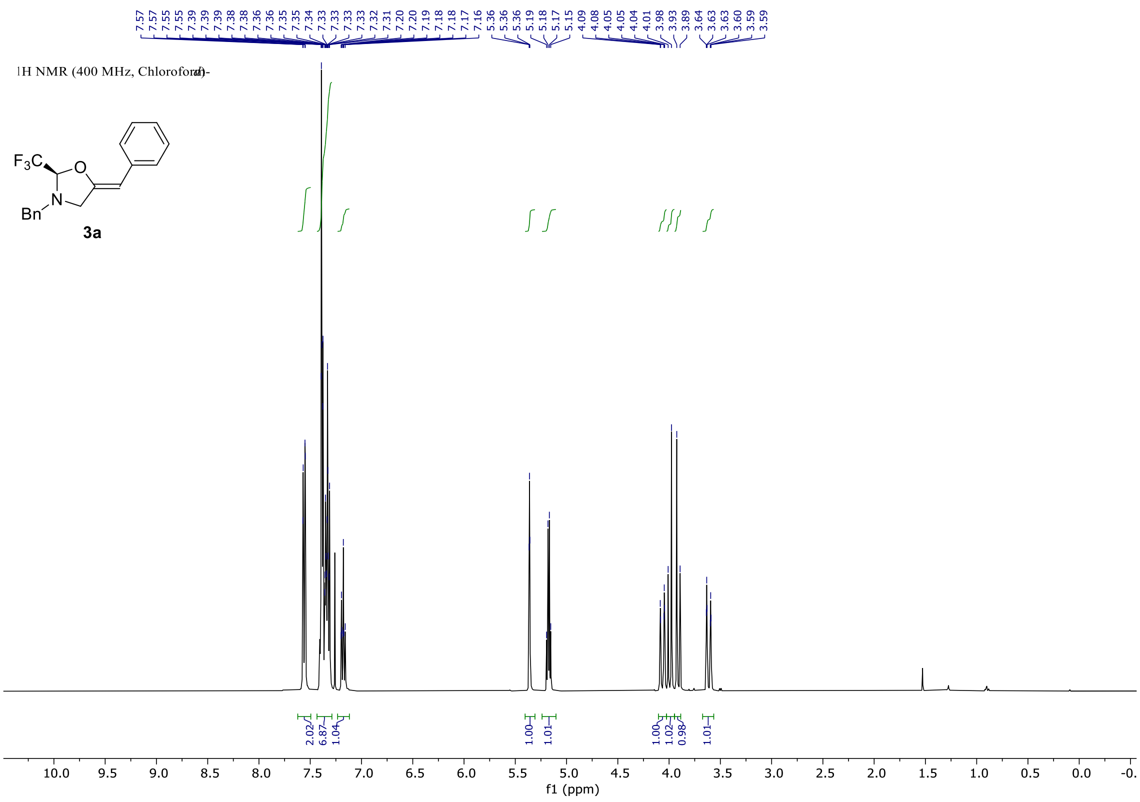

13C NMR (101 MHz, Chloroform-*d*)

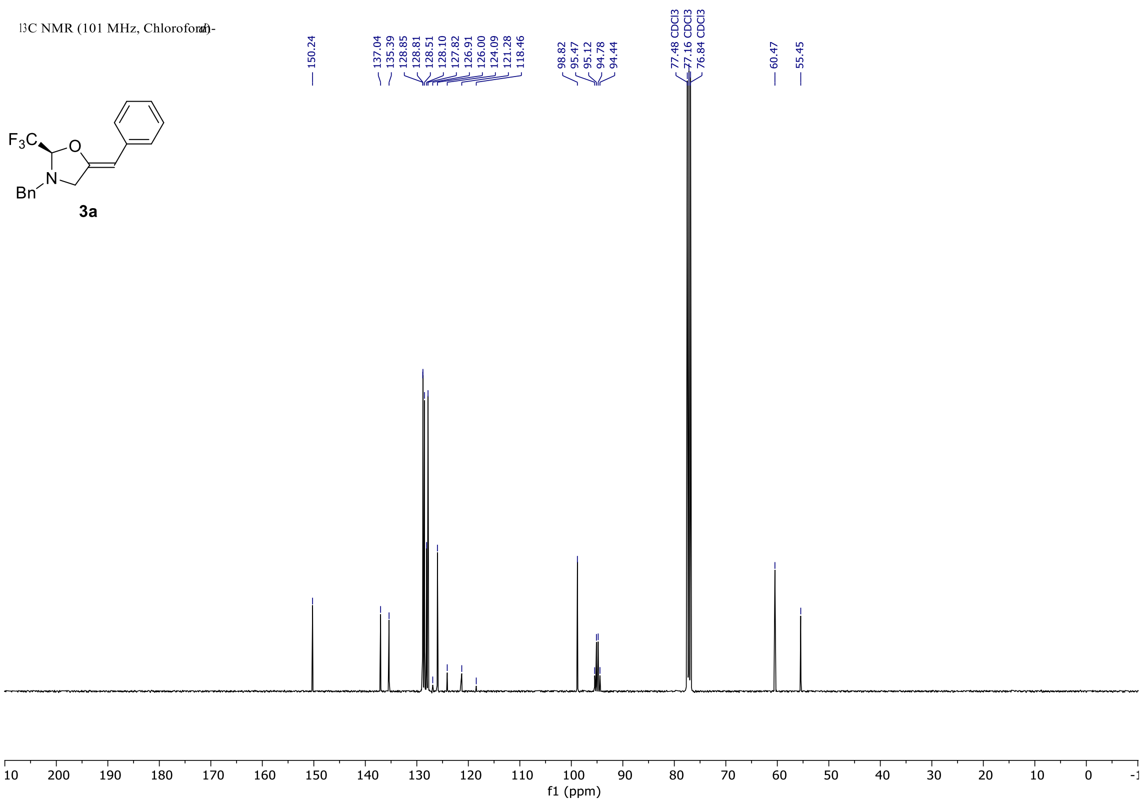

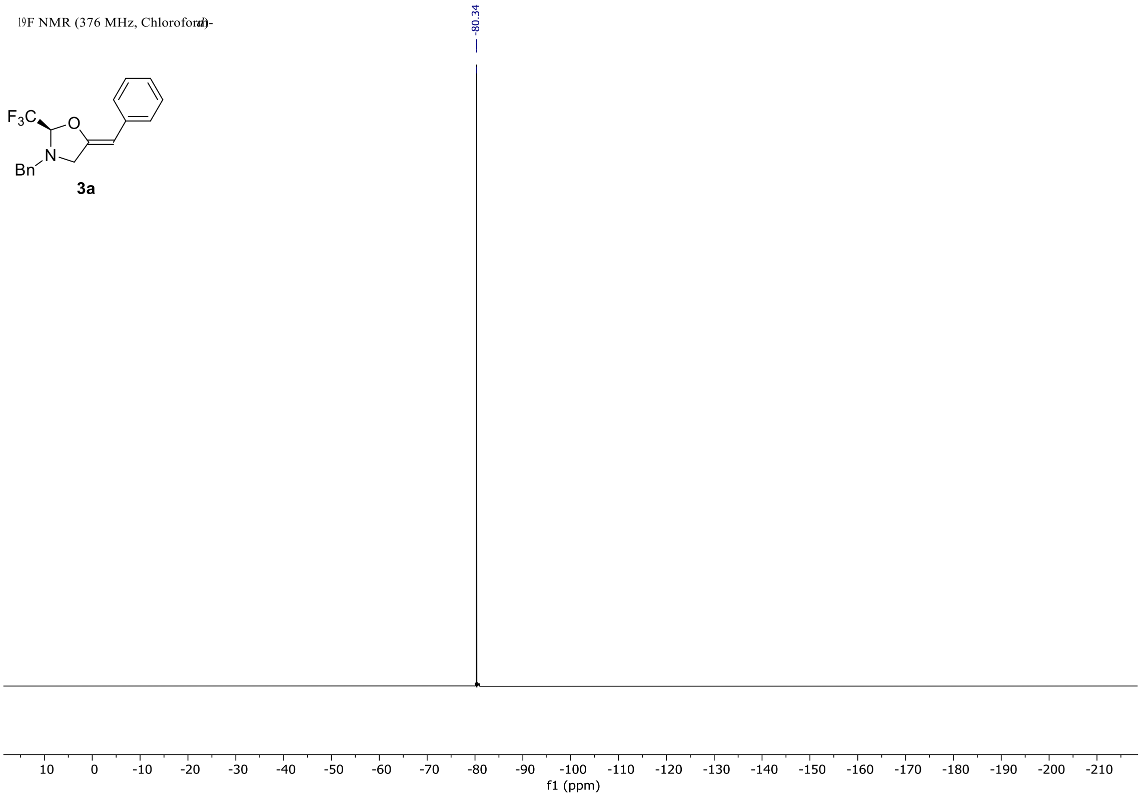
19F NMR (376 MHz, Chloroform-d)
-80.34
F3C
O
N
Bn
3a
f1 (ppm)

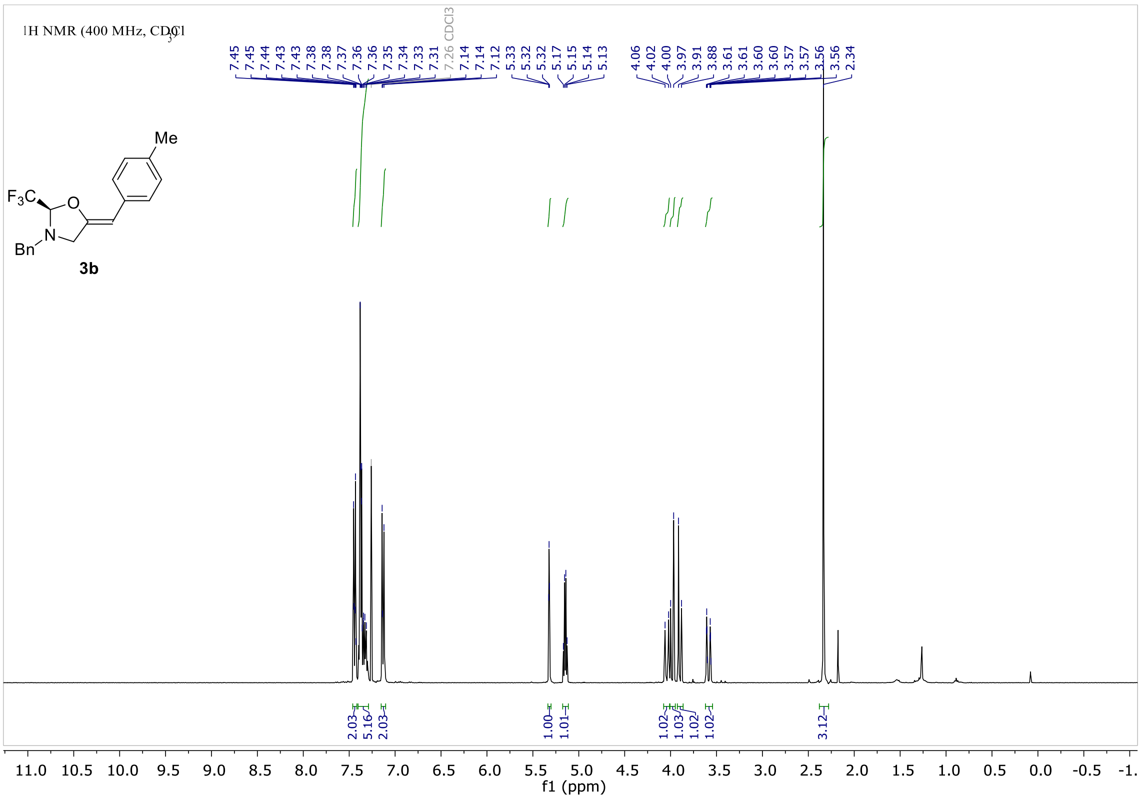
1H NMR (400 MHz, CDCl3)
7.45 7.45 7.44 7.43 7.43 7.38 7.38 7.37 7.36 7.36 7.35 7.34 7.33 7.31 7.26 CDCl3 7.14 7.14 7.12 5.33 5.32 5.17 5.15 5.14 5.13 4.06 4.02 4.00 3.97 3.91 3.88 3.61 3.61 3.60 3.57 3.57 3.56 2.34
Me
F3C
O
N
Bn
3b
2.03 5.16 2.03 1.00 1.01 1.02 1.03 1.02 1.02 3.12
f1 (ppm)

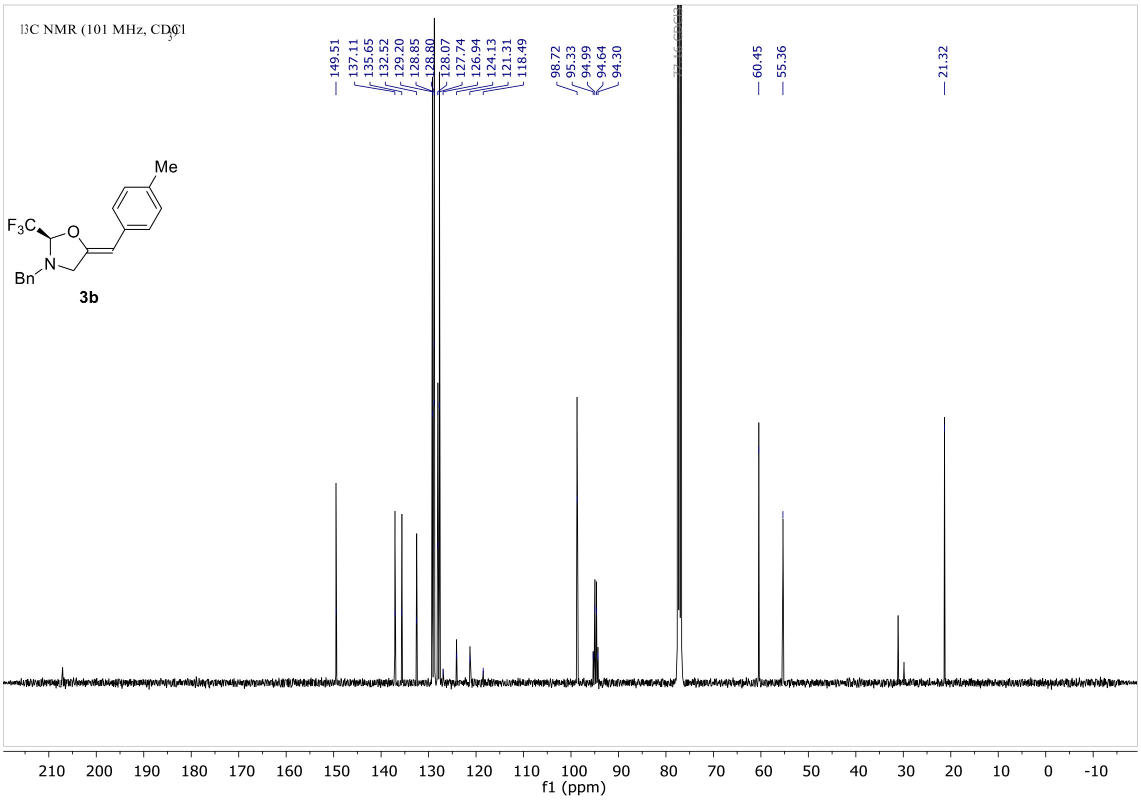
13C NMR (101 MHz, CDCl3
149.51
137.11
135.65
132.52
129.20
128.85
128.80
128.07
127.74
126.94
124.13
121.31
118.49
98.72
95.33
94.99
94.64
94.30
60.45
55.36
21.32
Me
F3C
O
N
Bn
3b
210 200 190 180 170 160 150 140 130 120 110 100 90 80 70 60 50 40 30 20 10 0 -10
f1 (ppm)

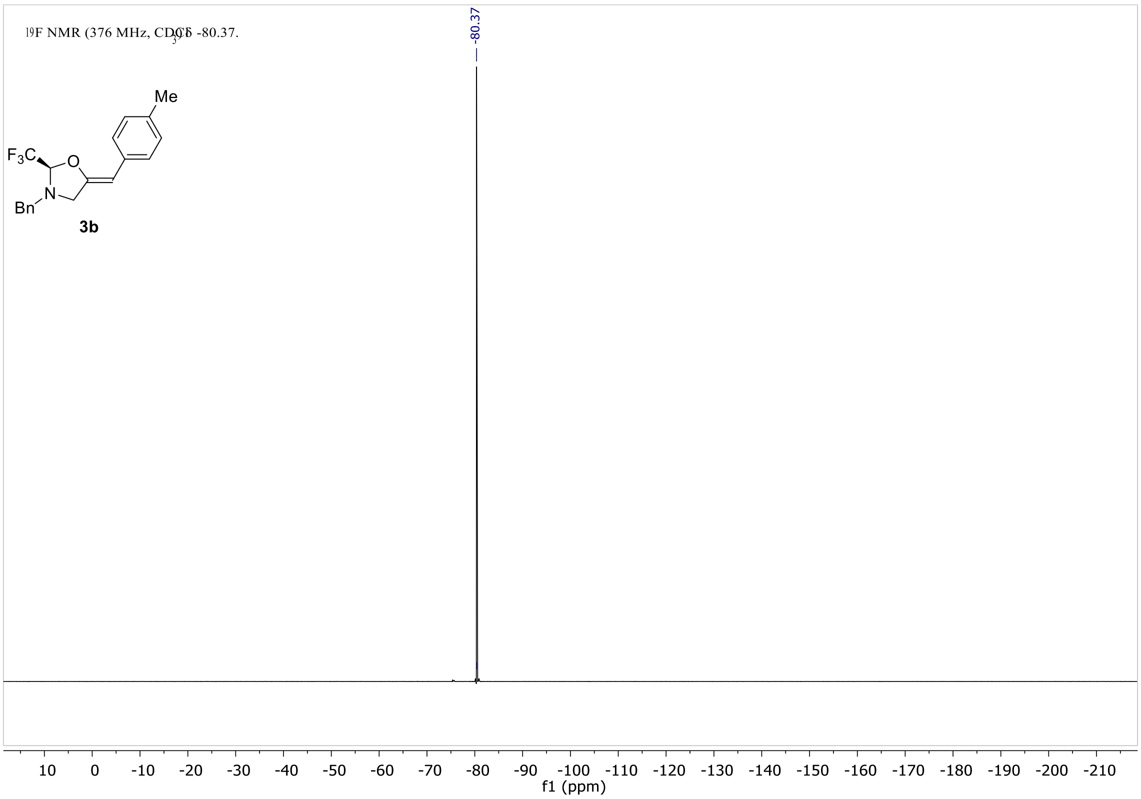
19F NMR (376 MHz, CDCl3) δ -80.37.
-80.37
Me
F3C
O
N
Bn
3b
10 0 -10 -20 -30 -40 -50 -60 -70 -80 -90 -100 -110 -120 -130 -140 -150 -160 -170 -180 -190 -200 -210
f1 (ppm)

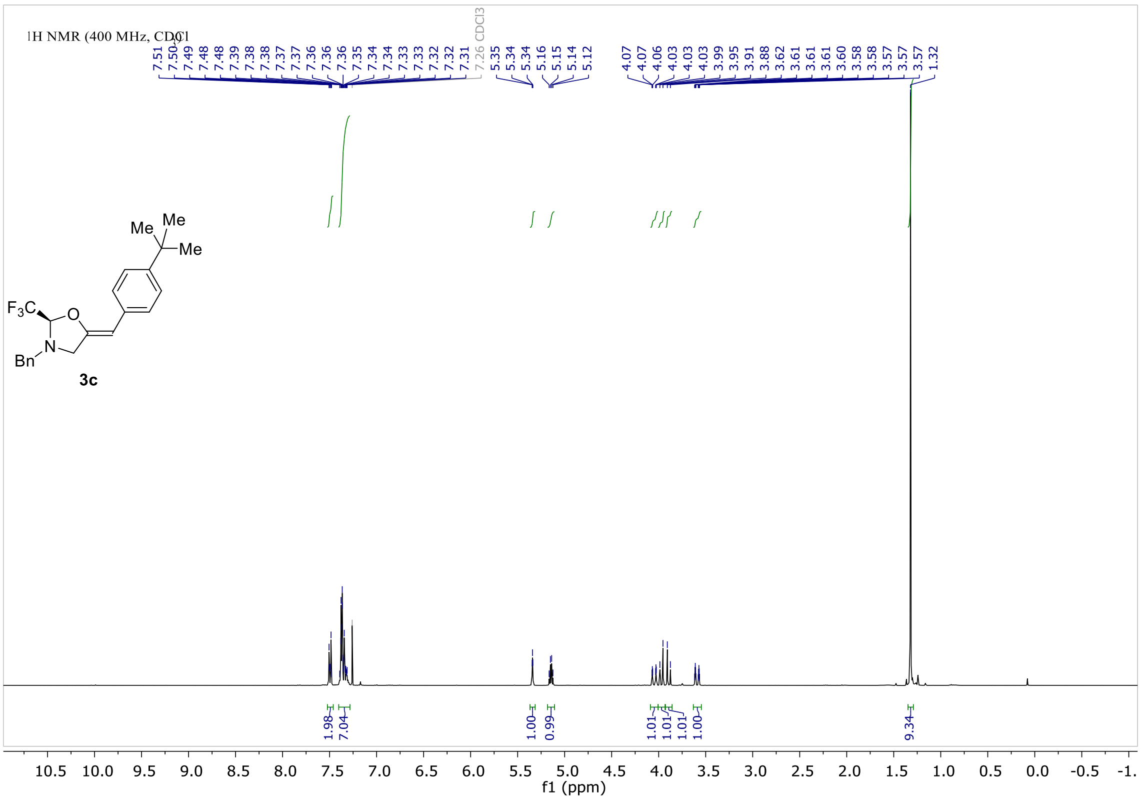
1H NMR (400 MHz, CDCl3)
7.51
7.50
7.49
7.48
7.48
7.39
7.38
7.38
7.37
7.37
7.36
7.36
7.36
7.35
7.34
7.34
7.33
7.33
7.32
7.32
7.31
7.26 CDCl3
5.35
5.34
5.34
5.16
5.15
5.14
5.12
4.07
4.07
4.06
4.03
4.03
4.03
3.99
3.95
3.91
3.88
3.62
3.61
3.61
3.60
3.58
3.58
3.57
3.57
1.32
Me
Me
Me
F3C
O
N
Bn
3c
1.98
7.04
1.00
0.99
1.01
1.01
1.01
1.00
9.34
f1 (ppm)

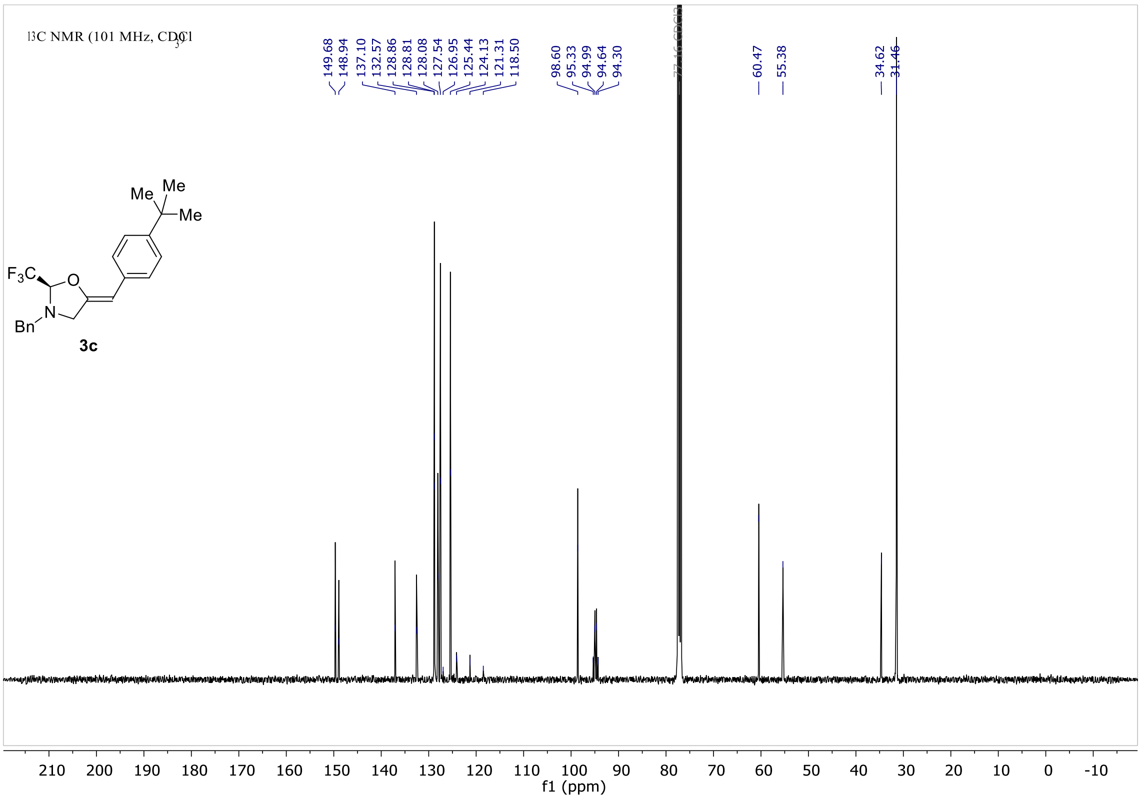
13C NMR (101 MHz, CDCl3)
149.68
148.94
137.10
132.57
128.86
128.81
128.08
127.54
126.95
125.44
124.13
121.31
118.50
98.60
95.33
94.99
94.64
94.30
77.16 CDCl3
60.47
55.38
34.62
31.46
Me
Me
Me
F3C
O
N
Bn
3c
f1 (ppm)

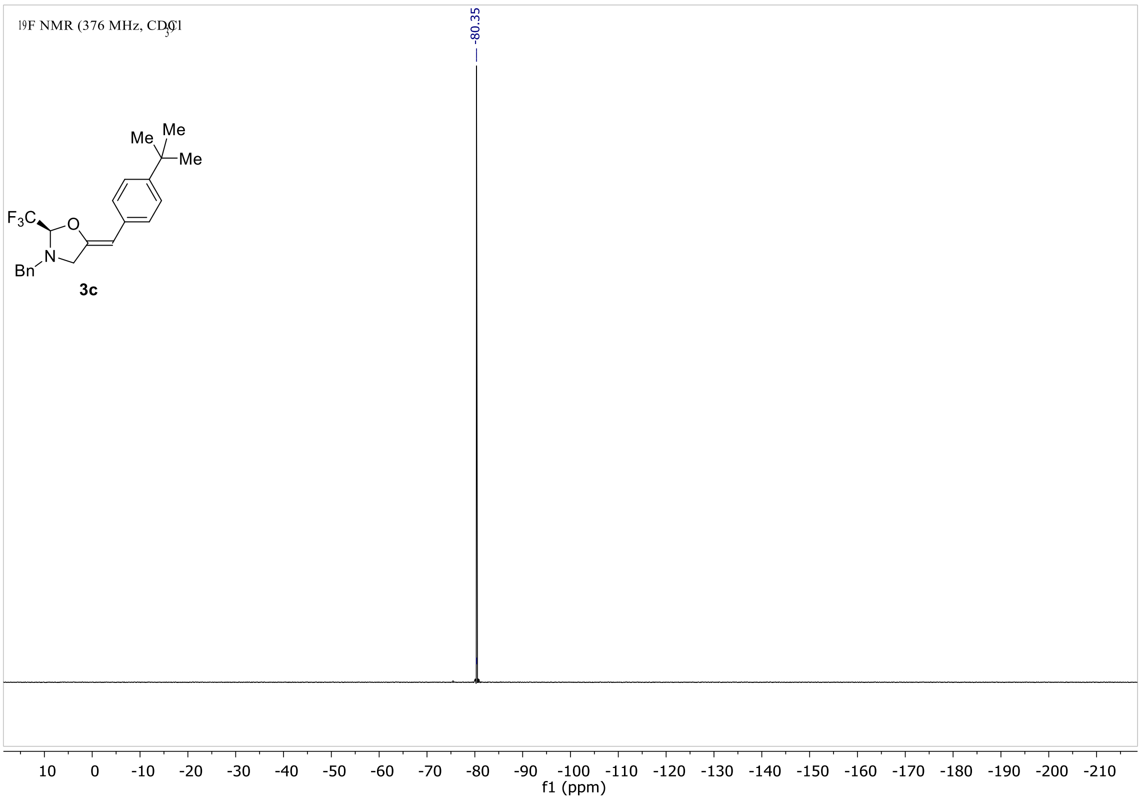
19F NMR (376 MHz, CDCl3
-80.35
Me
Me
Me
F3C
O
N
Bn
3c
f1 (ppm)

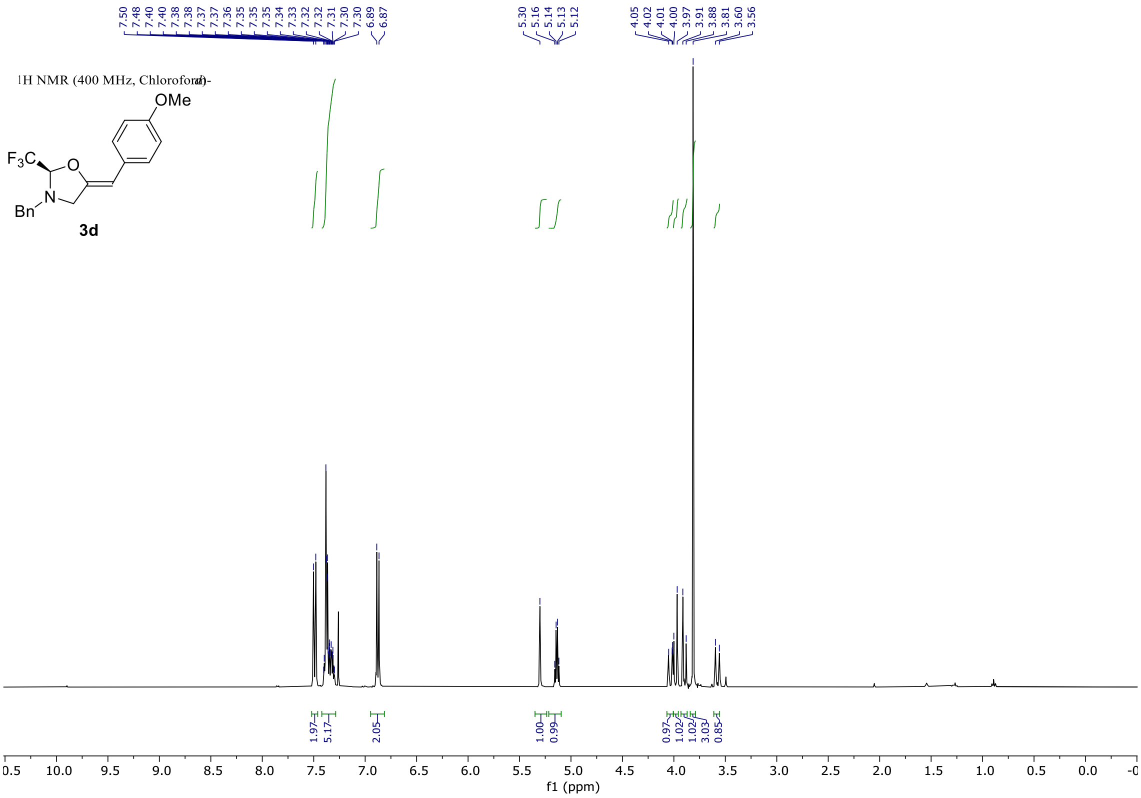
1H NMR (400 MHz, Chloroform-d)
7.50 7.48 7.40 7.40 7.38 7.38 7.37 7.37 7.36 7.35 7.35 7.35 7.34 7.33 7.32 7.32 7.31 7.30 7.30 6.89 6.87
5.30 5.16 5.14 5.13 5.12
4.05 4.02 4.01 4.00 3.97 3.91 3.88 3.81 3.60 3.56
OMe
F3C
O
N
Bn
3d
1.97 5.17 2.05 1.00 0.99 0.97 1.02 1.02 3.03 0.85
f1 (ppm)

^{13}C NMR (101 MHz, Chloroform-*d*)

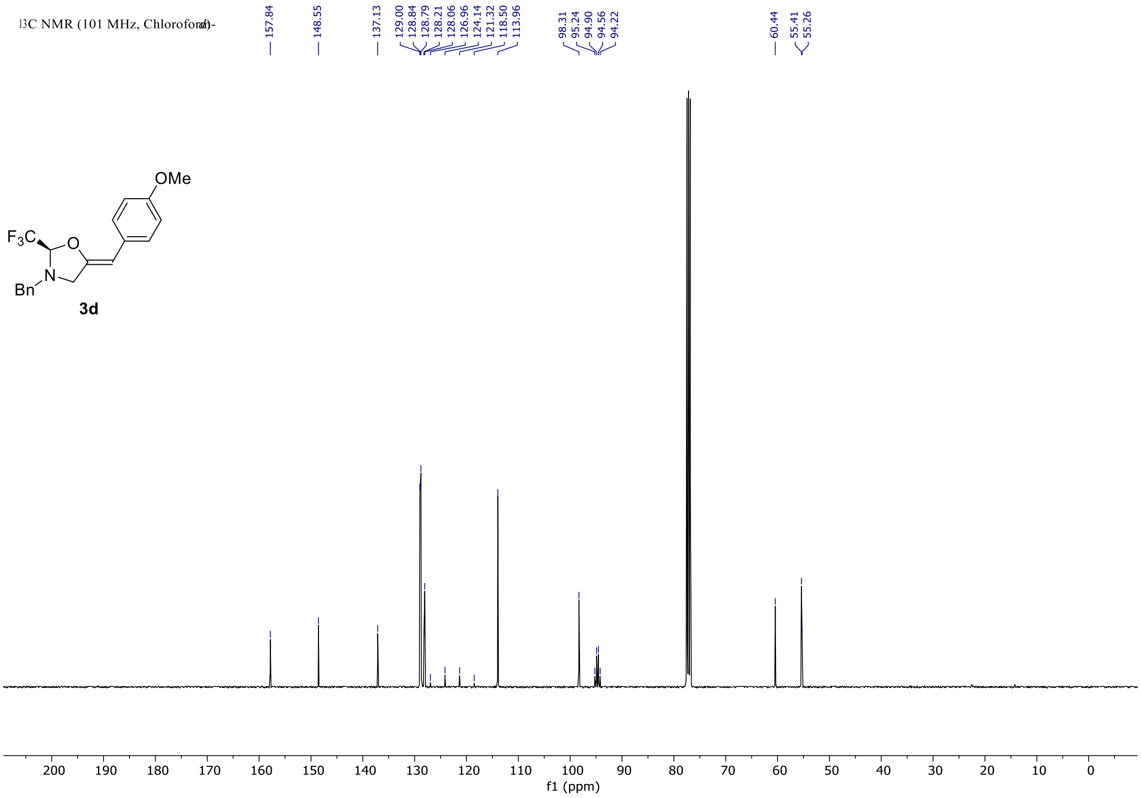

^{19}F NMR (376 MHz, Chloroform-*d*)

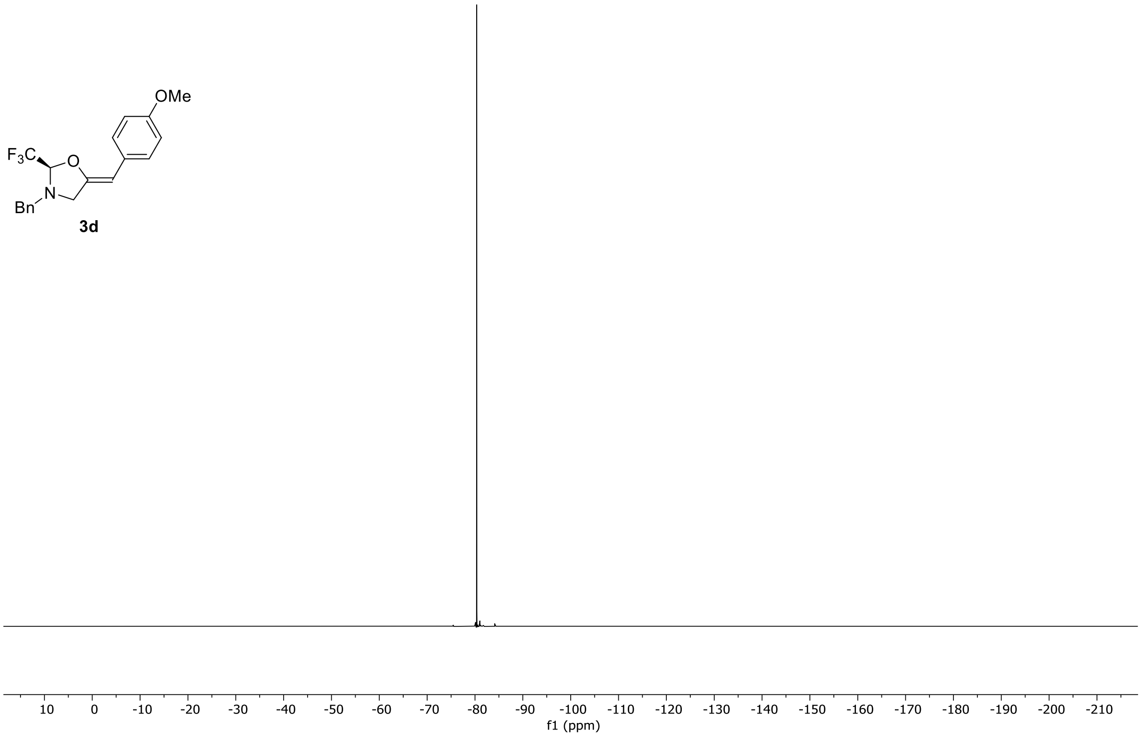

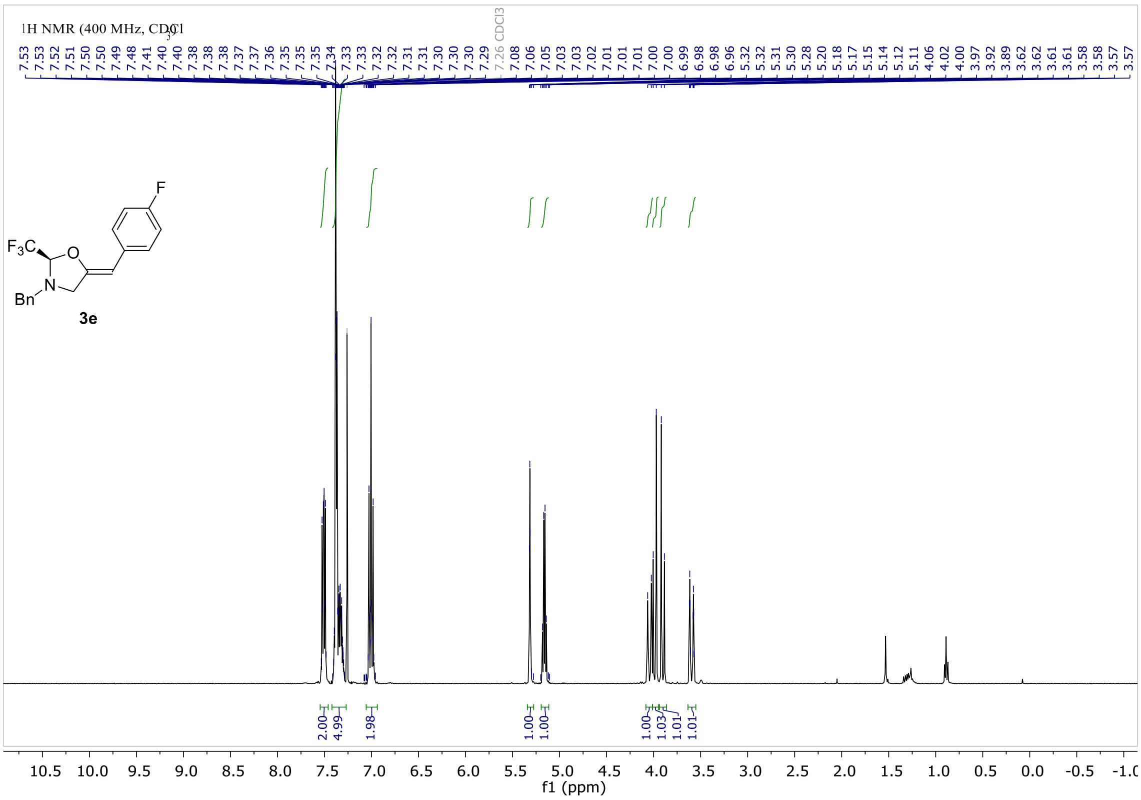

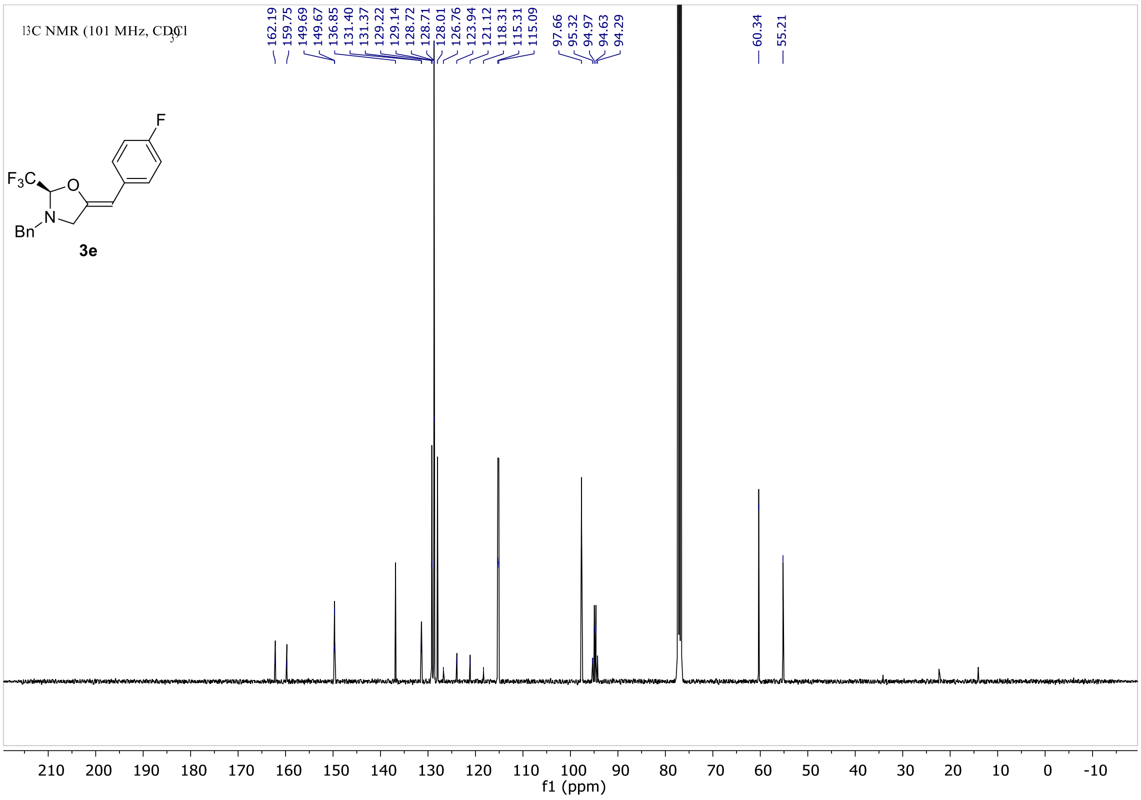

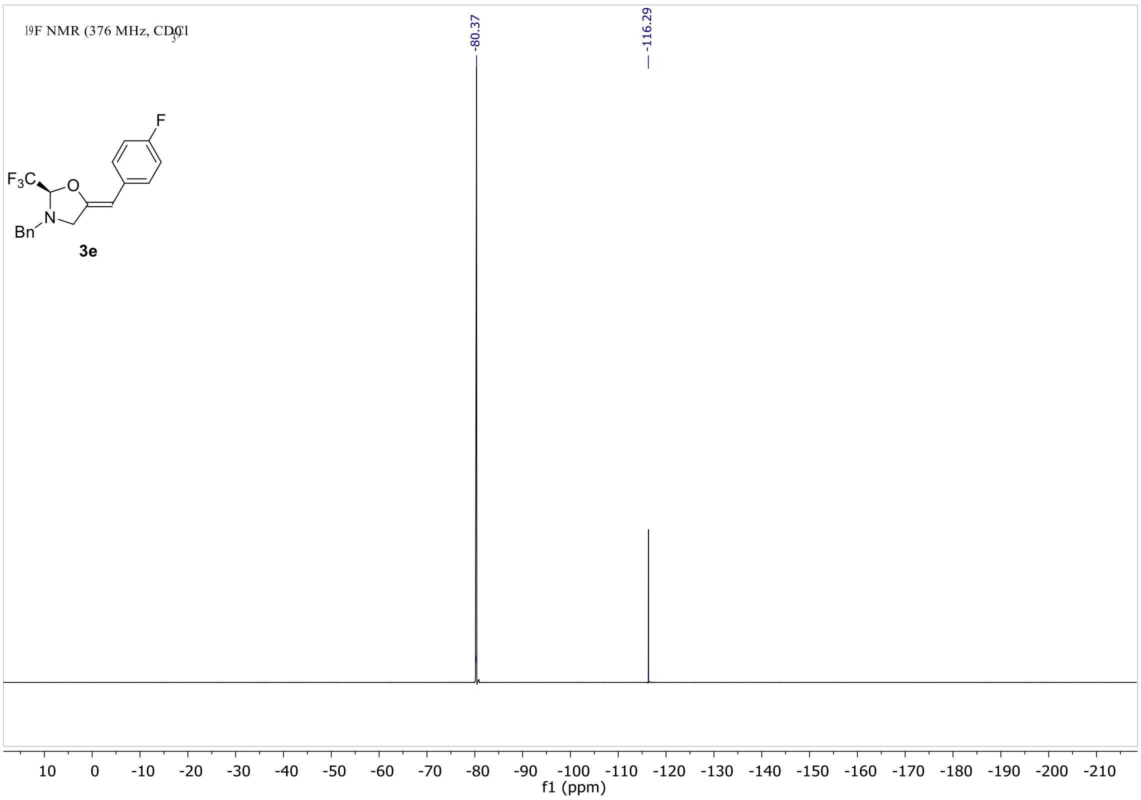
19F NMR (376 MHz, CDCl3)
-80.37
-116.29
3e
f1 (ppm)

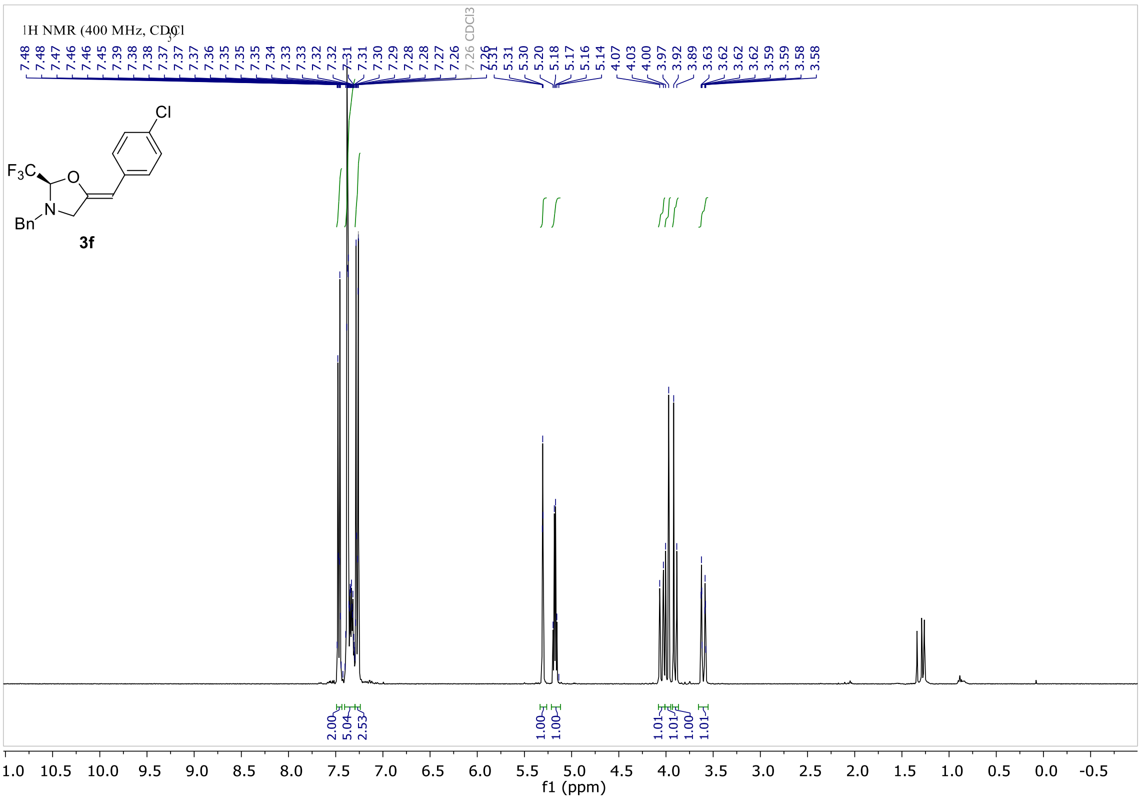
1H NMR (400 MHz, CDCl3)
7.26 CDCl3
3f
f1 (ppm)

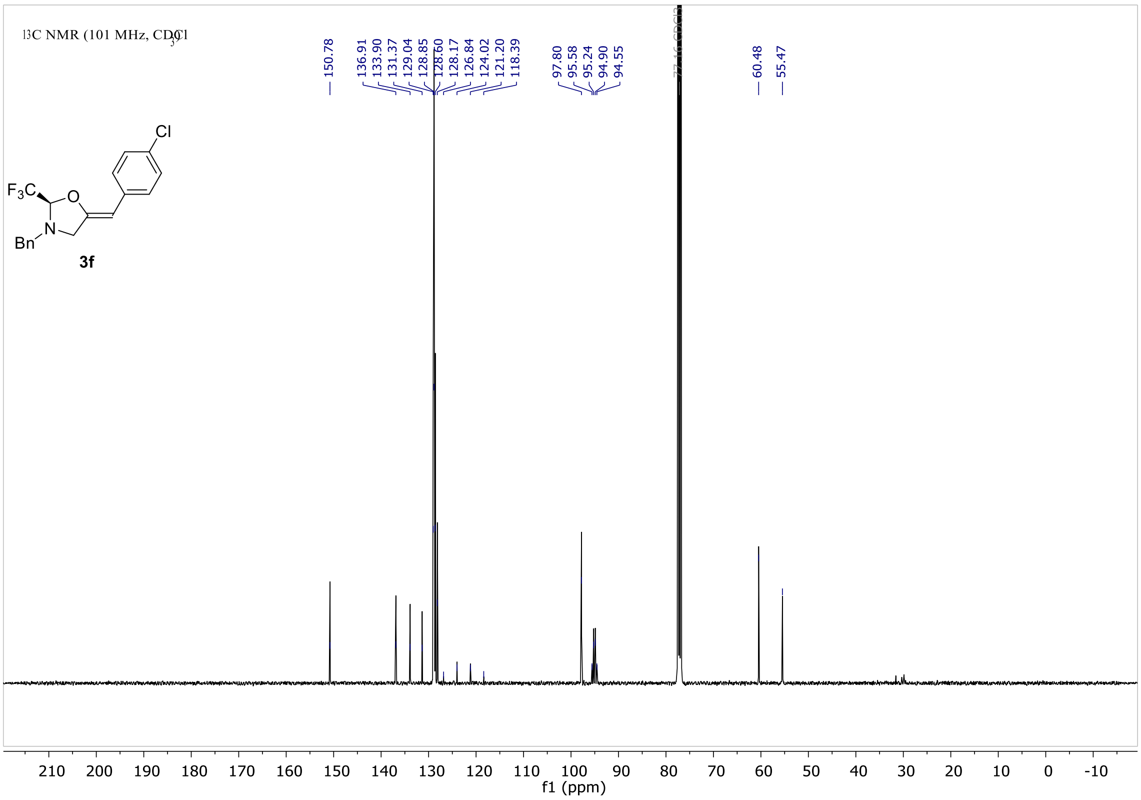
13C NMR (101 MHz, CDCl3)
150.78
136.91
133.90
131.37
129.04
128.85
128.60
128.17
126.84
124.02
121.20
118.39
97.80
95.58
95.24
94.90
94.55
60.48
55.47
Cl
F3C
O
N
Bn
3f
210 200 190 180 170 160 150 140 130 120 110 100 90 80 70 60 50 40 30 20 10 0 -10
f1 (ppm)

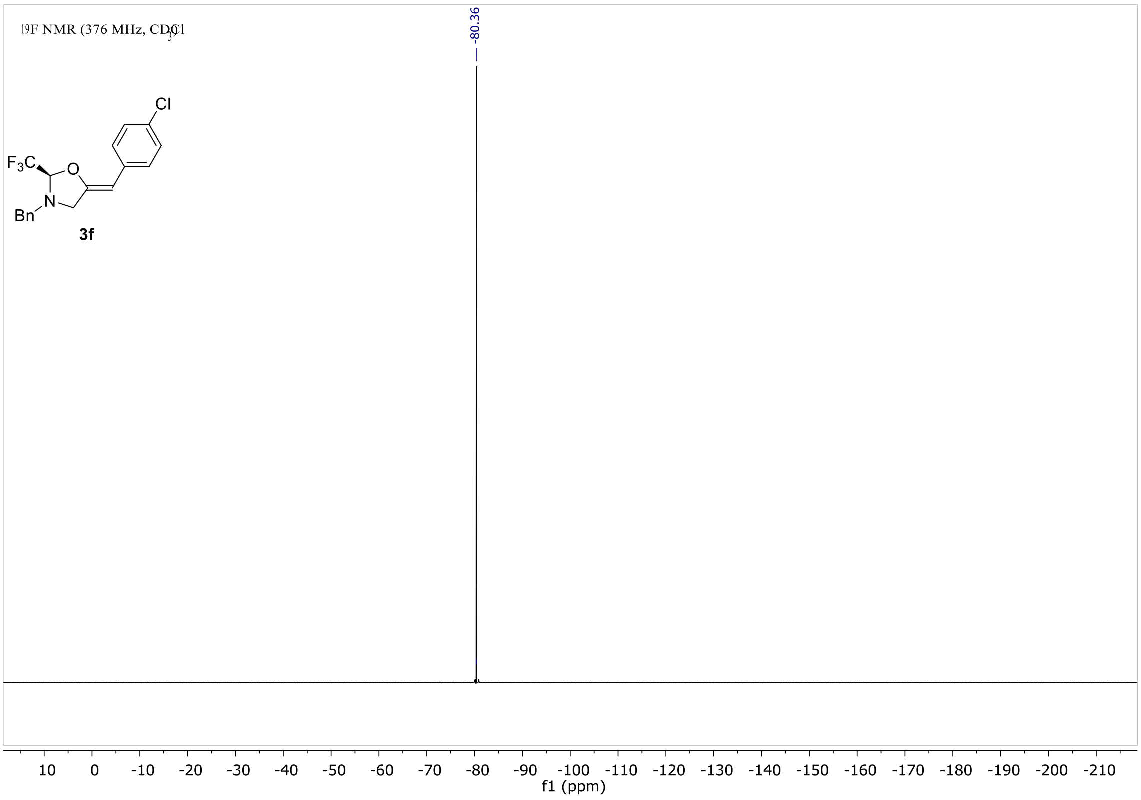
19F NMR (376 MHz, CDCl3)
-80.36
Cl
F3C
O
N
Bn
3f
10 0 -10 -20 -30 -40 -50 -60 -70 -80 -90 -100 -110 -120 -130 -140 -150 -160 -170 -180 -190 -200 -210
f1 (ppm)

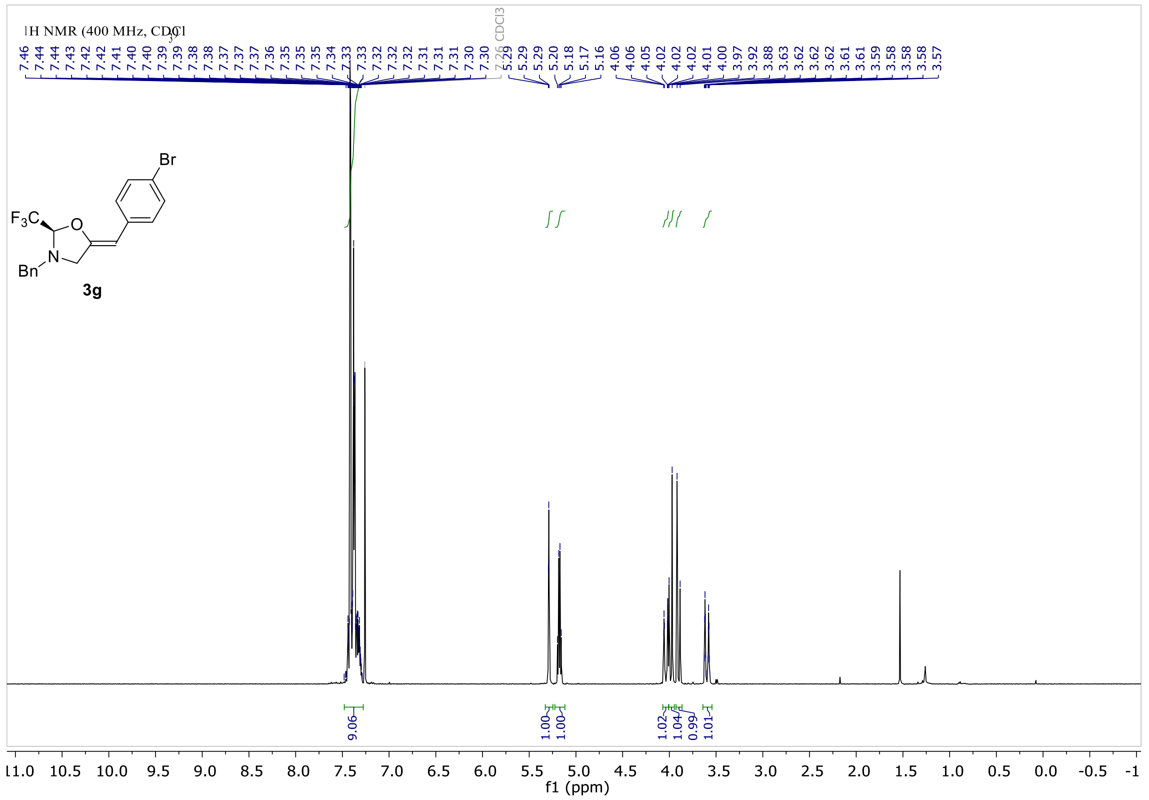

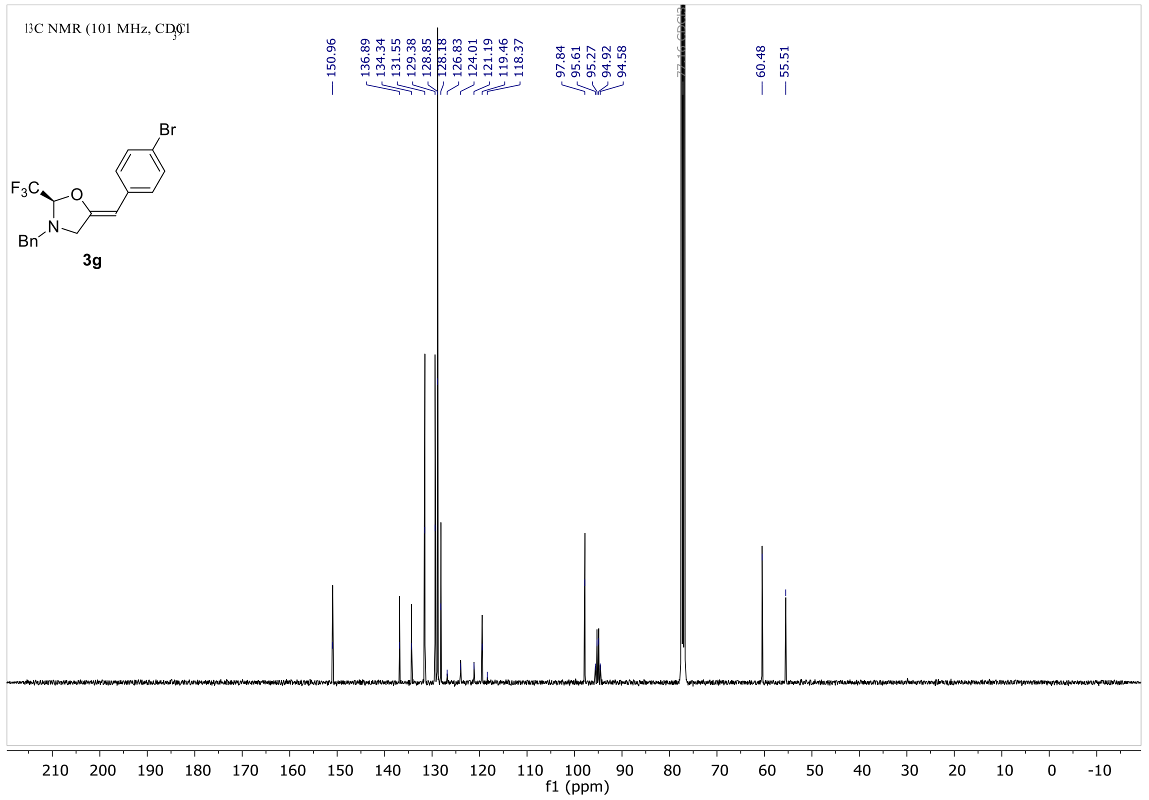

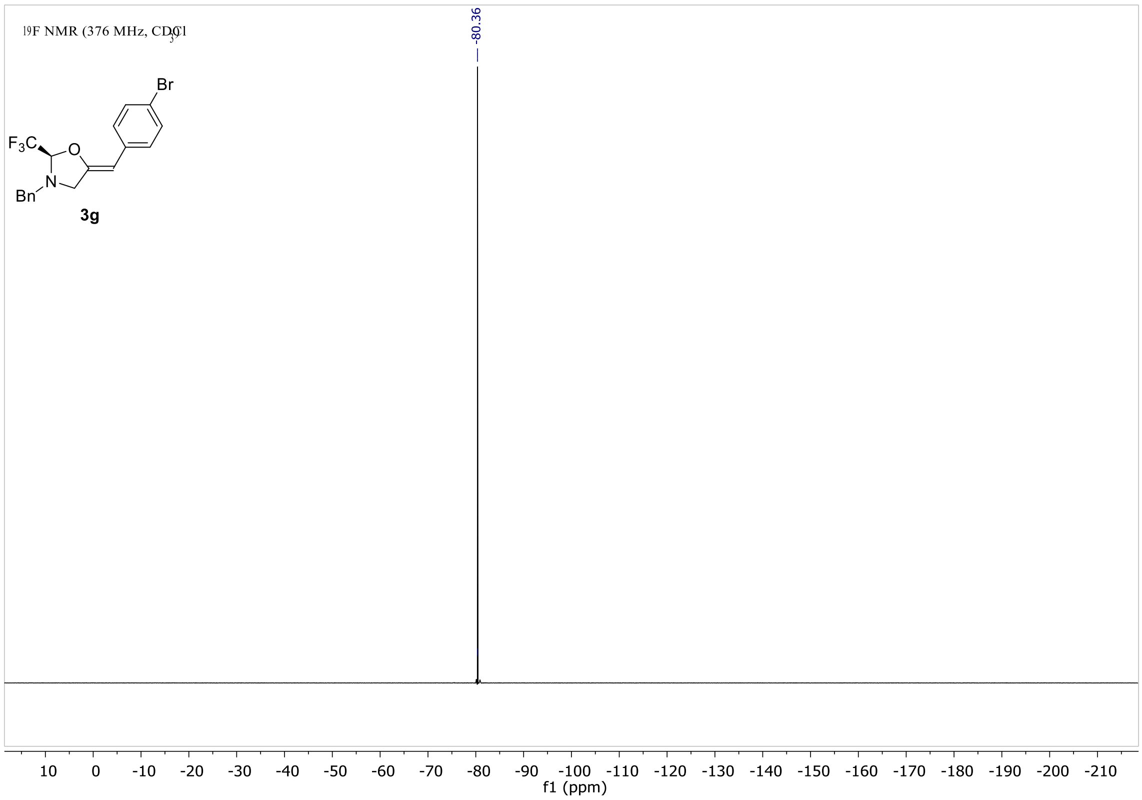
19F NMR (376 MHz, CDCl3)
-80.36
Br
F3C
O
N
Bn
3g
f1 (ppm)

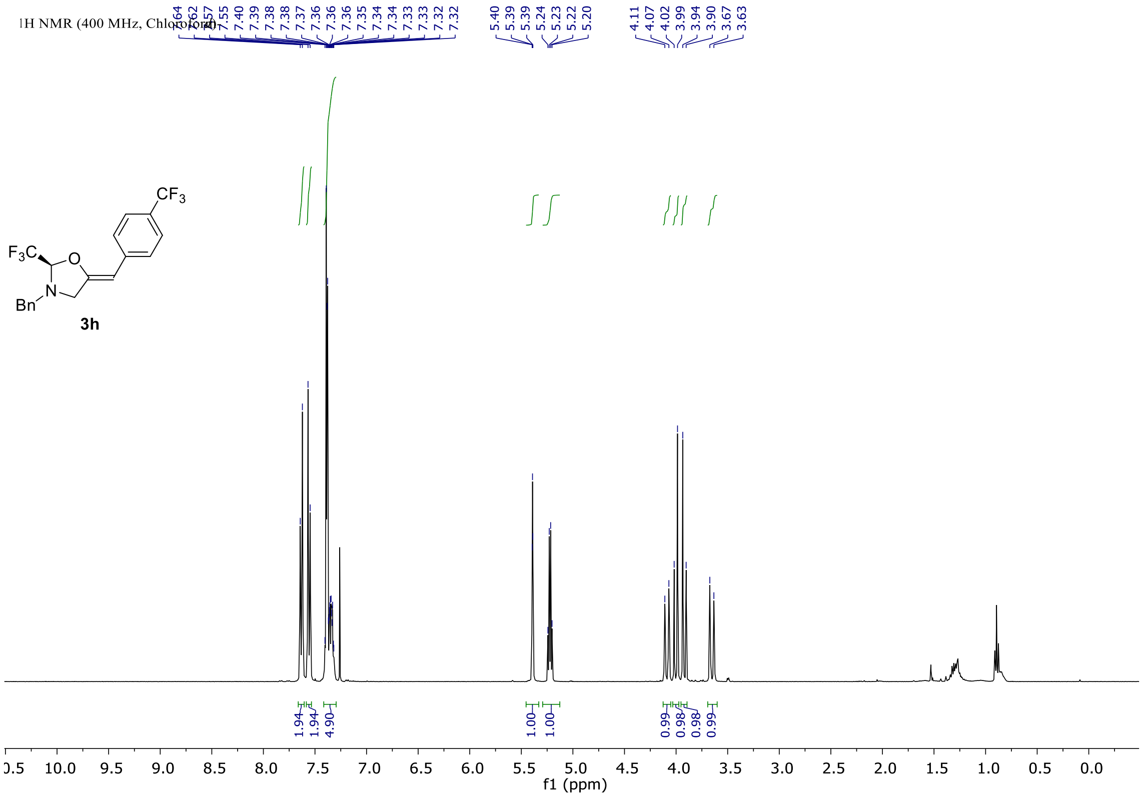
1H NMR (400 MHz, Chloroform)
CF3
F3C
O
N
Bn
3h
f1 (ppm)

^{13}C NMR (101 MHz, Chloroform-d)

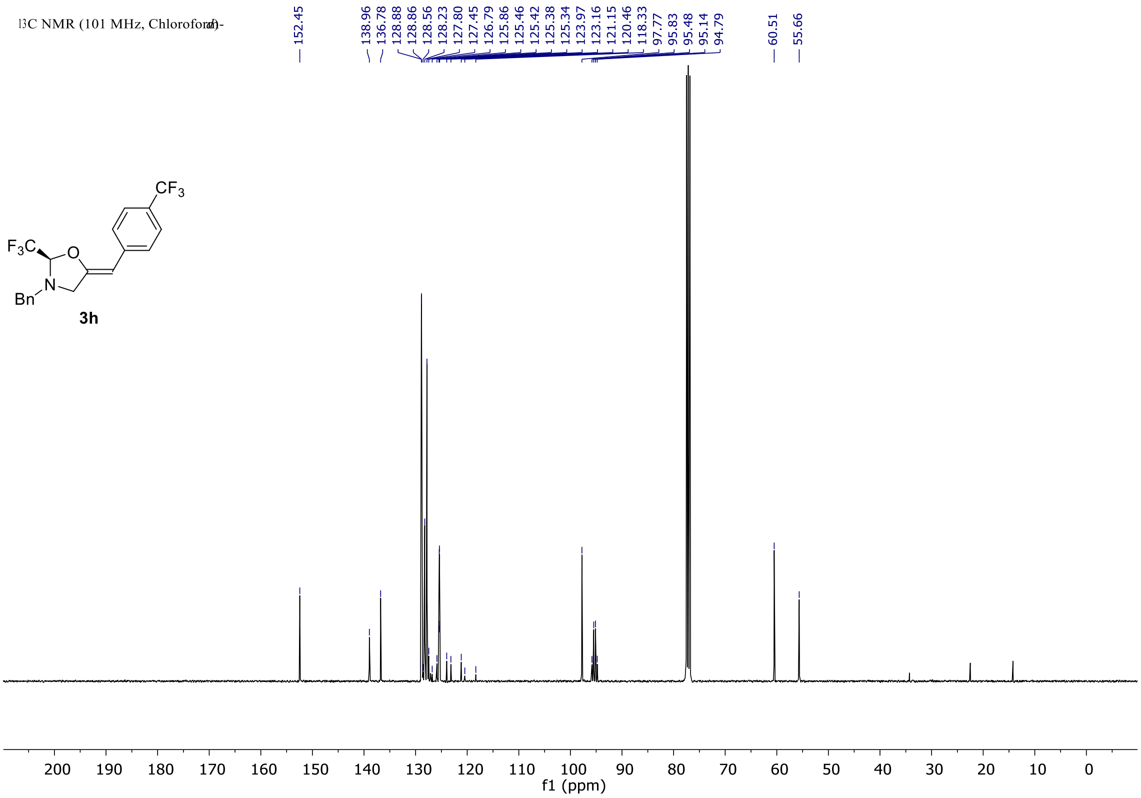

^{19}F NMR (376 MHz, Chloroform-d)

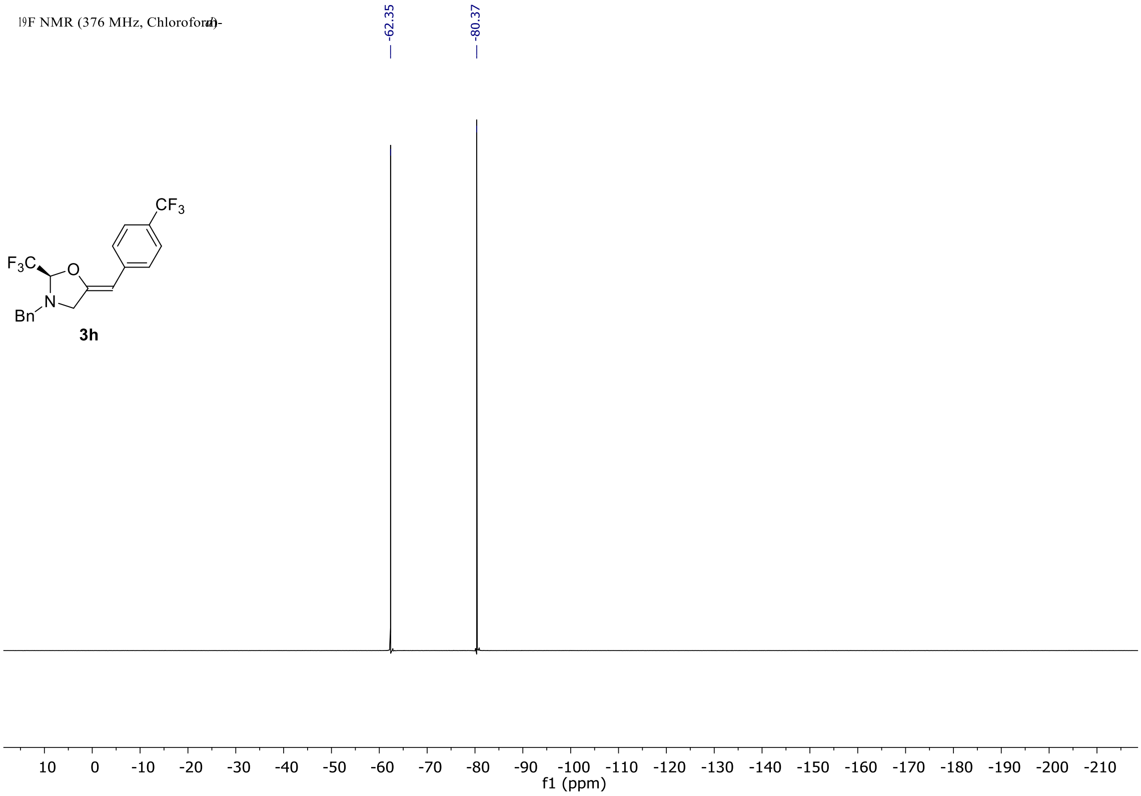

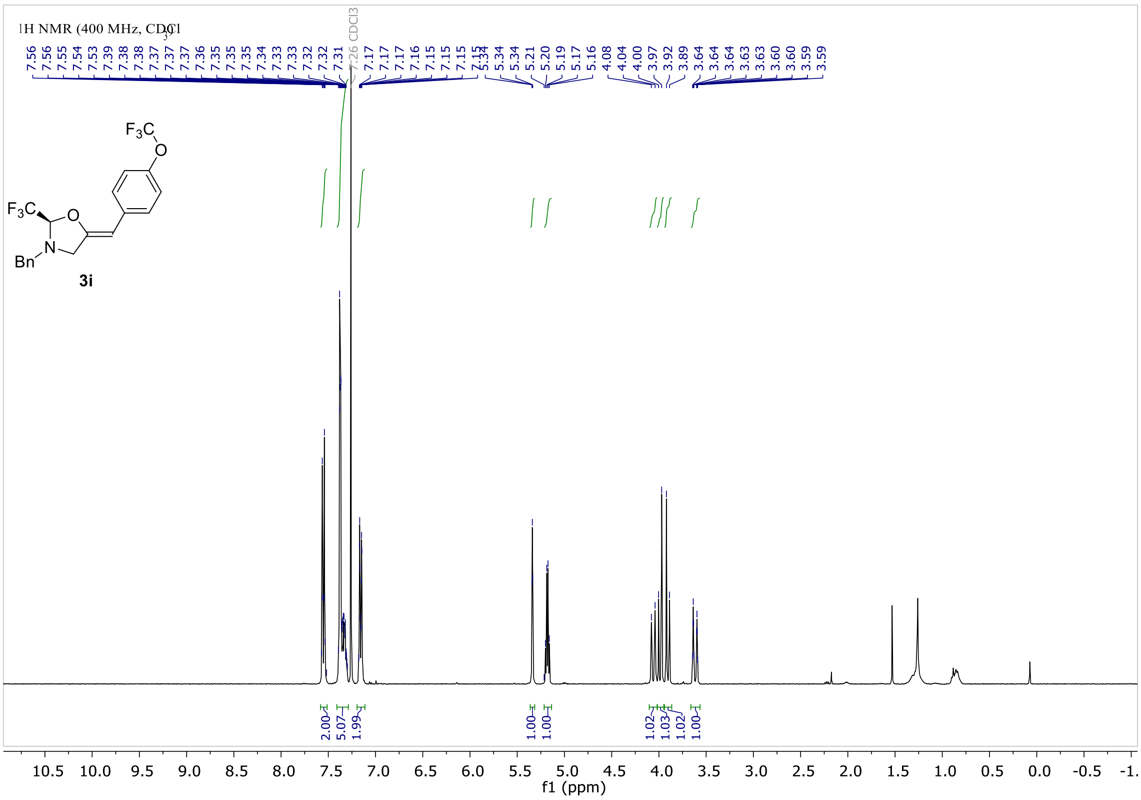
1H NMR (400 MHz, CDCl3)
7.56 7.56 7.55 7.54 7.53 7.39 7.38 7.38 7.37 7.37 7.37 7.36 7.35 7.35 7.35 7.34 7.33 7.33 7.32 7.31 7.26 CDCl3 7.17 7.17 7.17 7.16 7.15 7.15 7.15 7.15 5.35 5.34 5.34 5.21 5.20 5.19 5.17 5.16 4.08 4.04 4.00 3.97 3.92 3.89 3.64 3.64 3.64 3.63 3.60 3.60 3.59 3.59
2.00 5.07 1.99 1.00 1.00 1.02 1.03 1.02 1.00
3i
f1 (ppm)

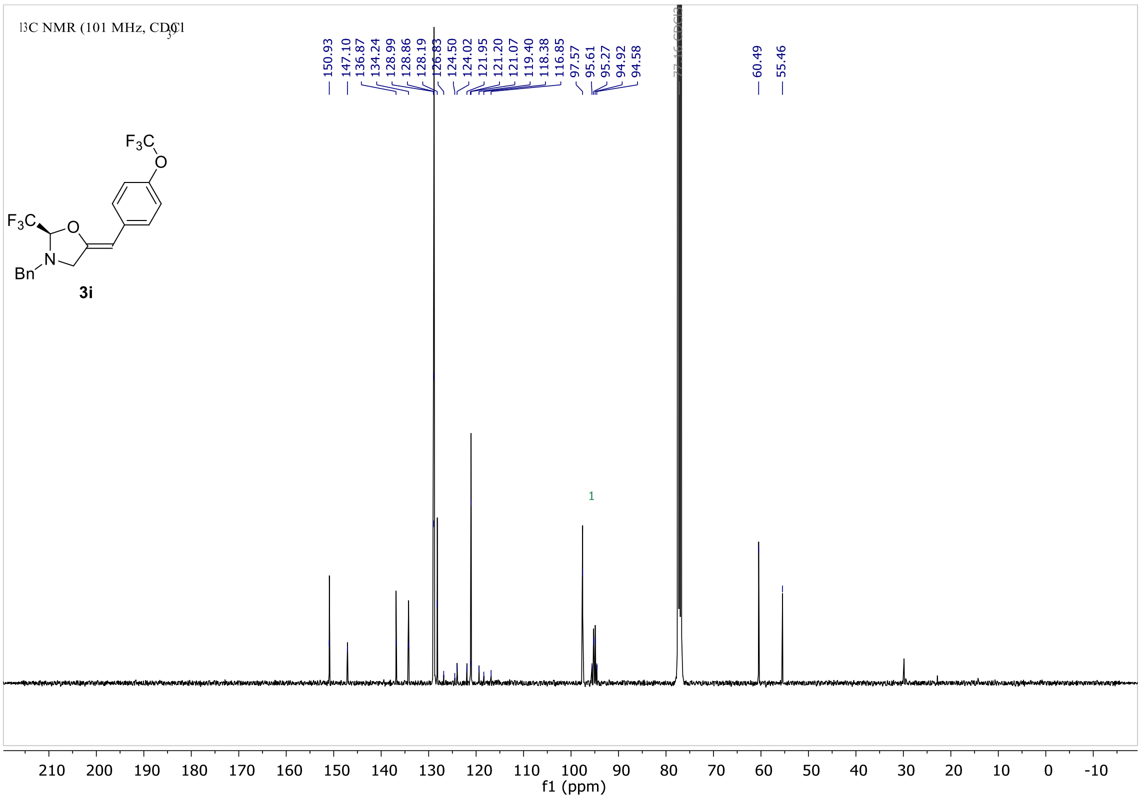
13C NMR (101 MHz, CDCl3)
150.93 147.10 136.87 134.24 128.99 128.86 128.19 126.83 124.50 124.02 121.95 121.20 121.07 119.40 118.38 116.85 97.57 95.61 95.27 94.92 94.58 77.16 CDCl3 60.49 55.46
3i
f1 (ppm)

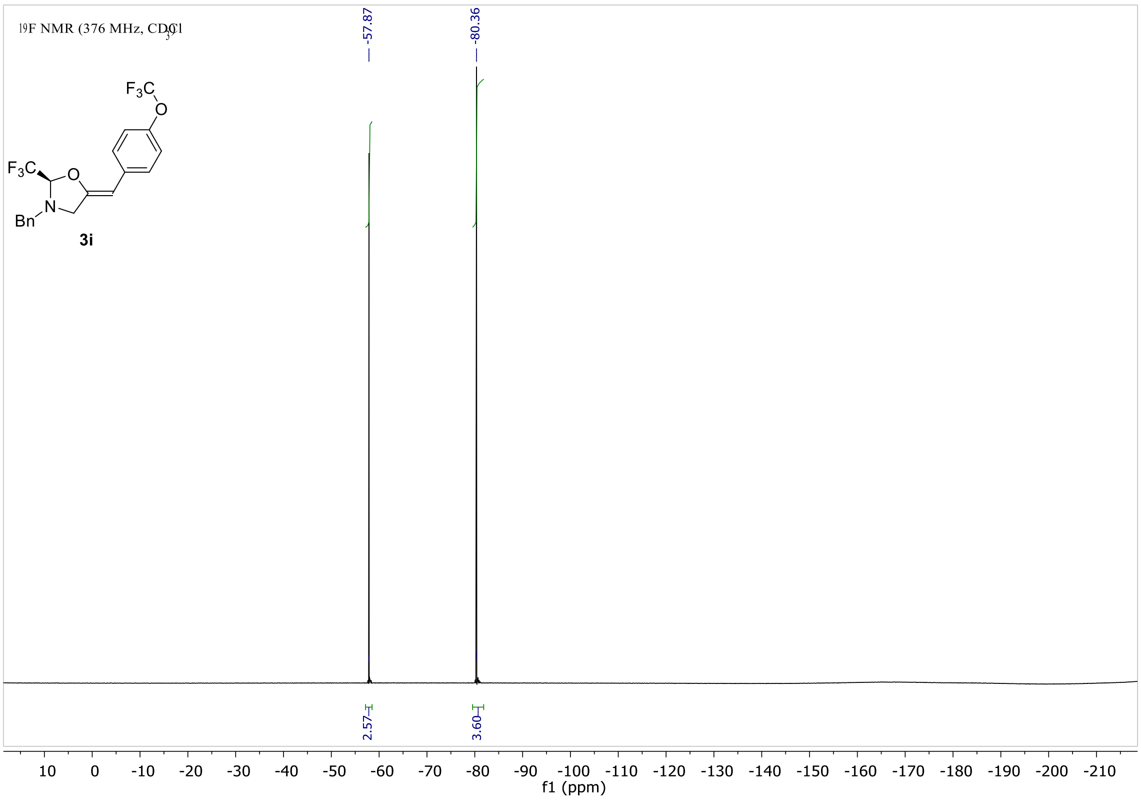
19F NMR (376 MHz, CDCl3)
F3C
O
F3C
O
N
Bn
3i
-57.87
-80.36
2.57
3.60
10 0 -10 -20 -30 -40 -50 -60 -70 -80 -90 -100 -110 -120 -130 -140 -150 -160 -170 -180 -190 -200 -210
f1 (ppm)

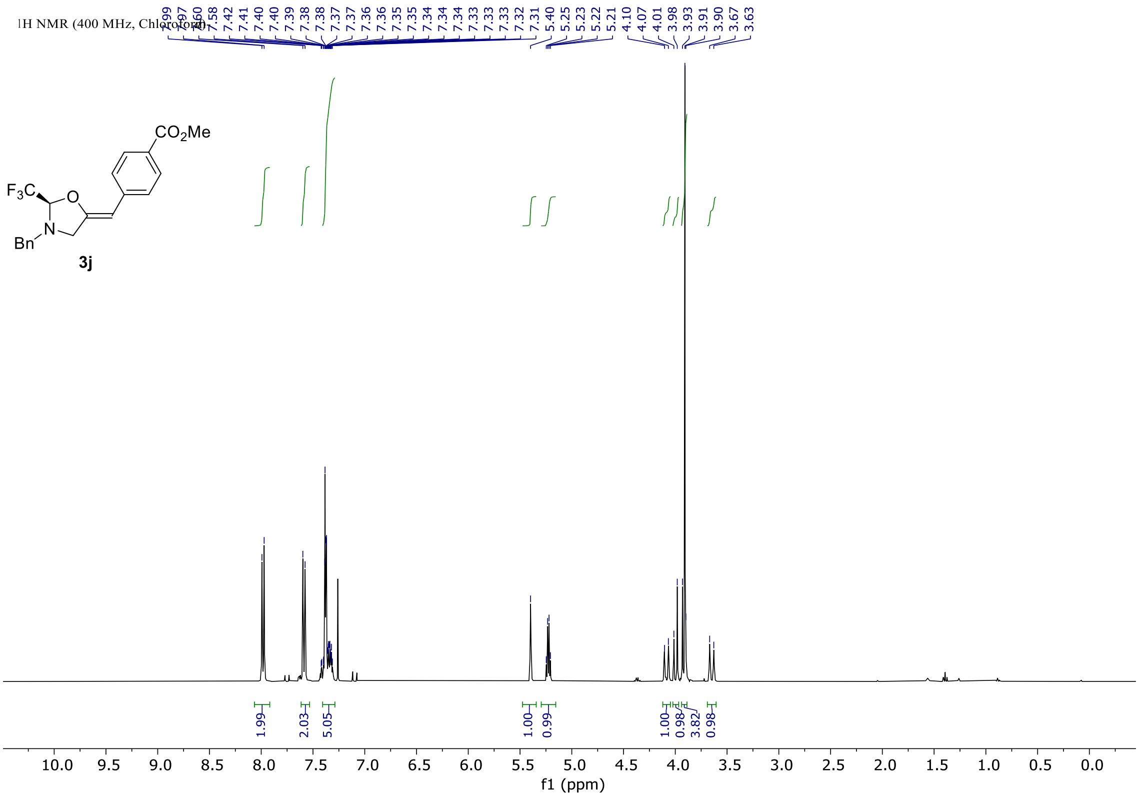
1H NMR (400 MHz, Chloroform-d)
CO2Me
F3C
O
N
Bn
3j
7.97
7.60
7.58
7.42
7.41
7.40
7.40
7.39
7.38
7.38
7.37
7.37
7.36
7.36
7.35
7.34
7.34
7.33
7.33
7.33
7.32
7.31
5.40
5.25
5.23
5.22
5.21
4.10
4.07
4.01
3.98
3.93
3.91
3.90
3.67
3.63
1.99
2.03
5.05
1.00
0.99
1.00
0.98
3.82
0.98
10.0 9.5 9.0 8.5 8.0 7.5 7.0 6.5 6.0 5.5 5.0 4.5 4.0 3.5 3.0 2.5 2.0 1.5 1.0 0.5 0.0
f1 (ppm)

13C NMR (101 MHz, Chloroform-d)

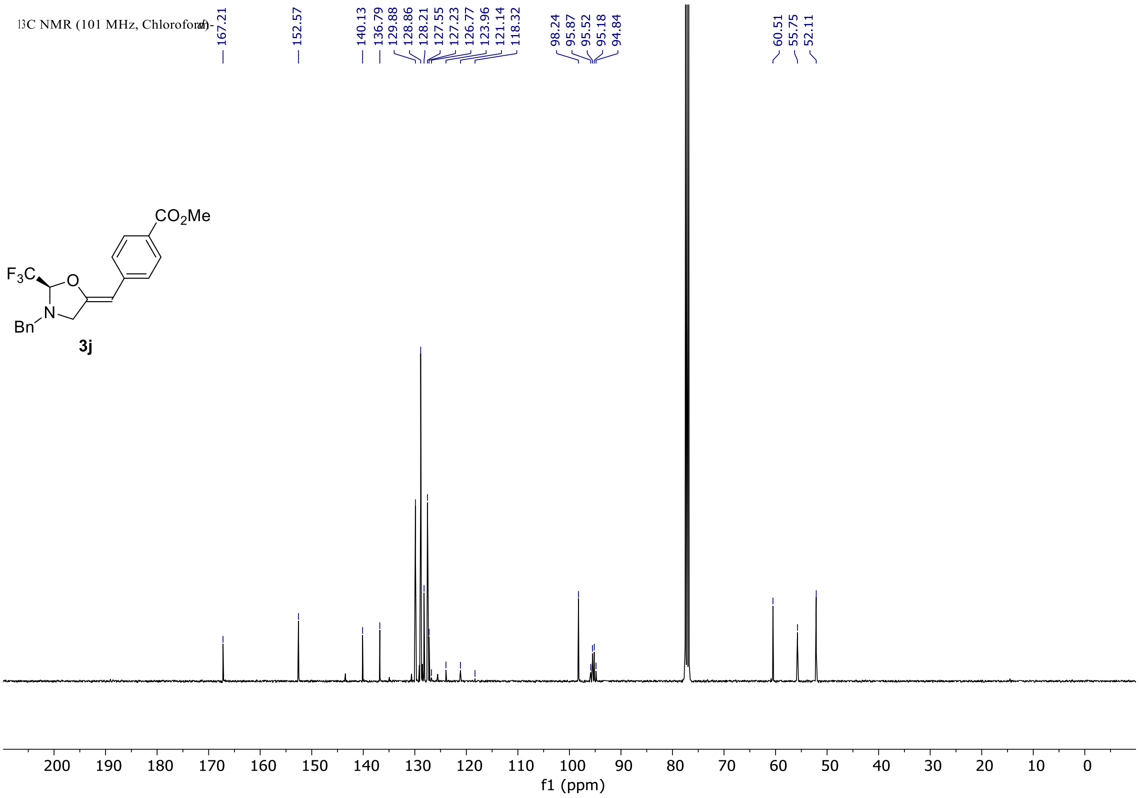

19F NMR (376 MHz, Chloroform-d)

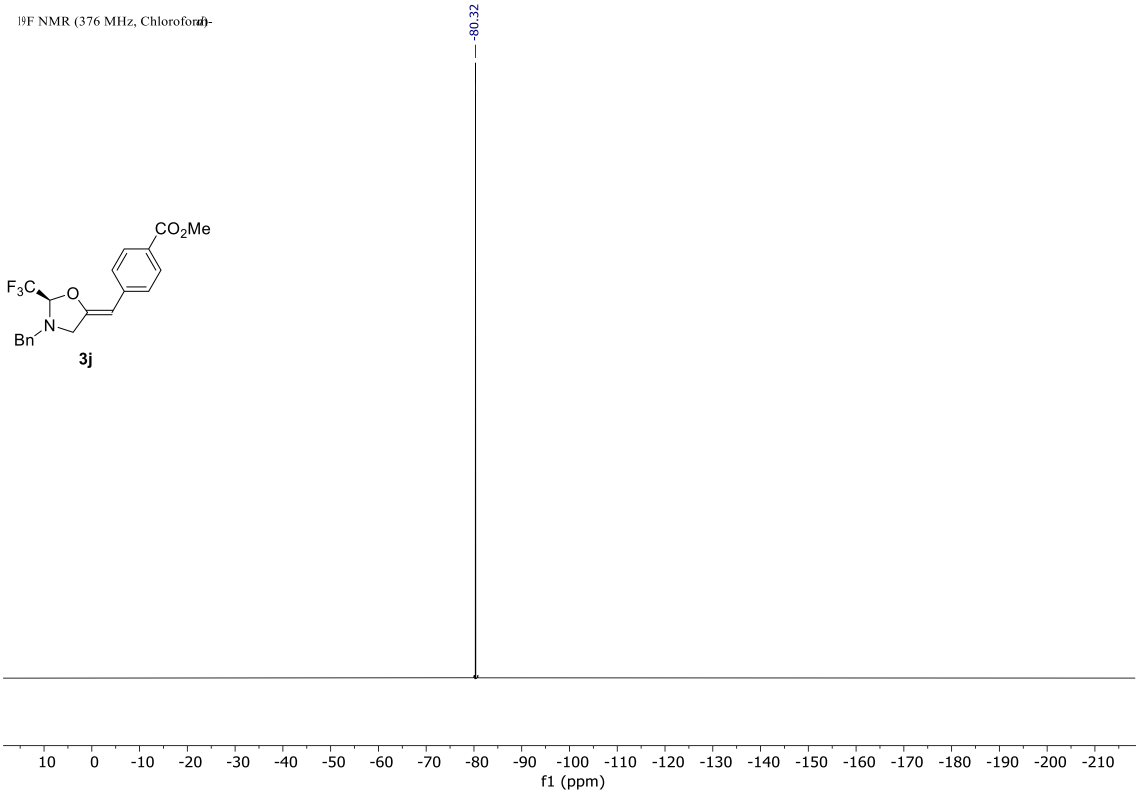

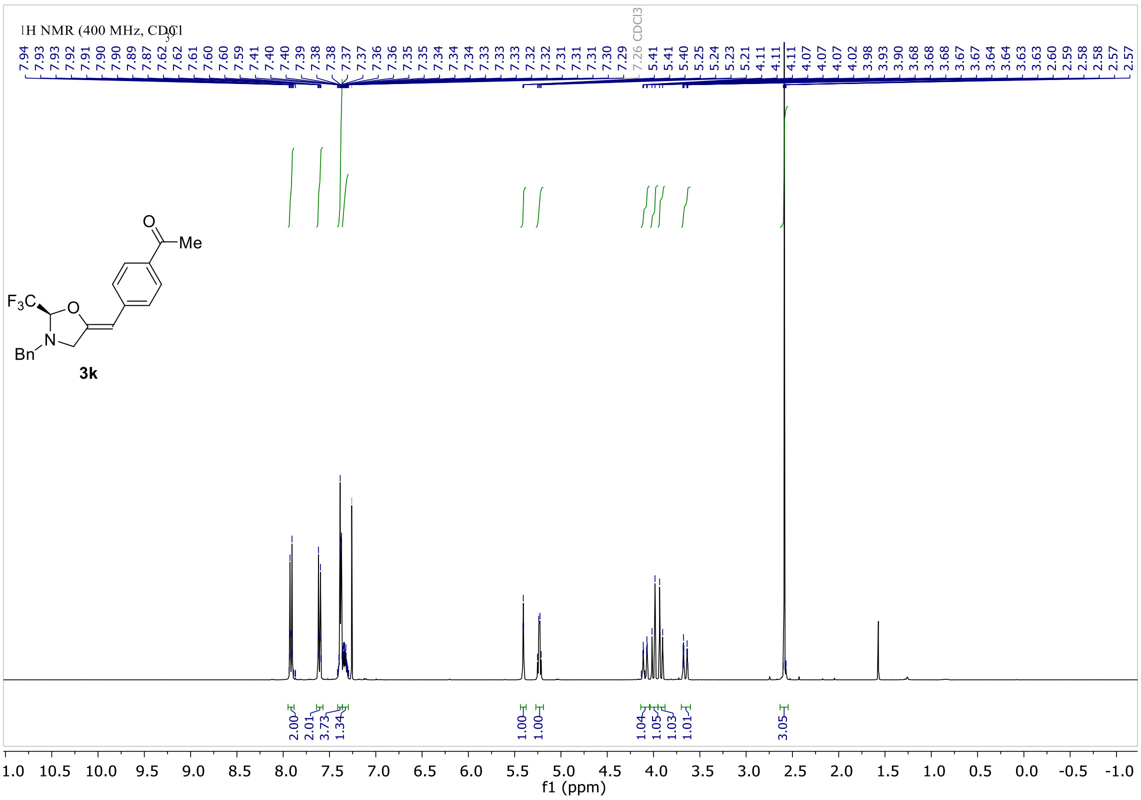
1H NMR (400 MHz, CDCl3)
3k
f1 (ppm)

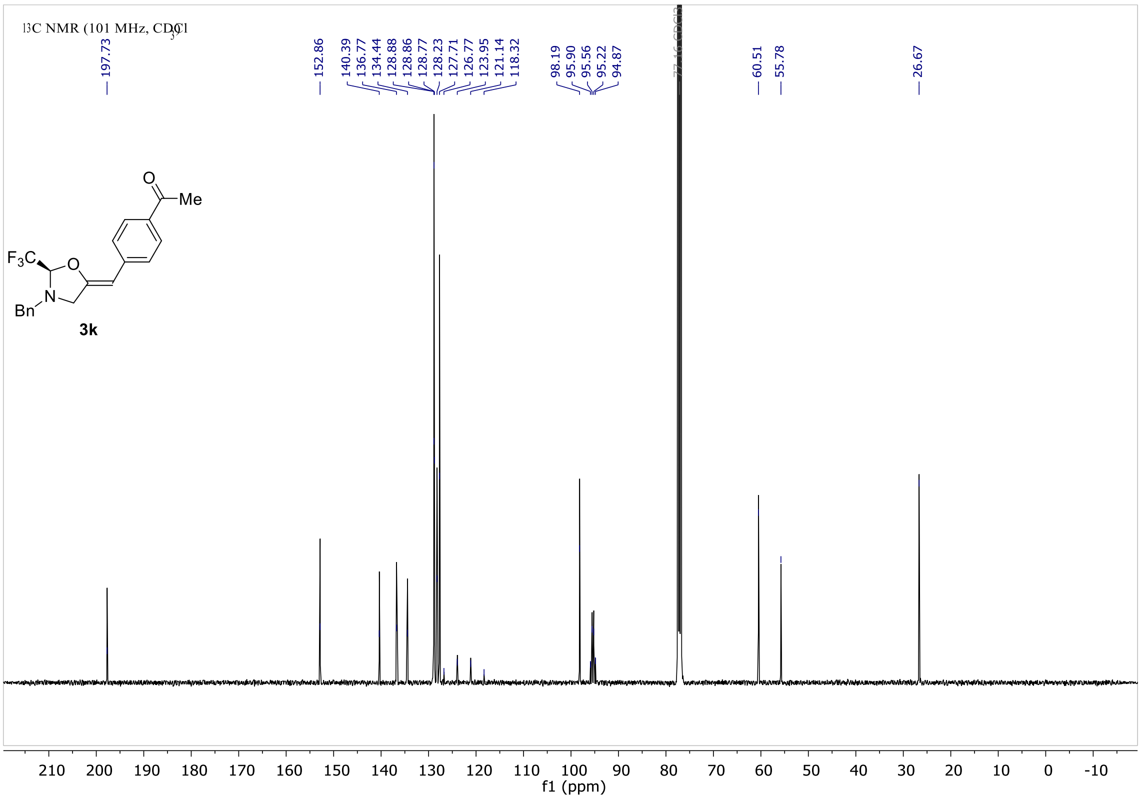
13C NMR (101 MHz, CDCl3)
3k
f1 (ppm)

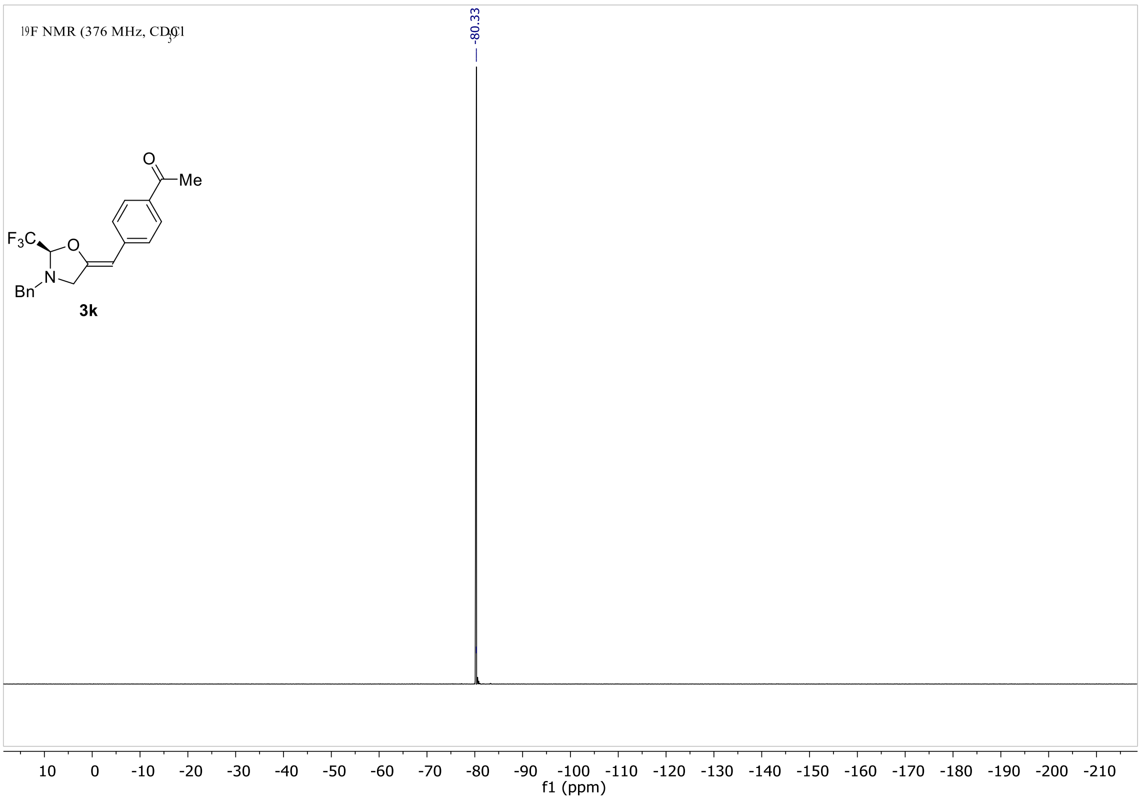
19F NMR (376 MHz, CDCl3)
-80.33
O
Me
F3C
O
N
Bn
3k
10 0 -10 -20 -30 -40 -50 -60 -70 -80 -90 -100 -110 -120 -130 -140 -150 -160 -170 -180 -190 -200 -210
f1 (ppm)

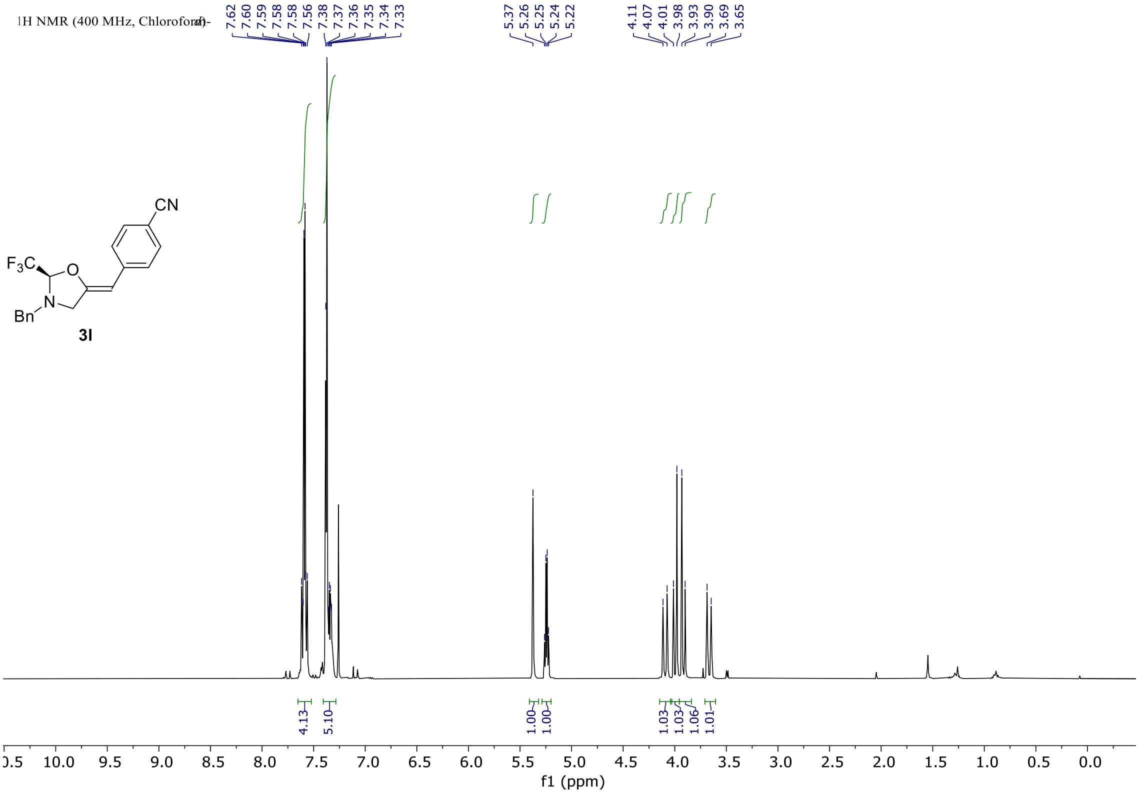
1H NMR (400 MHz, Chloroform-d)
7.62 7.60 7.59 7.58 7.58 7.56 7.38 7.37 7.36 7.35 7.34 7.33
5.37 5.26 5.25 5.24 5.22
4.11 4.07 4.01 3.98 3.93 3.90 3.69 3.65
CN
F3C
O
N
Bn
3l
4.13 5.10 1.00 1.00 1.03 1.03 1.06 1.01
10.0 9.5 9.0 8.5 8.0 7.5 7.0 6.5 6.0 5.5 5.0 4.5 4.0 3.5 3.0 2.5 2.0 1.5 1.0 0.5 0.0
f1 (ppm)

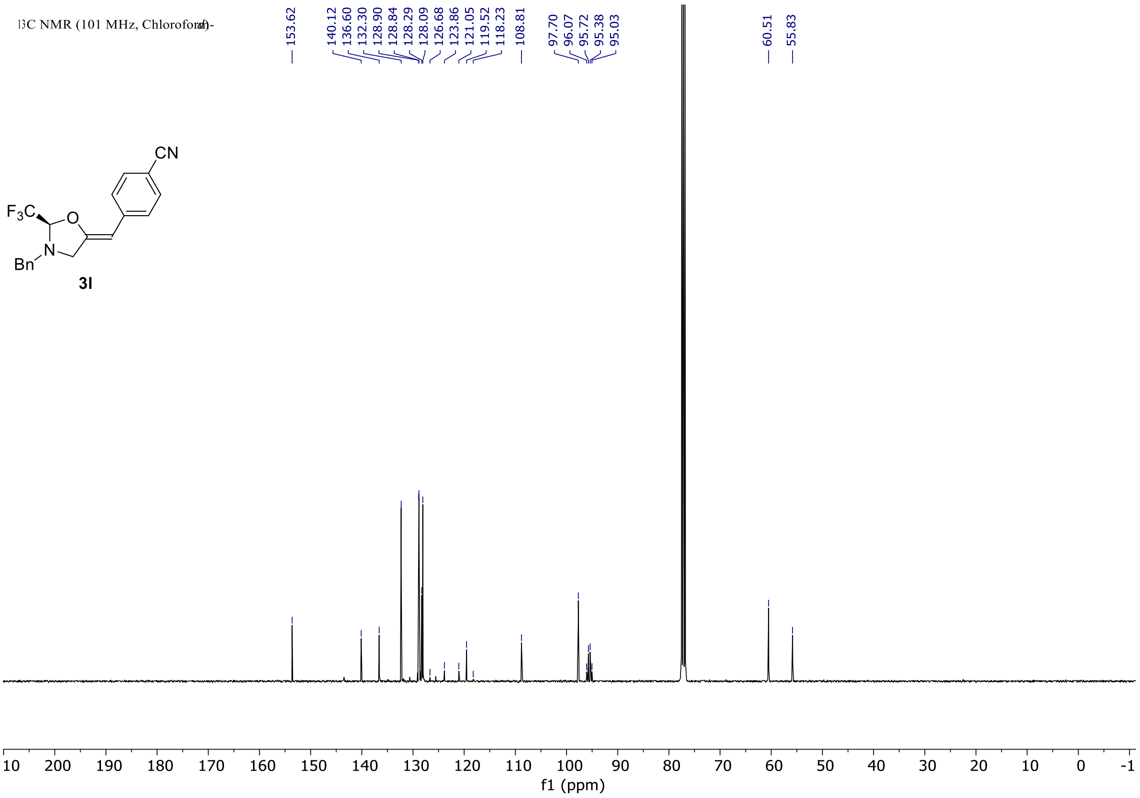
13C NMR (101 MHz, Chloroform-d)
153.62
140.12
136.60
132.30
128.90
128.84
128.29
128.09
126.68
123.86
121.05
119.52
118.23
108.81
97.70
96.07
95.72
95.38
95.03
60.51
55.83
CN
F3C
O
N
Bn
3l
f1 (ppm)

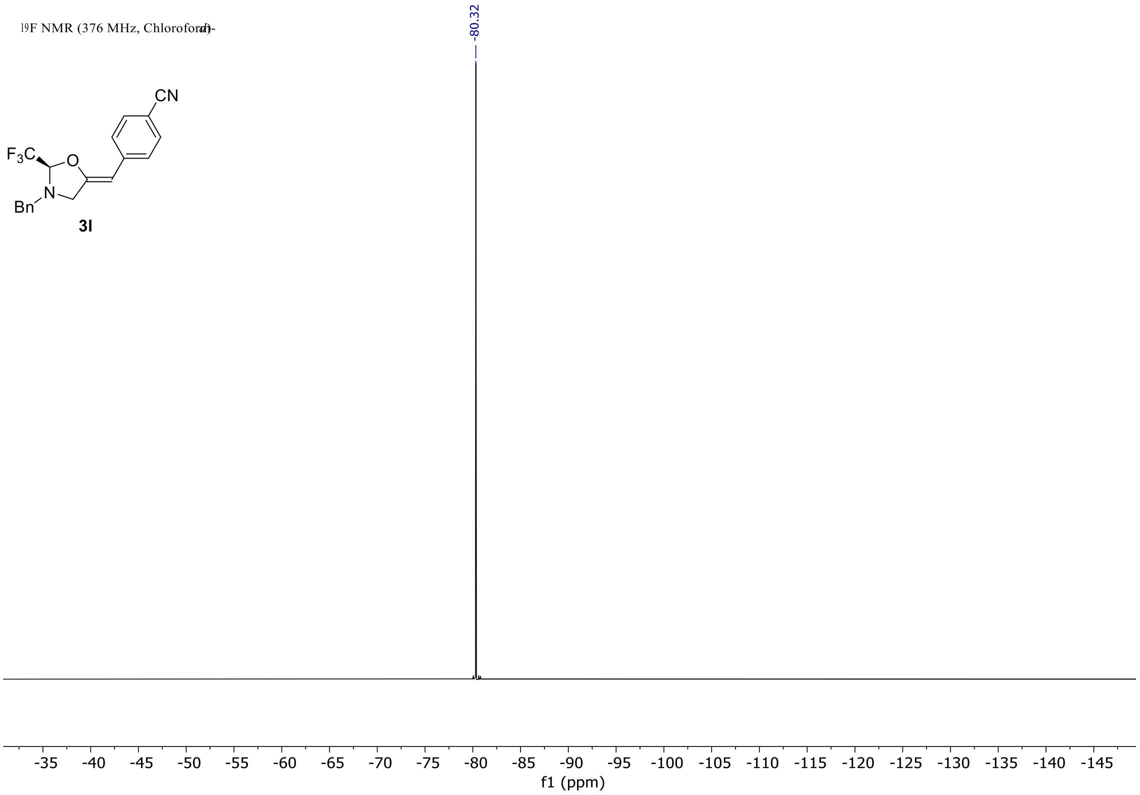
19F NMR (376 MHz, Chloroform-d)
-80.32
CN
F3C
O
N
Bn
3l
f1 (ppm)

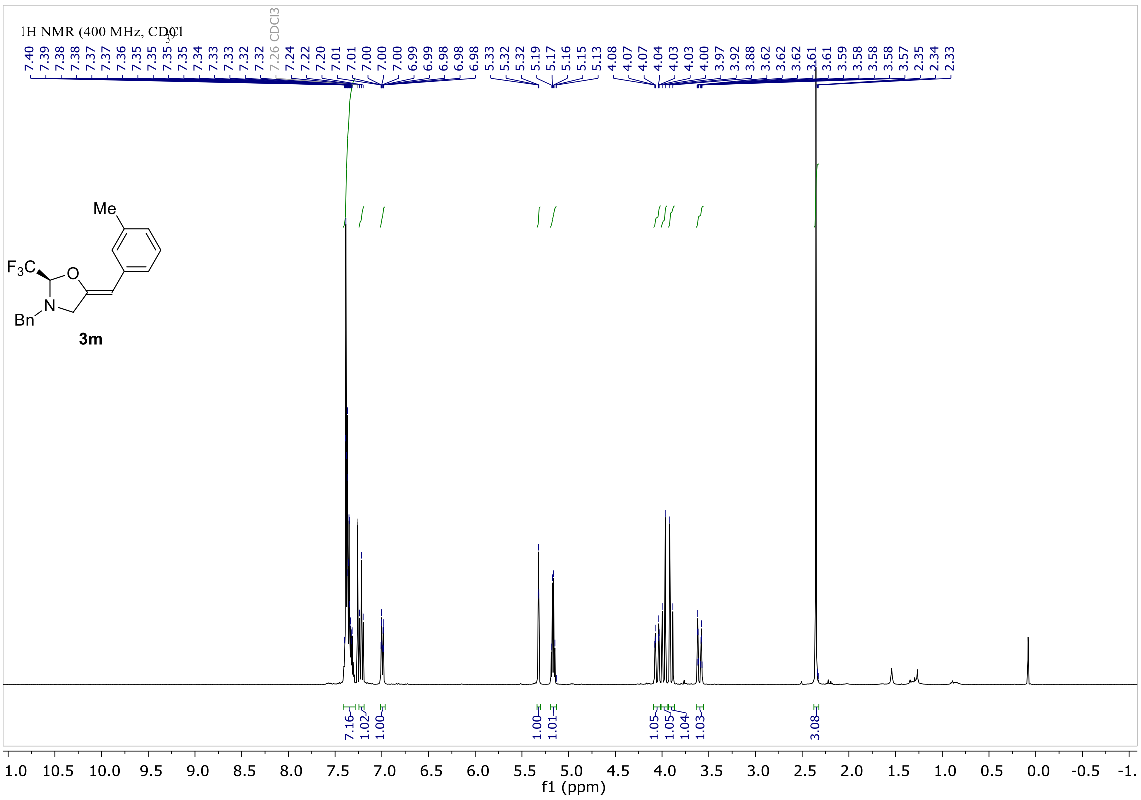

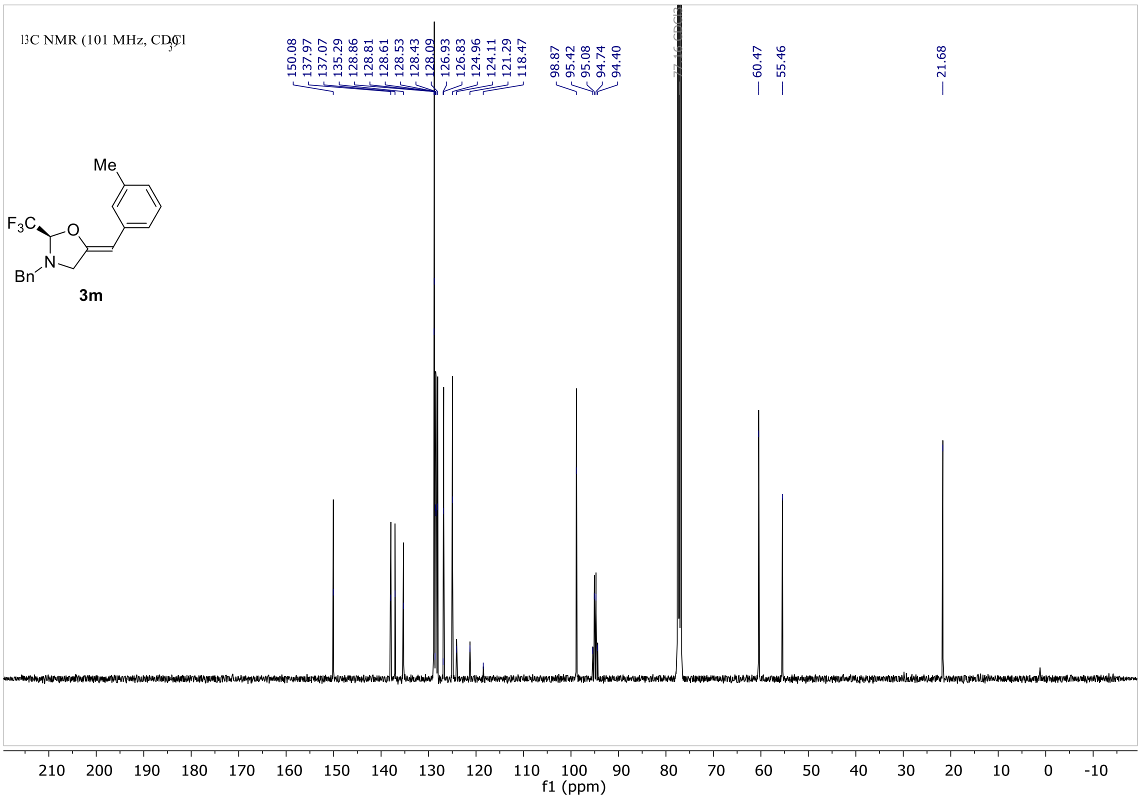

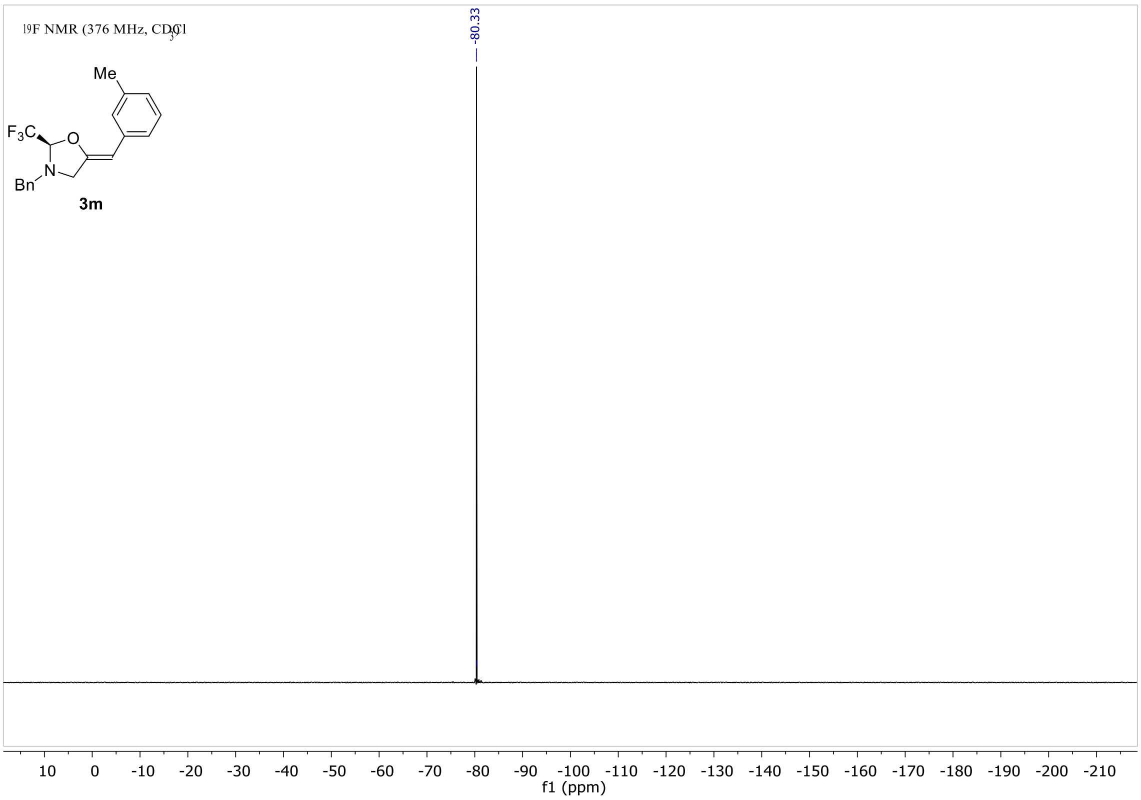
19F NMR (376 MHz, CDCl3
-80.33
Me
F3C
O
N
Bn
3m
10 0 -10 -20 -30 -40 -50 -60 -70 -80 -90 -100 -110 -120 -130 -140 -150 -160 -170 -180 -190 -200 -210
f1 (ppm)

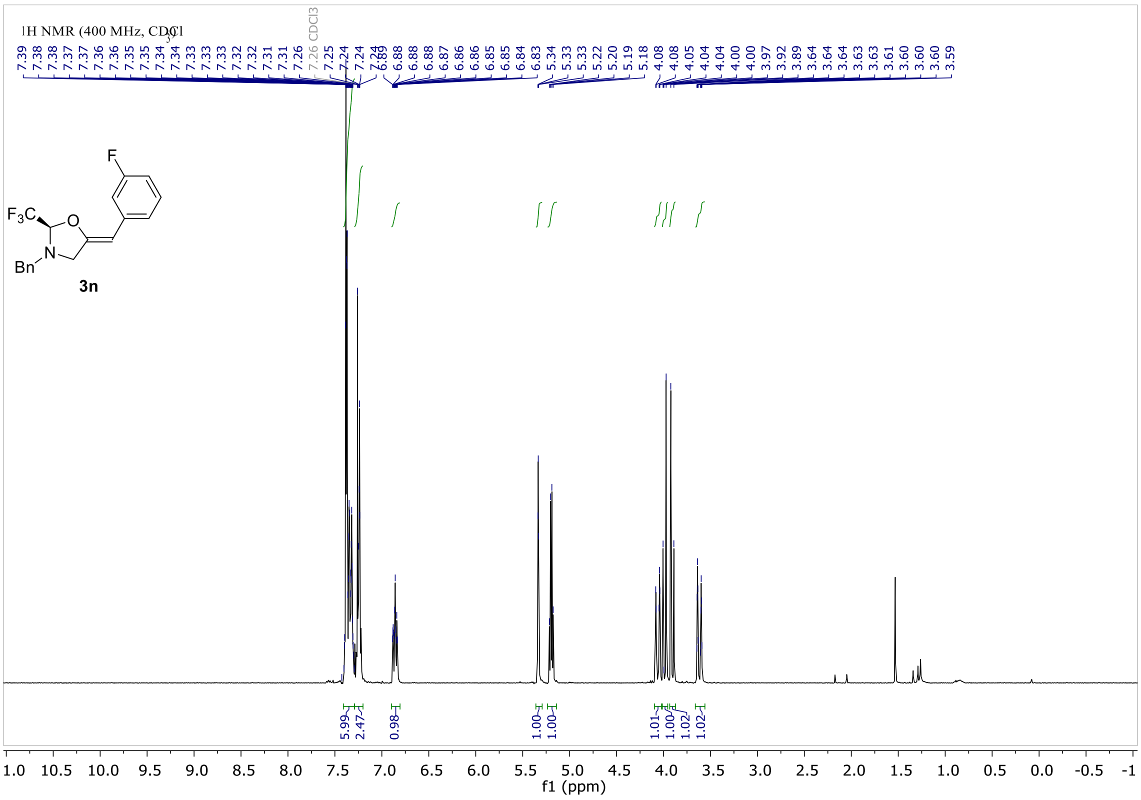
1H NMR (400 MHz, CDCl3
7.26 CDCl3
F
F3C
O
N
Bn
3n
5.99
2.47
0.98
1.00
1.00
1.01
1.00
1.02
1.02
1.0 10.5 10.0 9.5 9.0 8.5 8.0 7.5 7.0 6.5 6.0 5.5 5.0 4.5 4.0 3.5 3.0 2.5 2.0 1.5 1.0 0.5 0.0 -0.5 -1
f1 (ppm)

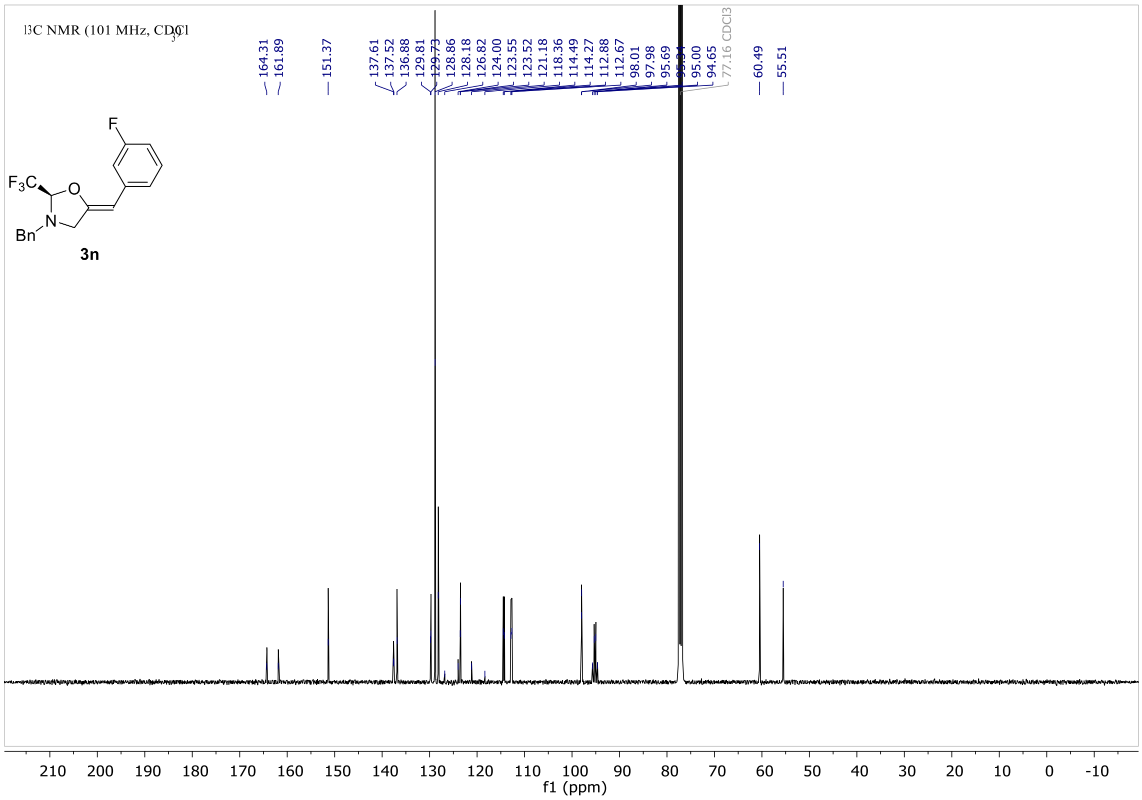
13C NMR (101 MHz, CDCl3)
3n
f1 (ppm)

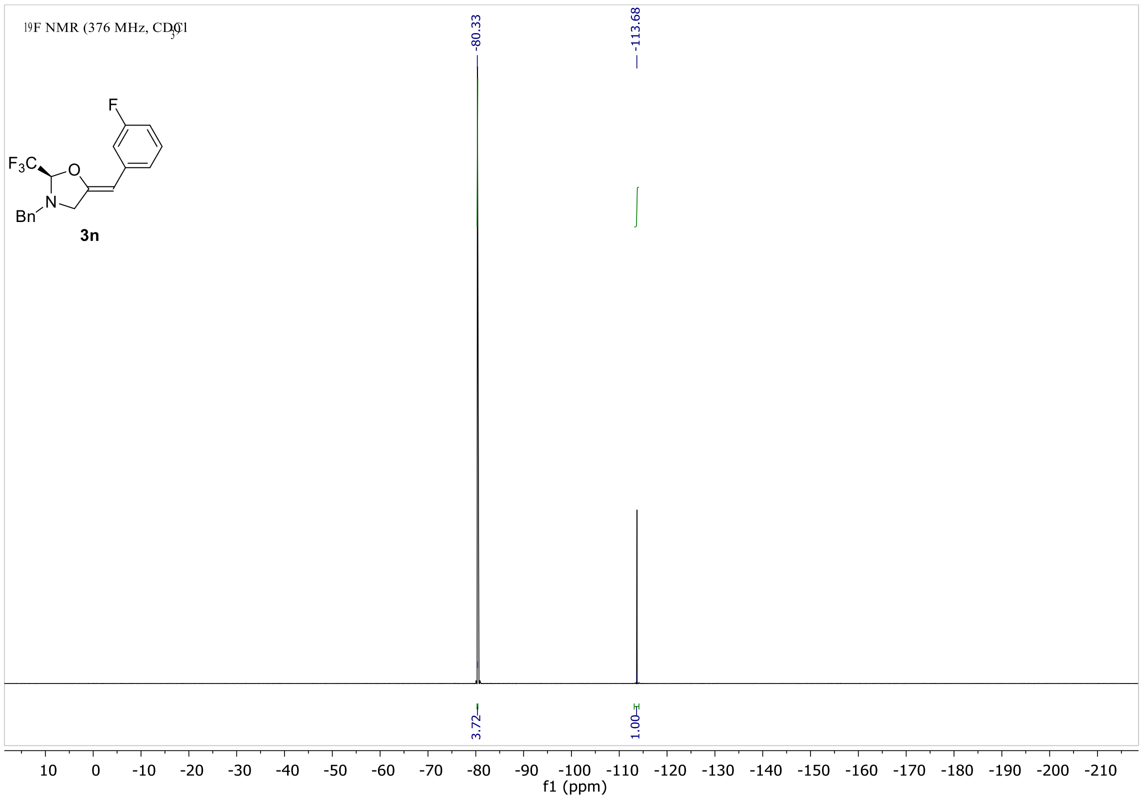
19F NMR (376 MHz, CDCl3)
-80.33
-113.68
3n
3.72
1.00
f1 (ppm)

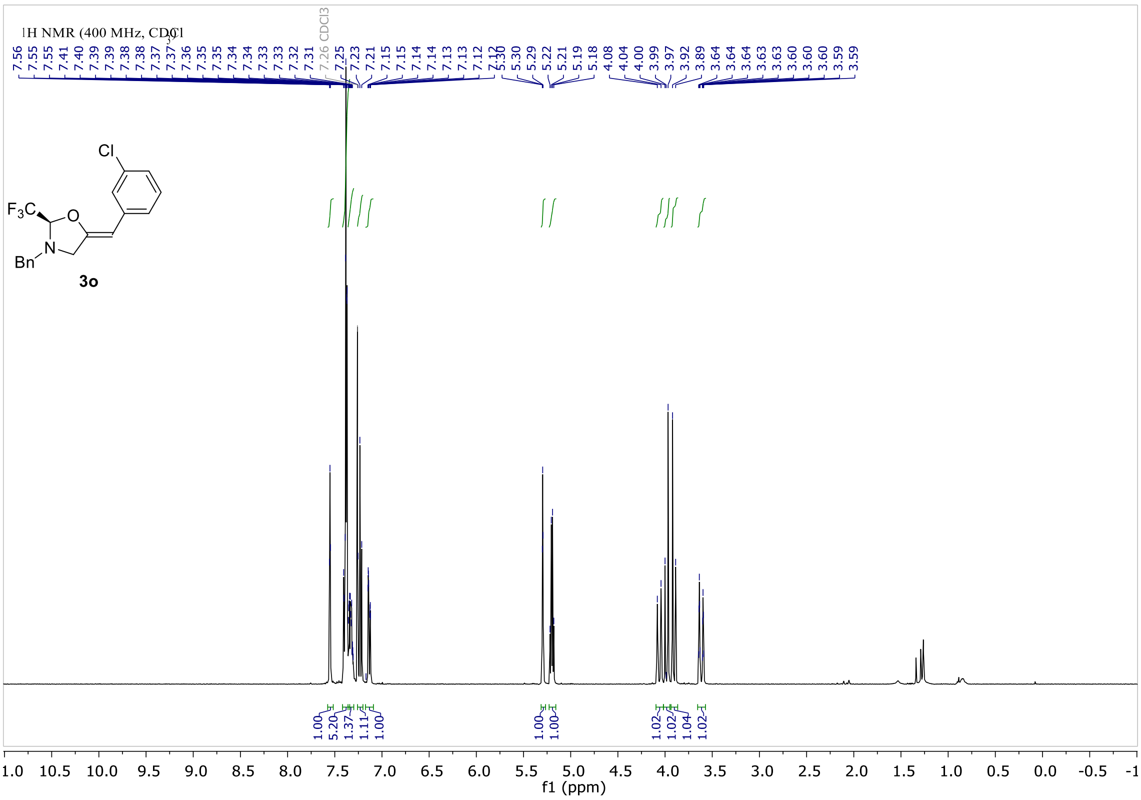

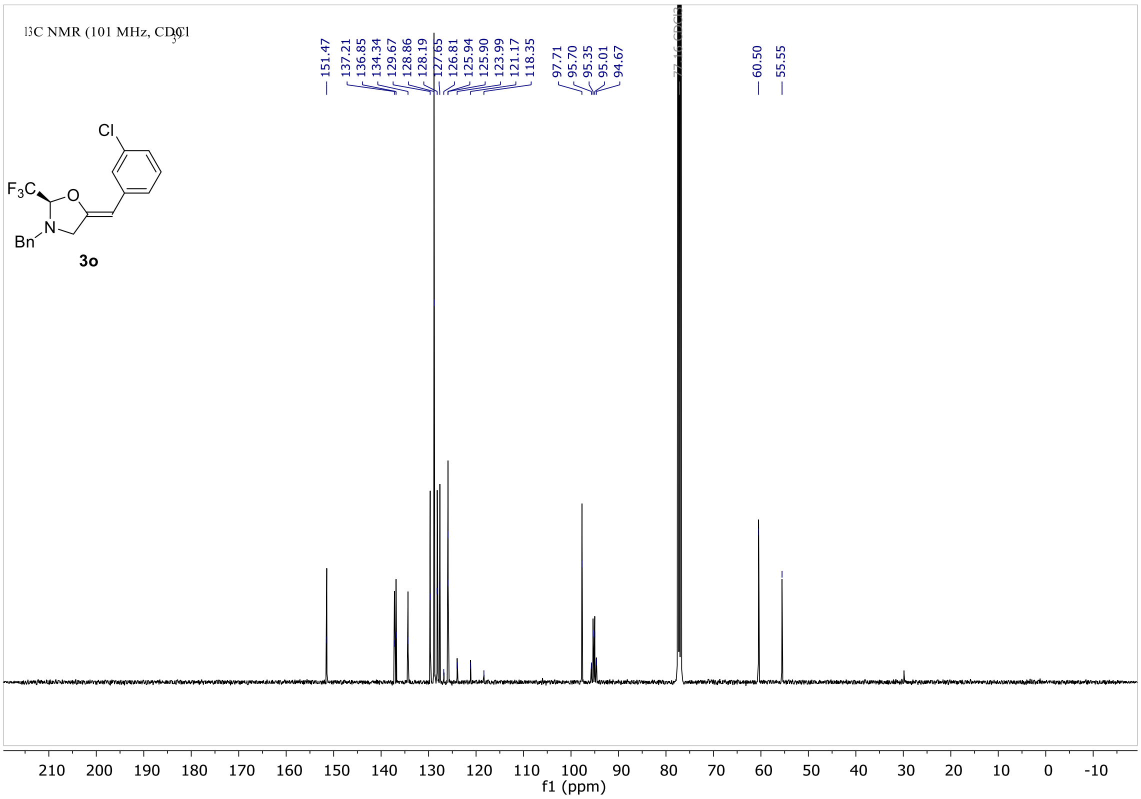

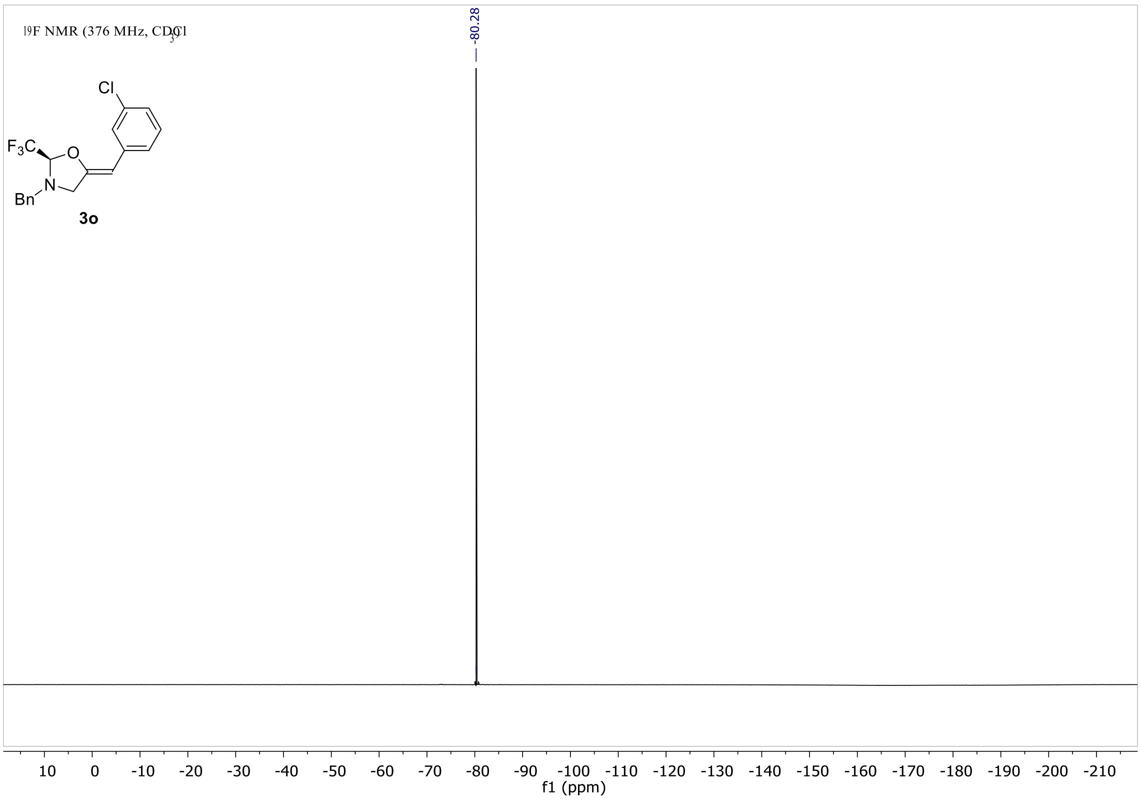
19F NMR (376 MHz, CDCl3)
-80.28
Cl
F3C
O
N
Bn
3o
f1 (ppm)

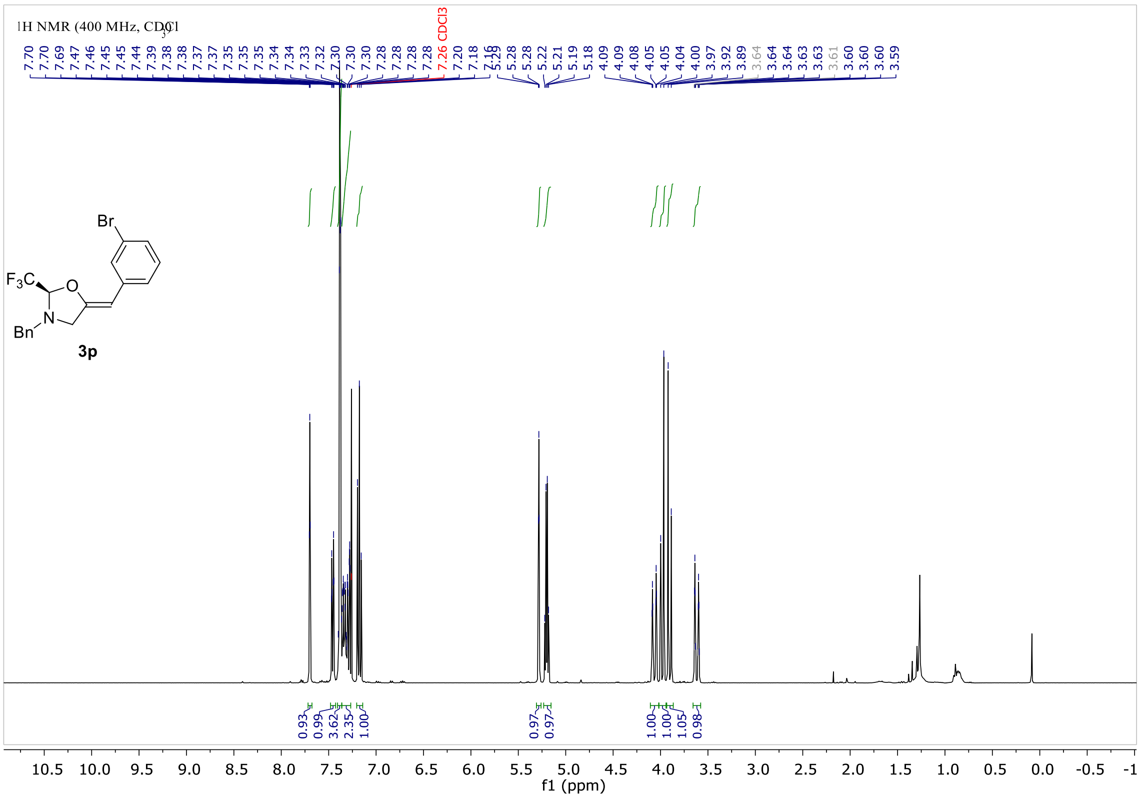
1H NMR (400 MHz, CDCl3)
7.26 CDCl3
Br
F3C
O
N
Bn
3p
0.93
0.99
3.62
2.35
1.00
0.97
0.97
1.00
1.00
1.05
0.98
f1 (ppm)

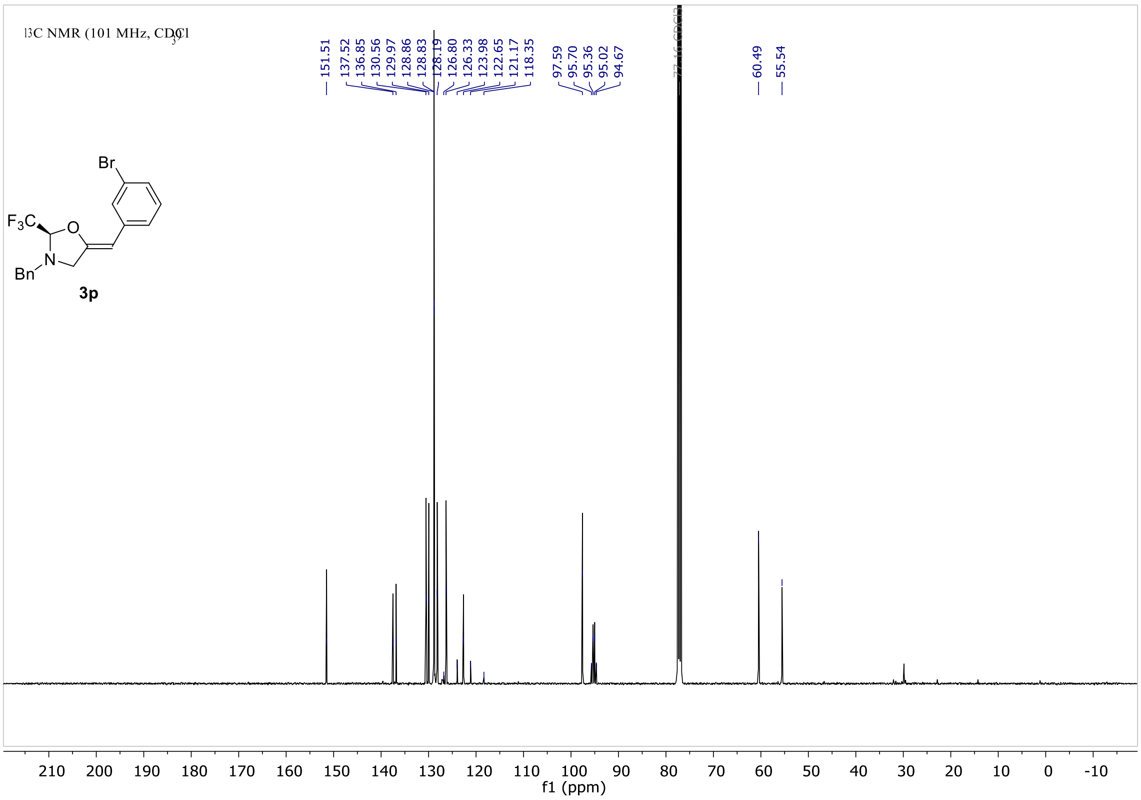
13C NMR (101 MHz, CDCl3)
151.51
137.52
136.85
130.56
129.97
128.86
128.83
128.19
126.80
126.33
123.98
122.65
121.17
118.35
97.59
95.70
95.36
95.02
94.67
60.49
55.54
Br
F3C
O
N
Bn
3p
210 200 190 180 170 160 150 140 130 120 110 100 90 80 70 60 50 40 30 20 10 0 -10
f1 (ppm)

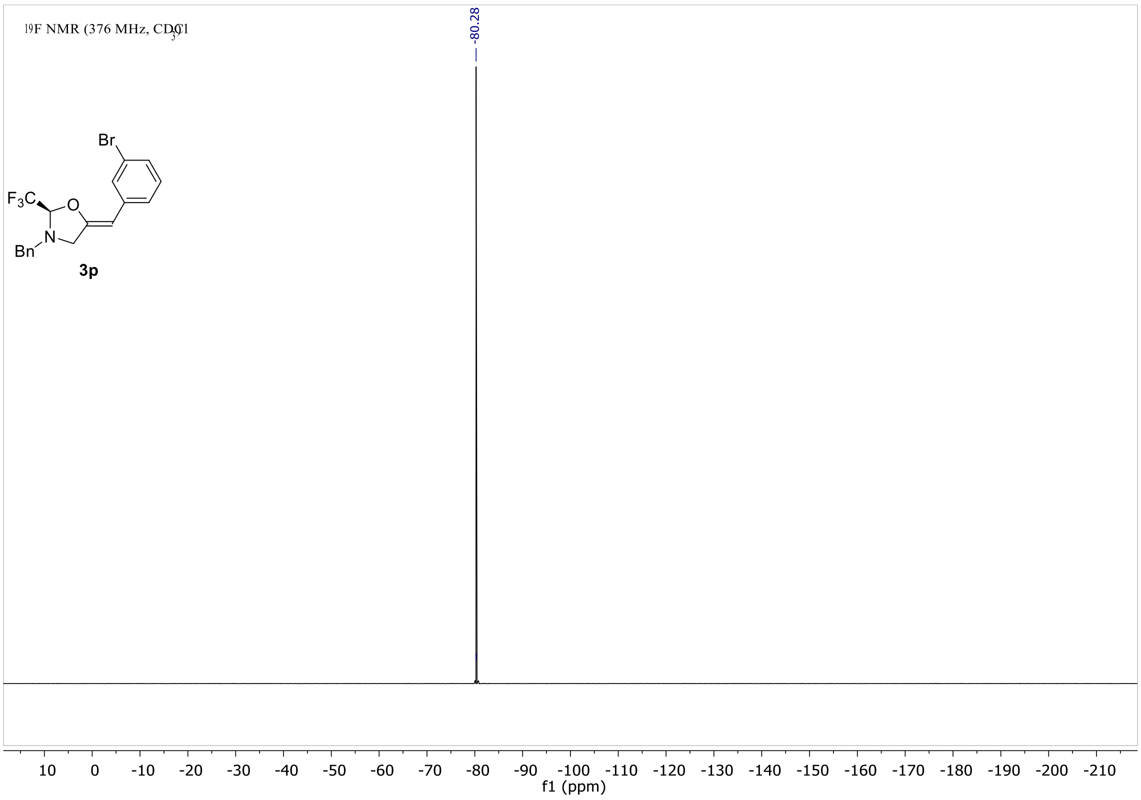
19F NMR (376 MHz, CDCl3)
-80.28
Br
F3C
O
N
Bn
3p
10 0 -10 -20 -30 -40 -50 -60 -70 -80 -90 -100 -110 -120 -130 -140 -150 -160 -170 -180 -190 -200 -210
f1 (ppm)

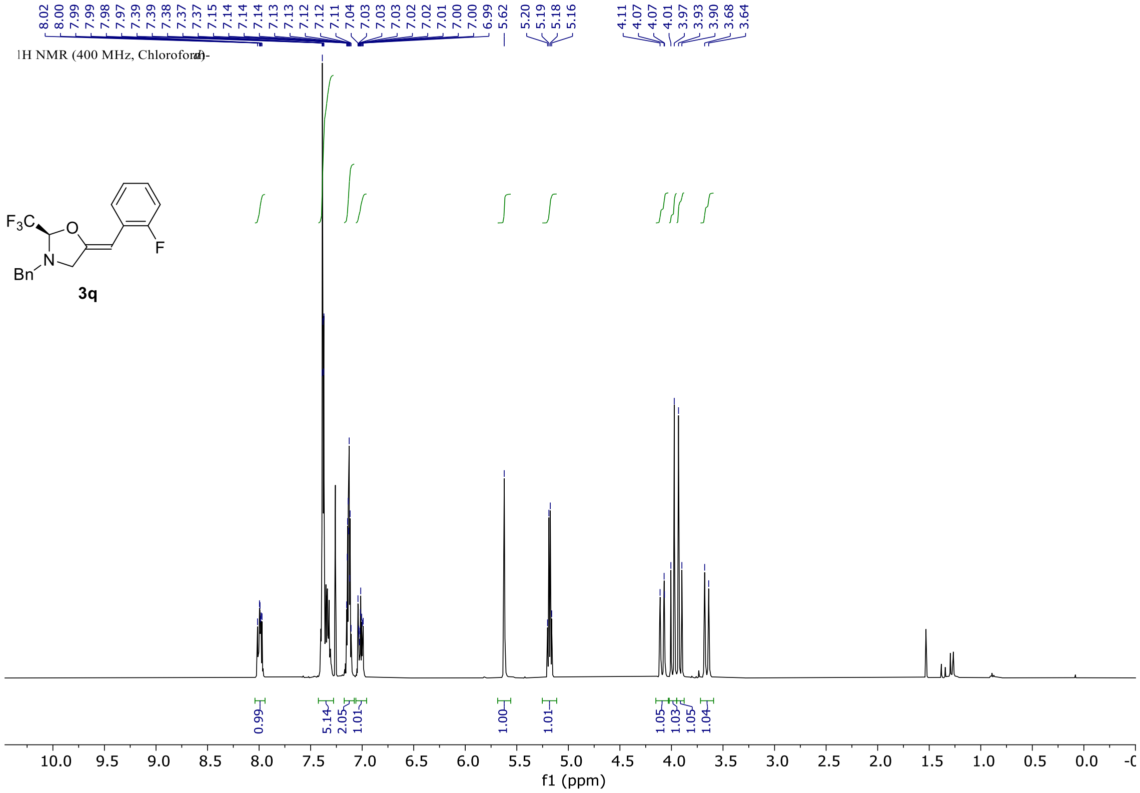
8.02
8.00
7.99
7.99
7.98
7.97
7.39
7.39
7.38
7.37
7.37
7.15
7.14
7.14
7.14
7.13
7.13
7.12
7.12
7.11
7.04
7.03
7.03
7.03
7.02
7.02
7.01
7.00
6.99
5.62
5.20
5.19
5.18
5.16
4.11
4.07
4.01
3.97
3.93
3.90
3.68
3.64
1H NMR (400 MHz, Chloroform-d)
F3C
O
N
Bn
F
3q
0.99
5.14
2.05
1.01
1.00
1.01
1.05
1.03
1.05
1.04
10.0
9.5
9.0
8.5
8.0
7.5
7.0
6.5
6.0
5.5
5.0
4.5
4.0
3.5
3.0
2.5
2.0
1.5
1.0
0.5
0.0
f1 (ppm)

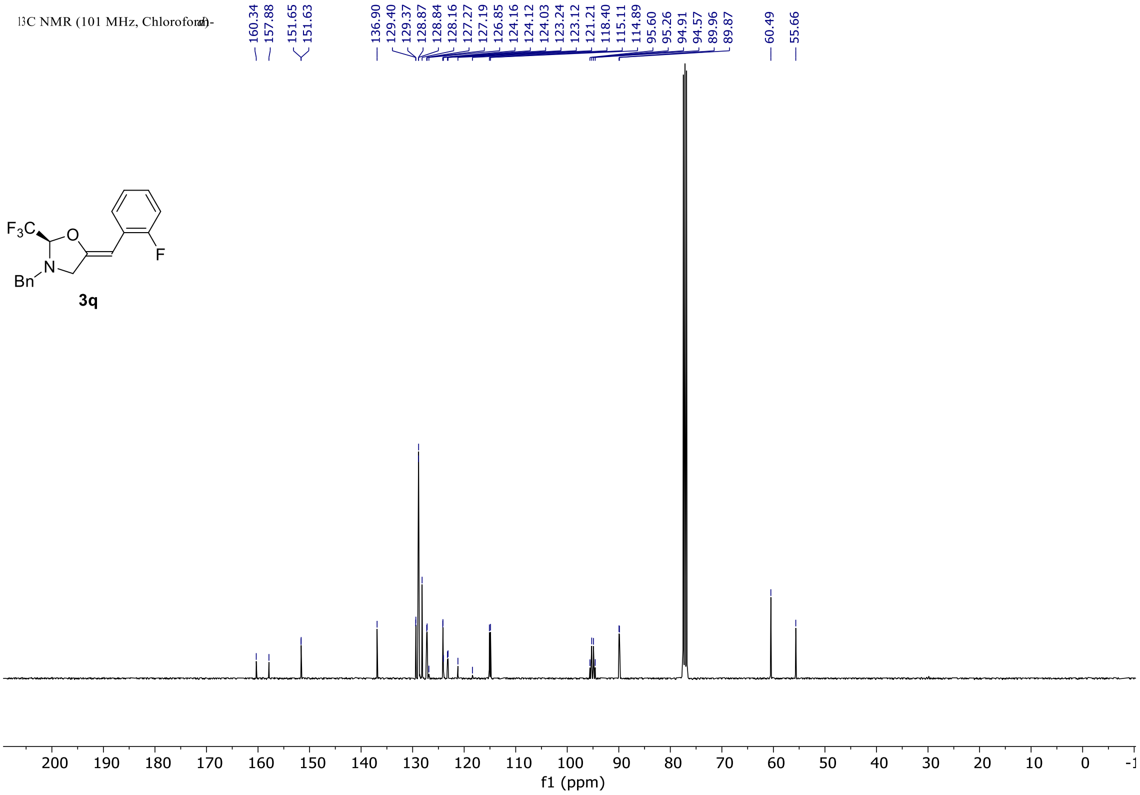
13C NMR (101 MHz, Chloroform-d)
160.34
157.88
151.65
151.63
136.90
129.40
129.37
128.87
128.84
128.16
127.27
127.19
126.85
124.16
124.12
124.03
123.24
123.12
121.21
118.40
115.11
114.89
95.60
95.26
94.91
94.57
89.96
89.87
60.49
55.66
F3C
O
N
Bn
F
3q
200
190
180
170
160
150
140
130
120
110
100
90
80
70
60
50
40
30
20
10
0
f1 (ppm)

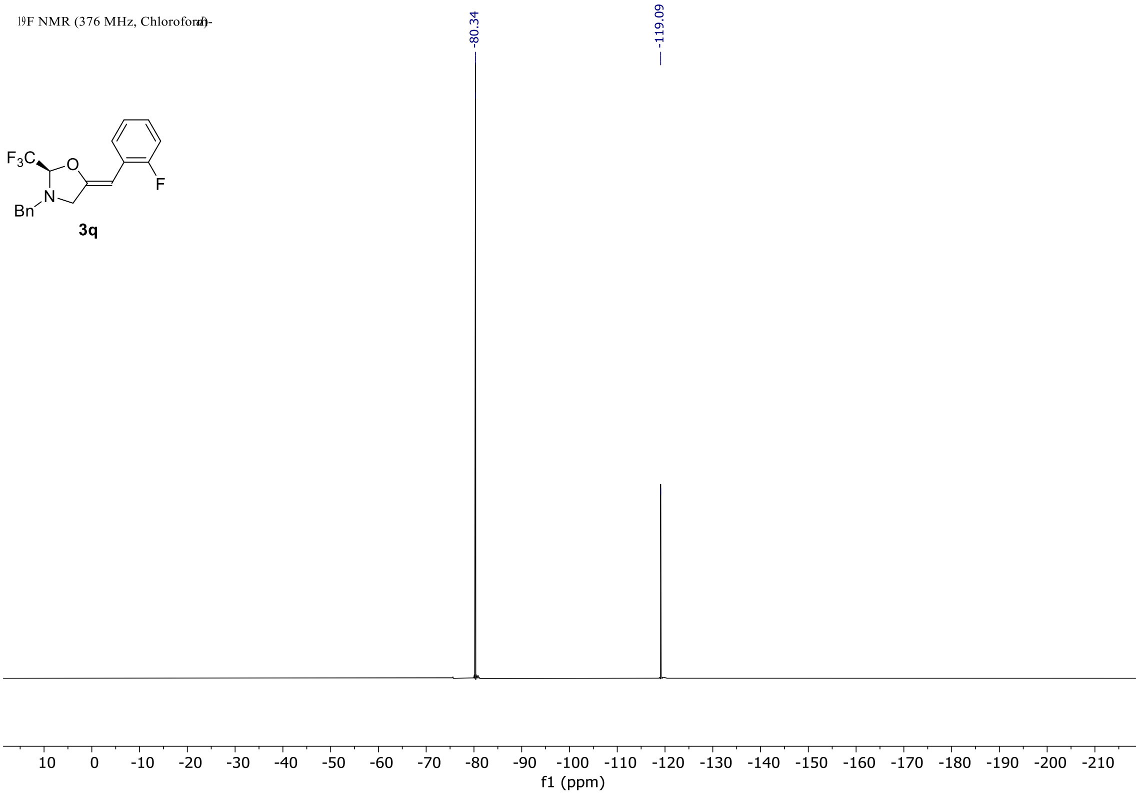
19F NMR (376 MHz, Chloroform-d)
-80.34
-119.09
F3C
O
N
Bn
F
3q
f1 (ppm)

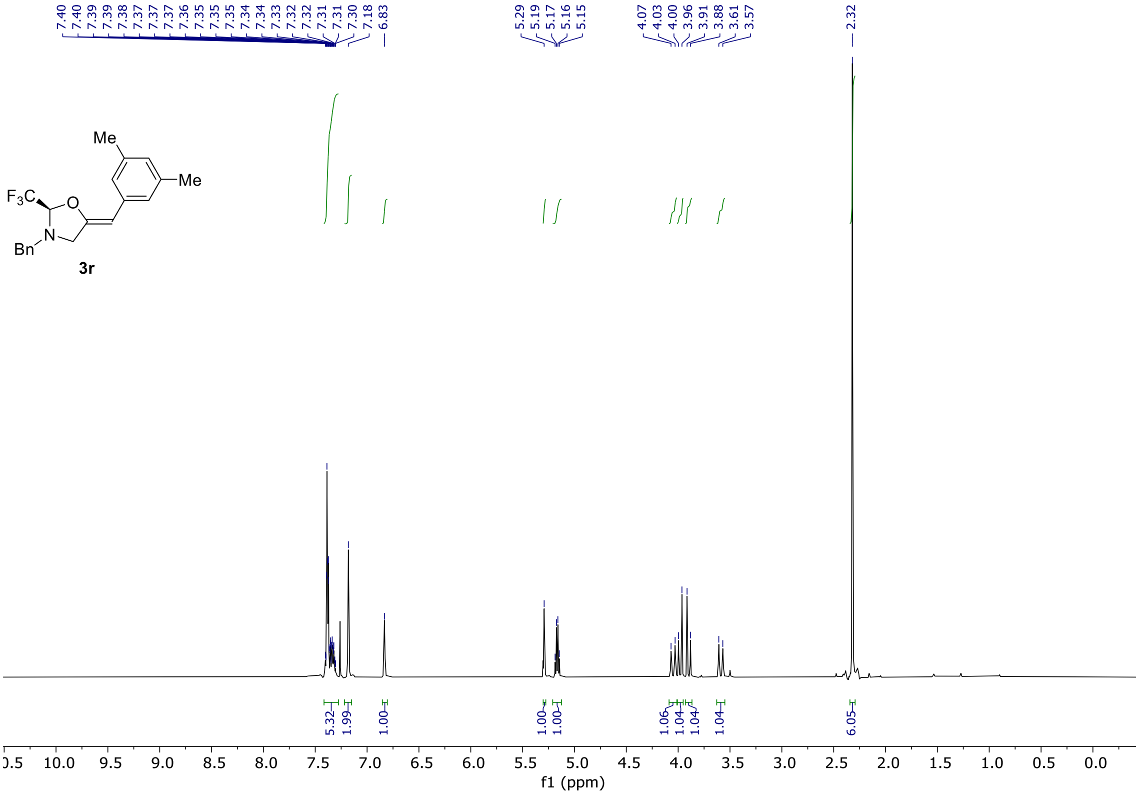
Me
Me
F3C
O
N
Bn
3r
f1 (ppm)

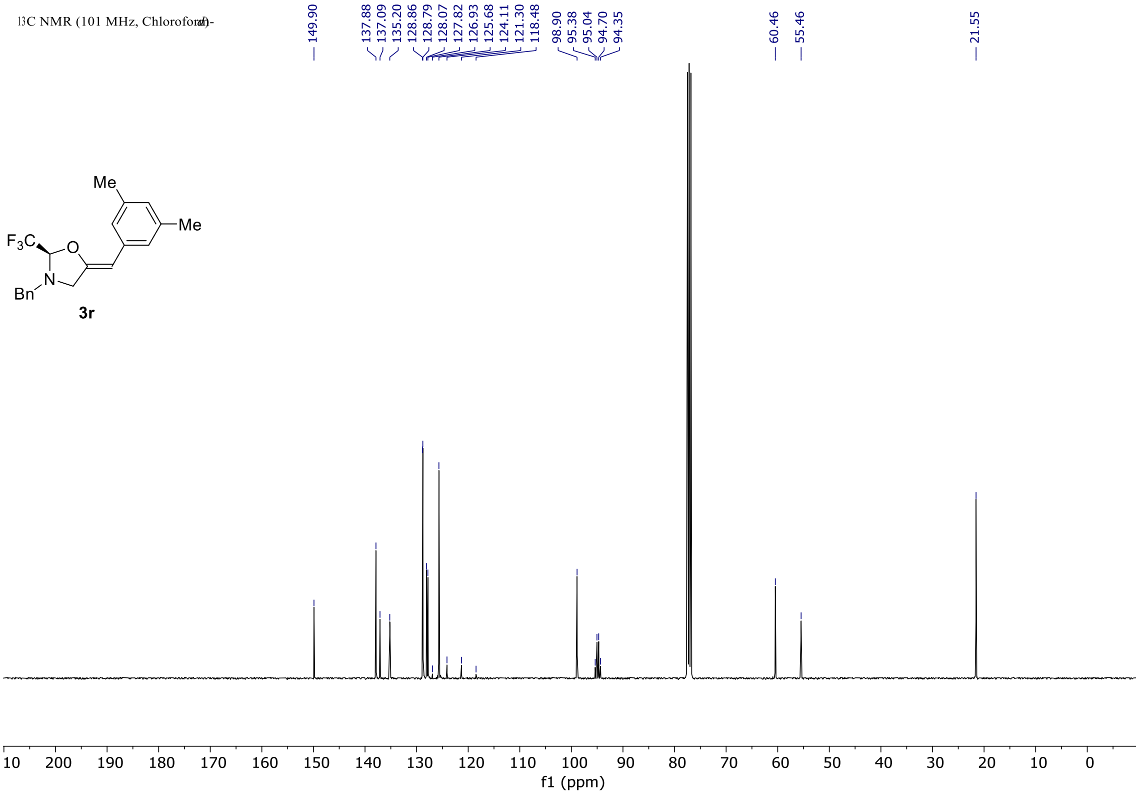
13C NMR (101 MHz, Chloroform-d)
149.90
137.88
137.09
135.20
128.86
128.79
128.07
127.82
126.93
125.68
124.11
121.30
118.48
98.90
95.38
95.04
94.70
94.35
60.46
55.46
21.55
Me
Me
F3C
O
N
Bn
3r
210 200 190 180 170 160 150 140 130 120 110 100 90 80 70 60 50 40 30 20 10 0
f1 (ppm)

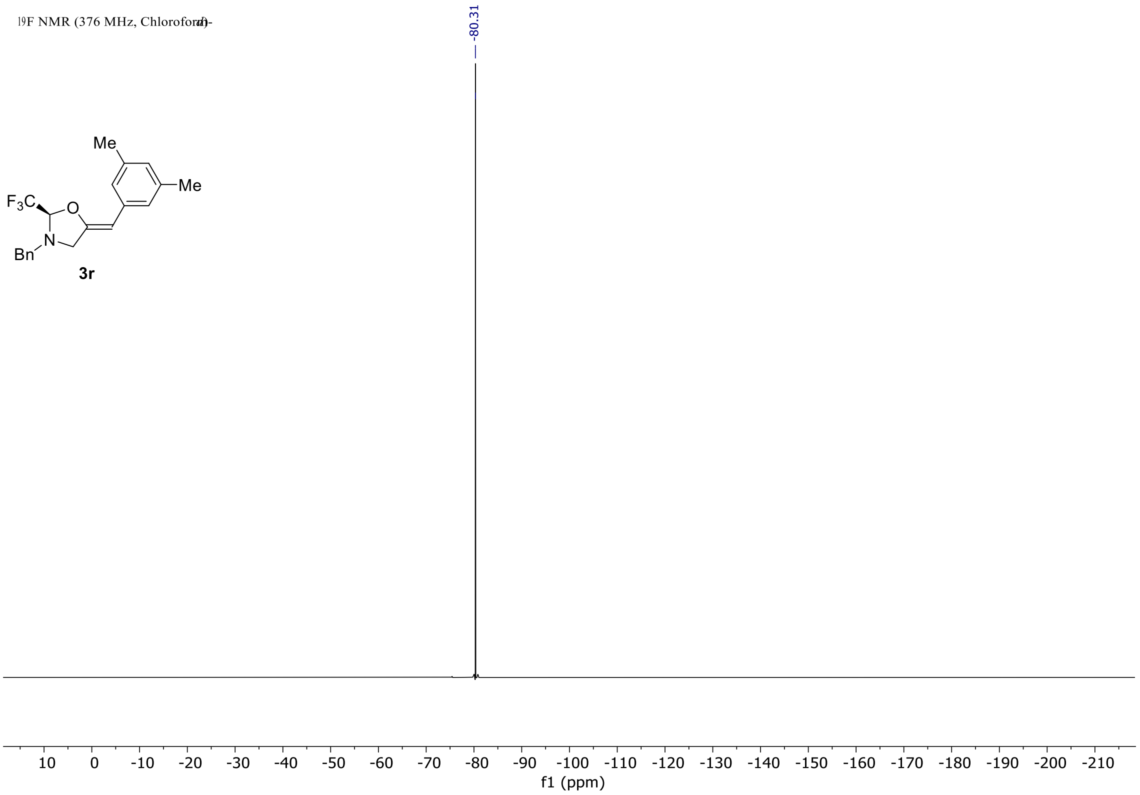
19F NMR (376 MHz, Chloroform-d)
-80.31
Me
Me
F3C
O
N
Bn
3r
10 0 -10 -20 -30 -40 -50 -60 -70 -80 -90 -100 -110 -120 -130 -140 -150 -160 -170 -180 -190 -200 -210
f1 (ppm)

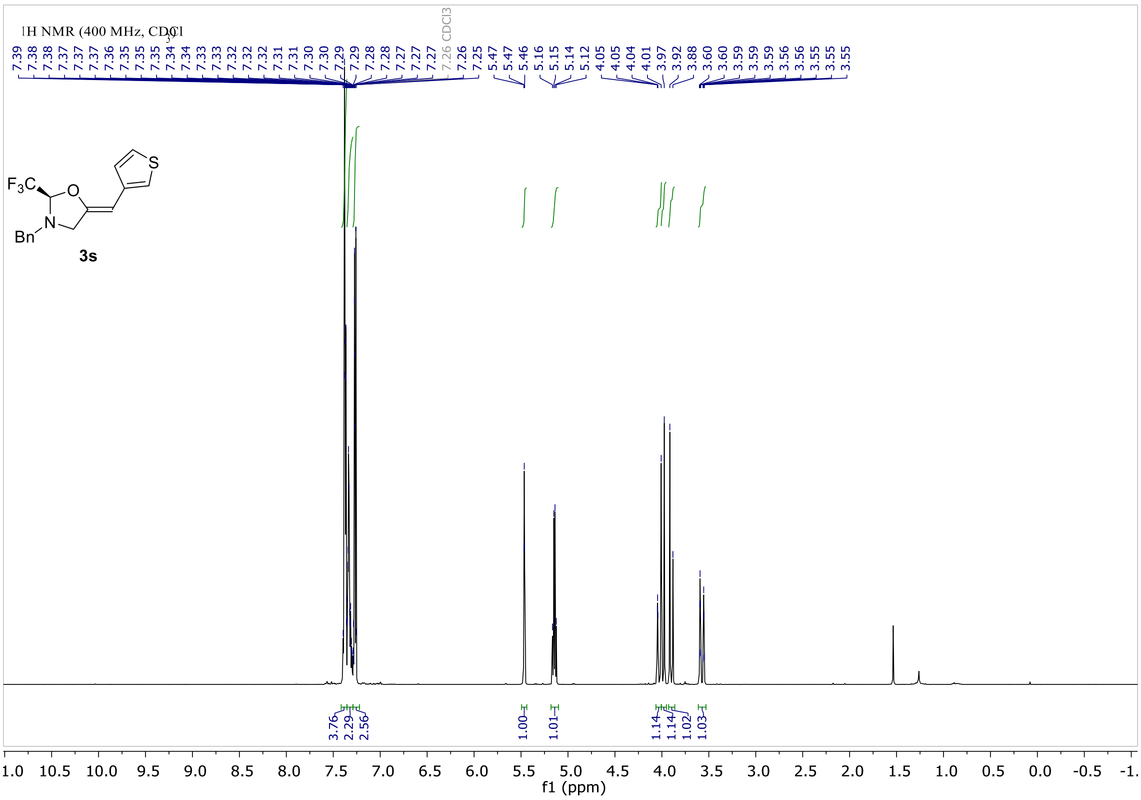
1H NMR (400 MHz, CDCl3)
7.39 7.38 7.37 7.37 7.37 7.36 7.35 7.35 7.34 7.34 7.33 7.33 7.32 7.32 7.32 7.31 7.30 7.30 7.29 7.29 7.28 7.28 7.27 7.27 7.27 7.26 CDCl3 7.26 7.25 5.47 5.47 5.46 5.16 5.15 5.14 5.12 4.05 4.05 4.04 4.01 3.97 3.92 3.88 3.60 3.60 3.59 3.59 3.59 3.56 3.56 3.55 3.55 3.55
F3C O S N Bn
3s
3.76 2.29 2.56 1.00 1.01 1.14 1.14 1.02 1.03
f1 (ppm)

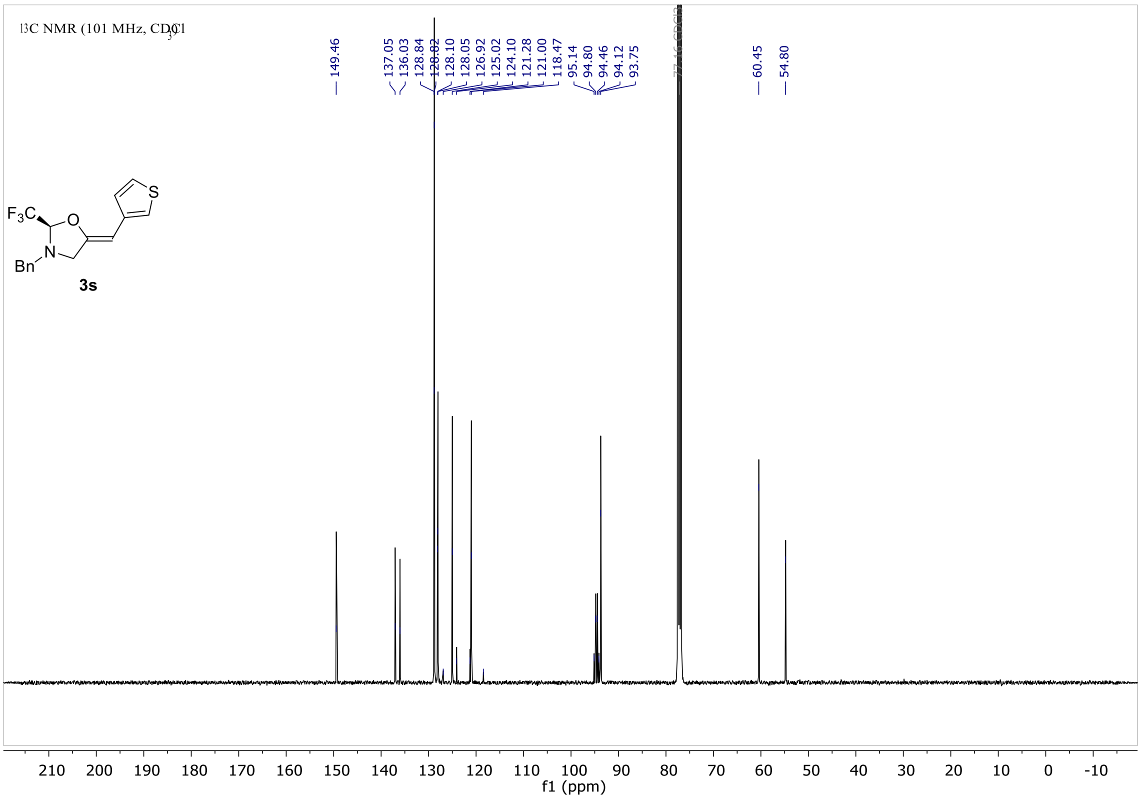
13C NMR (101 MHz, CDCl3)
149.46 137.05 136.03 128.84 128.82 128.10 128.05 126.92 125.02 124.10 121.28 121.00 118.47 95.14 94.80 94.46 94.12 93.75 77.16 CDCl3 60.45 54.80
F3C O S N Bn
3s
f1 (ppm)

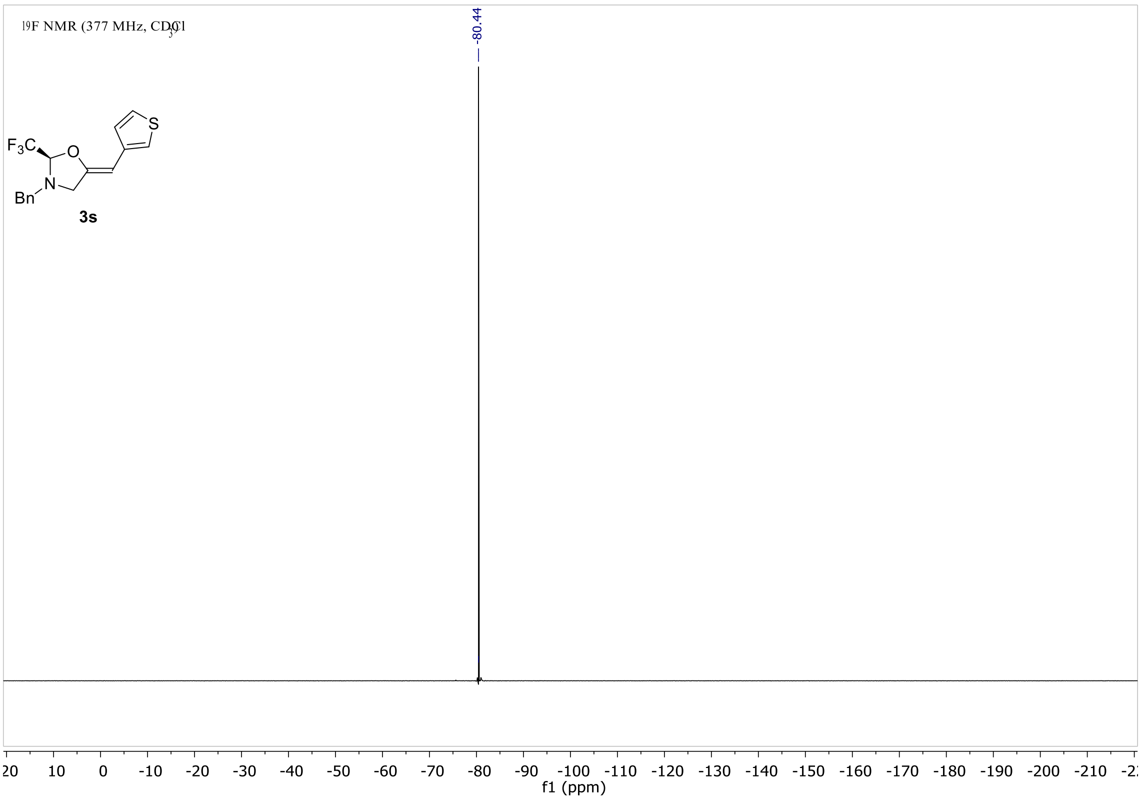
19F NMR (377 MHz, CDCl3)
-80.44
F3C
O
S
N
Bn
3s
f1 (ppm)

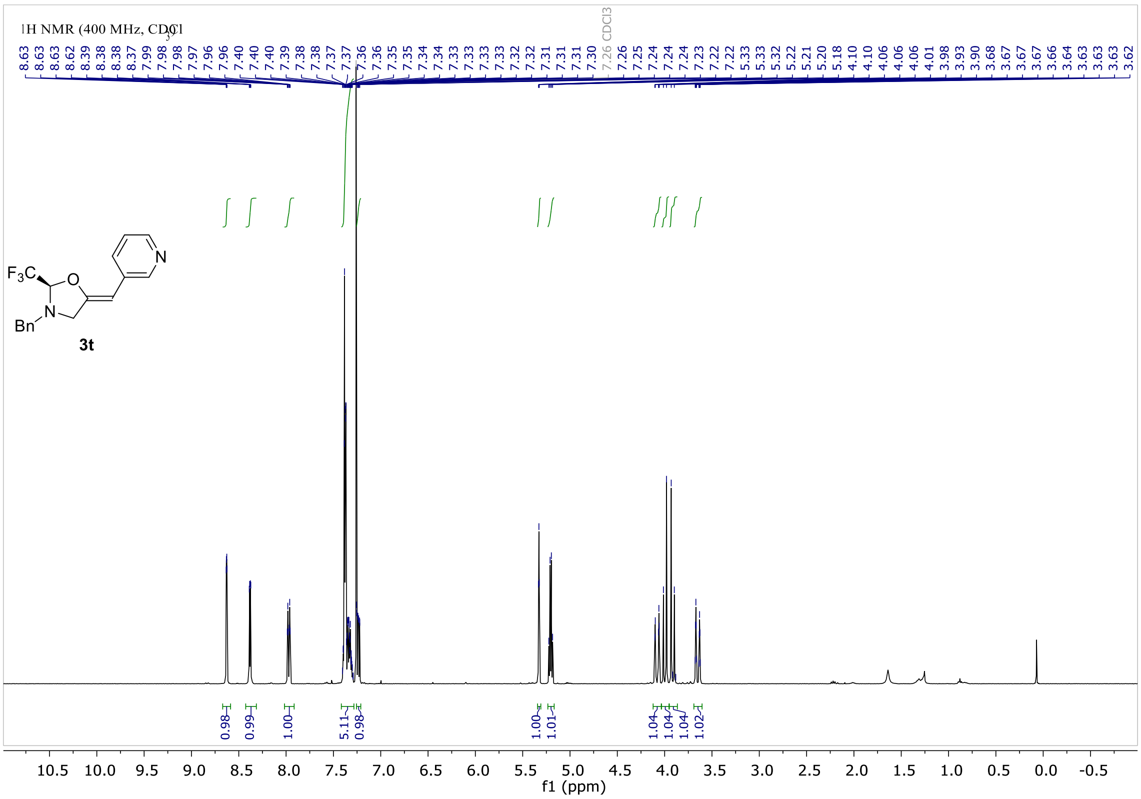
1H NMR (400 MHz, CDCl3)
7.26 CDCl3
F3C
O
N
N
Bn
3t
0.98
0.99
1.00
5.11
0.98
1.00
1.01
1.04
1.04
1.04
1.02
f1 (ppm)

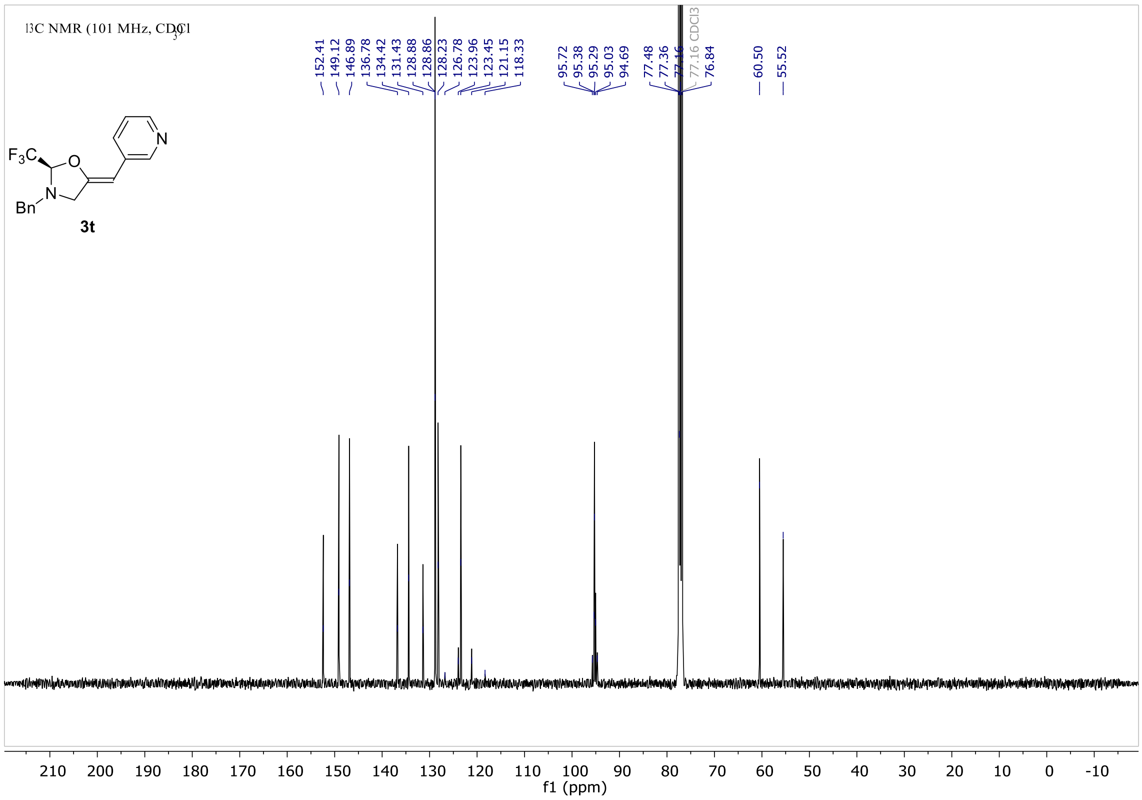
13C NMR (101 MHz, CDCl3)
152.41
149.12
146.89
136.78
134.42
131.43
128.86
128.88
128.23
126.78
123.96
123.45
121.15
118.33
95.72
95.38
95.29
95.03
94.69
77.48
77.36
77.16 CDCl3
76.84
60.50
55.52
F3C
O
N
N
Bn
3t
210 200 190 180 170 160 150 140 130 120 110 100 90 80 70 60 50 40 30 20 10 0 -10
f1 (ppm)

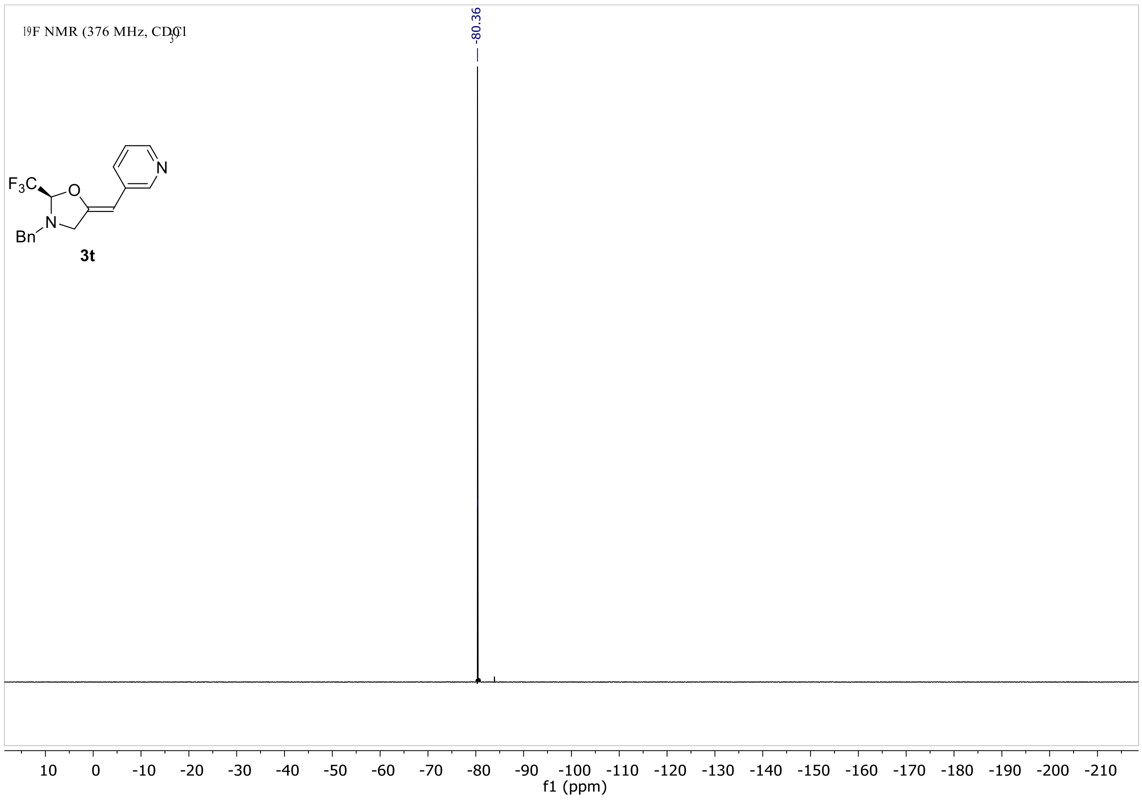
19F NMR (376 MHz, CDCl3)
-80.36
F3C
O
N
N
Bn
3t
10 0 -10 -20 -30 -40 -50 -60 -70 -80 -90 -100 -110 -120 -130 -140 -150 -160 -170 -180 -190 -200 -210
f1 (ppm)

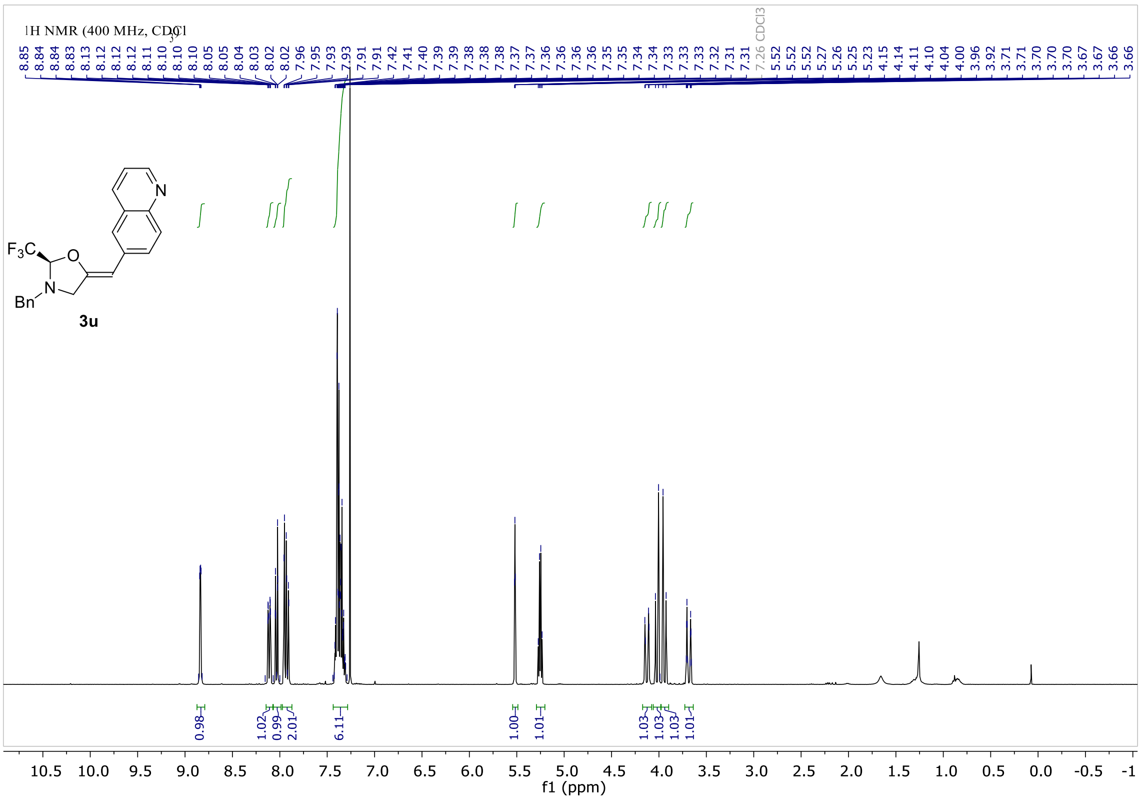
1H NMR (400 MHz, CDCl3)
3u
f1 (ppm)

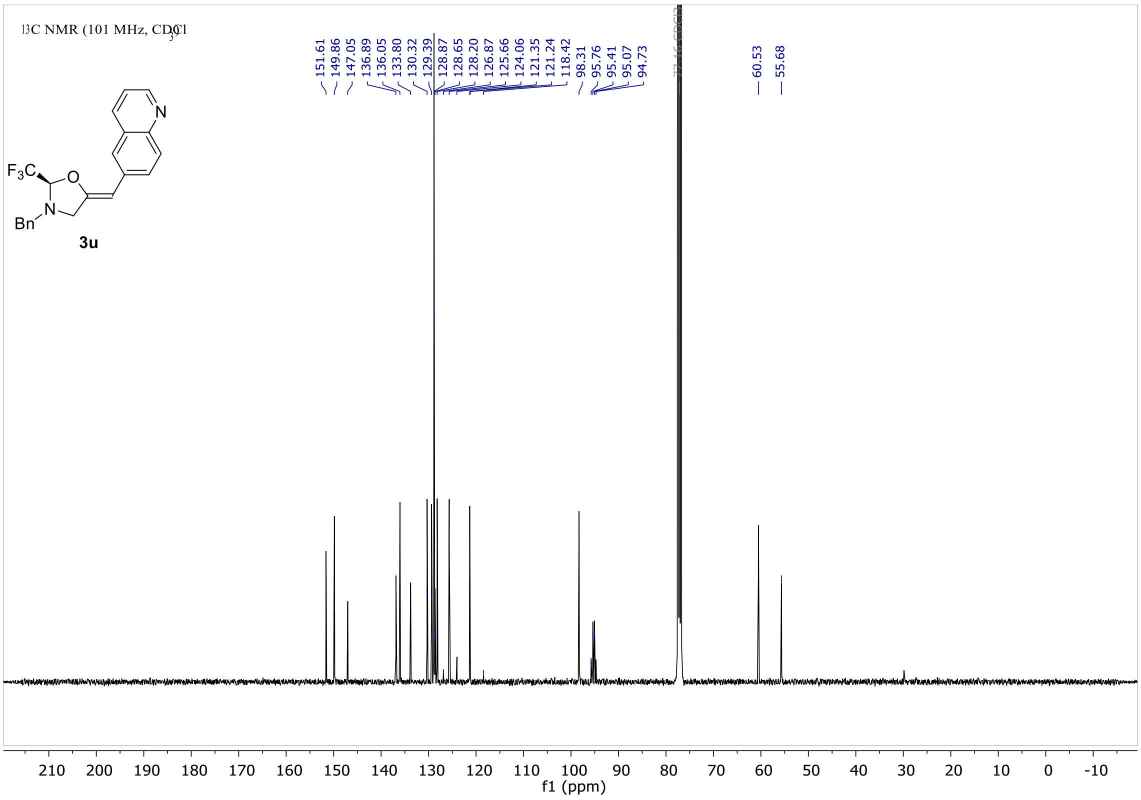
13C NMR (101 MHz, CDCl3)
3u
f1 (ppm)

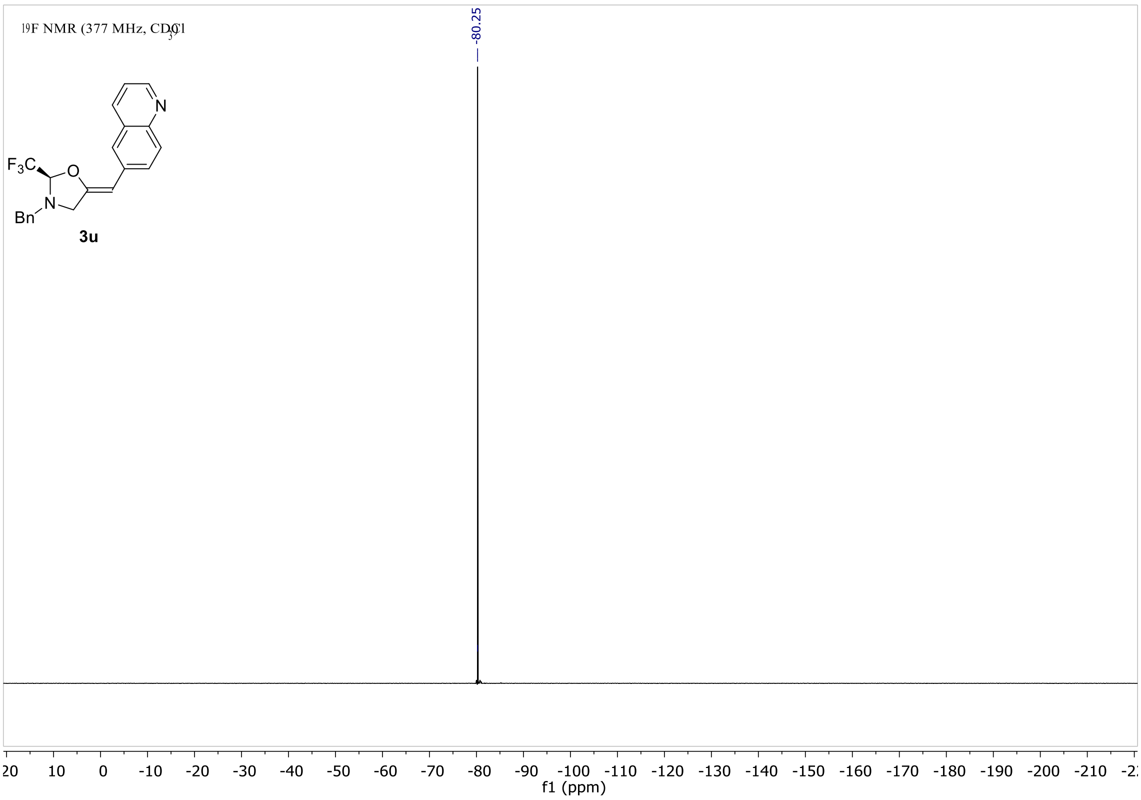
19F NMR (377 MHz, CDCl3)
-80.25
F3C
O
N
N
Bn
3u
20 10 0 -10 -20 -30 -40 -50 -60 -70 -80 -90 -100 -110 -120 -130 -140 -150 -160 -170 -180 -190 -200 -210
f1 (ppm)

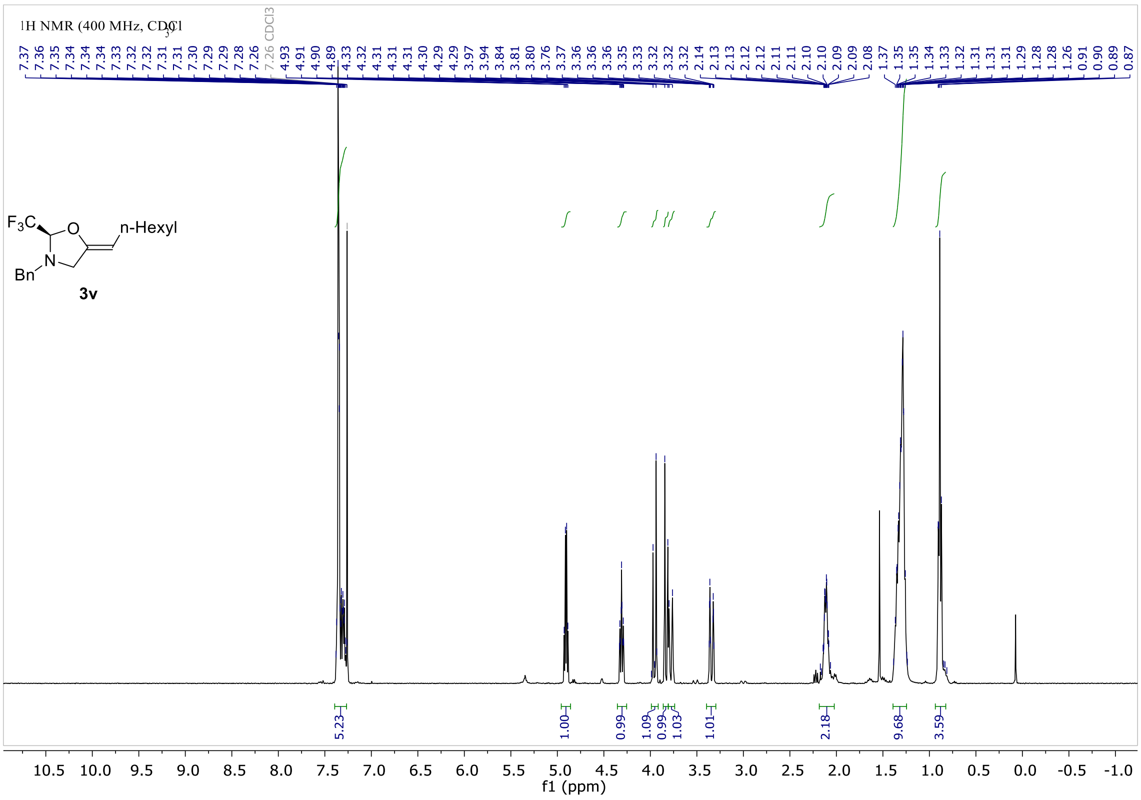
1H NMR (400 MHz, CDCl3)
7.37 7.36 7.35 7.34 7.34 7.34 7.33 7.32 7.32 7.31 7.31 7.30 7.29 7.29 7.28 7.26 CDCl3 7.26 4.93 4.91 4.90 4.89 4.33 4.32 4.31 4.31 4.31 4.30 4.29 4.29 3.97 3.94 3.84 3.81 3.80 3.76 3.37 3.36 3.36 3.35 3.33 3.32 3.32 3.32 2.14 2.13 2.13 2.12 2.12 2.11 2.11 2.10 2.10 2.09 2.09 2.08 1.37 1.35 1.35 1.34 1.33 1.32 1.31 1.31 1.31 1.29 1.28 1.28 1.26 0.91 0.90 0.89 0.87
F3C
O
n-Hexyl
N
Bn
3v
5.23 1.00 0.99 1.09 0.99 1.03 1.01 2.18 9.68 3.59
10.5 10.0 9.5 9.0 8.5 8.0 7.5 7.0 6.5 6.0 5.5 5.0 4.5 4.0 3.5 3.0 2.5 2.0 1.5 1.0 0.5 0.0 -0.5 -1.0
f1 (ppm)

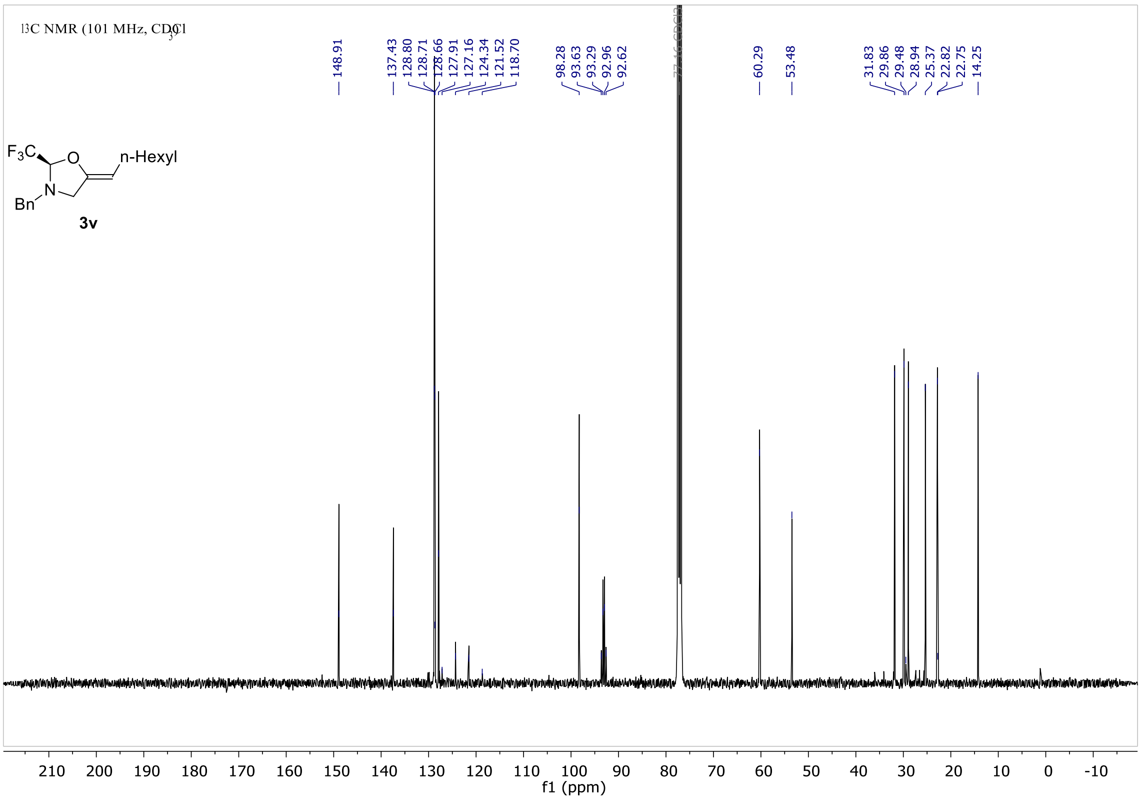
13C NMR (101 MHz, CDCl3)
148.91
137.43
128.80
128.71
128.66
127.91
127.16
124.34
121.52
118.70
98.28
93.63
93.29
92.96
92.62
60.29
53.48
31.83
29.86
29.48
28.94
25.37
22.82
22.75
14.25
F3C
O
n-Hexyl
N
Bn
3v
f1 (ppm)

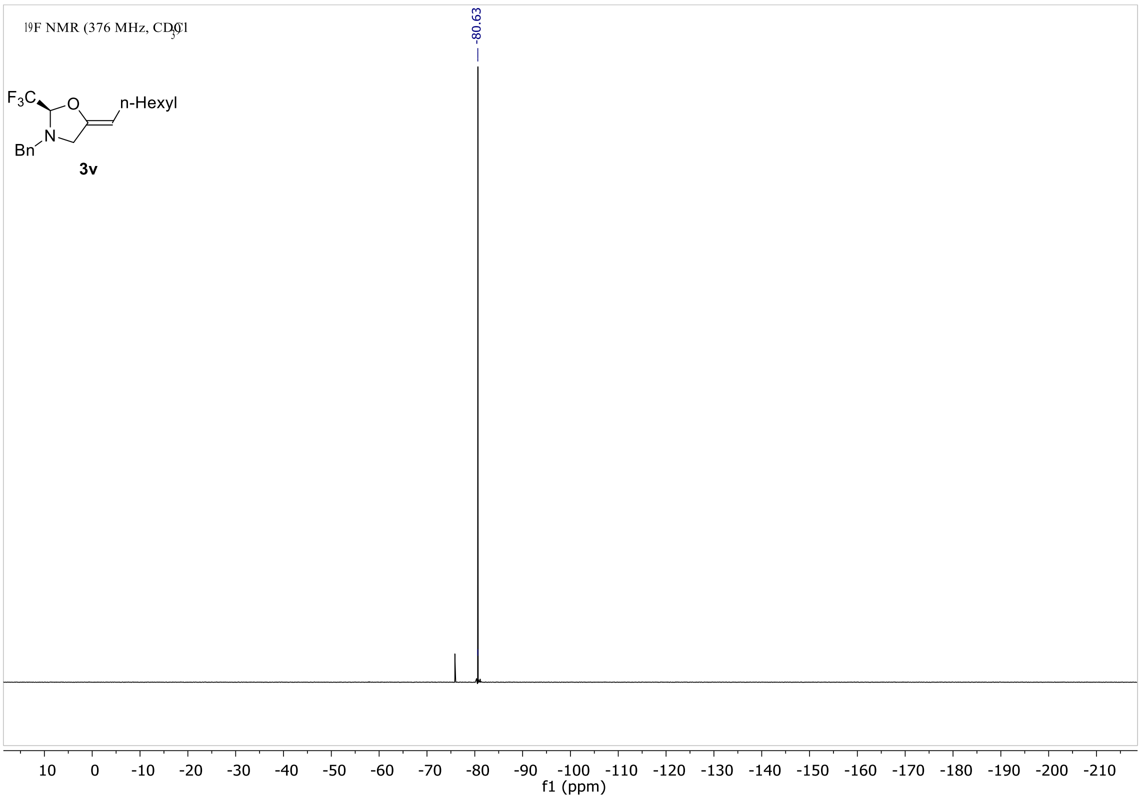
19F NMR (376 MHz, CDCl3)
-80.63
F3C
O
n-Hexyl
N
Bn
3v
f1 (ppm)

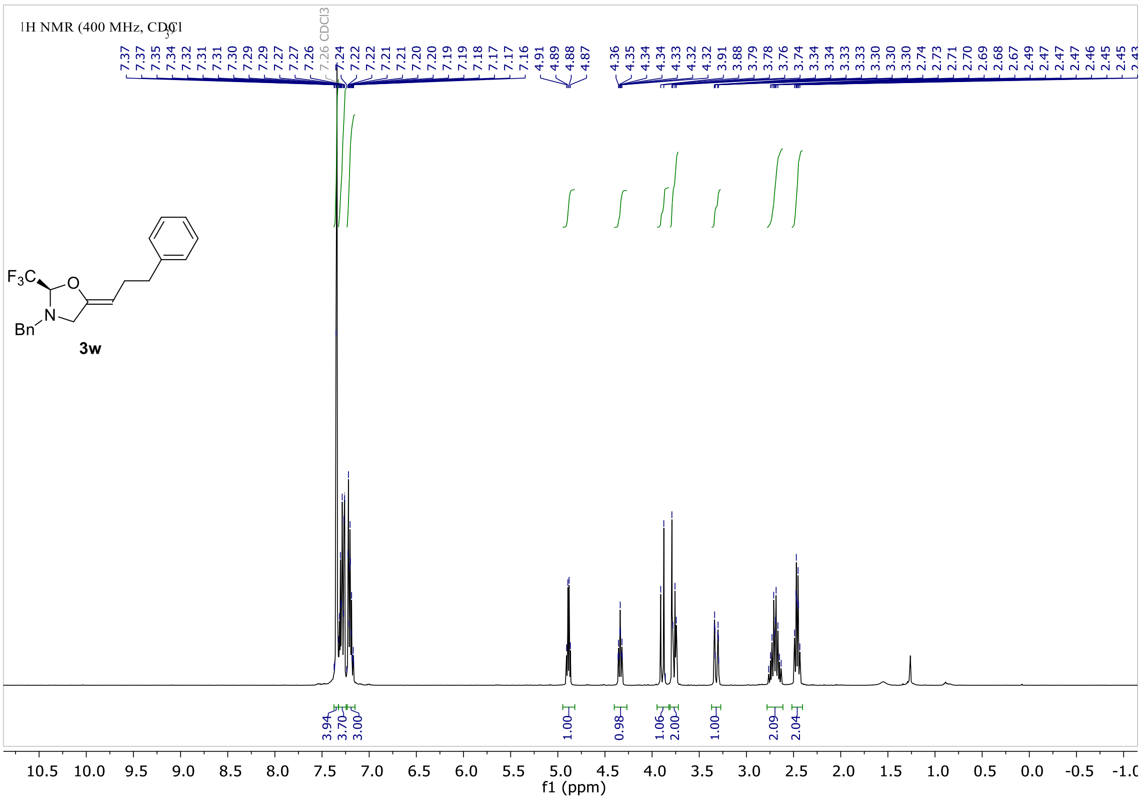
1H NMR (400 MHz, CDCl3)
3w
f1 (ppm)

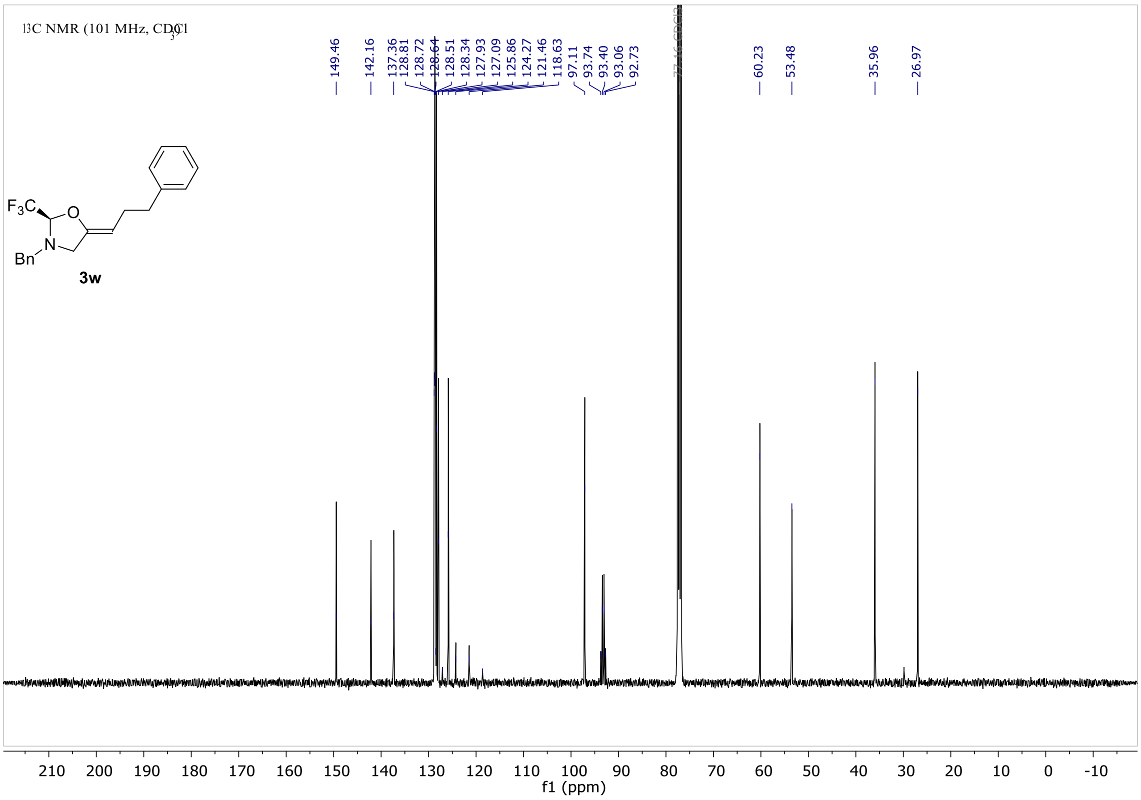
13C NMR (101 MHz, CDCl3)
3w
f1 (ppm)

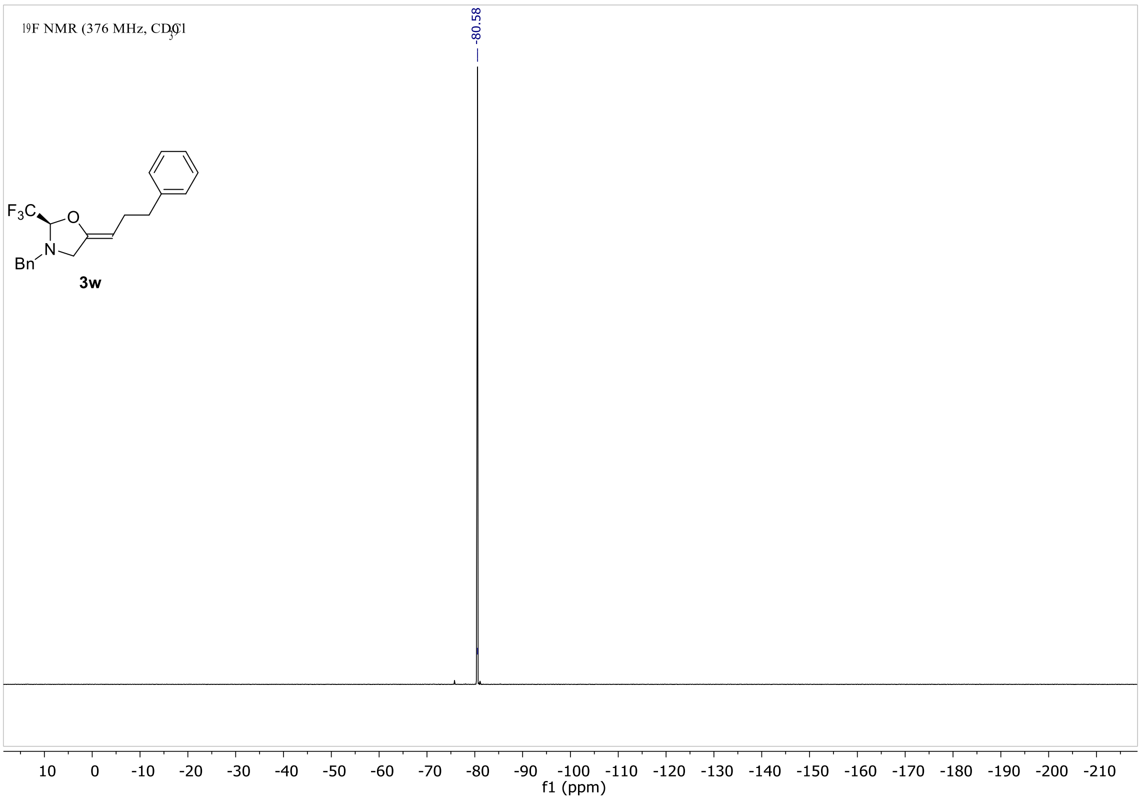

19F NMR (376 MHz, CDCl3)
-80.58
F3C
O
N
Bn
3w
10 0 -10 -20 -30 -40 -50 -60 -70 -80 -90 -100 -110 -120 -130 -140 -150 -160 -170 -180 -190 -200 -210
f1 (ppm)

1H NMR (400 MHz, Chloroform-d)

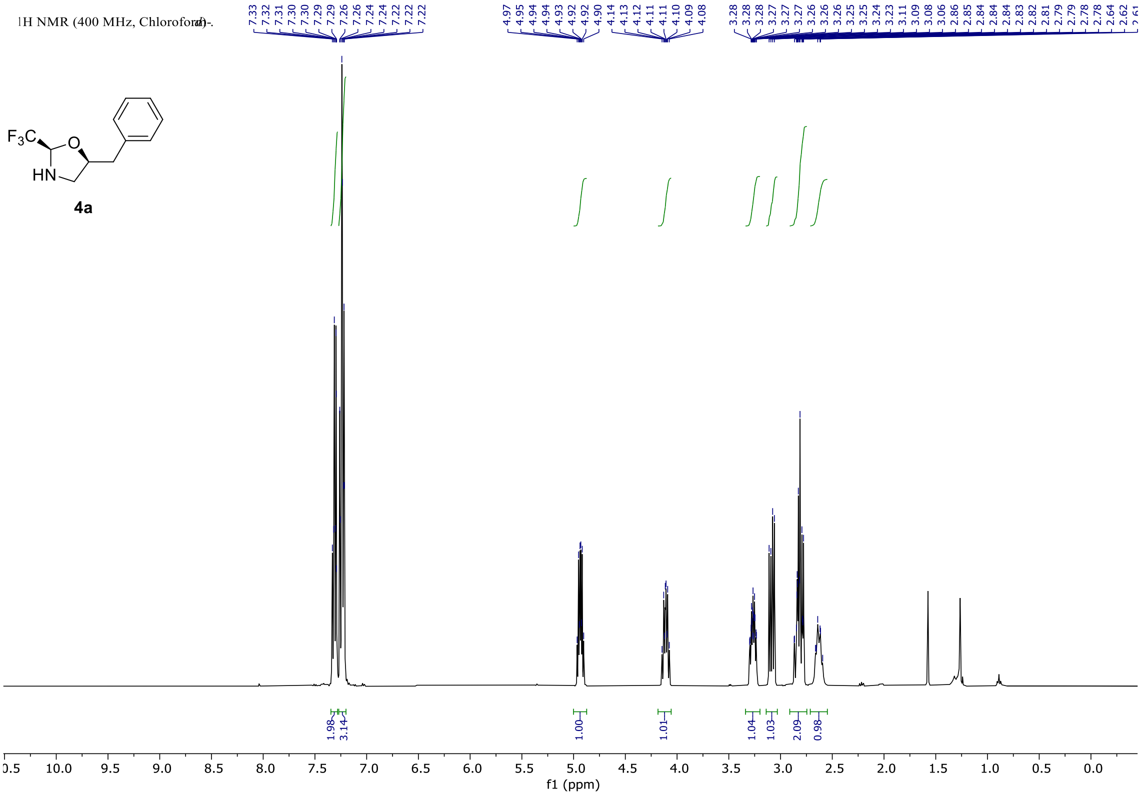

13C NMR (101 MHz, Chloroform-d)

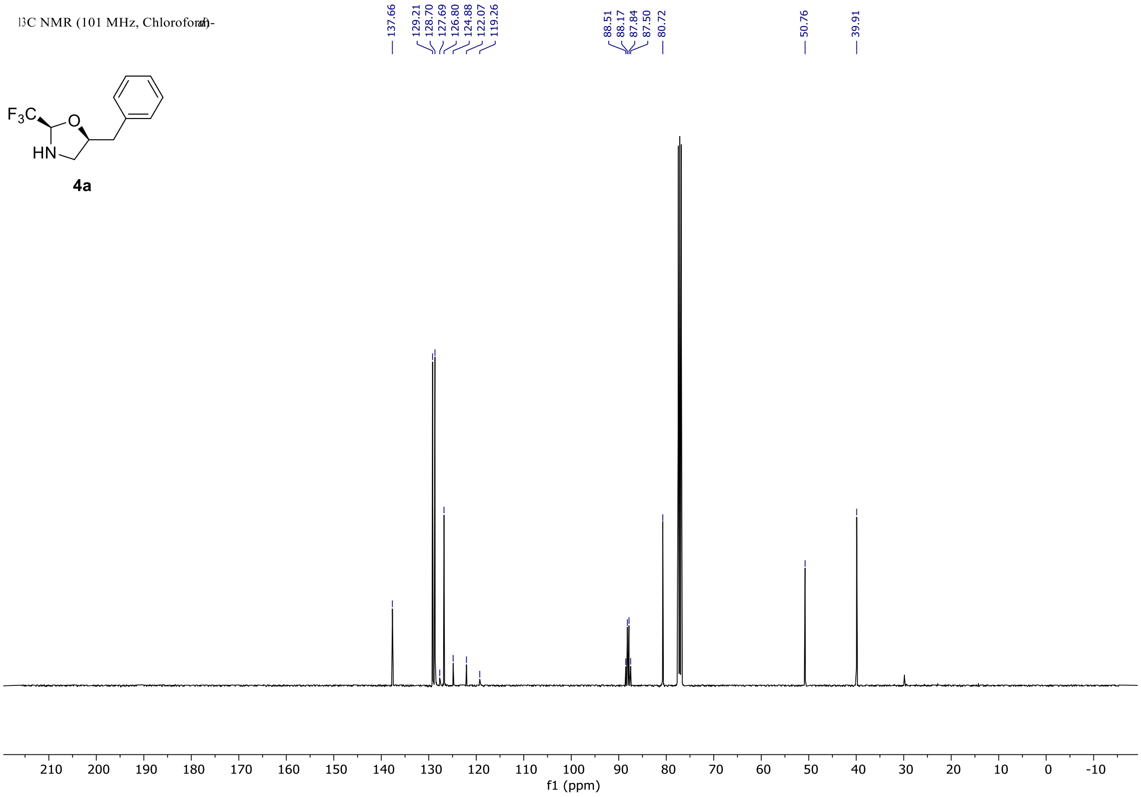

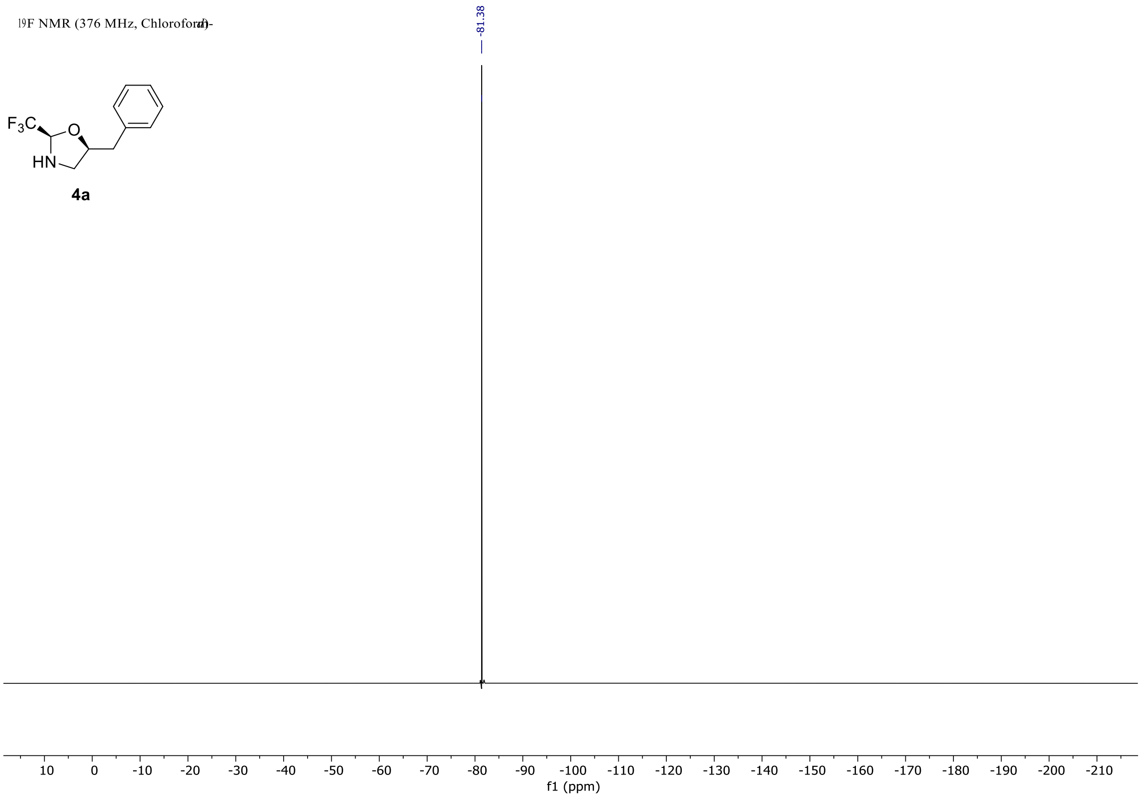

19F NMR (376 MHz, Chloroform-d)
-81.38
F3C
O
HN
4a
f1 (ppm)

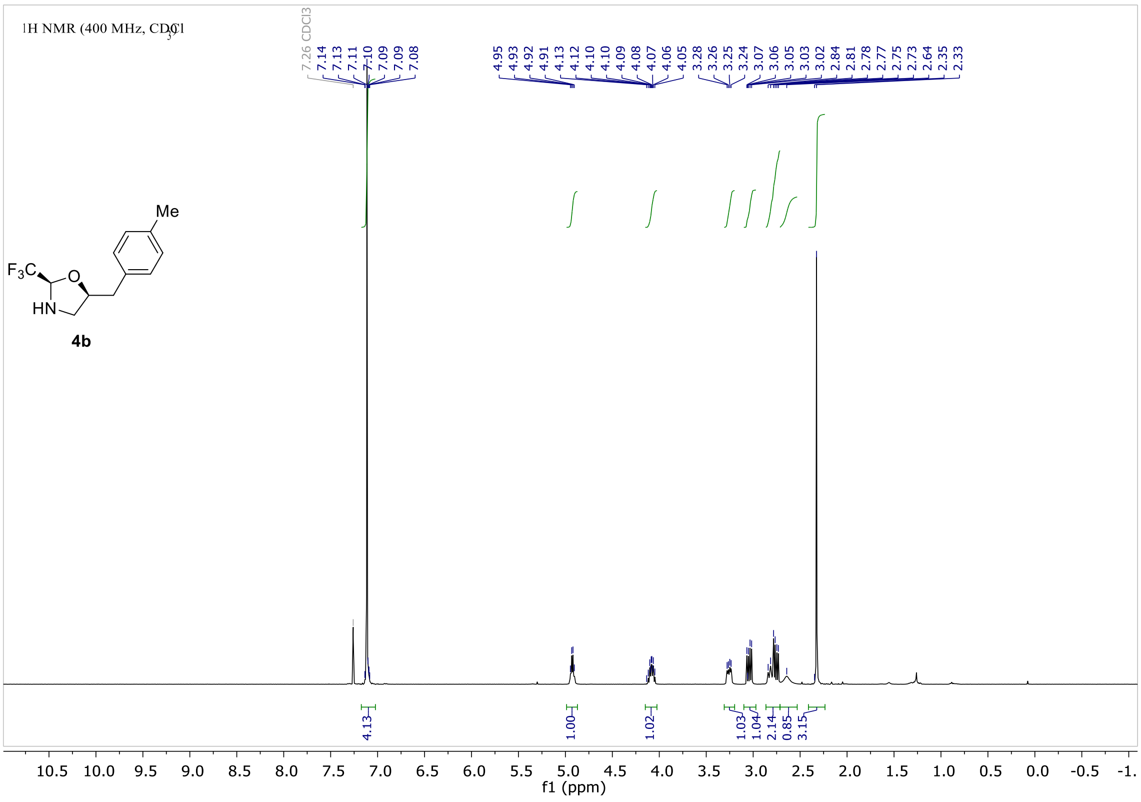

1H NMR (400 MHz, CDCl3)
7.26 CDCl3
7.14
7.13
7.11
7.10
7.09
7.09
7.08
4.95
4.93
4.92
4.91
4.13
4.12
4.10
4.10
4.09
4.08
4.07
4.06
4.05
3.28
3.26
3.25
3.24
3.07
3.06
3.05
3.03
3.02
2.84
2.81
2.78
2.77
2.75
2.73
2.64
2.35
2.33
Me
F3C
O
HN
4b
4.13
1.00
1.02
1.03
1.04
2.14
0.85
3.15
f1 (ppm)

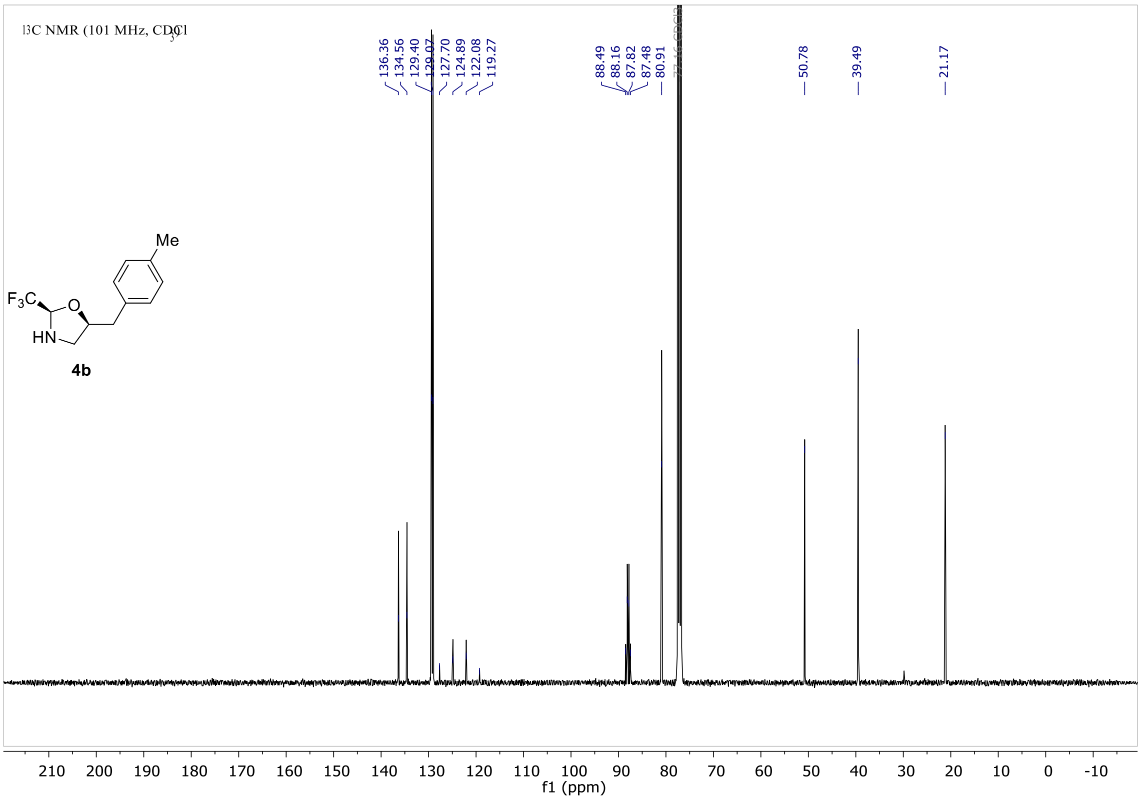
13C NMR (101 MHz, CDCl3)
136.36
134.56
129.40
129.07
127.70
124.89
122.08
119.27
88.49
88.16
87.82
87.48
80.91
50.78
39.49
21.17
Me
F3C
O
HN
4b
f1 (ppm)

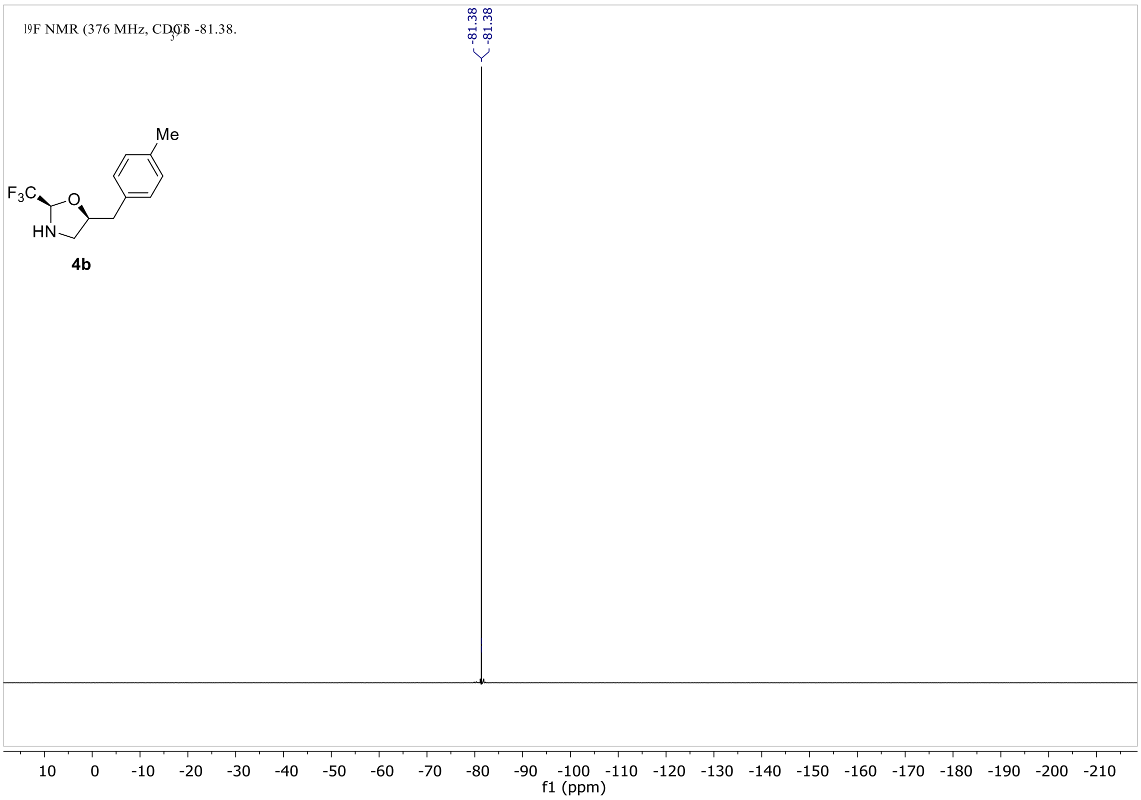
19F NMR (376 MHz, CDCl3) δ -81.38.
-81.38
-81.38
Me
F3C
O
HN
4b
f1 (ppm)

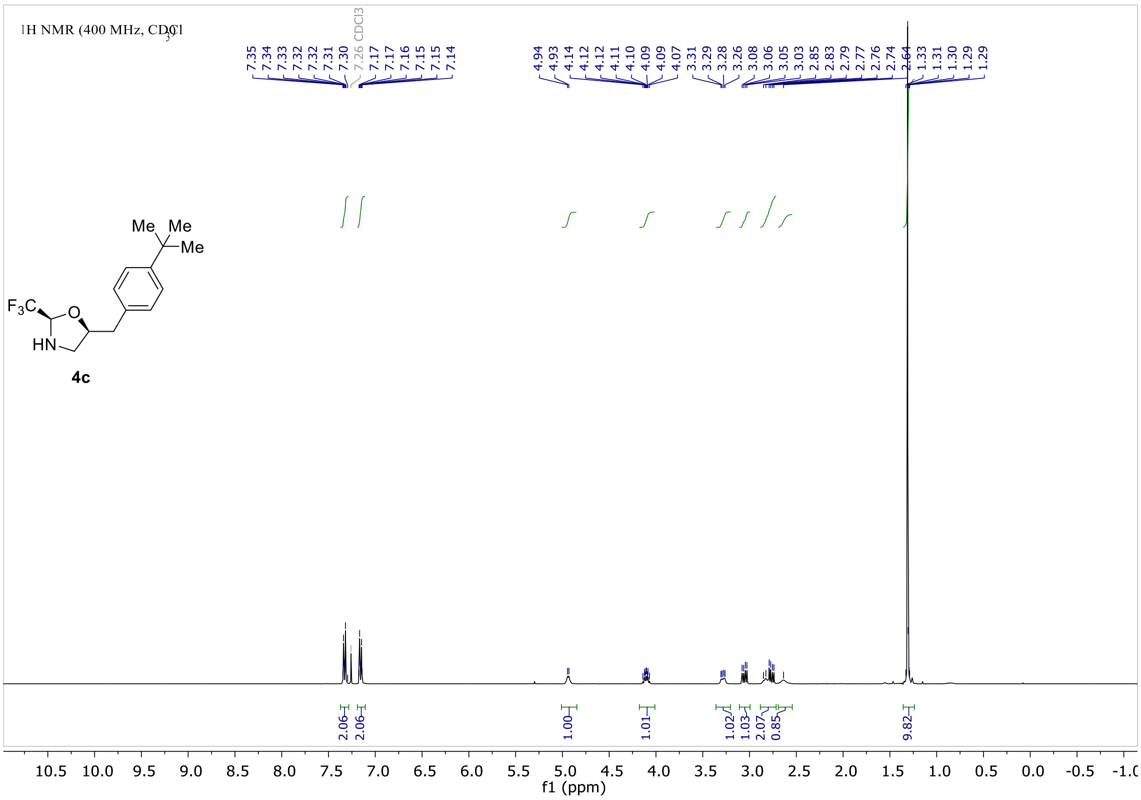
1H NMR (400 MHz, CDCl3)
4c
f1 (ppm)

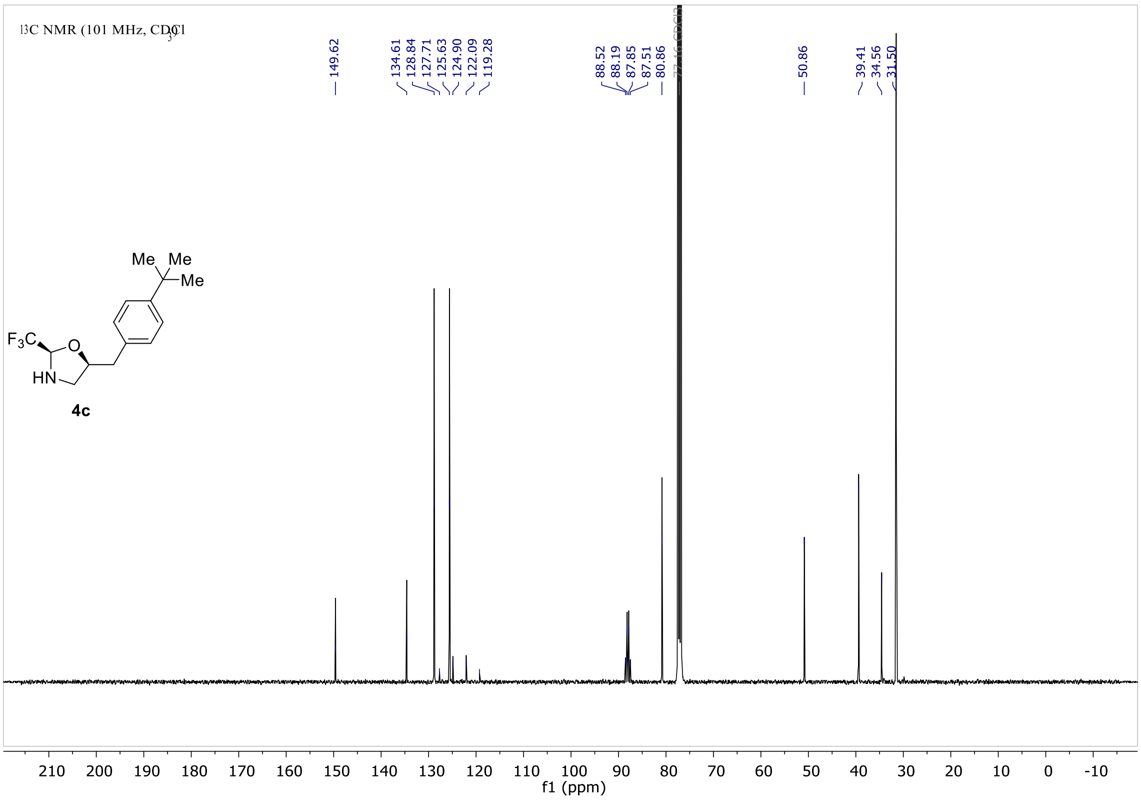
13C NMR (101 MHz, CDCl3)
4c
f1 (ppm)

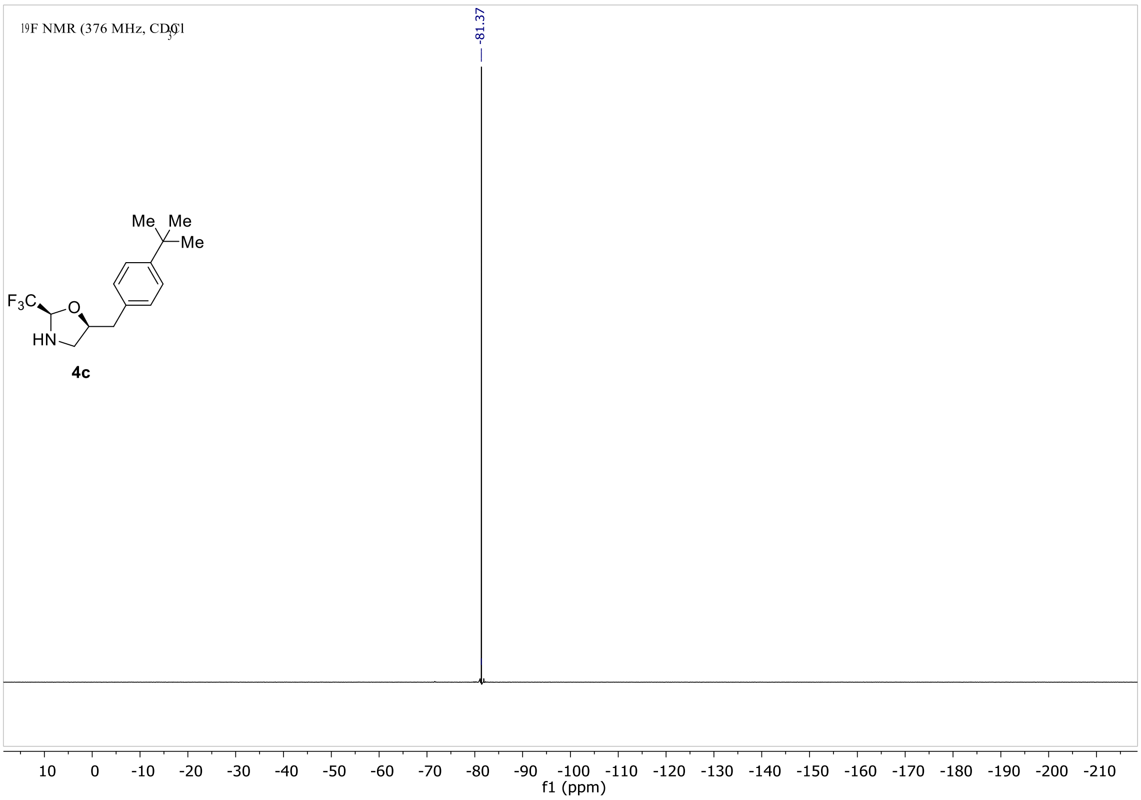

19F NMR (376 MHz, CDCl3)
-81.37
Me
Me
Me
F3C
O
HN
4c
10 0 -10 -20 -30 -40 -50 -60 -70 -80 -90 -100 -110 -120 -130 -140 -150 -160 -170 -180 -190 -200 -210
f1 (ppm)

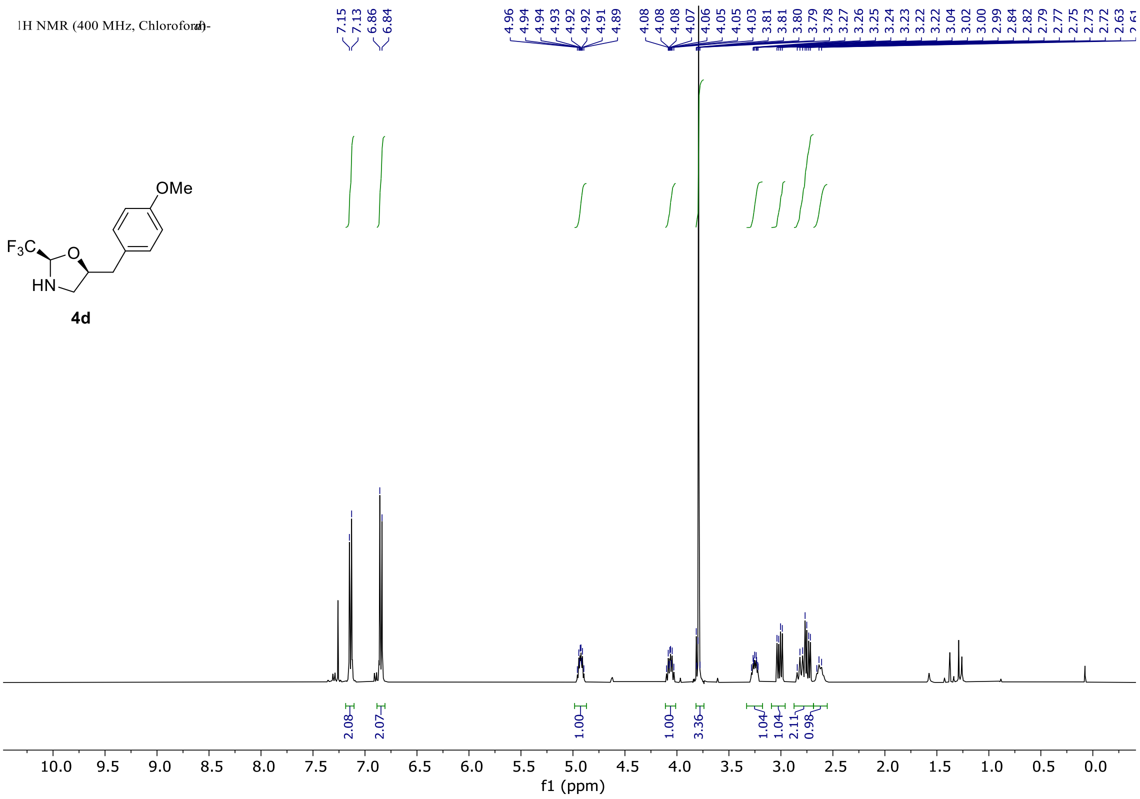

1H NMR (400 MHz, Chloroform-d)
7.15 7.13 6.86 6.84
4.96 4.94 4.94 4.93 4.92 4.92 4.91 4.89
4.08 4.08 4.07 4.06 4.06 4.05 4.05 4.03 3.81 3.81 3.80 3.79 3.78 3.27 3.26 3.25 3.24 3.23 3.22 3.04 3.02 3.00 2.99 2.84 2.82 2.79 2.77 2.75 2.73 2.72 2.63
OMe
F3C
O
HN
4d
2.08 2.07 1.00 1.00 3.36 1.04 1.04 2.11 0.98
10.0 9.5 9.0 8.5 8.0 7.5 7.0 6.5 6.0 5.5 5.0 4.5 4.0 3.5 3.0 2.5 2.0 1.5 1.0 0.5 0.0
f1 (ppm)

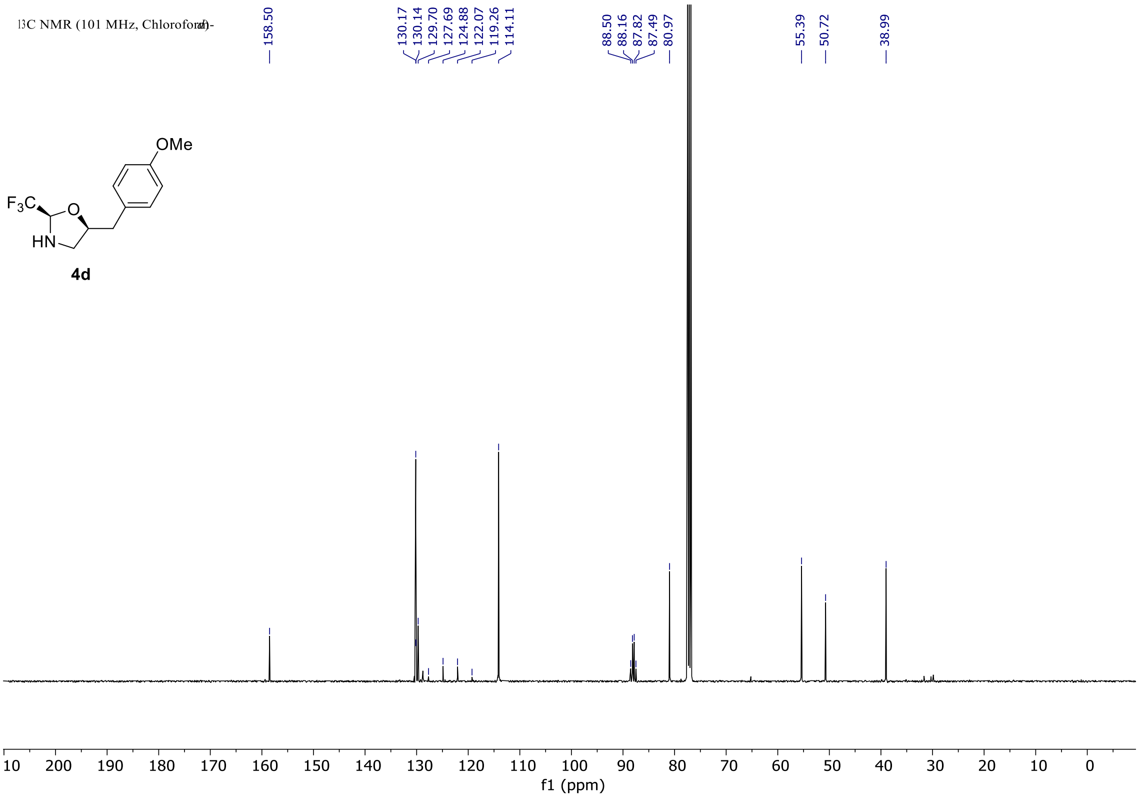
13C NMR (101 MHz, Chloroform-d)
158.50
130.17
130.14
129.70
127.69
124.88
122.07
119.26
114.11
88.50
88.16
87.82
87.49
80.97
55.39
50.72
38.99
OMe
F3C
O
HN
4d
f1 (ppm)

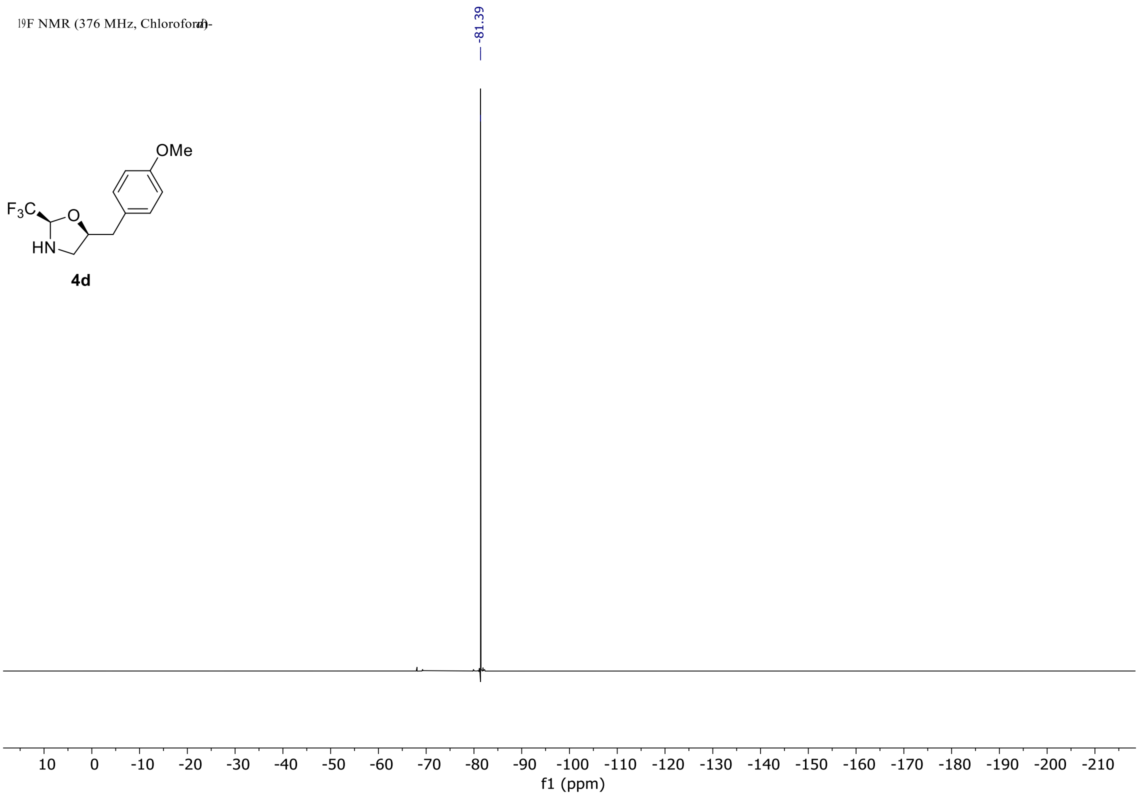
19F NMR (376 MHz, Chloroform-d)
-81.39
OMe
F3C
O
HN
4d
f1 (ppm)

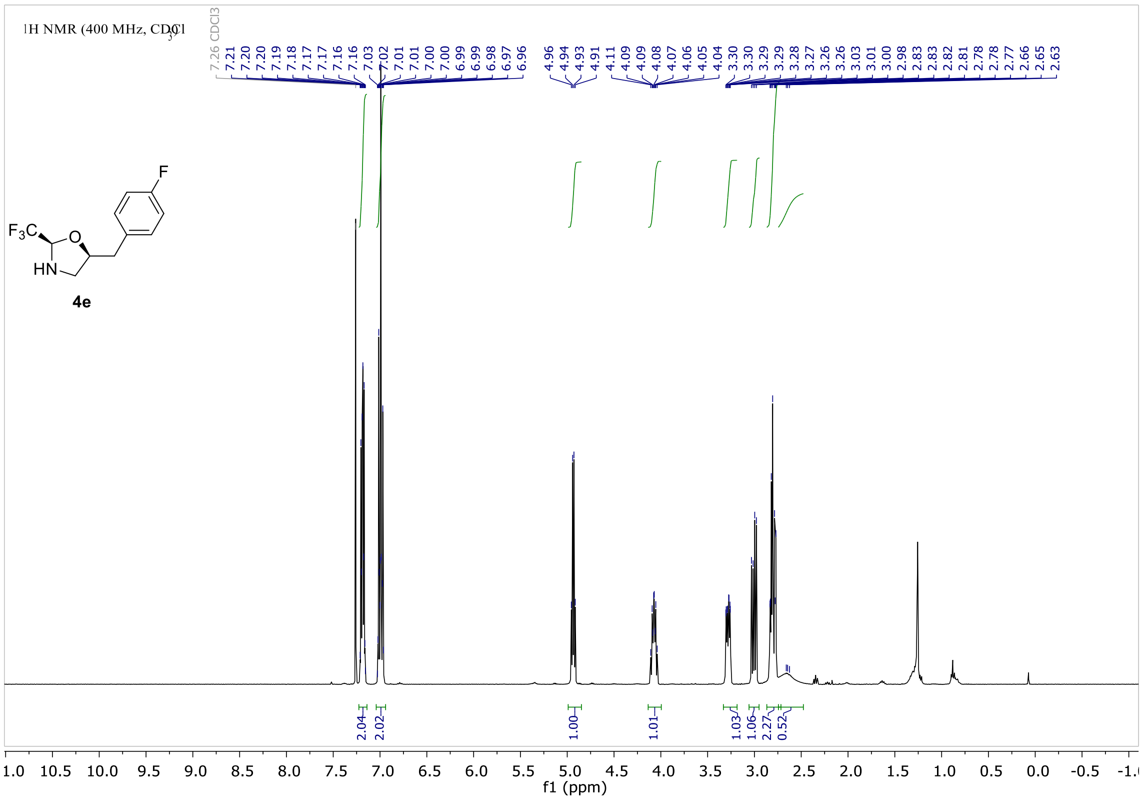
1H NMR (400 MHz, CDCl3)
4e
f1 (ppm)

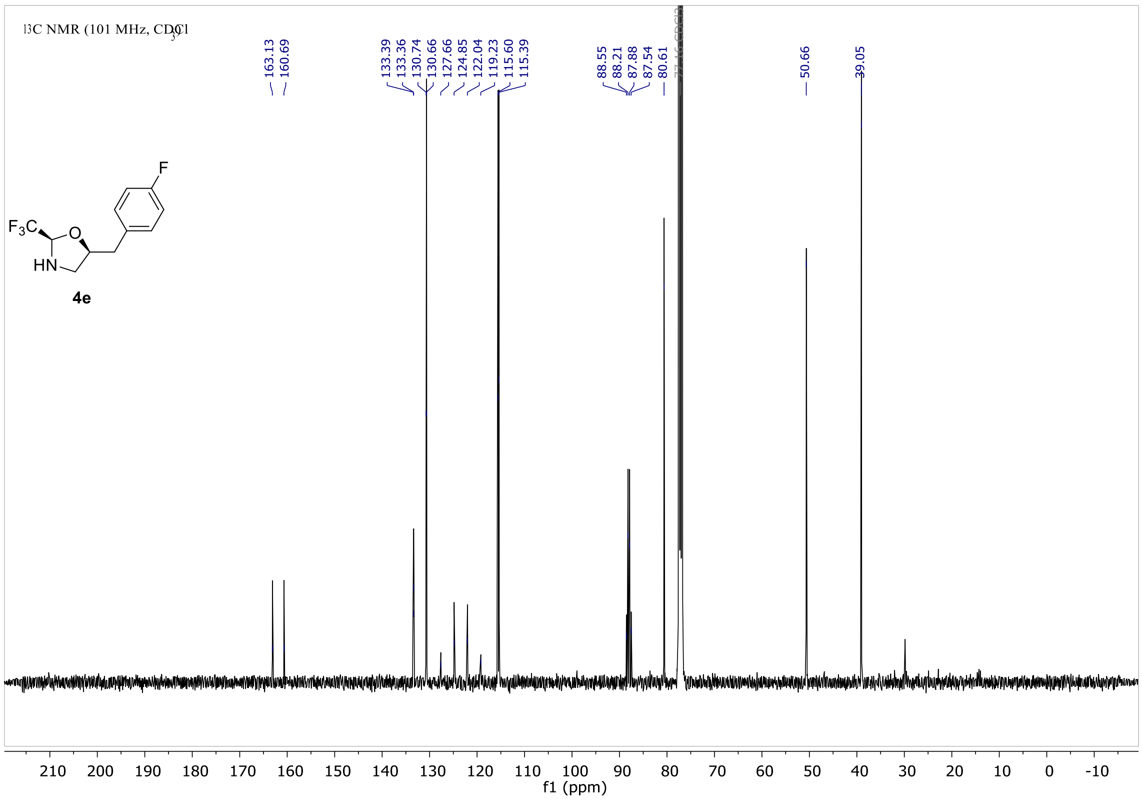
13C NMR (101 MHz, CDCl3)
4e
f1 (ppm)

19F NMR (376 MHz, CDCl3)

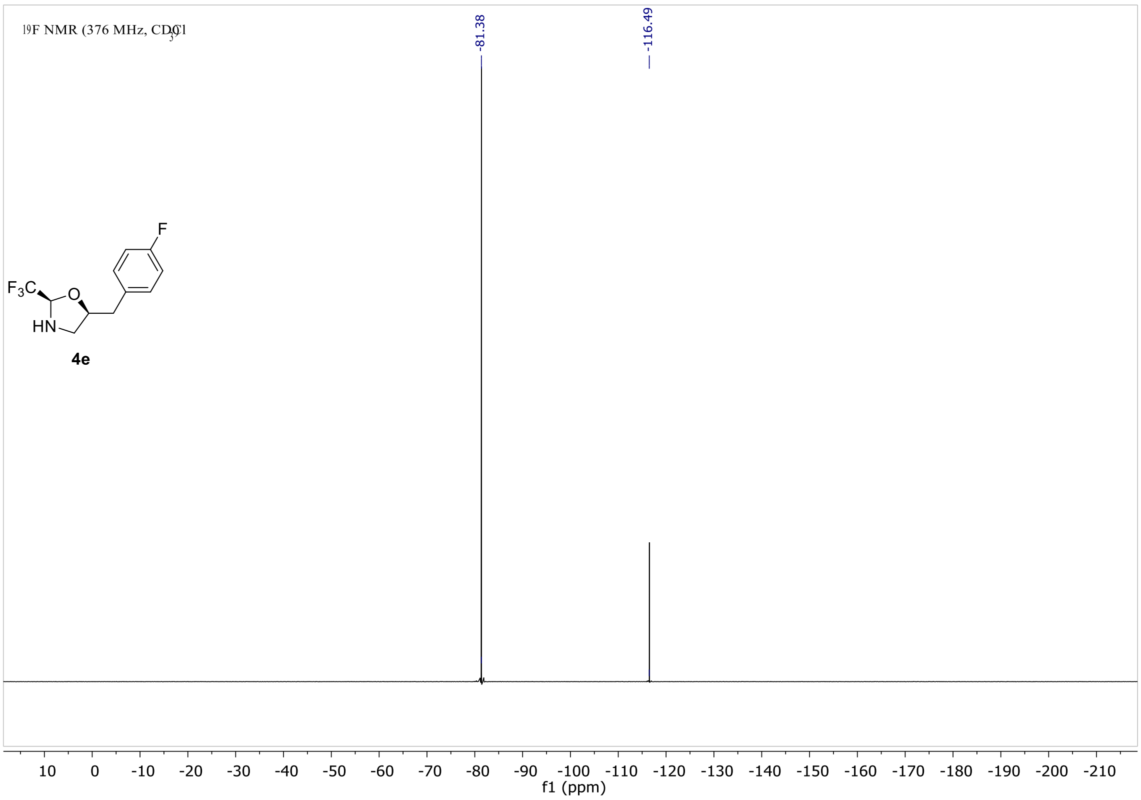

1H NMR (400 MHz, Chloroform-d)

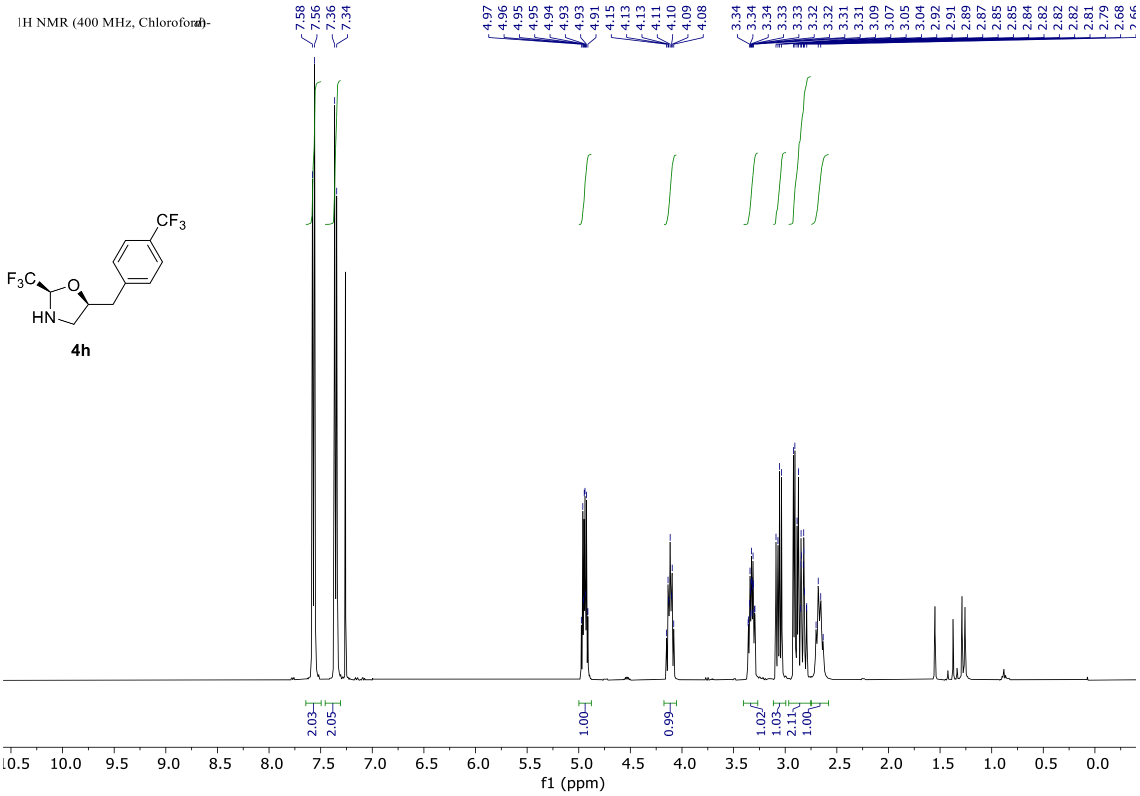

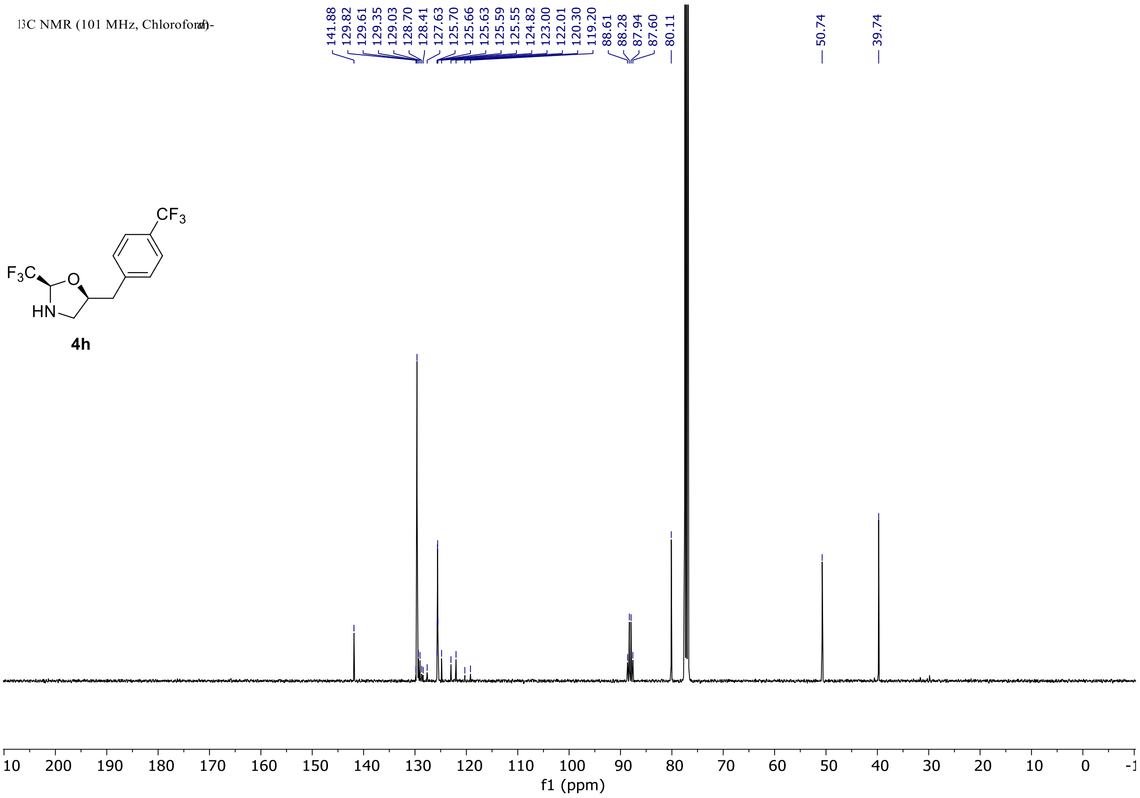
13C NMR (101 MHz, Chloroform-d)
141.88
129.82
129.61
129.35
129.03
128.70
128.41
127.63
125.70
125.66
125.63
125.59
125.55
124.82
123.00
122.01
120.30
119.20
88.61
88.28
87.94
87.60
80.11
50.74
39.74
CF3
F3C
O
HN
4h
f1 (ppm)

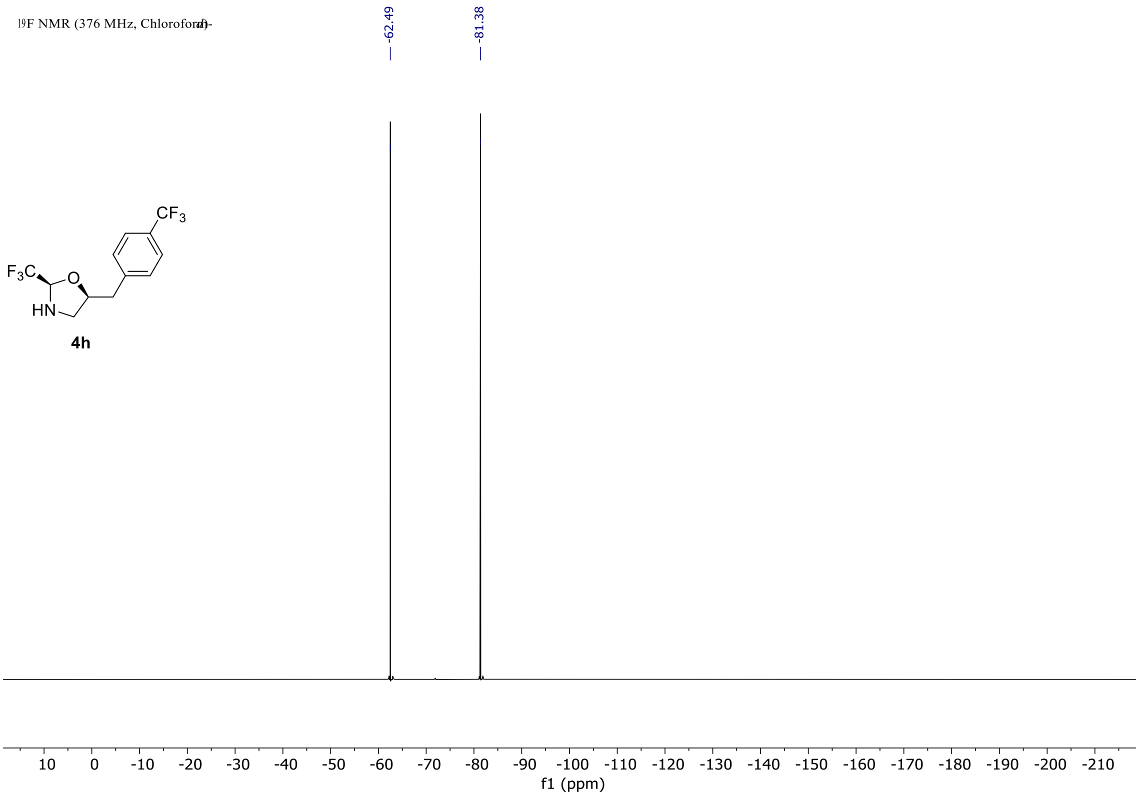
19F NMR (376 MHz, Chloroform-d)
-62.49
-81.38
CF3
F3C
O
HN
4h
f1 (ppm)

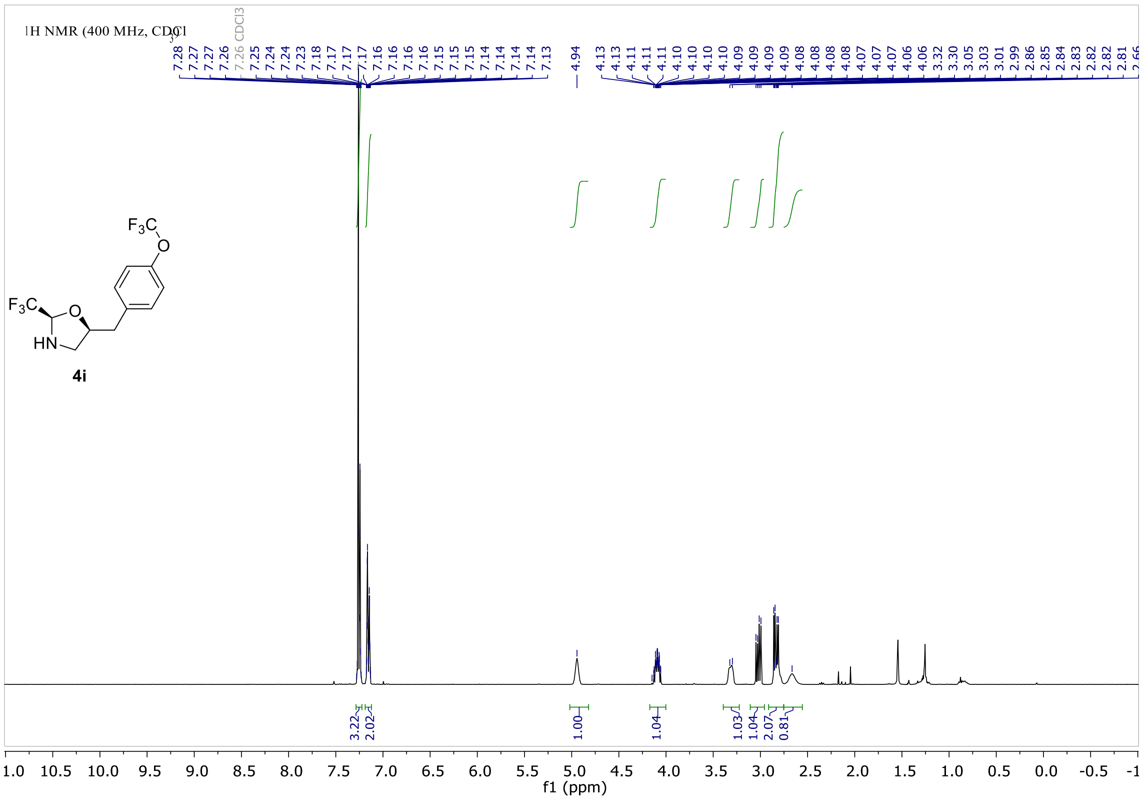
1H NMR (400 MHz, CDCl3)
4i
f1 (ppm)

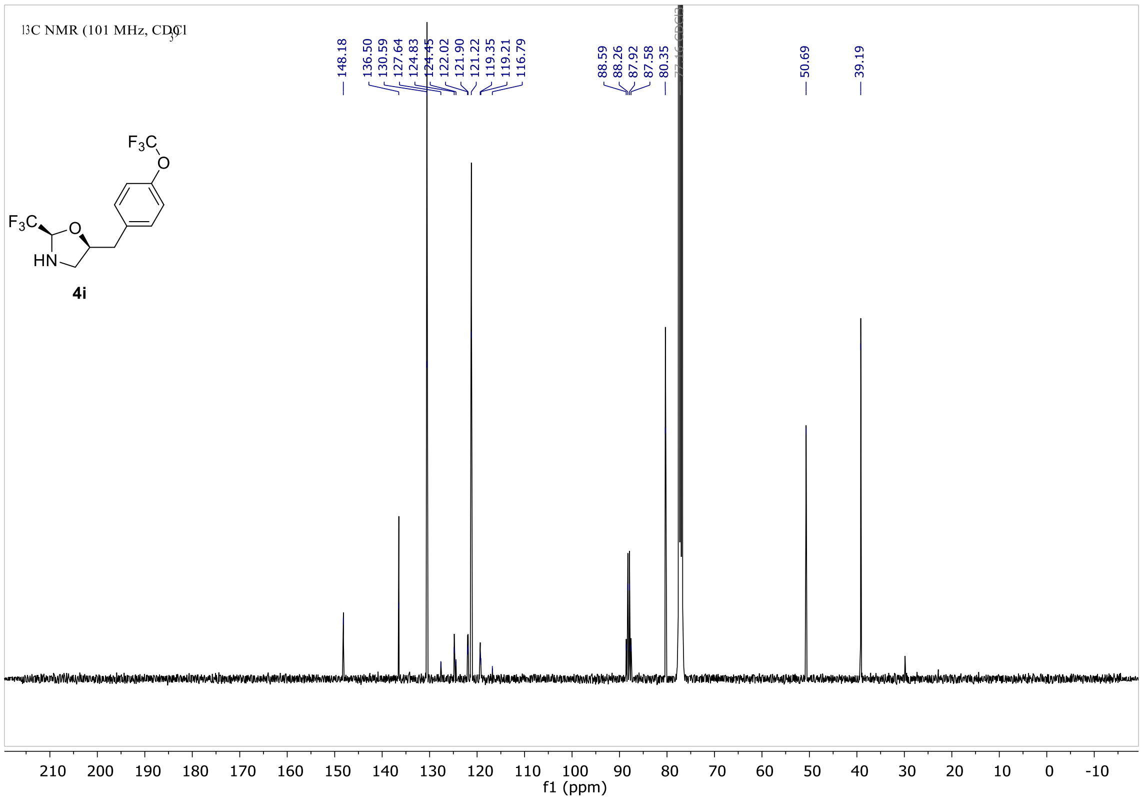
13C NMR (101 MHz, CDCl3)
4i
f1 (ppm)

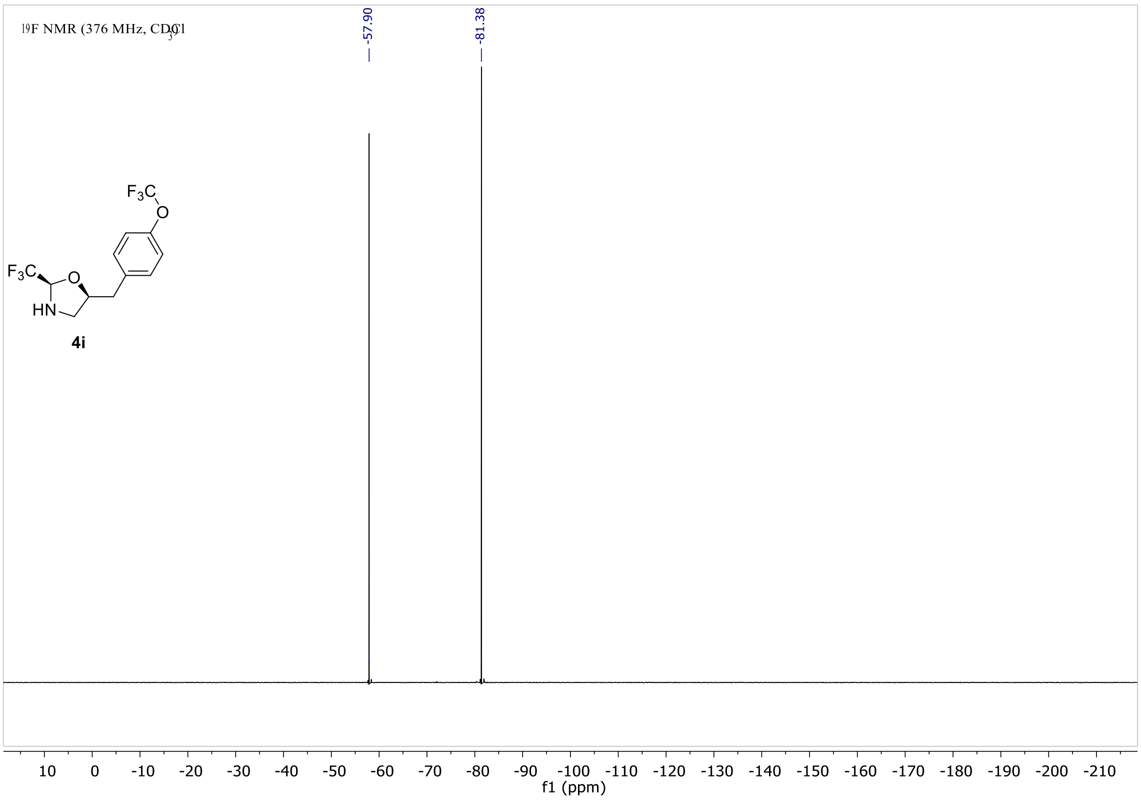
19F NMR (376 MHz, CDCl3)
-57.90
-81.38
F3C
O
HN
4i
f1 (ppm)

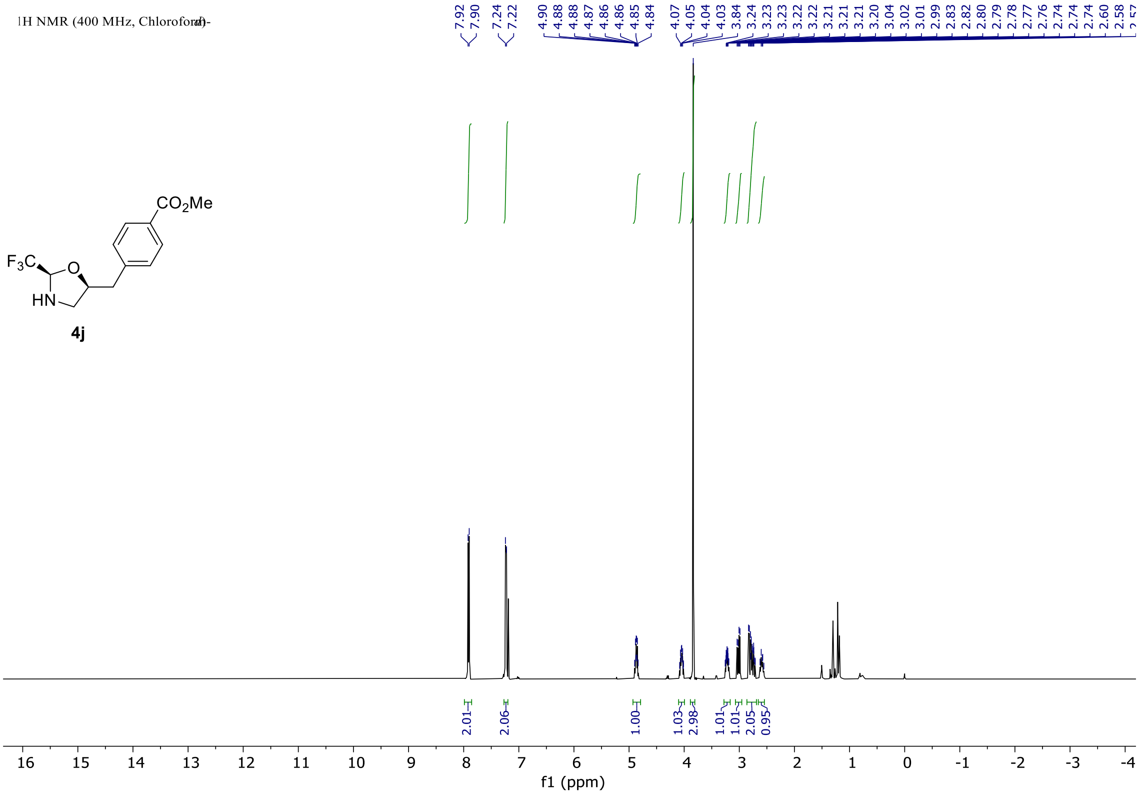
1H NMR (400 MHz, Chloroform-d)
7.92 7.90 7.24 7.22 4.90 4.88 4.88 4.87 4.86 4.86 4.85 4.84 4.07 4.05 4.04 4.03 3.84 3.24 3.23 3.22 3.22 3.21 3.21 3.21 3.20 3.04 3.02 3.01 2.99 2.83 2.82 2.80 2.79 2.78 2.77 2.76 2.74 2.74 2.74 2.60 2.58
CO2Me
F3C
O
HN
4j
2.01 2.06 1.00 1.03 2.98 1.01 1.01 2.05 0.95
f1 (ppm)

13C NMR (101 MHz, Chloroform-d)

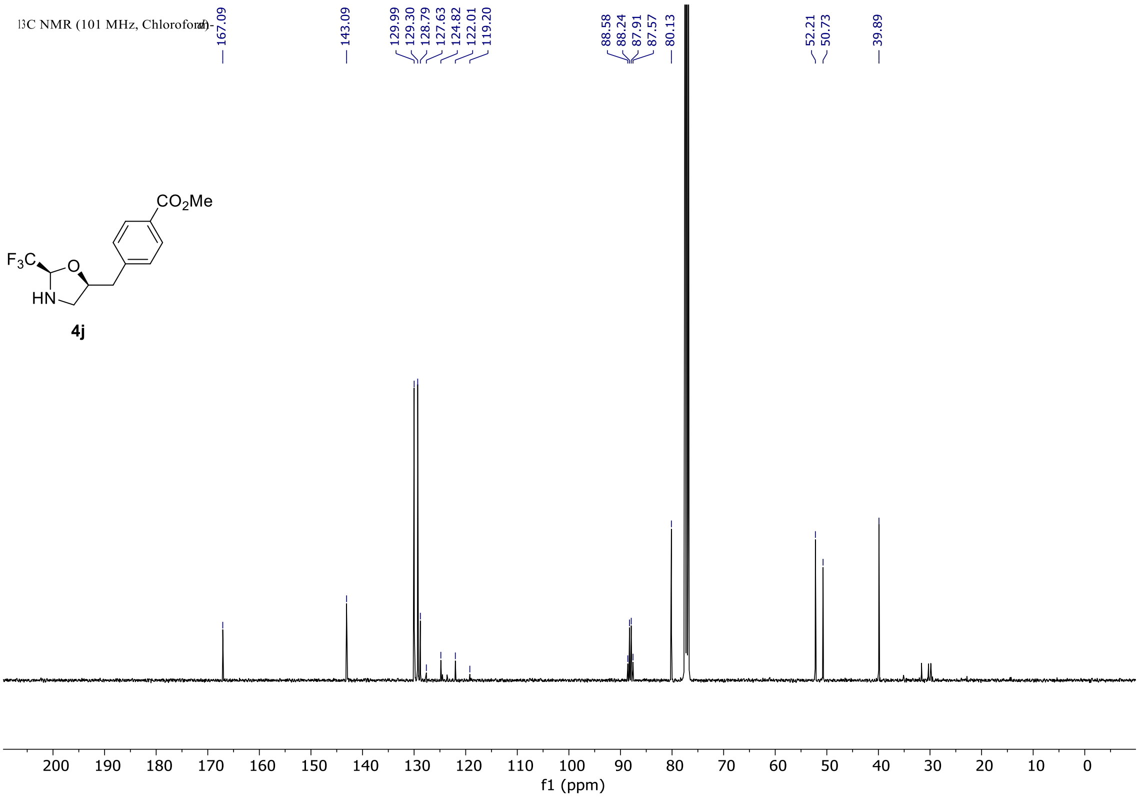

19F NMR (376 MHz, Chloroform-d)

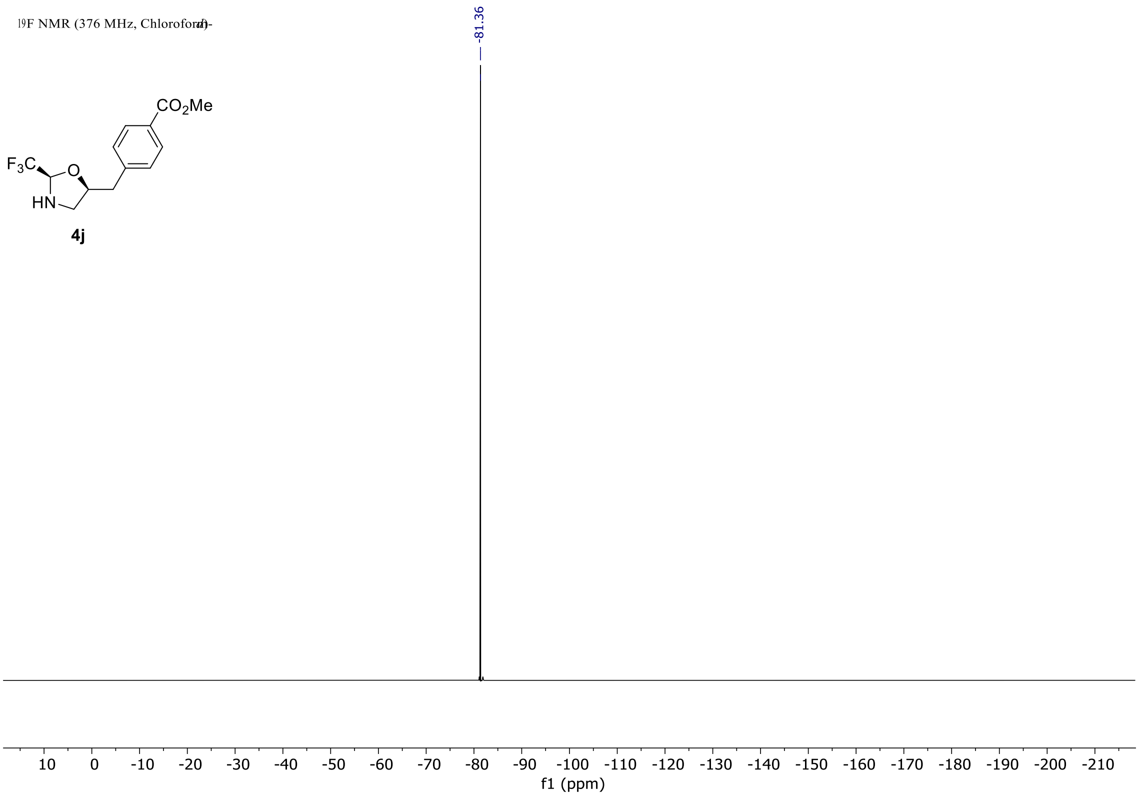

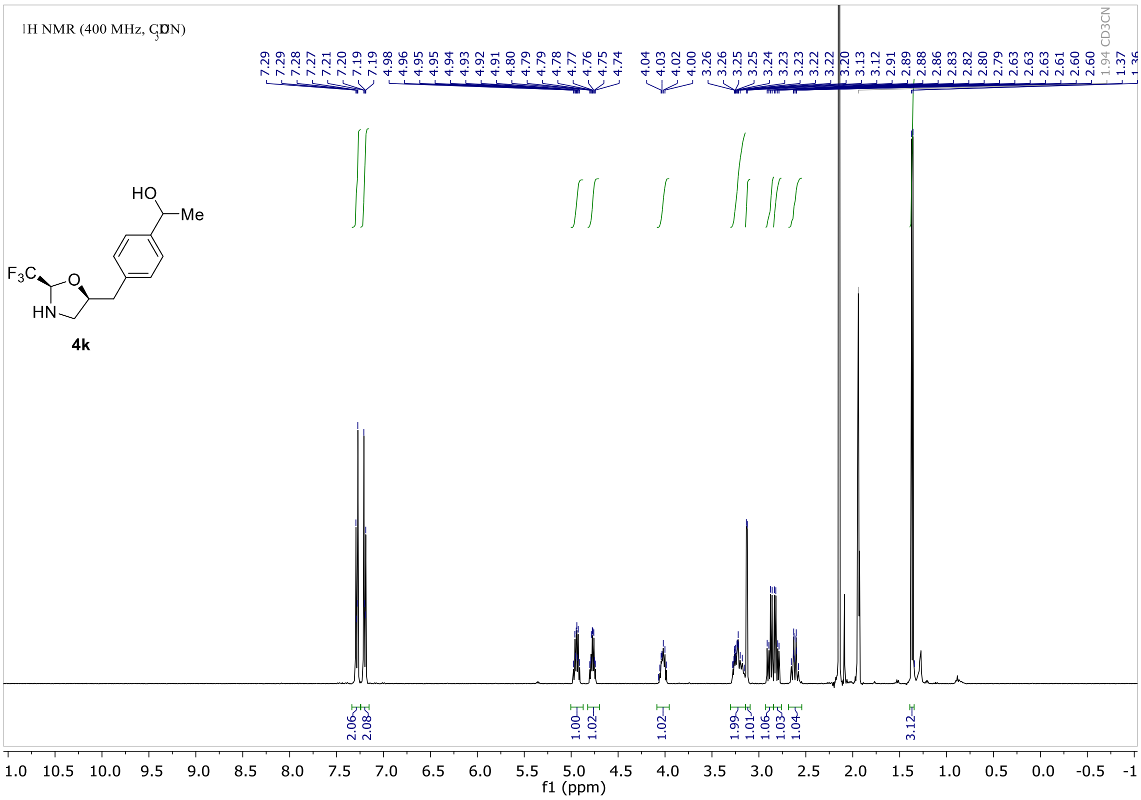
1H NMR (400 MHz, CD3CN)
4k
f1 (ppm)

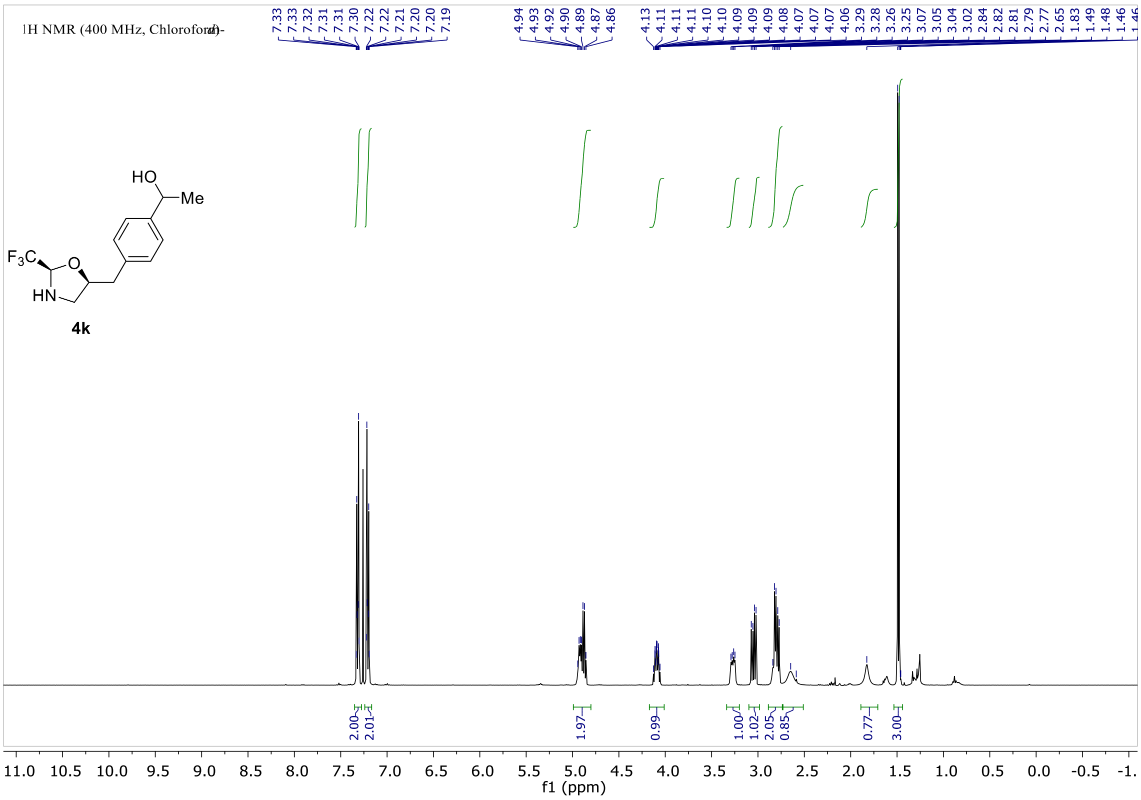
1H NMR (400 MHz, Chloroform-d)
4k
f1 (ppm)

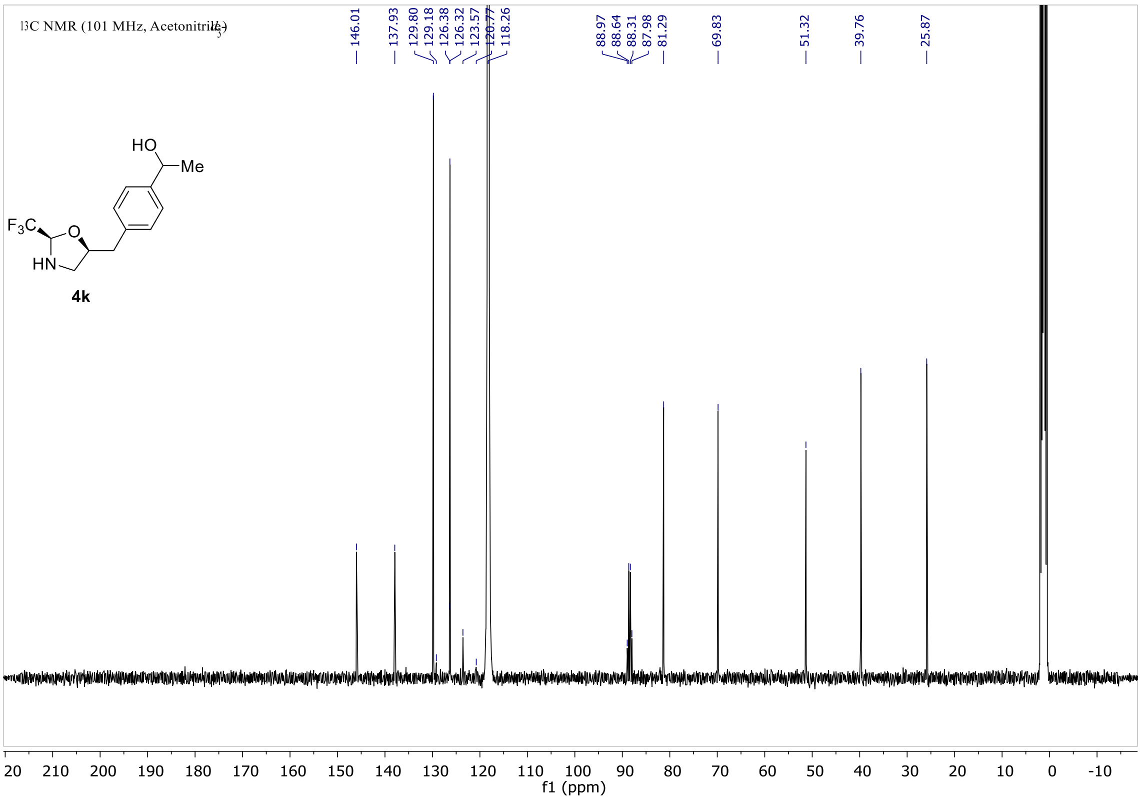
13C NMR (101 MHz, Acetonitrile-d3)
146.01
137.93
129.80
129.18
126.38
126.32
123.57
120.77
118.26
88.97
88.64
88.31
87.98
81.29
69.83
51.32
39.76
25.87
HO
Me
F3C
O
HN
4k
f1 (ppm)

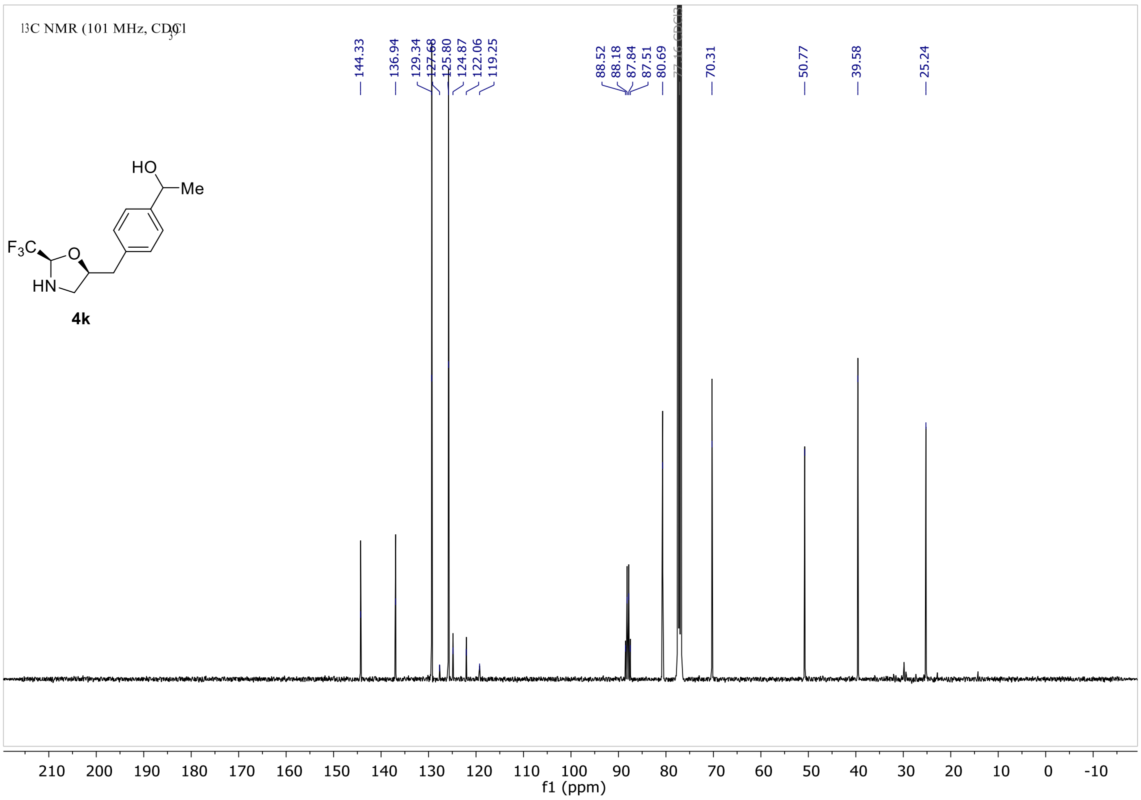
13C NMR (101 MHz, CDCl3)
144.33
136.94
129.34
127.68
125.80
124.87
122.06
119.25
88.52
88.18
87.84
87.51
80.69
70.31
50.77
39.58
25.24
HO
Me
F3C
O
HN
4k
f1 (ppm)

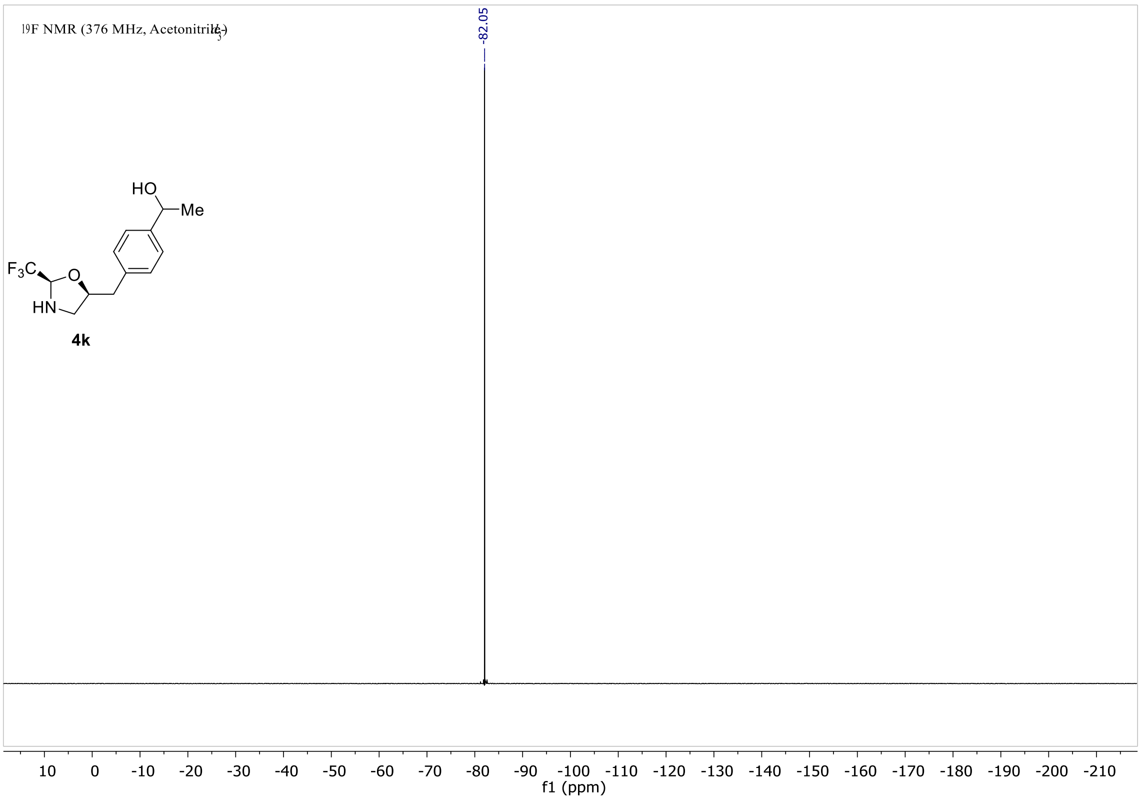
19F NMR (376 MHz, Acetonitrile-d3)
-82.05
HO
Me
F3C
O
HN
4k
10 0 -10 -20 -30 -40 -50 -60 -70 -80 -90 -100 -110 -120 -130 -140 -150 -160 -170 -180 -190 -200 -210
f1 (ppm)

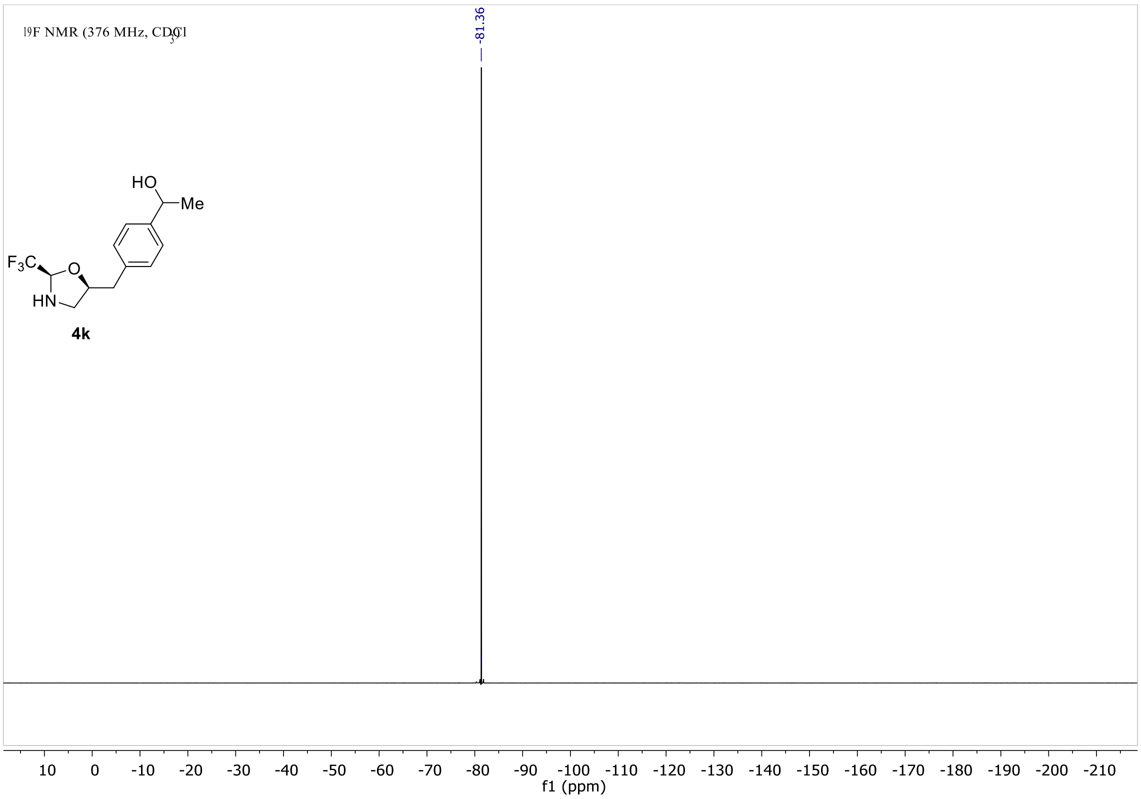
19F NMR (376 MHz, CDCl3
-81.36
HO
Me
F3C
O
HN
4k
10 0 -10 -20 -30 -40 -50 -60 -70 -80 -90 -100 -110 -120 -130 -140 -150 -160 -170 -180 -190 -200 -210
f1 (ppm)

1H NMR (400 MHz, Chloroform-d)

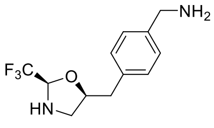

4l

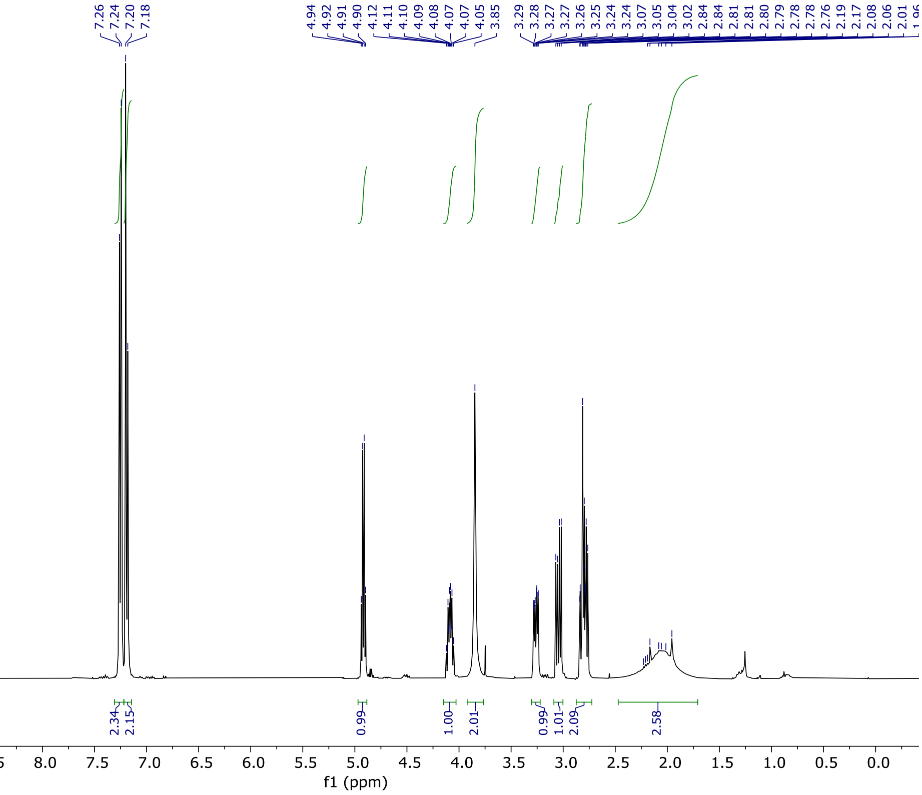

13C NMR (101 MHz, Chloroform-d)

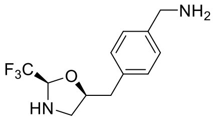

4l

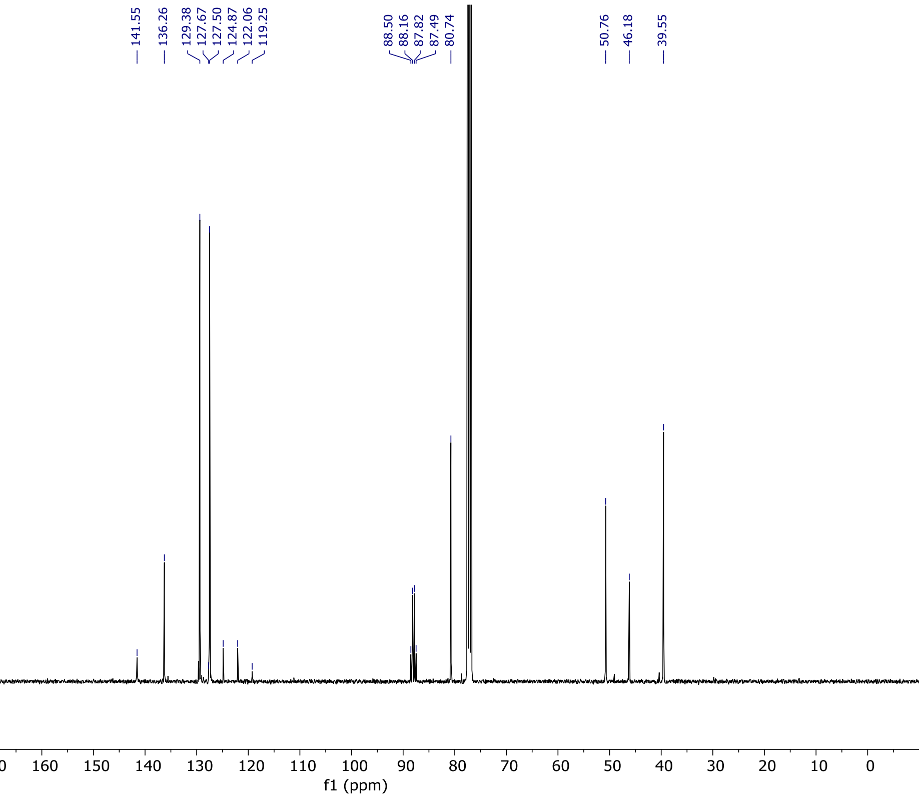

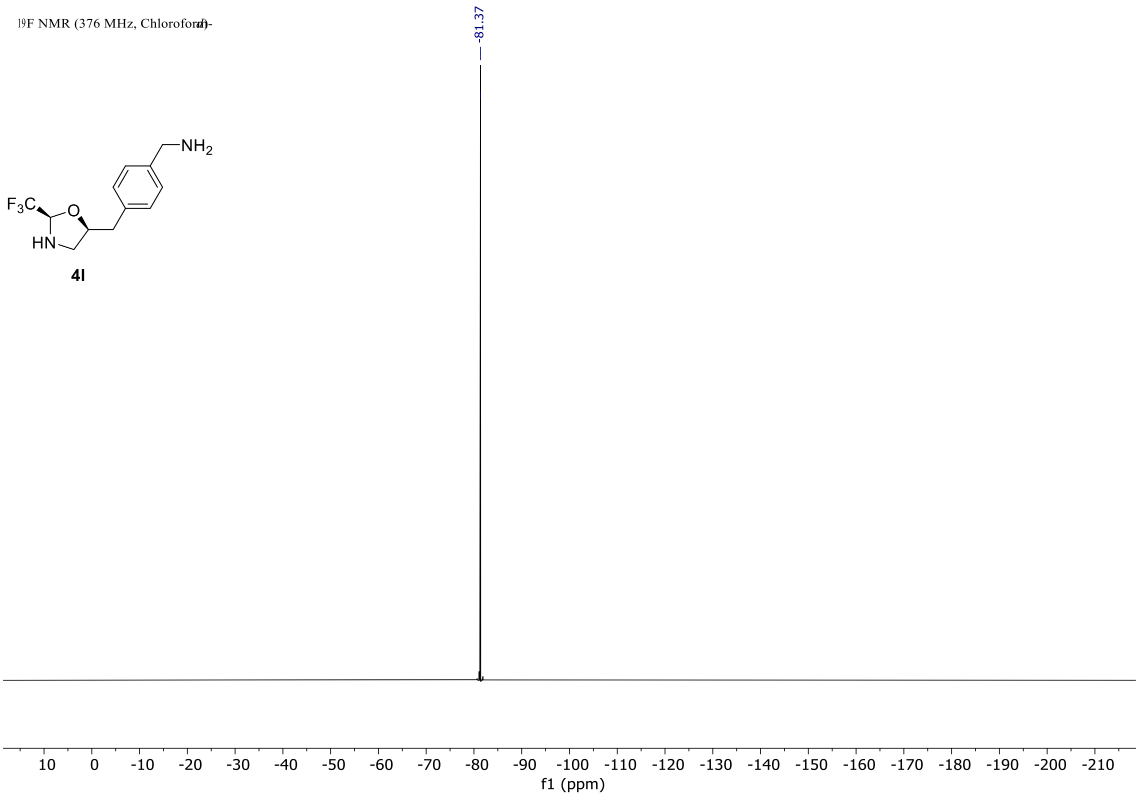
19F NMR (376 MHz, Chloroform-d)
-81.37
NH2
F3C
O
HN
4l
10
0
-10
-20
-30
-40
-50
-60
-70
-80
-90
-100
-110
-120
-130
-140
-150
-160
-170
-180
-190
-200
-210
f1 (ppm)

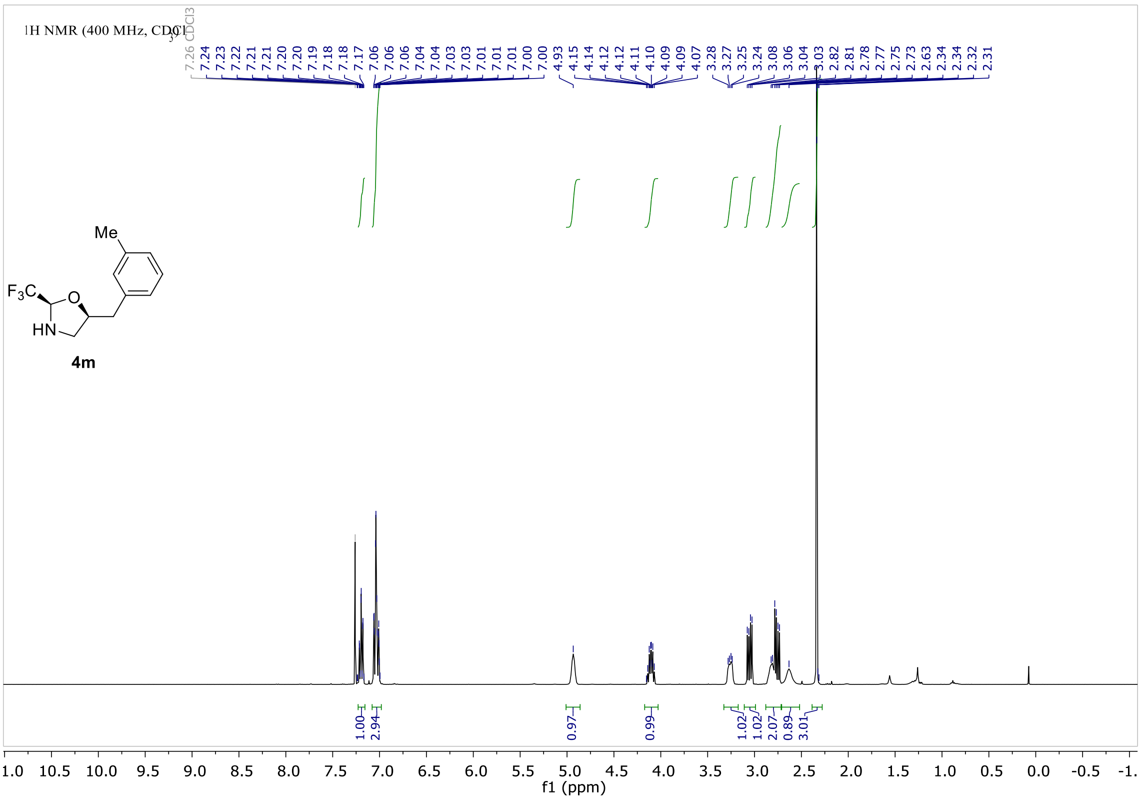
1H NMR (400 MHz, CDCl3)
7.26 CDCl3
7.24
7.23
7.22
7.21
7.21
7.20
7.19
7.18
7.18
7.17
7.06
7.06
7.04
7.04
7.03
7.03
7.01
7.01
7.00
7.00
4.93
4.15
4.14
4.12
4.12
4.11
4.10
4.09
4.09
4.07
3.28
3.27
3.25
3.24
3.08
3.06
3.04
3.03
2.82
2.81
2.78
2.77
2.75
2.73
2.63
2.34
2.34
2.32
2.31
Me
F3C
O
HN
4m
1.00
2.94
0.97
0.99
1.02
1.02
2.07
0.89
3.01
f1 (ppm)

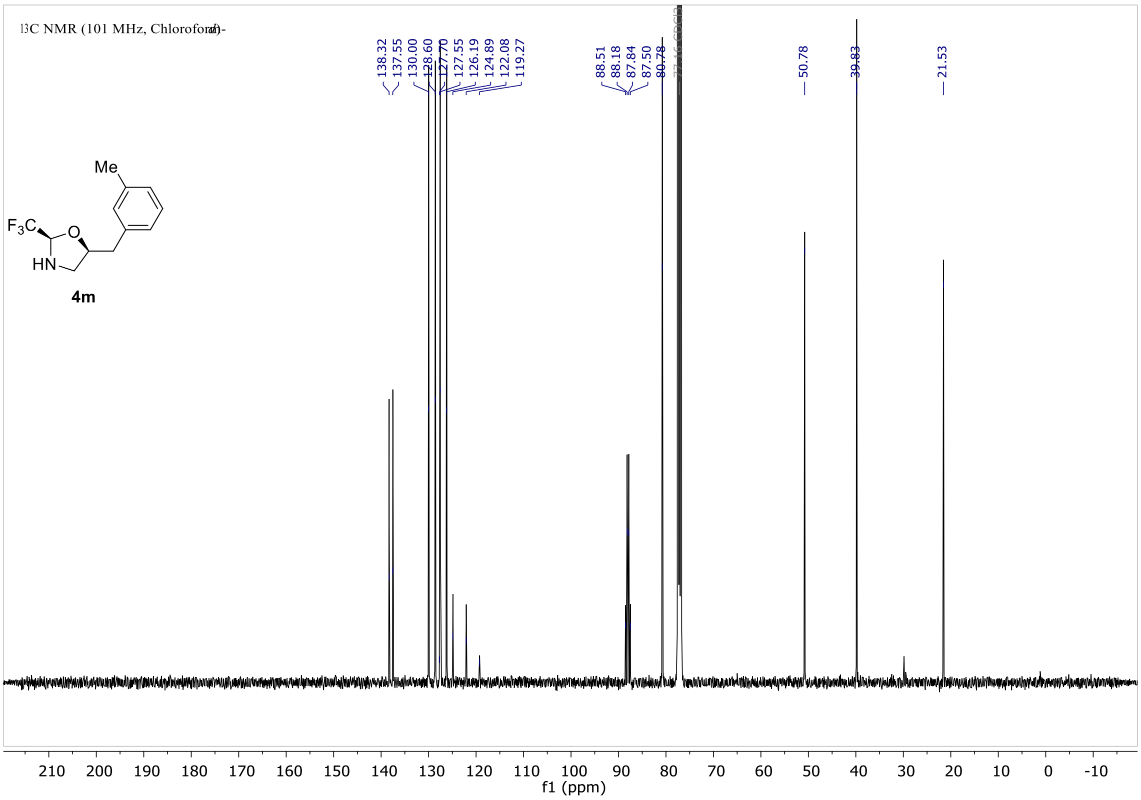
13C NMR (101 MHz, Chloroform-d)
138.32
137.55
130.00
128.60
127.70
127.55
126.19
124.89
122.08
119.27
88.51
88.18
87.84
87.50
80.78
50.78
39.83
21.53
Me
F3C
O
HN
4m
f1 (ppm)

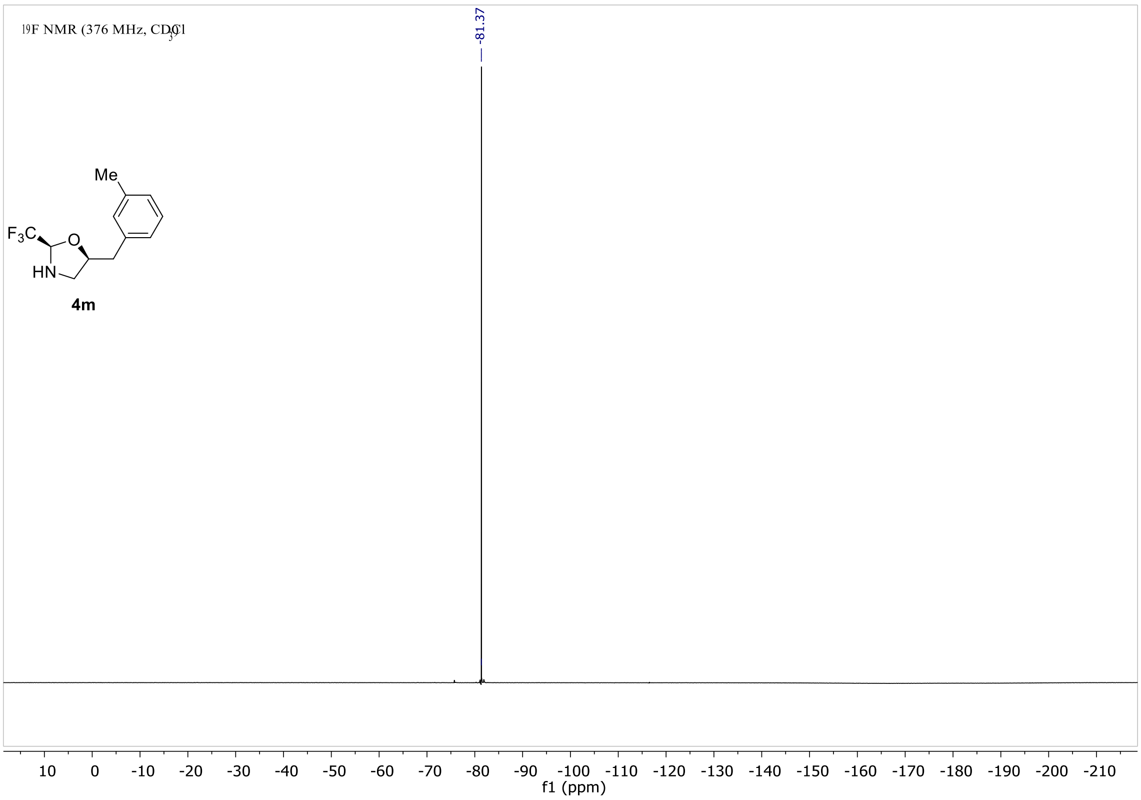
19F NMR (376 MHz, CDCl3)
-81.37
Me
F3C
O
HN
4m
f1 (ppm)

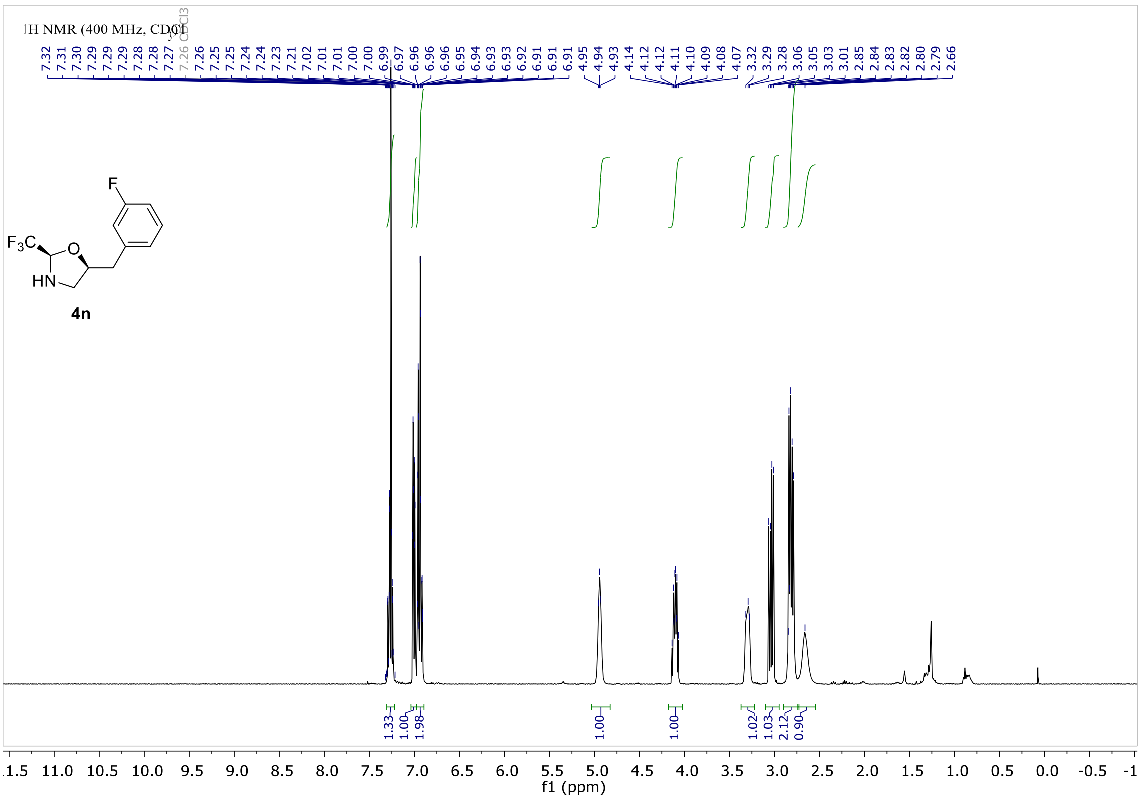
1H NMR (400 MHz, CDCl3)
4n
f1 (ppm)

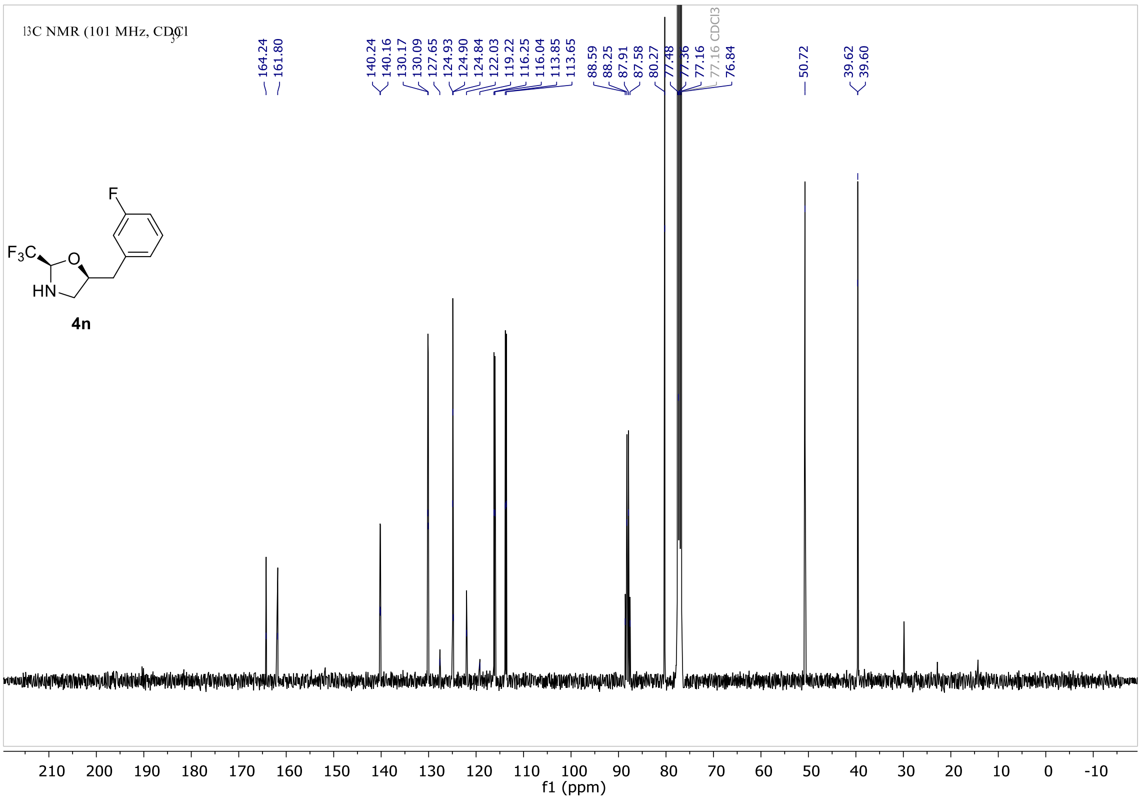
13C NMR (101 MHz, CDCl3)
4n
f1 (ppm)

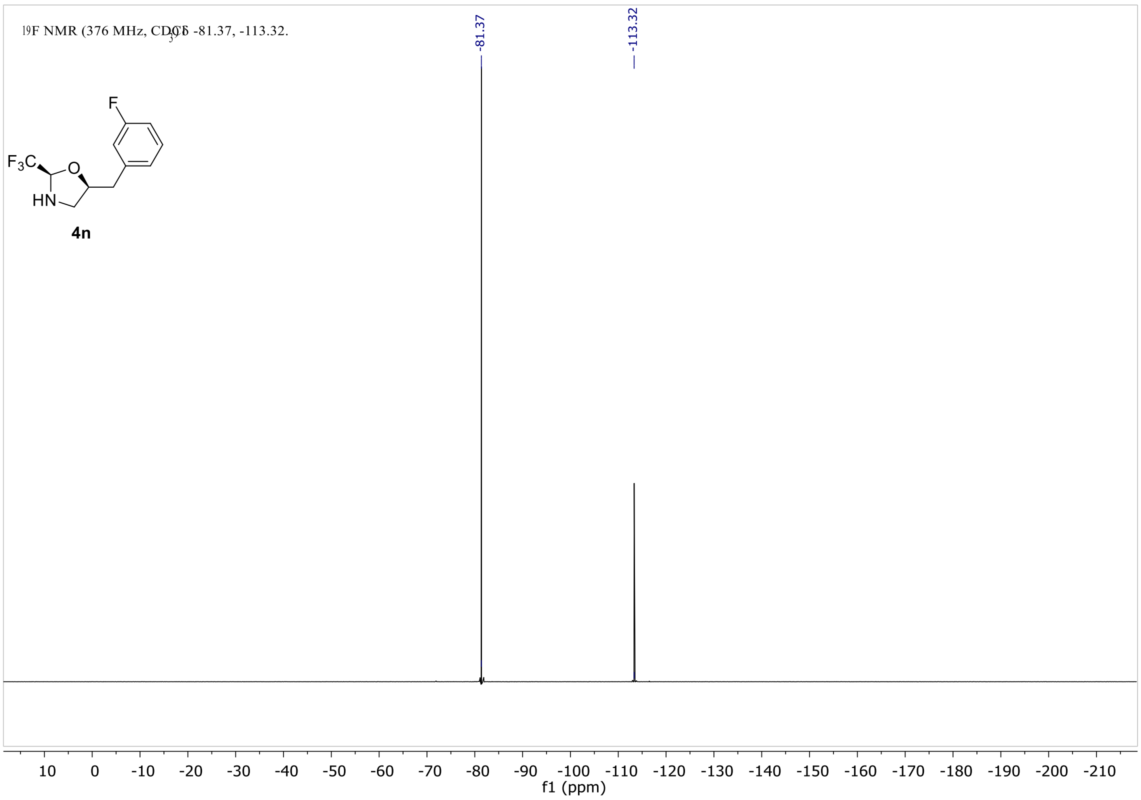
19F NMR (376 MHz, CDCl3) δ -81.37, -113.32.
-81.37
-113.32
4n
f1 (ppm)

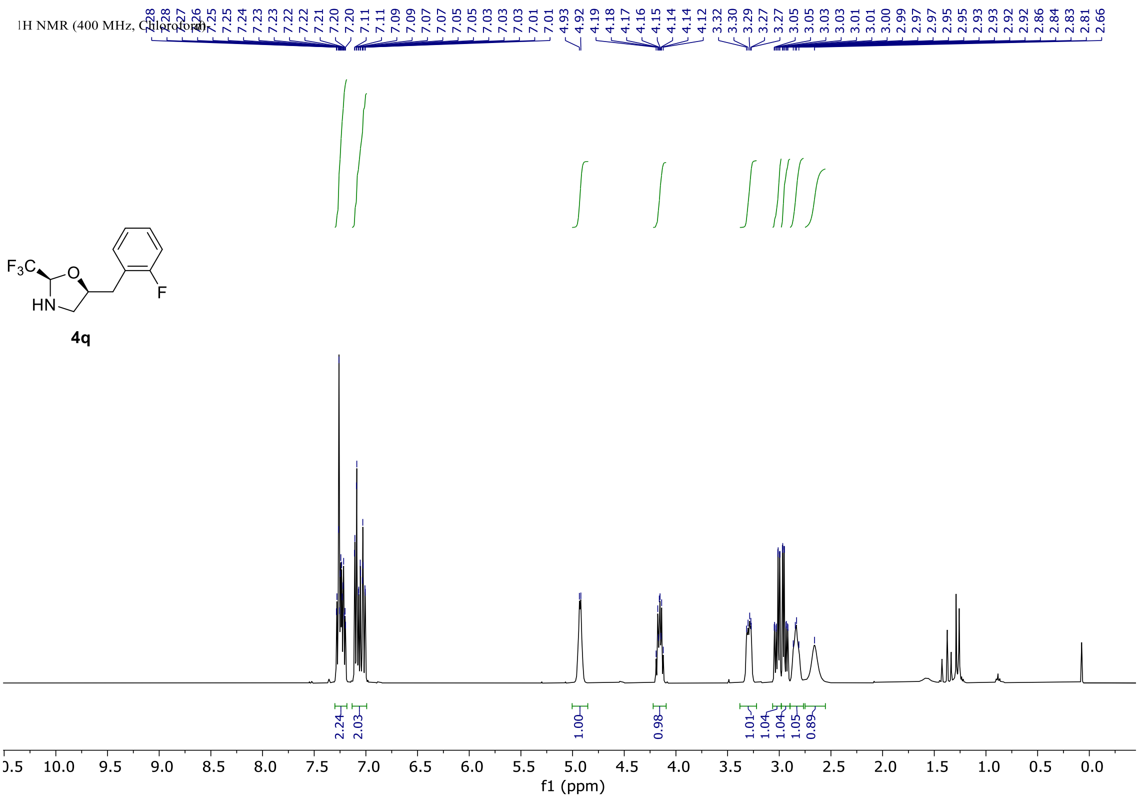
1H NMR (400 MHz,
4q
2.24
2.03
1.00
0.98
1.01
1.04
1.04
1.05
0.89
f1 (ppm)

13C NMR (101 MHz, Chloroform-d)

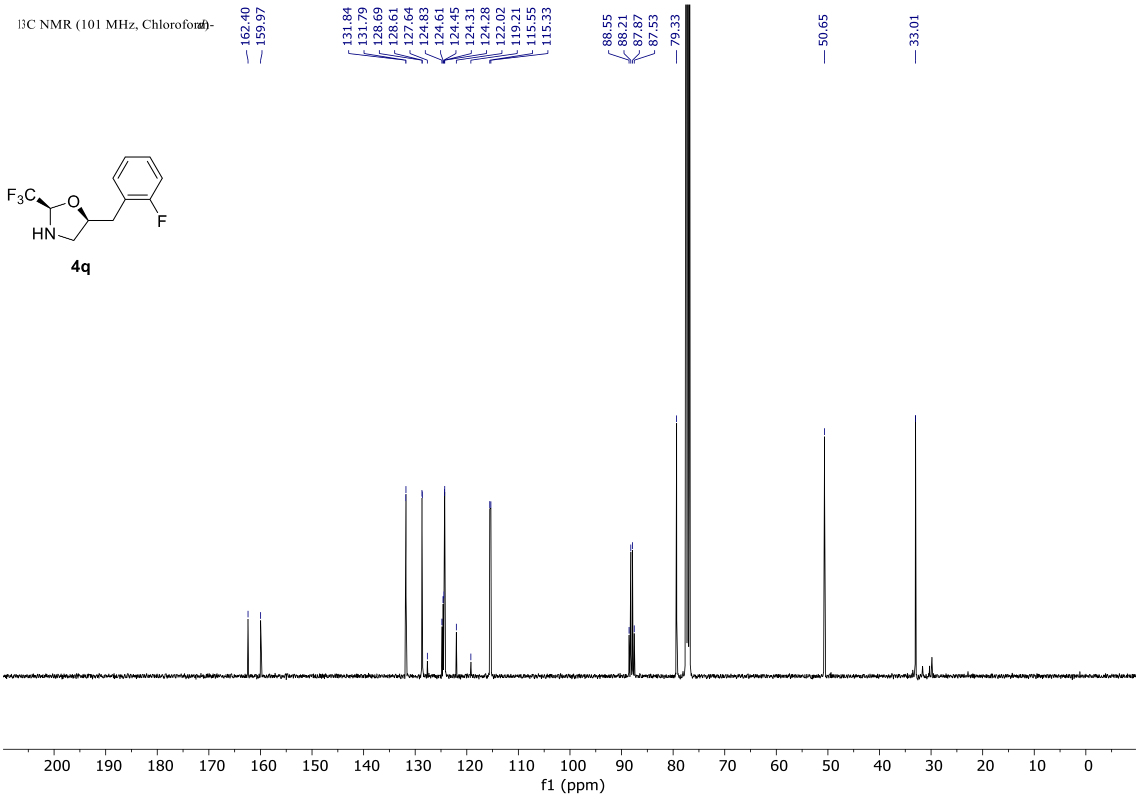

19F NMR (376 MHz, Chloroform-d)

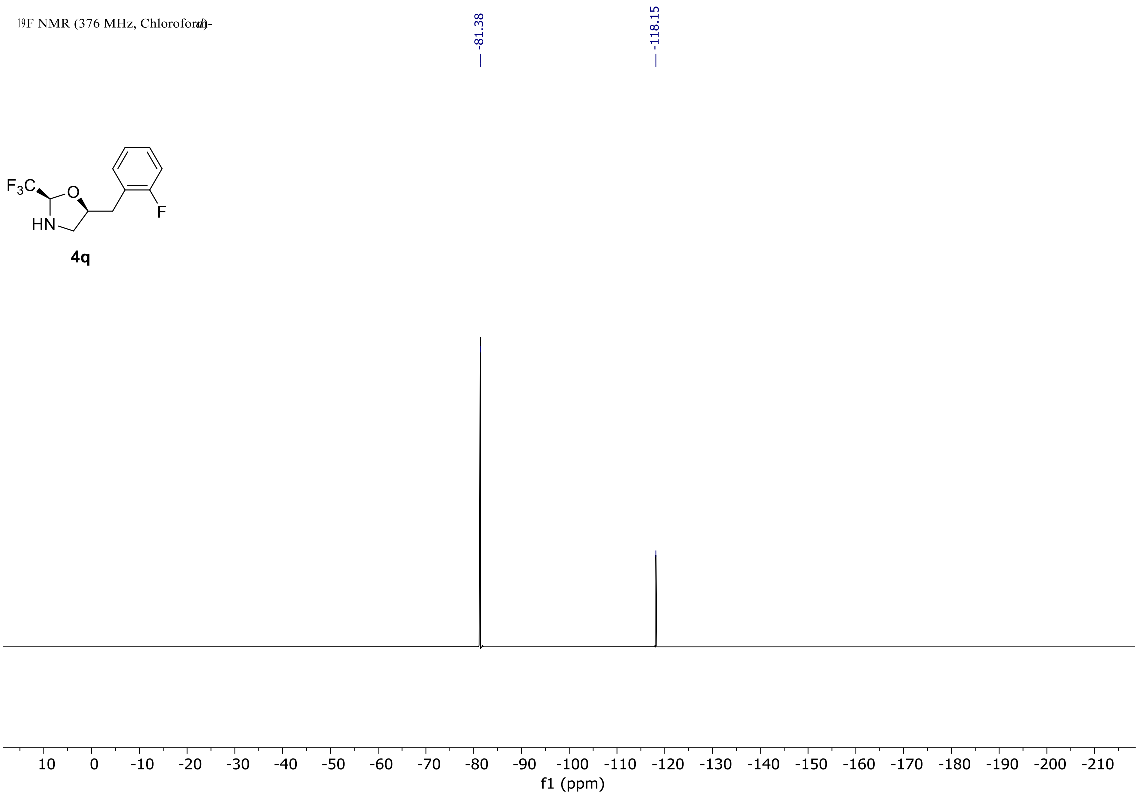

1H NMR (400 MHz, Chloroform-d)

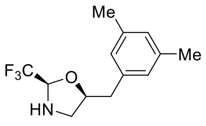

4r

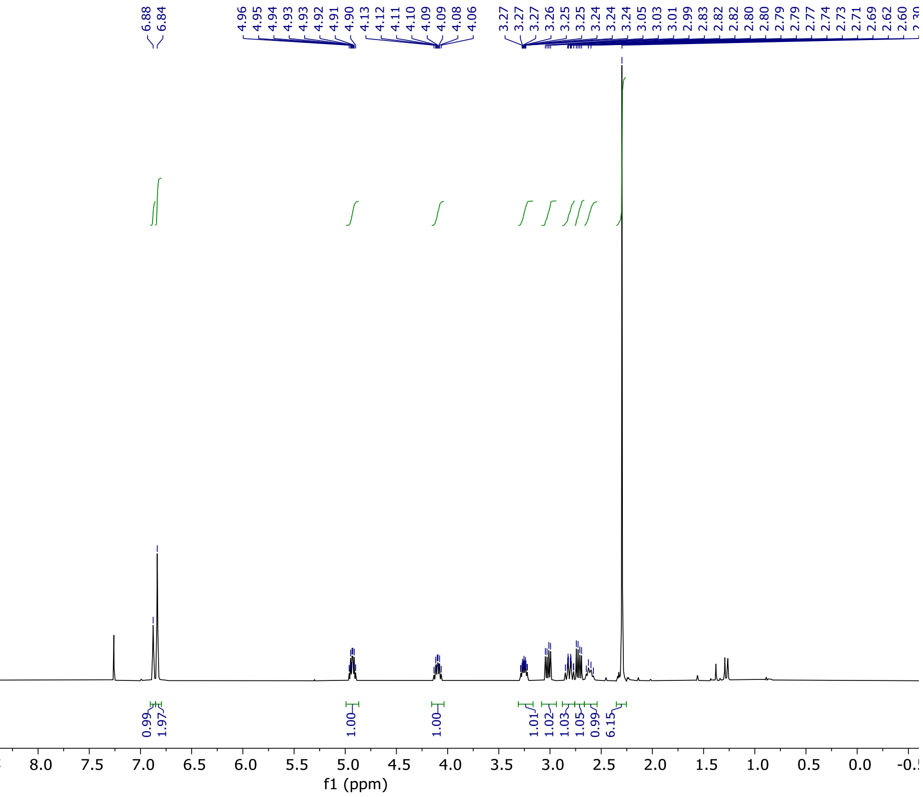

13C NMR (101 MHz, Chloroform-d)

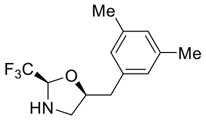

4r

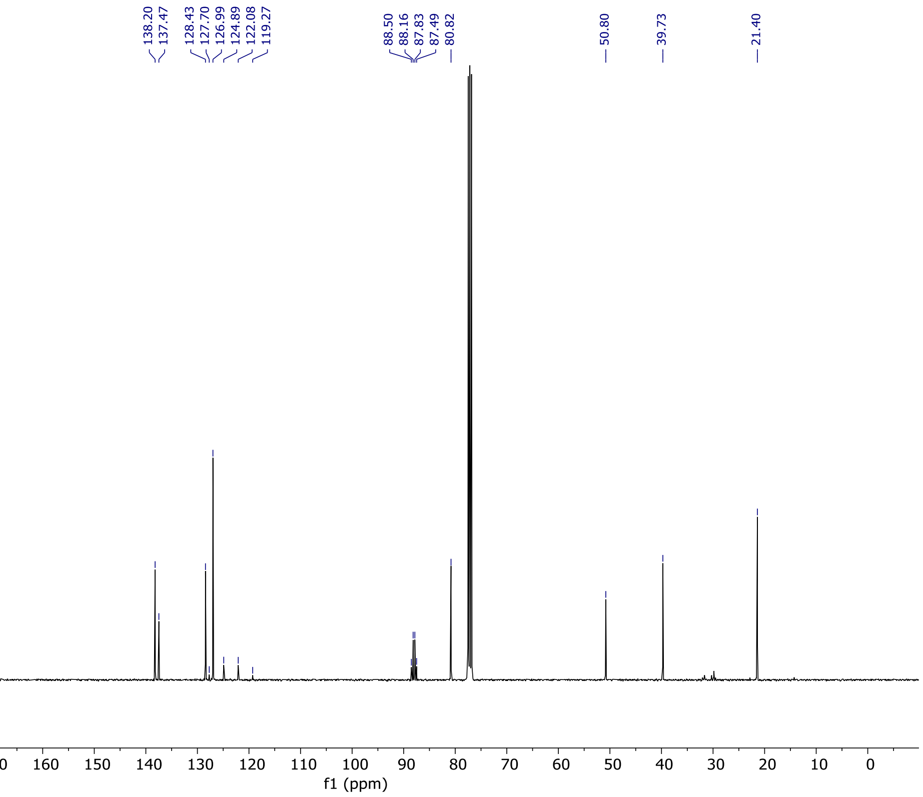

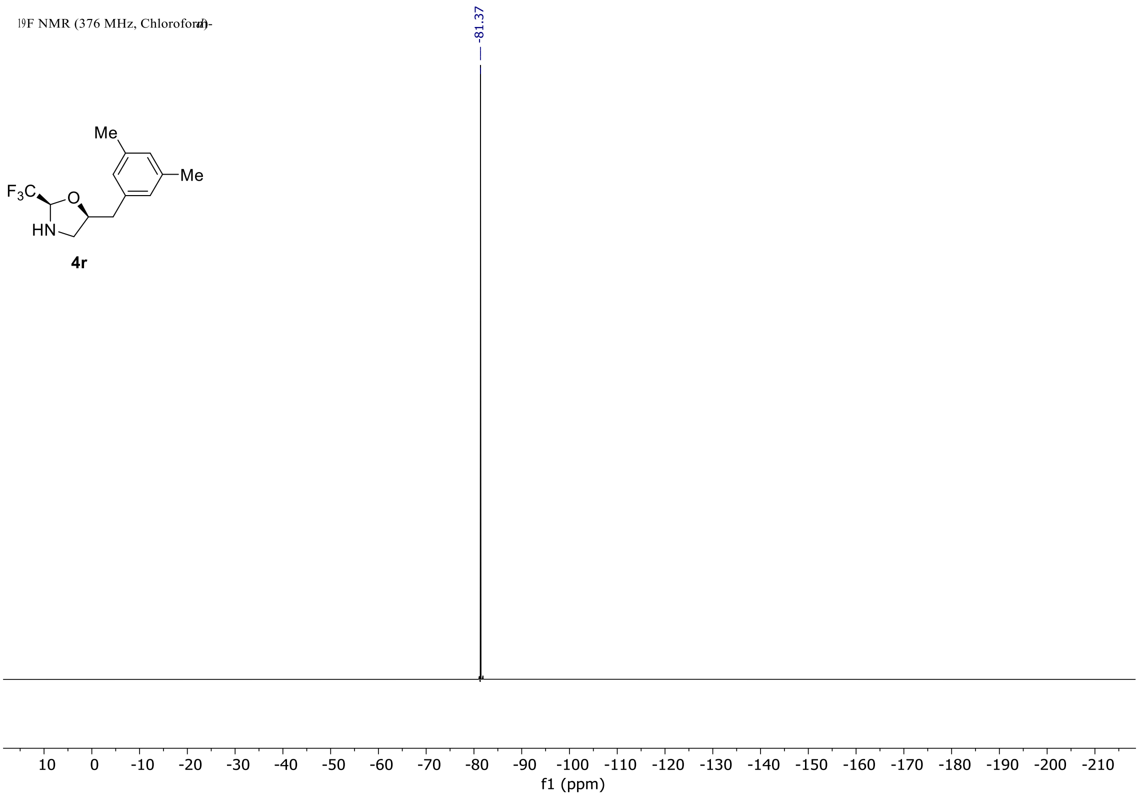
19F NMR (376 MHz, Chloroform-d)
-81.37
F3C
O
HN
Me
Me
4r
f1 (ppm)

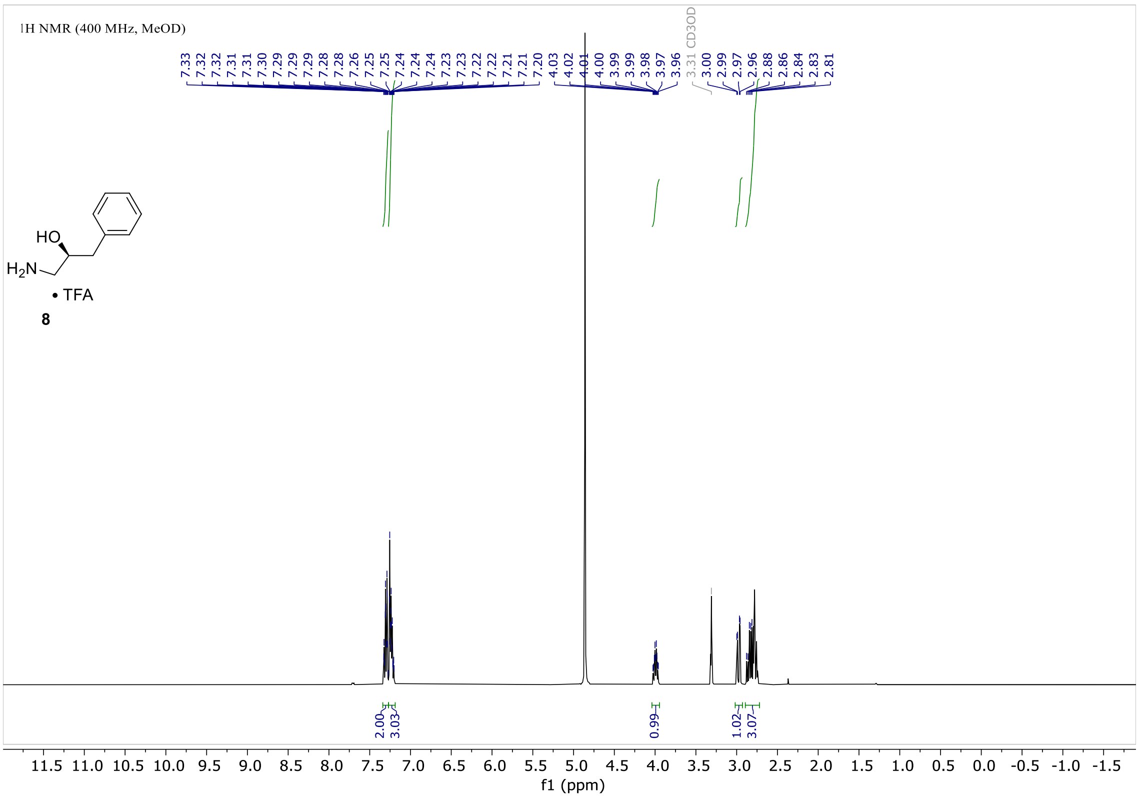
1H NMR (400 MHz, MeOD)
HO
H2N
• TFA
8
3.31 CD3OD
f1 (ppm)

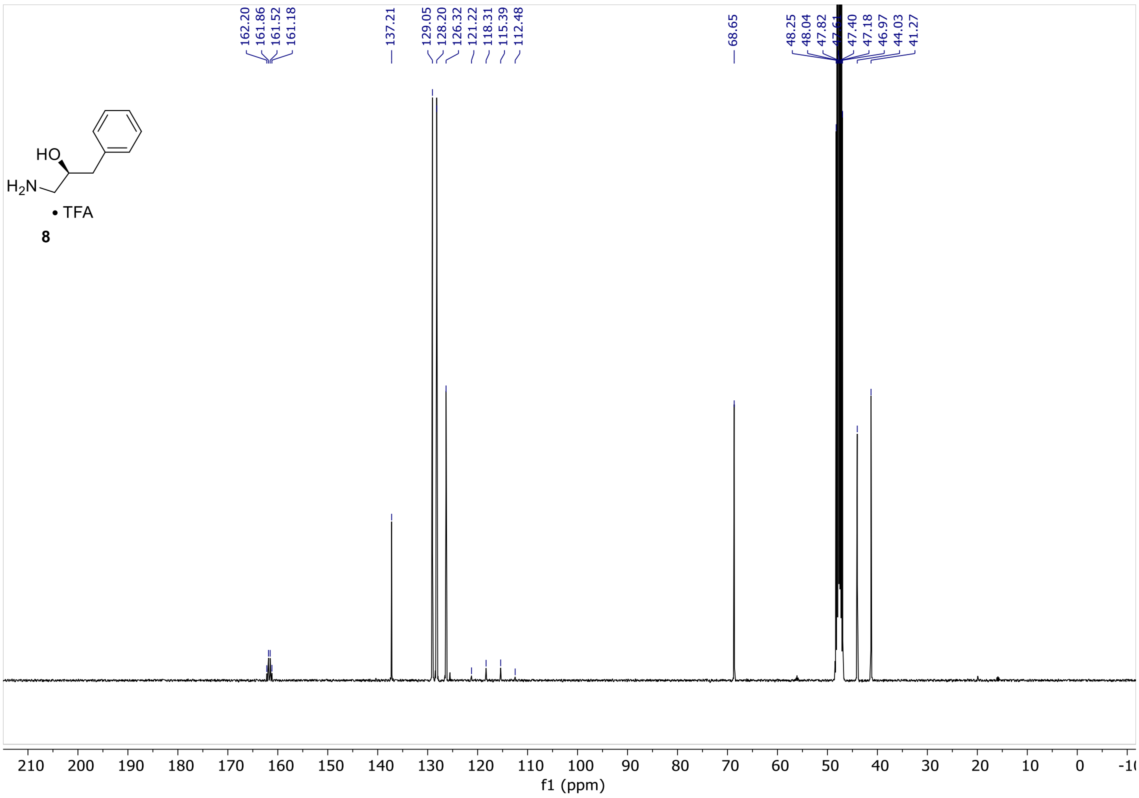
162.20
161.86
161.52
161.18
137.21
129.05
128.20
126.32
121.22
118.31
115.39
112.48
68.65
48.25
48.04
47.82
47.61
47.40
47.18
46.97
44.03
41.27
HO
H2N
• TFA
8
f1 (ppm)

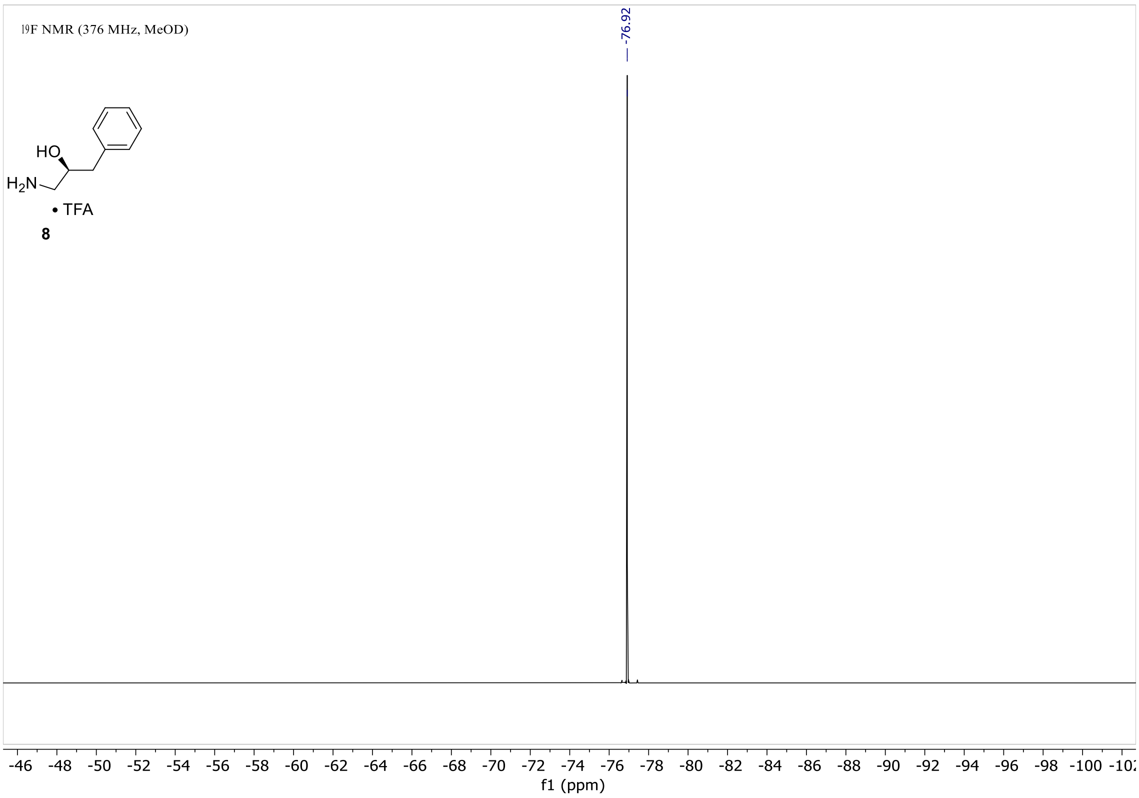
19F NMR (376 MHz, MeOD)
-76.92
HO
H2N
• TFA
8
f1 (ppm)
